# Ligand-independent EPHA2 signaling sustains neural progenitors via ECSIT and NAD^+^

**DOI:** 10.1038/s44319-026-00858-6

**Published:** 2026-07-10

**Authors:** Tran Diem Nghi, Truong Thi My Nhung, Seunghyun Kim, Jonghyeok Yun, Nguyen Tran Nam Tien, Ara Lee, Minkyo Jung, Eun Jin Jang, Bon Seong Goo, Jin Yeong Yoo, Sung Ryong Shin, Ji Young Mun, Kyeng Min Park, Nguyen Phuoc Long, Cana Park, Sang Ki Park

**Affiliations:** 1 Department of Life Sciences, Pohang University of Science and Technology, Pohang, Republic of Korea; 2 Department of Biology, University of Massachusetts Amherst, Amherst, MA USA; 3 Department of Pharmacology and PharmacoGenomics Research Center, Inje University College of Medicine, Busan, Republic of Korea; 4 Department of Biochemistry, Daegu Catholic University School of Medicine, Daegu, Republic of Korea; 5 Neural Circuit Research Group, Korea Brain Research Institute, Daegu, Republic of Korea; 6 Department of Psychiatry and Behavioral Sciences, Johns Hopkins University School of Medicine, Baltimore, MD USA; 7 Graduate Institute of Biomedical Sciences, College of Medicine, Chang Gung University, Taoyuan, Taiwan; 8 Molecular Medicine Research Center, Chang Gung University, Taoyuan, Taiwan

**Keywords:** Metabolism, Neuroscience

## Abstract

Embryonic neural stem and progenitor cells occupy a specialized niche along the lateral ventricles, where receptors on their apical membrane sense extracellular cues essential for preserving progenitor identity. How such surface signals are coupled to mitochondrial metabolism remains unclear. Here, we identify EPHA2 as a receptor enriched in cortical progenitors and show that disruption of its non-canonical, ligand-independent signaling compromises progenitor maintenance in vivo. EPHA2 perturbation reduces mitochondrial respiration, Complex I activity, and mitochondrial NAD^+^ regeneration, and is accompanied by lower mitochondrial abundance of the Complex I assembly factor ECSIT. Restoring mitochondrial NAD^+^ regeneration with MTS-*Lb*NOX, or restoring mitochondrial ECSIT with ECSIT WT—but not a mitochondrial targeting-deficient mutant—attenuates the progenitor defects caused by EPHA2 perturbation. Maternal supplementation with NAD^+^ or its precursor NMN similarly mitigates these developmental defects. We further identify a PP2A-sensitive ECSIT phospho-state, including T179, that is consistent with regulated mitochondrial ECSIT accumulation downstream of EPHA2. Together, these findings support a model in which EPHA2 helps maintain embryonic cortical progenitors by sustaining ECSIT-dependent Complex I-linked mitochondrial redox homeostasis.

## Synopsis

EPHA2 sustains embryonic cortical progenitors by coupling apical receptor signaling to ECSIT-dependent Complex I activity and mitochondrial NAD^+^, providing a functional link between cell surface receptor state and mitochondrial redox control during corticogenesis.

- Ligand-independent EPHA2 signaling is required for cortical progenitor maintenance in vivo.
- EPHA2 perturbation reduces mitochondrial respiration, Complex I activity, NAD^+^ regeneration, and mitochondrial ECSIT abundance.
- Restoring mitochondrial NAD^+^ regeneration or mitochondrial ECSIT attenuates progenitor defects caused by EPHA2 disruption.
- A PP2A-sensitive ECSIT phospho-state, including T179, links EPHA2 signaling to mitochondrial ECSIT accumulation.

## Abstract (web summary)

EPHA2 sustains embryonic cortical progenitors by coupling apical receptor signaling to ECSIT-dependent Complex I activity and mitochondrial NAD^+^, providing a functional link between cell surface receptor state and mitochondrial redox control during corticogenesis.

## Graphical abstract

**Figure:**
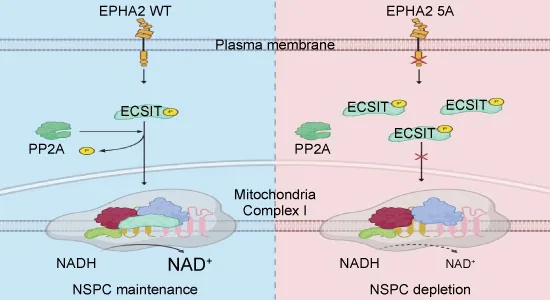

**Figure:**
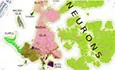

## Introduction

Mammalian neocortical development depends on tightly regulated processes driven by neural stem and progenitor cells (NSPCs) (Götz and Huttner, 2005). During early corticogenesis, radial glial cells in the ventricular zone (VZ) undergo symmetric proliferative divisions to expand the progenitor pool. As development progresses, asymmetric neurogenic divisions become predominant, generating neurons directly or via intermediate progenitors. Newborn neurons then migrate toward the basal pia and organize into the six-layered neocortex in an inside-out manner. NSPCs reside in a stem cell niche in the VZ, where embryonic cerebrospinal fluid-derived factors, such as growth factors and signaling molecules, orchestrate their proliferative capacity, lineage commitment, and timing of differentiation (Eşiyok and Heide, 2023). NSPC access to these cues is largely mediated by surface receptors localized to the apical membrane and primary cilium, including epidermal growth factor receptor (EGFR), insulin-like growth factor receptor (IGFR), and ephrin/erythropoietin-producing hepatoma (EPH) family receptors (Solozobova et al, 2012). Although receptor pathways have been sporadically implicated in NSPC regulation, it remains unclear how receptor state is coupled to intracellular programs that preserve progenitor identity and proliferation.

Mitochondrial dynamics and metabolism have emerged as vital regulators of stem cell behavior (Chakrabarty and Chandel, 2021; Scandella et al, 2023). In the developing cortex, NSPCs are largely glycolytic, yet they remain sensitive to perturbations in mitochondrial electron transport and Complex I function (Scandella et al, 2023). Prior work has extensively defined an intrinsic mitochondrial requirement for NSPC behavior (Cabello-Rivera et al, 2019; Iwata et al, 2020; Khacho et al, 2016; Khacho et al, 2017), but not how extracellular signals impose that requirement with spatial and temporal precision in the progenitor niche. This raises a fundamental unresolved question in developmental neurobiology.

Ephrin type-A receptor 2 (EPHA2) is a candidate receptor for such coupling. EPHA2 belongs to the EPH receptor tyrosine kinase family, a group widely recognized for its roles in neuronal development, cell migration, tissue patterning, and angiogenesis (Rasool and Jahani-Asl, 2024; Wilkinson, 2001). EPHA2 signaling occurs via two modes of action (Miao et al, 2009). The canonical pathway involves ligand binding, primarily by ephrin-A1, leading to receptor autophosphorylation on tyrosine residues. Alternatively, EPHA2 can engage in a distinct, non-canonical mode that does not require ephrin binding. In its ligand-free state, EPHA2 becomes a substrate for various serine/threonine (S/T) kinases that phosphorylate it on multiple consecutive residues (S892, S897, T898, S899, and S901)—with S897 serving as a commonly used readout (Lechtenberg et al, 2021). Phosphorylation of these residues is considered key to regulating the ligand-independent EPHA2 pathway. EPHA2 is highly conserved between humans and mice, exhibiting ~93% amino acid sequence identity (Intoh et al, 2024). Activation of EPHA receptors by ephrin-A1 promotes neuronal differentiation without altering NSPC proliferation (Aoki et al, 2004). Phosphoproteomic analysis of membrane proteins in human NSPCs further identified EPHA2 Ser897 among prominent phosphosites, raising the possibility that ligand-independent EPHA2 signaling is active in these cells (Melo-Braga et al, 2014). However, whether this signaling mode has a physiological role during corticogenesis is unknown.

Here, we asked whether ligand-independent EPHA2 signaling sustains cortical progenitor maintenance by coupling receptor state to mitochondrial function. We show that EPHA2 is enriched in embryonic NSPCs and that disrupting its ligand-independent, but not canonical ligand-dependent, signaling compromises progenitor maintenance together with mitochondrial respiration and Complex I-linked redox function. We further identify an Evolutionarily Conserved Signaling Intermediate in Toll pathways (ECSIT)-dependent downstream mechanism linking EPHA2 signaling to the mitochondrial program that supports progenitor behavior during corticogenesis.

## Results

### EPHA2 is selectively enriched in embryonic cortical progenitors

**Figure 1:**
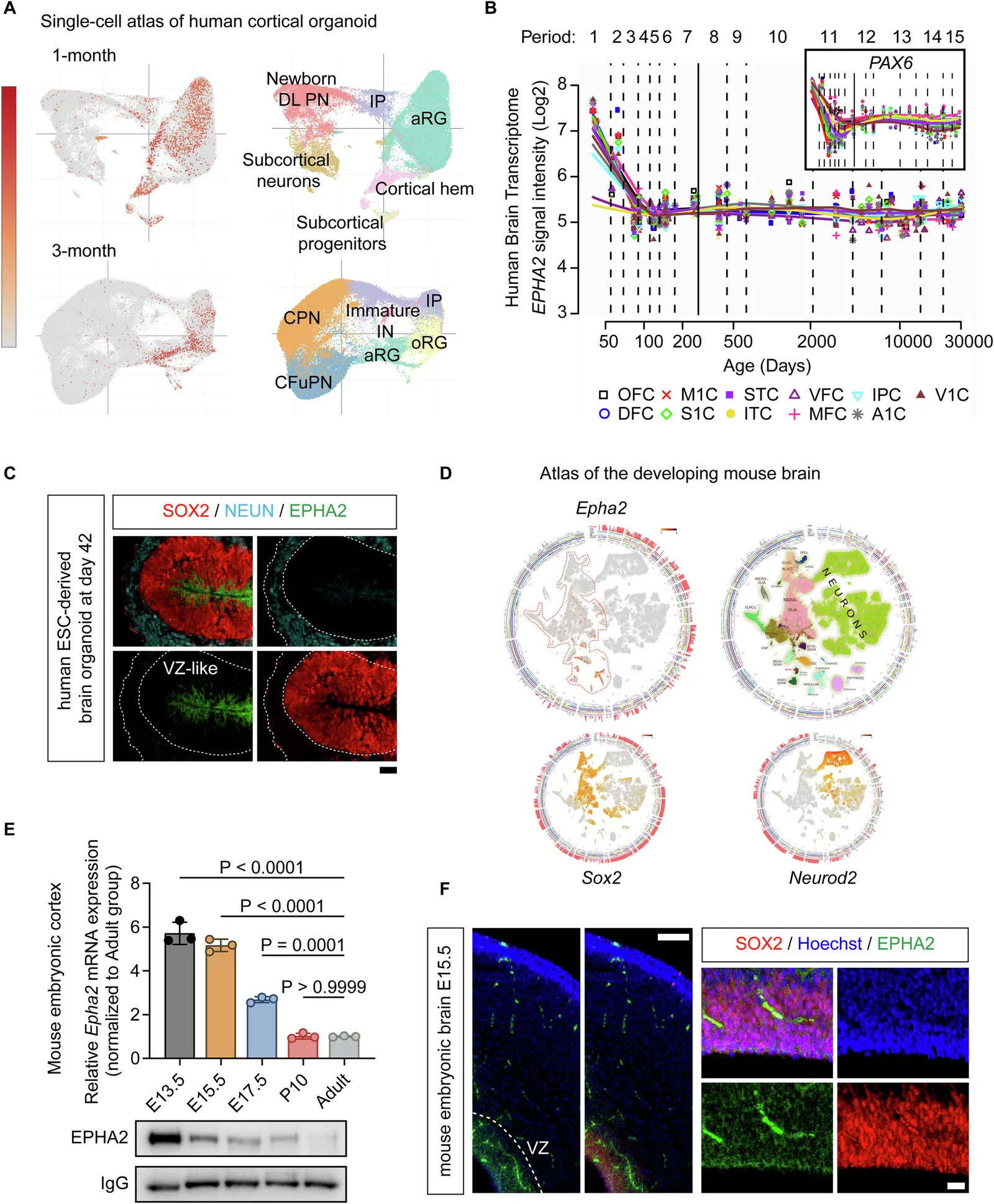
EPHA2 is selectively enriched in embryonic cortical progenitors. (**A**) Single-cell transcriptomic maps of human cortical organoids at 1 month and 3 months, showing *EPHA2* expression across major cell populations. Cells expressing *EPHA2* are highlighted in red. Cells are colored by cell type. aRG apical radial glia, oRG outer radial glia, IP intermediate progenitor, DL PN deep-layer projection neuron, IN interneuron, CPN callosal projection neuron, CFuPN corticofugal projection neuron. Dataset from Uzquiano et al, 2022. (**B**) Temporal transcriptome profiling from the Human Brain Transcriptome project. *EPHA2* signal intensity in neocortex areas during embryonic/fetal development (periods 1–7), postnatal development (periods 8–12), and adulthood (periods 13–15). OFC orbital frontal cortex, DFC dorsolateral frontal cortex, VFC ventrolateral frontal cortex, MFC medial frontal cortex, M1C primary motor cortex, S1C primary somatosensory cortex, V1C primary visual cortex, A1C primary auditory cortex, STC posterior superior cortex, ITC anterior inferior cortex, IPC posterior inferior cortex. Inset highlights the expression dynamics of *PAX6*, an NSPC marker. (**C**) Immunofluorescence of day-42 human embryonic stem cell (ESC)-derived forebrain organoids stained for SOX2, NEUN, and EPHA2, showing EPHA2 enrichment in the SOX2-positive ventricular zone-like region. Dashed lines illustrate the boundary of a single rosette and the region like ventricular zone (VZ). EPHA2, SOX2, and NEUN were co-stained. Scale bar, 30 μm. (**D**) Single-cell transcriptomic mapping of the developing mouse brain. Cell-type clusters expressing *Epha2* are circled by a red dashed line. Expression patterns of *Sox2* and *Neurod2* markers are shown in the bottom panels. Dataset from La Manno et al, 2021. (**E**) Top, qRT-PCR analysis of *Epha2* mRNA across mouse brain development (*n* = 3 embryos). Bottom, immunoprecipitation followed by immunoblotting showing developmental changes in EPHA2 protein abundance. Statistical analysis was performed using one-way ANOVA followed by Tukey’s post hoc test. Quantitative data were presented as mean ± SD. Exact *P* values are indicated above the bars in each quantification plot. (**F**) Immunofluorescence of E15.5 mouse cortex stained for SOX2 and EPHA2, showing enrichment of EPHA2 in the ventricular zone. Scale bars, 100 μm (left), 20 μm (right). Source data are available online for this figure.

To determine whether EPHA2 is positioned to regulate neural stem and progenitor cells (NSPCs) during corticogenesis, we first examined its spatiotemporal expression. Single-cell transcriptomic analysis of human cortical organoids (Uzquiano et al, 2022) revealed enriched *EPHA2* expression in progenitor populations, including apical and outer radial glia and intermediate progenitors. In contrast, *EPHA2* was largely absent from immature and mature neurons (Fig. 1A). Human cortical developmental transcriptome data (Kang et al, 2011) showed high *EPHA2* expression during early developmental stages, followed by a postnatal decline, in parallel with the NSPC marker *PAX6* (Fig. 1B). Consistent with these datasets, immunostaining of day-42 human embryonic stem cell-derived forebrain organoids supported selective EPHA2 expression in the SOX2^+^ VZ-like region (Fig. 1C). In the developing mouse cortex, single-cell transcriptomic analysis (La Manno et al, 2021) likewise demonstrated *Epha2* in radial glial and other non-neuronal clusters including vascular cells, with comparatively low expression in *Neurod2*-positive neuronal populations (Fig. 1D). Quantitative RT-PCR and immunoblotting showed that *Epha2* expression peaked at embryonic day 13.5 (E13.5) and declined thereafter among the examined developmental time points (Fig. 1E). Immunostaining of brains at E15.5 confirmed EPHA2 enrichment in the VZ, beyond known vascular expression (Fig. 1F), in line with the transcriptomic profile. Together, these data identify EPHA2 as a receptor preferentially expressed in embryonic cortical progenitors.

### Ligand-independent EPHA2 signaling is required for progenitor maintenance

**Figure EV1:**
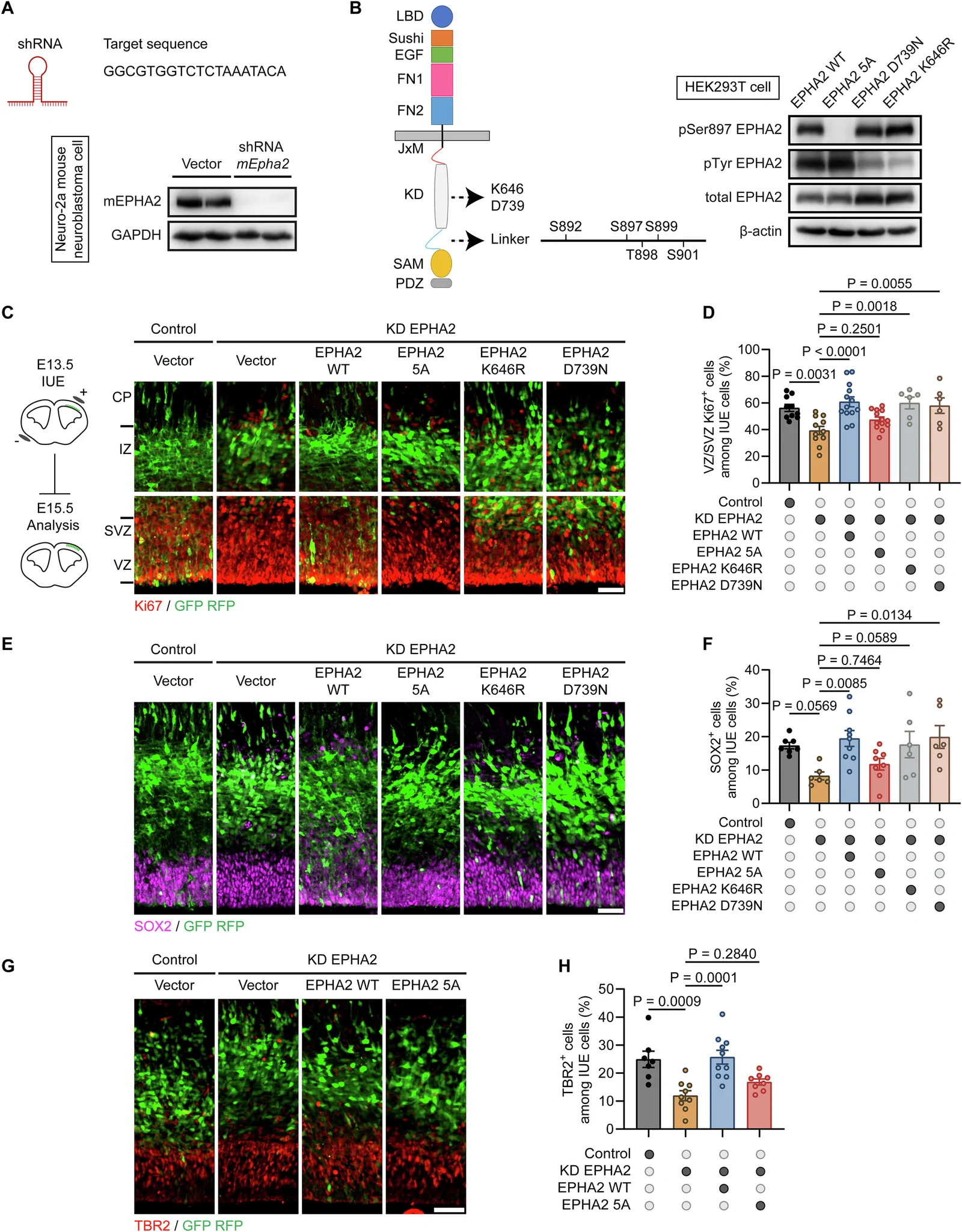
Additional validation of ligand-independent EPHA2 signaling in embryonic NSPCs. (**A**) Immunoblot validation of shRNA-mediated knockdown of endogenous mouse *Epha2* in Neuro-2a cells. (**B**) Schematic of EPHA2 domain structure and mutants used to selectively disrupt ligand-dependent (K646R and D739N) or ligand-independent (5A) signaling, with corresponding phospho-Ser897 EPHA2 and phospho-tyrosine immunoblots after expression in HEK293T cells. LBD ligand-binding domain, EGF epidermal growth factor-like, FN1 and FN2 fibronectin type III, JxM juxtamembrane segment, KD kinase domain, SAM sterile alpha motif, PDZ PDZ-binding motif. (**C**–**H**) IUE at E13.5 with EGFP-empty vector (control), EGFP-shRNA targeting mouse *Epha2* (KD EPHA2) together with either RFP-empty vector, EPHA2 WT, EPHA2 5A, EPHA2 K646R, or EPHA2 D739N. Analysis at E15.5. (**C**) GFP RFP (green) and Ki67 (red) triple IF. Scale bar, 50 μm. (**D**) Quantification of Ki67-positive electroporated cells (Control *n* = 10, KD EPHA2 *n* = 11, KD EPHA2 + EPHA2 WT *n* = 13, KD EPHA2 + EPHA2 5A *n* = 13, KD EPHA2 + EPHA2 K646R *n* = 6, KD EPHA2 + EPHA2 D739N *n* = 6 embryos). (**E**) GFP RFP (green) and SOX2 (magenta) triple IF. Scale bar, 50 μm. (**F**) Quantification of SOX2-positive electroporated cells (Control *n* = 7, KD EPHA2 *n* = 6, KD EPHA2 + EPHA2 WT *n* = 9, KD EPHA2 + EPHA2 5A *n* = 8, KD EPHA2 + EPHA2 K646R *n* = 6, KD EPHA2 + EPHA2 D739N *n* = 6 embryos). (**G**) GFP RFP (green) and TBR2 (red) triple IF. Scale bar, 50 μm. (**H**) Quantification of TBR2-positive electroporated cells (Control *n* = 7, KD EPHA2 *n* = 9, KD EPHA2 + EPHA2 WT *n* = 10, KD EPHA2 + EPHA2 5A *n* = 8 embryos). Statistical analysis was performed using one-way ANOVA followed by Tukey’s post hoc test (**D**, **F**, **H**). Quantitative data were presented as mean ± SD. Exact *P* values are indicated above the bars in each quantification plot.

**Figure 2:**
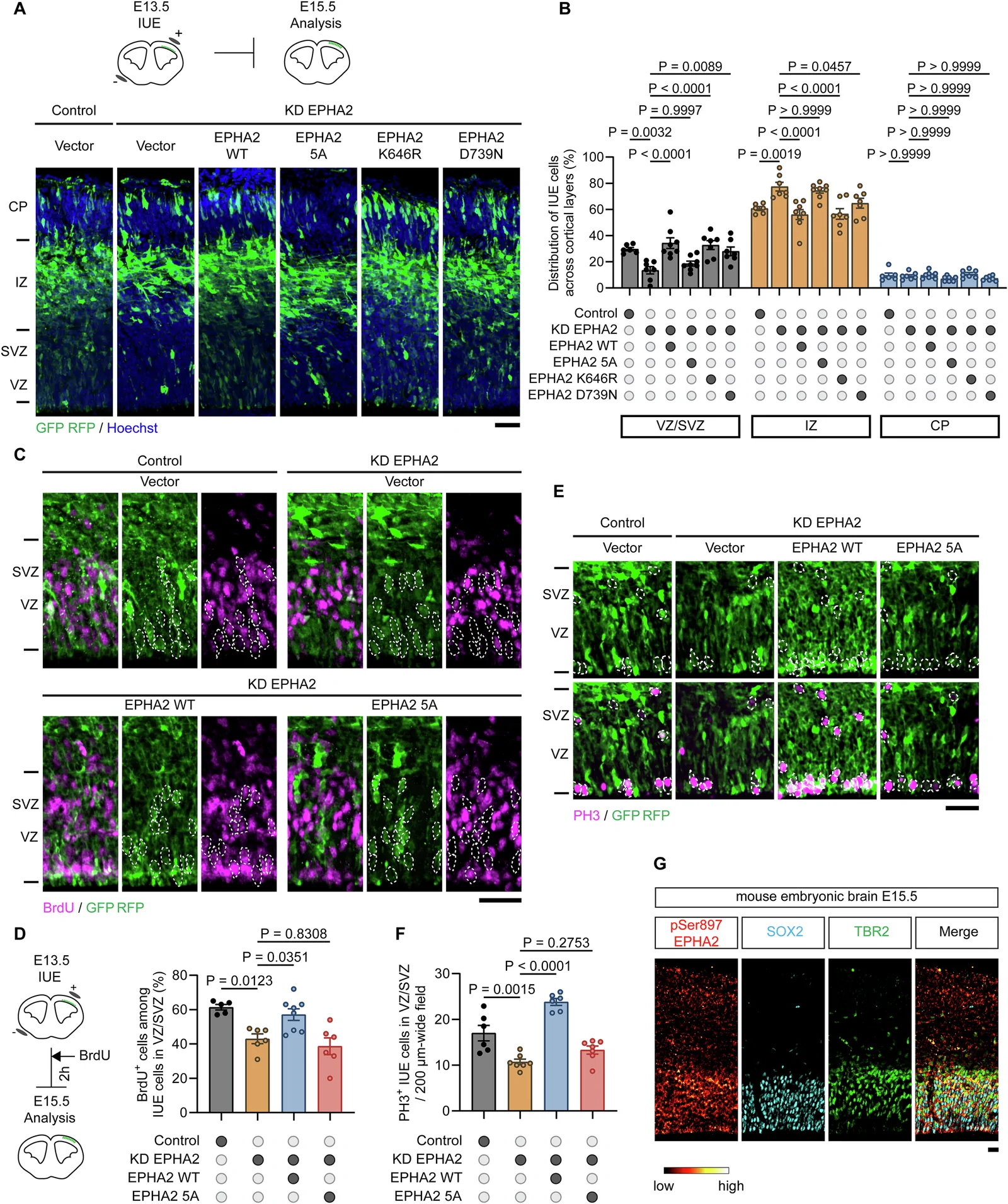
Ligand-independent EPHA2 signaling is required for progenitor maintenance. IUE at E13.5 with EGFP-empty vector (control), EGFP-shRNA targeting mouse *Epha2* (KD EPHA2) together with either RFP-empty vector, EPHA2 WT, EPHA2 5A, EPHA2 K646R, or EPHA2 D739N. Analysis at E15.5. (**A**) GFP RFP electroporated cells are shown relative to cortical layers defined by Hoechst density. Scale bar, 50 μm. (**B**) Quantification of the distribution of electroporated cells across VZ/SVZ, IZ, and CP under the indicated conditions, showing rescue by EPHA2 WT and kinase-dead mutants, but not by EPHA2 5A (Control *n *= 6, KD EPHA2 *n* = 7, KD EPHA2 + EPHA2 WT *n* = 8, KD EPHA2 + EPHA2 5A *n* = 8, KD EPHA2 + EPHA2 K646R *n* = 7, KD EPHA2 + EPHA2 D739N *n* = 7 embryos). (**C**) GFP RFP (green) and BrdU (magenta) triple IF. White dashed lines highlight IUE cells that are either BrdU-positive or BrdU-negative. Scale bar, 50 μm. (**D**) Quantification of S-phase BrdU-positive electroporated cells in the VZ/SVZ (Control *n* = 5, KD EPHA2 *n* = 6, KD EPHA2 + EPHA2 WT *n* = 8, KD EPHA2 + EPHA2 5A *n* = 6 embryos). (**E**) GFP RFP (green) and PH3 (magenta) triple IF. White dashed lines highlight IUE cells that are either PH3-positive or PH3-negative. Scale bar, 50 μm. (**F**) Quantification of mitotic PH3-positive electroporated cells in the VZ/SVZ (Control *n* = 6, KD EPHA2 *n* = 7, KD EPHA2 + EPHA2 WT *n* = 6, KD EPHA2 + EPHA2 5A *n* = 7 embryos). (**G**) Immunofluorescence of E15.5 mouse cortex stained for phospho-Ser897 EPHA2, SOX2, and TBR2, showing enrichment of ligand-independent EPHA2 signaling in the progenitor zone. Scale bar, 20 μm. Statistical analysis was performed using two-way ANOVA with Šídák’s multiple comparisons test (**B**) and one-way ANOVA followed by Tukey’s post hoc test (**D**, **F**). Quantitative data are presented as mean ± SD. Exact *P* values are indicated above the bars in each quantification plot. Source data are available online for this figure.

Given its enrichment in progenitors, we next assessed how the two distinct EPHA2 activation modes influence NSPC behavior in vivo. Mouse *Epha2* was knocked down (KD) by shRNA, and efficient depletion was confirmed by immunoblotting (Fig. EV1A). We evaluated the resulting phenotype in an E13.5 in utero electroporation (IUE) rescue paradigm using EPHA2 WT or signaling-selective mutants (Fig. 2A). K646R and D739N are kinase-dead mutants that suppress canonical ligand-dependent EPHA2 signaling but preserve the ligand-independent signaling state. By contrast, EPHA2 5A is a phosphosite mutant that selectively disrupts the ligand-independent pathway. These distinct properties were validated by phosphorylation at Ser897, a commonly used readout of ligand-independent signaling, and tyrosine phosphorylation, indicative of canonical ligand-dependent EPHA2 activation (Fig. EV1B). At E15.5, KD EPHA2 reduced the proportion of electroporated cells in the ventricular/subventricular zone (VZ/SVZ) and increased their accumulation in the intermediate zone (IZ). This laminar distribution defect was rescued by EPHA2 WT, K646R, and D739N, but not by EPHA2 5A (Fig. 2A,B). These data indicate that progenitor maintenance depends on ligand-independent rather than on canonical ligand-dependent EPHA2 signaling.

This conclusion was reinforced by assessing direct proliferative capacity within the progenitor zone. S-phase proliferating cells were pulse-labeled with BrdU two hours prior to tissue collection at E15.5, revealing a reduced fraction of S-phase electroporated cells in the VZ/SVZ following EPHA2 depletion. This defect was restored by WT but not by the 5A mutant (Fig. 2C,D). Similarly, the proportion of PH3^+^ mitotic electroporated cells in the VZ/SVZ was diminished upon knockdown and rescued only by WT (Fig. 2E,F). Additional analyses demonstrated a decline in Ki67^+^ actively cycling cells, accompanied by depletion of both SOX2^+^ radial glial cell (RGC) and TBR2^+^ intermediate progenitor (IP) populations after KD EPHA2, which could not be rescued by EPHA2 5A re-expression (Fig. EV1C–H). In parallel, phospho-Ser897 EPHA2 was prominently detected in the VZ/SVZ where SOX2^+^ and TBR2^+^ NSPCs reside, supporting the presence of ligand-independent EPHA2 signaling in the progenitor zone (Fig. 2G). Together, these findings indicate that ligand-independent EPHA2 signaling sustains both apical RGC and basal IP pools, highlighting its critical role in NSPC maintenance.

### Loss of ligand-independent EPHA2 signaling promotes VZ/SVZ cell cycle exit and alters cortical projection neuron composition

**Figure 3:**
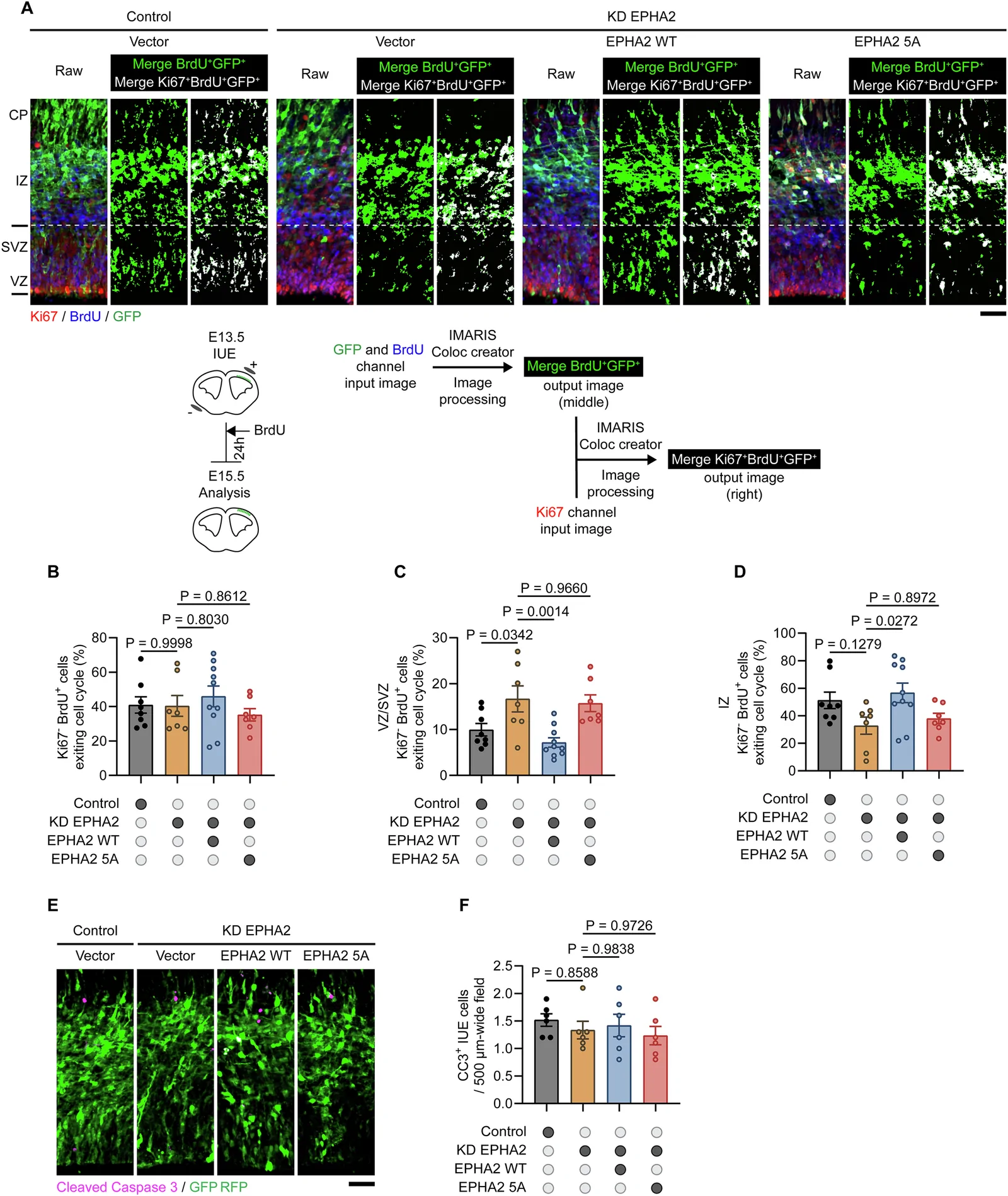
Loss of ligand-independent EPHA2 signaling promotes VZ/SVZ cell cycle exit. (**A**–**D**) IUE at E13.5 with EGFP-empty vector (control), EGFP-KD EPHA2 together with either EGFP-empty vector, EPHA2 WT, or EPHA2 5A. BrdU administration at E14.5 and tissue collection at E15.5. (**A**) BrdU/Ki67 labeling scheme and representative images used to assess cell cycle exit after IUE. GFP (green), BrdU (blue), and Ki67 (red) triple IF. Co-localized areas between GFP and BrdU, as well as between GFP, BrdU, and Ki67, were pseudo-colored to green and white, respectively, using Imaris software. The VZ/SVZ and IZ were defined based on differences in cellular density, as visualized by Hoechst nuclear staining. Scale bar, 50 μm. (**B**) Quantification of Ki67-negative BrdU-positive electroporated cells across the entire brain region (Control *n* = 8, KD EPHA2 *n* = 7, KD EPHA2 + EPHA2 WT *n* = 10, KD EPHA2 + EPHA2 5A *n* = 7 embryos). (**C**) Quantification of Ki67-negative BrdU-positive electroporated cells within the VZ/SVZ (Control *n* = 8, KD EPHA2 *n* = 7, KD EPHA2 + EPHA2 WT *n* = 10, KD EPHA2 + EPHA2 5A *n* = 7 embryos). (**D**) Quantification of Ki67-negative BrdU-positive electroporated cells within the IZ (Control *n* = 8, KD EPHA2 *n* = 7, KD EPHA2 + EPHA2 WT *n* = 10, KD EPHA2 + EPHA2 5A *n* = 7 embryos). (**E**,** F**) IUE at E13.5 with EGFP-empty vector (control), EGFP-KD EPHA2 together with either RFP-empty vector, EPHA2 WT, or EPHA2 5A. Analysis at E15.5. (**E**) GFP RFP (green) and cleaved Caspase 3 (CC3) (magenta) triple IF. Scale bar, 50 μm. (**F**) Quantification of cleaved Caspase 3-positive electroporated cells expressed per 500-μm-wide microscopic field (Control *n* = 6, KD EPHA2 *n* = 6, KD EPHA2 + EPHA2 WT *n* = 6, KD EPHA2 + EPHA2 5A *n* = 6 embryos). Statistical analysis was performed using one-way ANOVA followed by Tukey’s post hoc test (**B**–**D**, **F**). Quantitative data are presented as mean ± SD. Exact *P* values are indicated above the bars in each quantification plot. Source data are available online for this figure.

Reduced NSPC proliferation may reflect either premature cell cycle exit or increased apoptosis. BrdU/Ki67 co-labeling was first performed to assess cell cycle exit (Fig. 3A). BrdU injection at E14.5 labels S-phase cells during a 24-h pulse, while Ki67 marks actively cycling cells at the time of analysis at E15.5. Ki67^−^ BrdU^+^ cells thus represent those that exited the cell cycle within this labeling interval. Although overall cortical cell cycle exit was unchanged, region-specific analysis showed an increased fraction of Ki67^−^ BrdU^+^ electroporated cells in the VZ/SVZ after KD EPHA2. This defect was rescued by EPHA2 WT but not by EPHA2 5A, indicating that ligand-independent EPHA2 signaling restrains cell cycle exit within the progenitor zone (Fig. 3B,C). Conversely, EPHA2 5A reduced the apparent exit fraction in the IZ (Fig. 3D), consistent with the enhanced displacement from the VZ/SVZ and accumulation within the IZ observed in the laminar distribution analysis. Cleaved caspase 3 staining was unchanged across conditions, ruling out apoptosis as a factor affecting cell cycle progression (Fig. 3E,F).

**Figure EV2:**
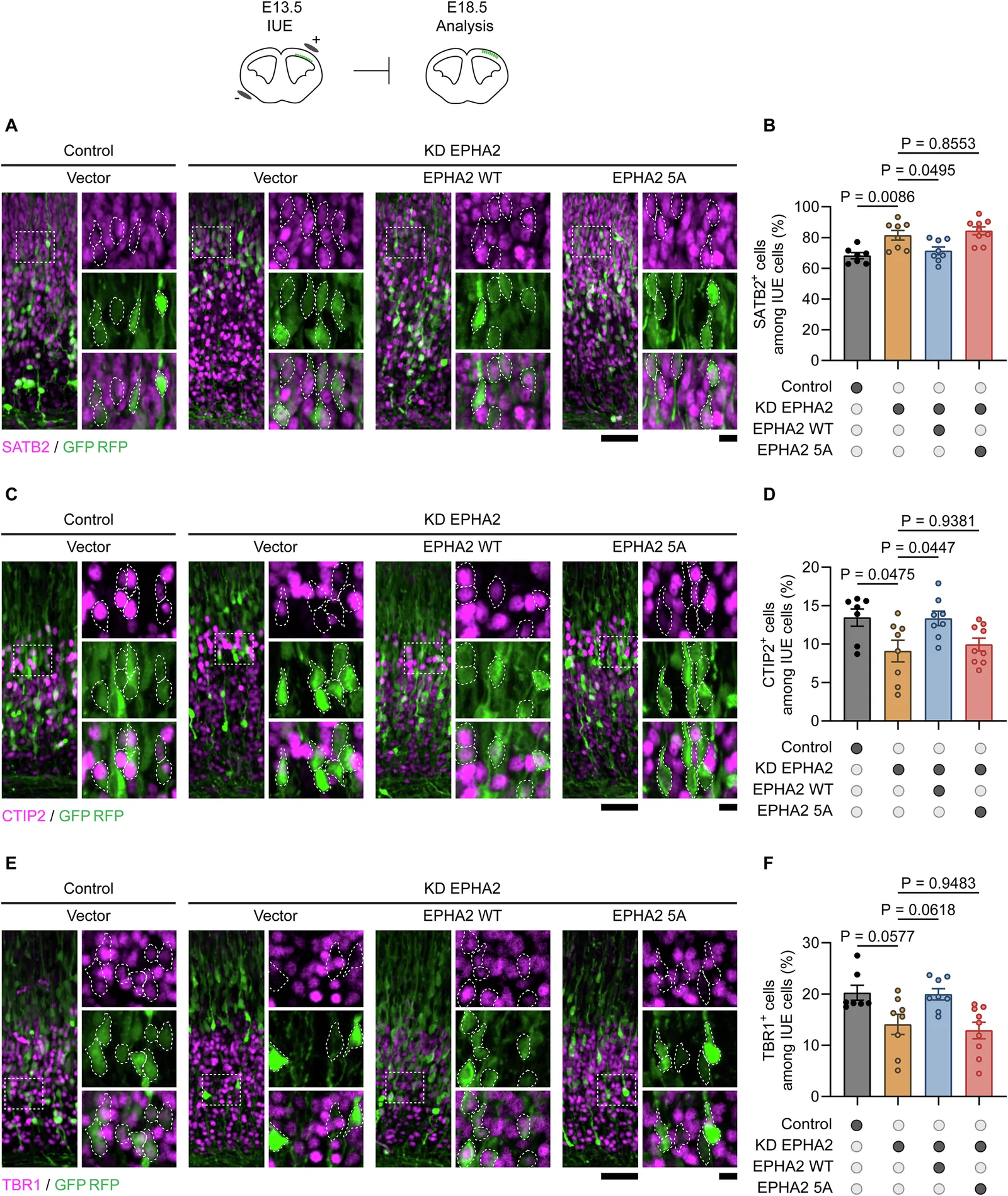
Ligand-independent EPHA2 disruption alters cortical projection neuron composition. IUE at E13.5 with EGFP-empty vector (control), EGFP-KD EPHA2 together with either RFP-empty vector, EPHA2 WT, or EPHA2 5A. Analysis at E18.5. (**A**) GFP RFP (green) and SATB2 (magenta) triple IF. Boxed areas denote zoomed areas. Dashed lines encircle electroporated cells that are SATB2-positive or SATB2-negative. Scale bars, 50 μm (left), 10 μm (right). (**B**) Quantification of SATB2-positive electroporated cells (Control *n* = 7, KD EPHA2 *n* = 8, KD EPHA2 + EPHA2 WT *n* = 8, KD EPHA2 + EPHA2 5A *n* = 9 embryos). (**C**) GFP RFP (green) and CTIP2 (magenta) triple IF. Boxed areas denote zoomed areas. Dashed lines encircle electroporated cells that are CTIP2-positive or CTIP2-negative. Scale bars, 50 μm (left), 10 μm (right). (**D**) Quantification of CTIP2-positive electroporated cells (Control *n* = 7, KD EPHA2 *n* = 8, KD EPHA2 + EPHA2 WT *n* = 8, KD EPHA2 + EPHA2 5A *n* = 9 embryos). (**E**) GFP RFP (green) and TBR1 (magenta) triple IF. Boxed areas denote zoomed areas. Dashed lines encircle electroporated cells that are TBR1-positive or TBR1-negative. Scale bars, 50 μm (left), 10 μm (right). (**F**) Quantification of TBR1-positive electroporated cells (Control *n* = 7, KD EPHA2 *n* = 8, KD EPHA2 + EPHA2 WT *n* = 8, KD EPHA2 + EPHA2 5A *n* = 9 embryos). Statistical analysis was performed using one-way ANOVA followed by Tukey’s post hoc test (**B**, **D**, **F**). Quantitative data were presented as mean ± SD. Exact *P* values are indicated above the bars in each quantification plot.

We then asked whether this early progenitor defect altered neuronal output at the end of embryonic neurogenesis. At E18.5, disruption of ligand-independent EPHA2 signaling increased the proportion of SATB2^+^ upper-layer neurons, accompanied by a decrease in CTIP2^+^ and TBR1^+^ early-born deep-layer neurons (Fig. EV2A–F). These results suggest that loss of ligand-independent EPHA2 signaling biases neuronal fate toward later-born neurons at the expense of early-born populations.

Collectively, our findings demonstrate that ligand-independent EPHA2 signaling promotes NSPC proliferation by sustaining cell cycle progression in the VZ/SVZ and also regulates timely cell cycle exit in the IZ, thereby coordinating progenitor maintenance and neurogenic progression during neocortical development.

### Proteomic analyses implicate mitochondrial pathways downstream of EPHA2 signaling

**Figure EV3:**
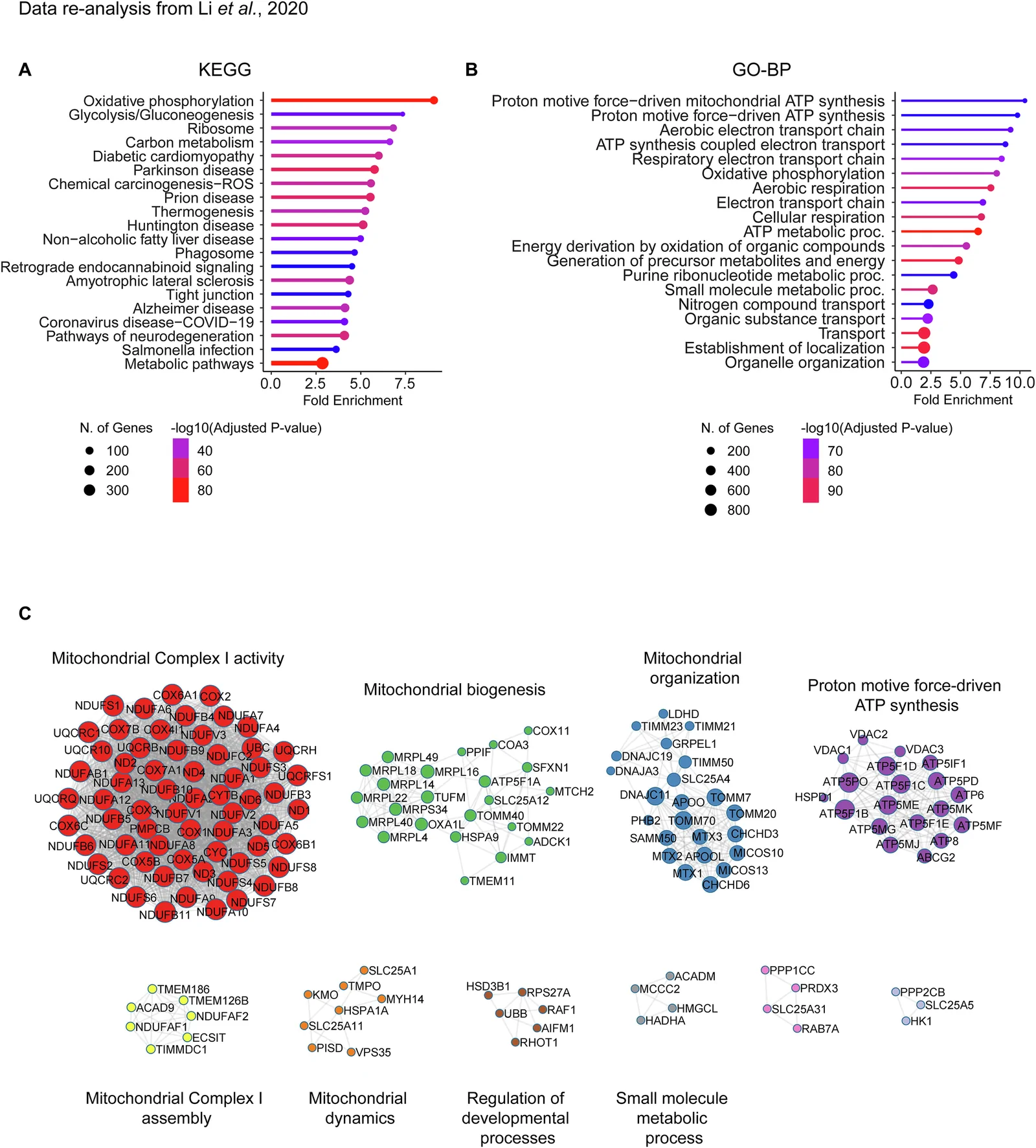
Re-analysis of the EPHA2-associated proteome highlights mitochondrial pathways. (**A**–**C**) EPHA2-associated proteome from non-cancerous mouse kidney tissue. Dataset from Li et al, 2020, was refined to include only proteins conserved in *Homo sapiens*, yielding 1757 interaction partners. (**A**) Kyoto Encyclopedia of Genes and Genomes (KEGG) pathway enrichment analysis of the EPHA2-associated proteome derived from non-cancerous mouse tissue. (**B**) Gene Ontology Biological Process (GO-BP) enrichment analysis of the same dataset. (**C**) Metascape protein interaction network of 321 mitochondrial proteins associated with EPHA2, highlighting modules linked to respiratory function and mitochondrial organization.

To identify physiological candidate effectors downstream of EPHA2, we re-analyzed a non-cancerous EPHA2-associated proteome reported by Li et al, (2020) (Li et al, 2020). Mitochondrial pathways, including oxidative phosphorylation (OXPHOS), respiratory electron transport, and ATP synthesis, were among the most strongly enriched categories (Fig. EV3A–C). These analyses nominated mitochondrial regulation as a potential downstream output of EPHA2 signaling.

**Figure 4:**
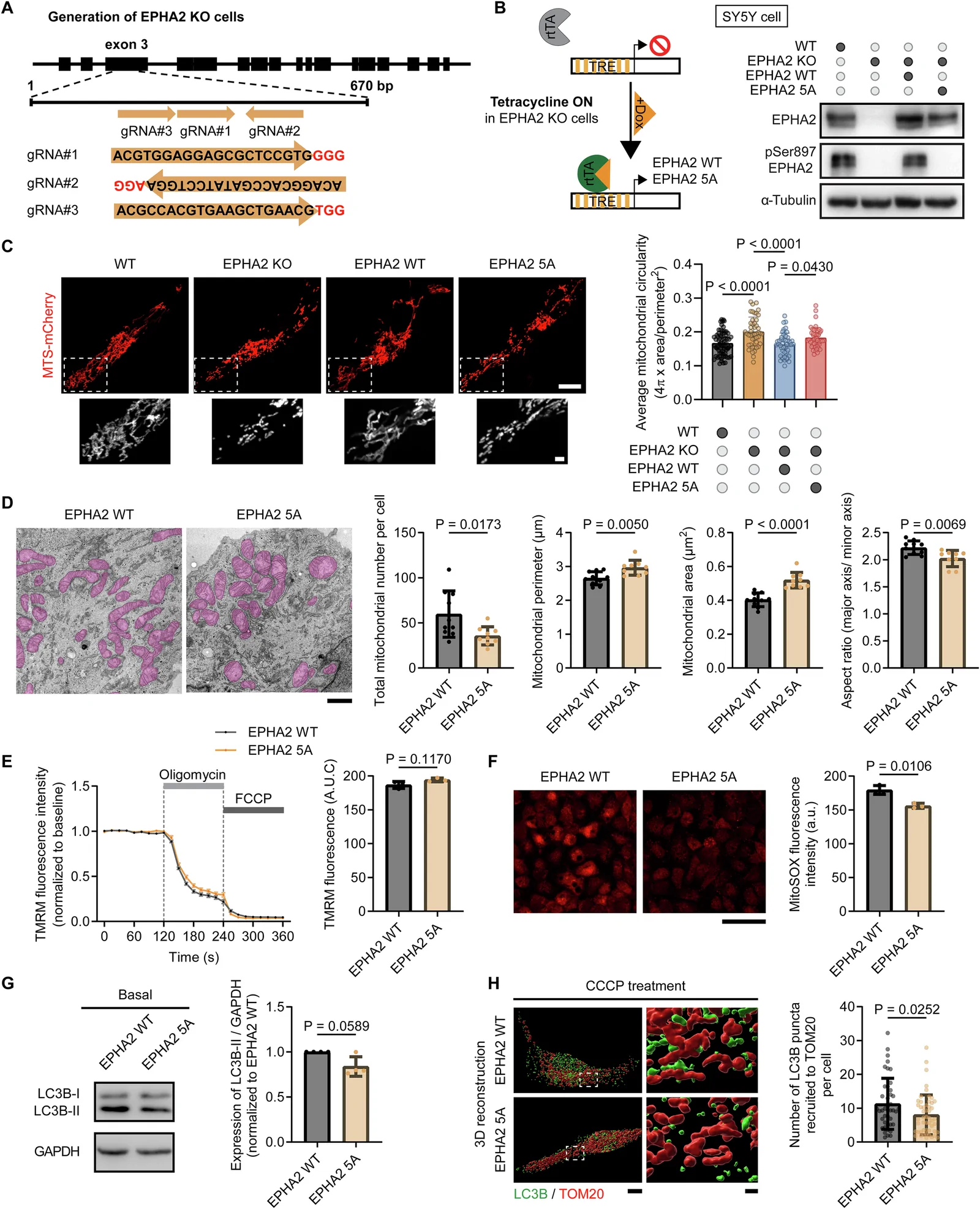
Ligand-independent EPHA2 signaling controls mitochondrial morphology and function. (**A**) Schematic of CRISPR-Cas9-mediated generation of EPHA2-knockout (KO) SH-SY5Y cells. (**B**) Immunoblot validation of EPHA2 KO and doxycycline-inducible rescue with EPHA2 WT or EPHA2 5A. Doxycycline was used at 0.5 µg/mL. (**C**) Mitochondrial morphology analysis in SH-SY5Y cells expressing the mitochondrial marker MTS-mCherry (WT *n* = 69 cells, EPHA2 KO *n* = 46 cells, EPHA2 WT *n* = 47 cells, EPHA2 5A *n* = 46 cells). Boxed areas denote zoomed areas. Scale bars, 10 µm (top), 2 µm (bottom). (**D**) Quantitative electron microscopy analysis of mitochondrial morphology in inducible EPHA2 WT and EPHA2 5A cells (EPHA2 WT *n* = 10 cells, EPHA2 5A *n* = 9 cells). Scale bar, 2 µm. (**E**) TMRM-based assessment of mitochondrial membrane potential before and after 2.5 μg/mL oligomycin and 5 μM FCCP treatment in inducible EPHA2 WT and EPHA2 5A cells (EPHA2 WT *n* = 3 replicates, EPHA2 5A *n* = 3 replicates). (**F**) MitoSOX-based assessment of mitochondrial superoxide levels in inducible EPHA2 WT and EPHA2 5A cells (EPHA2 WT *n* = 3 replicates, EPHA2 5A *n* = 3 replicates). Scale bar, 50 µm. (**G**) Basal LC3B-II immunoblot analysis in inducible EPHA2 WT and EPHA2 5A cells (EPHA2 WT *n* = 4 replicates, EPHA2 5A *n* = 4 replicates). (**H**) CCCP-induced mitophagy assay showing LC3B puncta co-localization with TOM20 (EPHA2 WT *n* = 42 cells, EPHA2 5A *n* = 51 cells). Boxed areas denote zoomed areas. Cells were reconstructed using Imaris software. Scale bars, 10 µm (left), 1 µm (right). Statistical analysis was performed using one-way ANOVA followed by Tukey’s post hoc test (**C**) and unpaired t-test with Welch’s correction (**D**–**H**). Quantitative data were presented as mean ± SD. Exact *P* values are indicated above the bars in each quantification plot. Source data are available online for this figure.

To test this possibility, we generated EPHA2-knockout (KO) SH-SY5Y cells and re-expressed EPHA2 WT or EPHA2 5A under doxycycline control (Fig. 4A,B). Expression of EPHA2 5A was associated with swollen and fragmented mitochondria, as assessed by electron microscopy and fluorescence-based morphometry (Fig. 4C,D). EPHA2 5A-expressing cells also exhibited modest changes in membrane potential and mitochondrial superoxide production, together with reduced LC3B recruitment to mitochondria following CCCP-induced mitophagy (Fig. 4E–H). These assays support perturbed mitochondrial homeostasis, but do not by themselves define the primary lesion.

**Figure 5:**
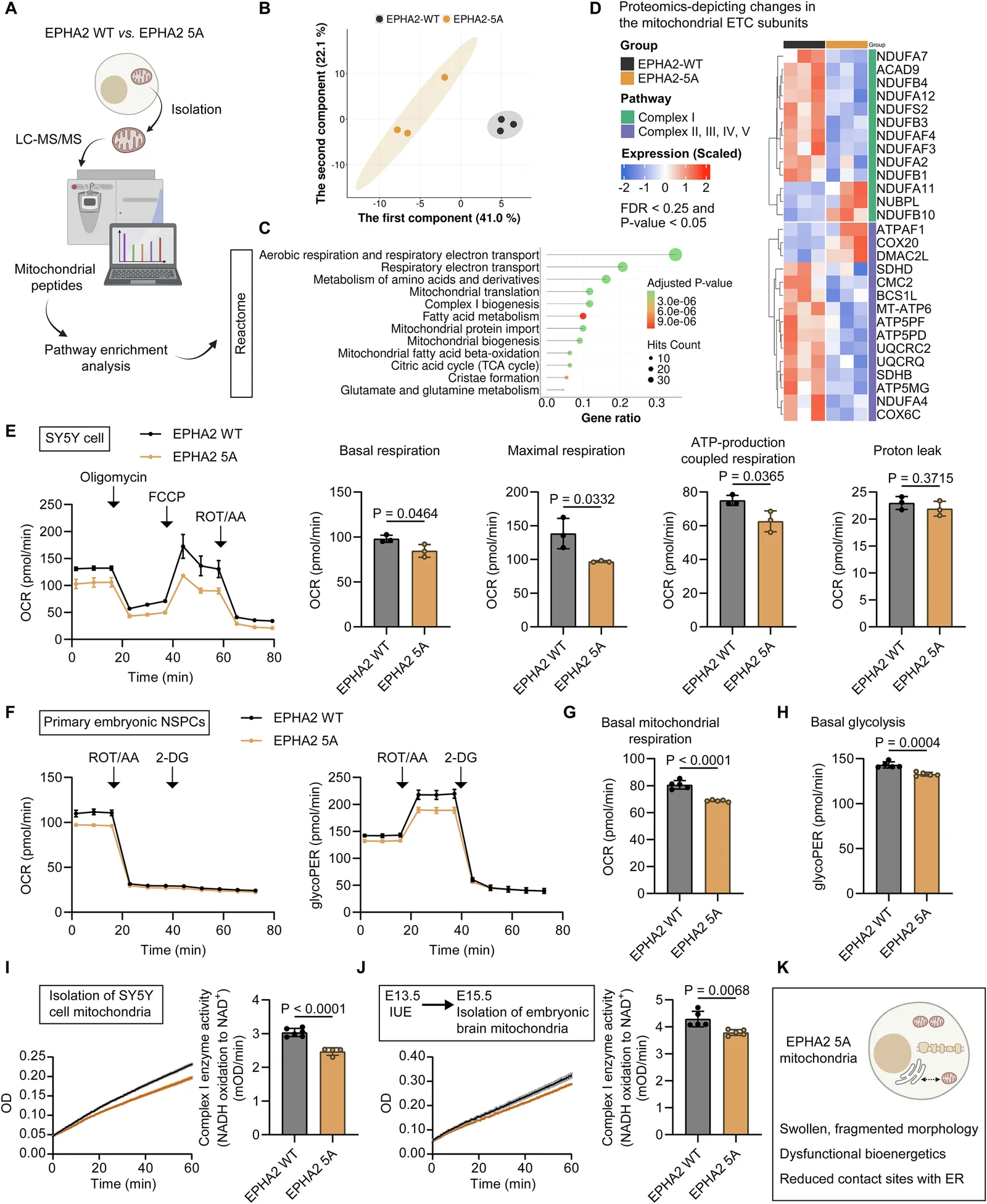
Ligand-independent EPHA2 sustains Complex I-linked mitochondrial respiration. (**A**) Experimental workflow for label-free quantitative proteomics of mitochondria isolated from inducible EPHA2 WT and EPHA2 5A SH-SY5Y cells. (**B**) Principal component analysis of mitochondrial proteomes, showing separation of EPHA2 WT and EPHA2 5A samples. (**C**) Reactome pathway enrichment analysis of differentially abundant mitochondrial proteins. (**D**) Heatmap of altered electron transport chain proteins, grouped by respiratory complex. (**E**) Seahorse analysis of oxygen consumption (Mito Stress Test) in inducible EPHA2 WT and EPHA2 5A SH-SY5Y cells, with quantification of basal, maximal, ATP-linked respiration, and proton leak (EPHA2 WT *n* = 3 replicates, EPHA2 5A *n* = 3 replicates). (**F**) Seahorse analysis of oxygen consumption and proton efflux rate (glycolytic rate assay) in primary NSPCs expressing EPHA2 WT or EPHA2 5A. (**G**) Quantification of basal mitochondrial respiration in primary NSPCs (EPHA2 WT *n* = 5 replicates, EPHA2 5A *n* = 5 replicates). (**H**) Quantification of basal glycolytic activity in primary NSPCs (EPHA2 WT *n* = 5 replicates, EPHA2 5A *n* = 5 replicates). (**I**) Complex I enzyme activity in mitochondria isolated from inducible EPHA2 WT and EPHA2 5A SH-SY5Y cells (EPHA2 WT *n* = 6 replicates, EPHA2 5A *n* = 4 replicates). mOD/min millioptical density per minute. (**J**) Complex I enzyme activity in mitochondria isolated from embryonic brains expressing EPHA2 WT or EPHA2 5A (EPHA2 WT *n* = 5 replicates, EPHA2 5A *n* = 5 replicates). (**K**) Schematic of mitochondrial defects in EPHA2 5A cells. Illustrations created with BioRender.com. Statistical analysis was performed using over-representation analysis with Fisher’s exact test (**C**), limma-based moderated *t*-test (**D**), and unpaired *t*-test with Welch’s correction (**E**, **G**, **H**–**J**). Quantitative data were presented as mean ± SD. Exact *P* values are indicated above the bars in each quantification plot. Source data are available online for this figure.

**Figure EV4:**
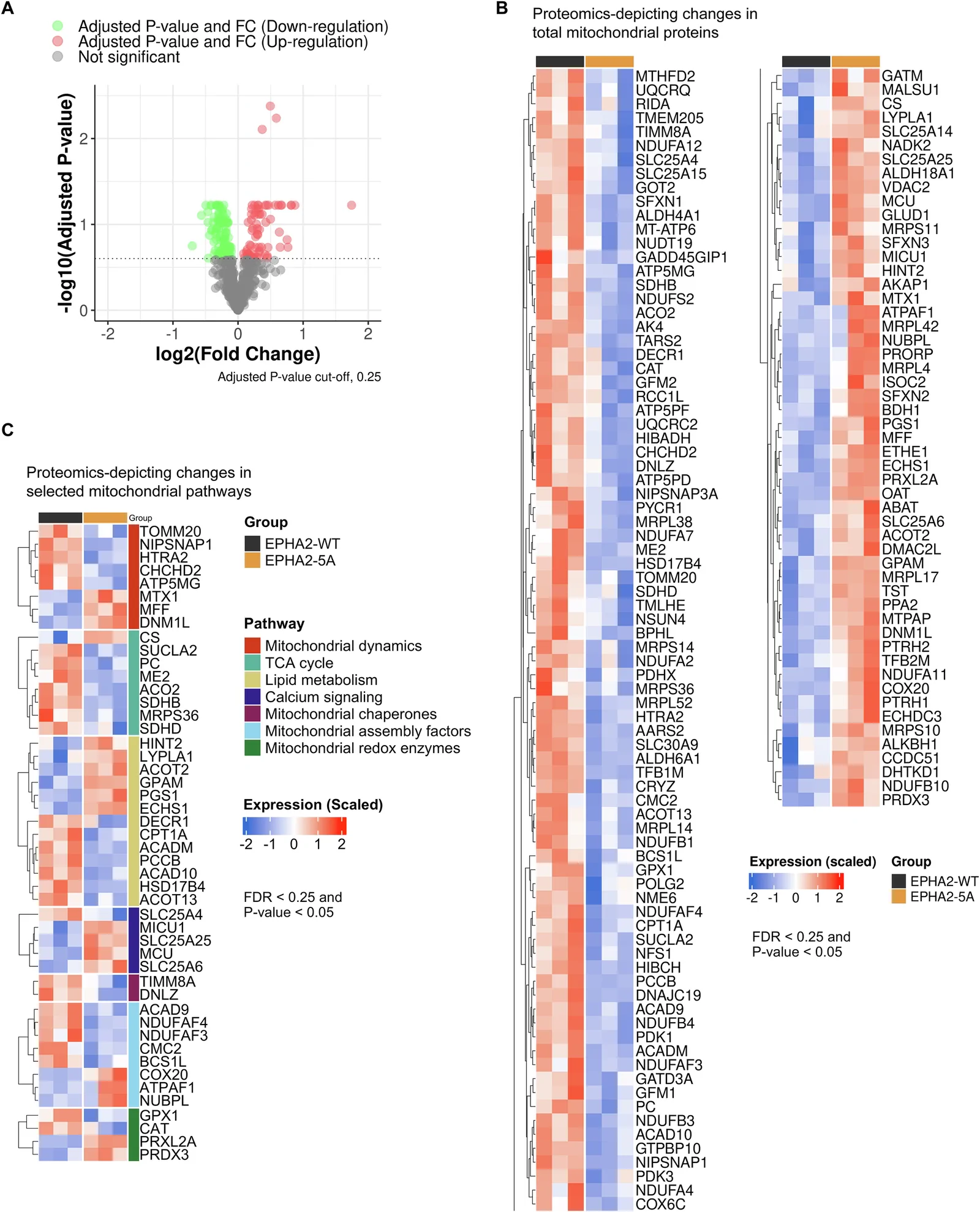
Expanded mitochondrial proteome analysis following disruption of ligand-independent EPHA2 signaling. (**A**) Volcano plot of differentially abundant mitochondrial proteins in inducible EPHA2 WT and EPHA2 5A cells (*n* = 3 replicates per condition). (**B**) Heatmap of significantly altered mitochondrial proteins. (**C**) Analysis of selected mitochondrial pathways revealed that mitochondrial dynamics, TCA cycle, and calcium signaling were also sensitive to disruption of ligand-independent EPHA2 signaling. Statistical analysis was performed using limma-based moderated *t*-test (**A**–**C**). Proteins shown met the significance thresholds used in the proteomic analysis. Exact thresholds are indicated in the figure.

We therefore performed label-free quantitative proteomic analysis of mitochondria isolated from EPHA2 WT and 5A cells (Fig. 5A). Principal component analysis demonstrated clear separation between the two groups, indicating distinct mitochondrial proteomic profiles (Figs. 5B and EV4A,B). Pathway enrichment identified aerobic respiration and the respiratory electron transport chain (ETC) among the most significantly altered mitochondrial programs (Fig. 5C). Respiratory-chain heatmaps revealed broad remodeling of ETC components, with prominent representation of Complex I-related proteins, although changes were not restricted to Complex I (Figs. 5D and EV4C).

### Ligand-independent EPHA2 sustains Complex I-linked mitochondrial respiration

**Figure EV5:**
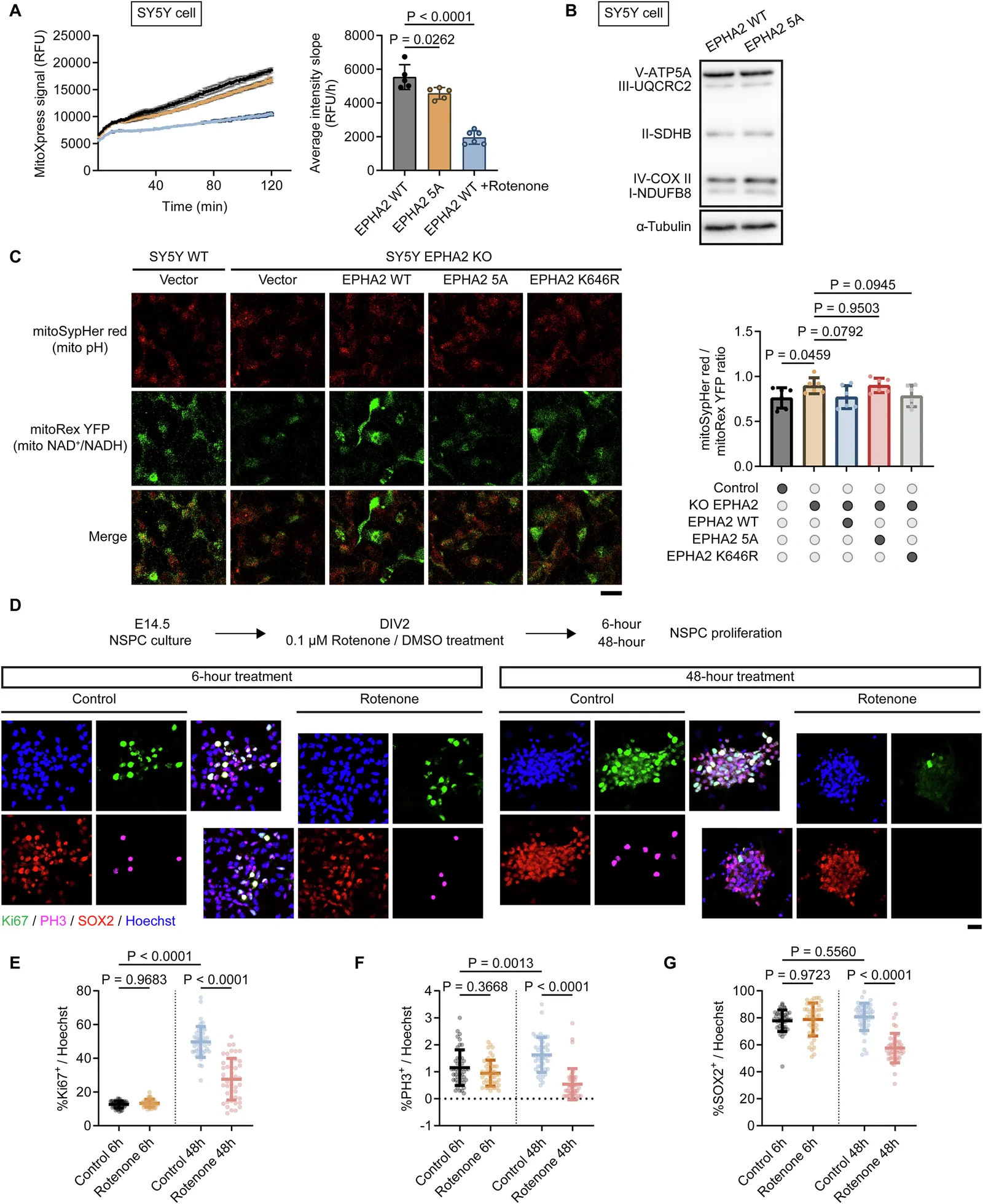
Pharmacological Complex I inhibition phenocopies the progenitor defect caused by EPHA2 disruption. (**A**) MitoXpress assay showing reduced oxygen consumption in inducible EPHA2 5A cells. Rotenone at 1 μM used as assay control (EPHA2 WT *n* = 5, EPHA2 5A *n* = 5, Rotenone *n* = 6 replicates). RFU/h, relative fluorescence units per hour. (**B**) Immunoblot analysis of total OXPHOS complex subunits in inducible EPHA2 WT and EPHA2 5A cells. (**C**) Ratiometric mitoRexYFP and mitoSypHer red imaging showing rescue of the mitochondrial redox phenotype by EPHA2 WT and EPHA2 K646R, but not by EPHA2 5A (*n* = 6 replicates per condition). Scale bar, 30 µm. (**D**) Representative images of primary NSPCs treated with vehicle or low-dose Rotenone and stained for proliferation markers. For each condition, 45 randomly selected microscopic fields were acquired. Scale bar, 20 µm. (**E**) Quantification of Ki67-positive NSPCs after Rotenone treatment (*n* = 45 per condition). (**F**) Quantification of PH3-positive NSPCs after Rotenone treatment (*n* = 45 per condition). (**G**) Quantification of SOX2-positive NSPCs after Rotenone treatment (*n* = 45 per condition). Statistical analysis was performed using one-way ANOVA followed by Tukey’s post hoc test (**A**, **C**, **E**–**G**). Quantitative data are presented as mean ± SD. Exact *P* values are indicated above the bars in each quantification plot.

We then assessed mitochondrial function directly. Seahorse analysis in SH-SY5Y cells showed that EPHA2 5A reduced basal respiration, maximal respiration, and ATP-linked respiration (Fig. 5E). In primary NSPCs, EPHA2 5A similarly reduced basal mitochondrial respiration and basal glycolytic activity (Fig. 5F–H). This decrease in respiration was recapitulated using MitoXpress, a complementary fluorescence-based oxygen consumption assay (Fig. EV5A). Immunoblot analysis of representative OXPHOS subunits did not reveal a major loss of the tested respiratory complex markers (Fig. EV5B), arguing that the defect is not explained by wholesale depletion of these proteins.

Having established reduced mitochondrial respiration, we next asked whether the defect involved impaired Complex I. Direct enzymatic assays in isolated mitochondria showed that EPHA2 5A reduced Complex I activity in both inducible SH-SY5Y cells and embryonic brain tissue (Fig. 5I,J). In line with this, EPHA2 KO SH-SY5Y cells exhibited a higher mitoSypHer red/mitoRexYFP ratio, consistent with altered Complex I NADH dehydrogenase activity. Re-expression of EPHA2 WT or the kinase-dead mutant K646R partially restored this ratio toward control levels, whereas EPHA2 5A failed to do so (Fig. EV5C). Pharmacological inhibition of Complex I using low-dose rotenone in primary NSPCs phenocopied the core progenitor defects caused by EPHA2 perturbation, reducing the fractions of Ki67^+^, PH3^+^, and SOX2^+^ cells (Fig. EV5D–G). Together, these data place a Complex I-linked mitochondrial respiratory defect downstream of ligand-independent EPHA2 signaling.

### Restoring mitochondrial NAD^+^/NADH redox balance attenuates altered laminar distribution induced by EPHA2 disruption

**Figure 6:**
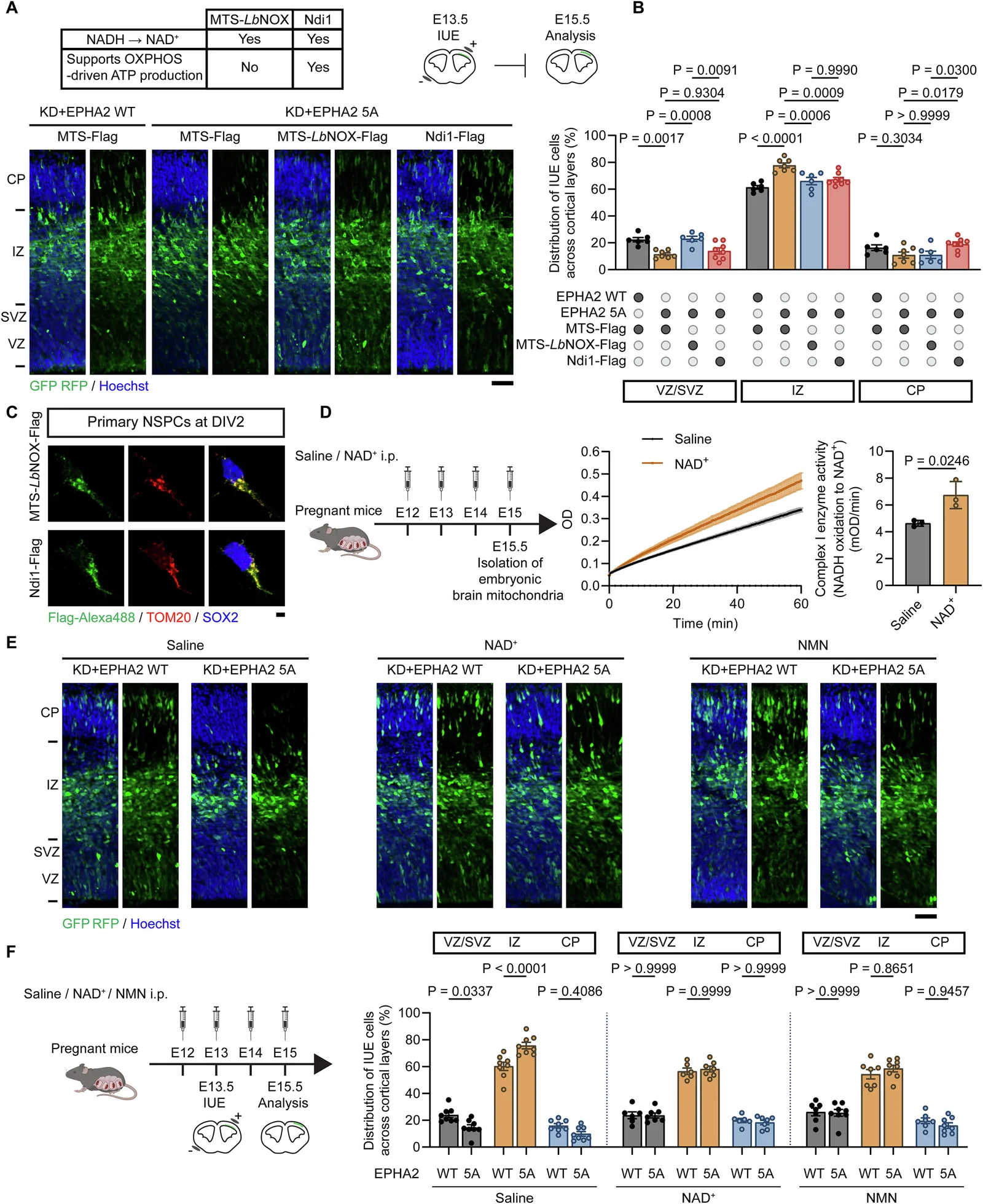
Restoring mitochondrial NAD^+^/NADH redox balance attenuates altered laminar distribution induced by EPHA2 disruption. (**A**,** B**) Functional comparison of MTS-*Lb*NOX and Ndi1 as tools to restore mitochondrial NAD^+^ regeneration. IUE at E13.5 with EGFP-KD EPHA2 + RFP-EPHA2 WT, EGFP-KD EPHA2 + RFP-EPHA2 5A together with either MTS-Flag, MTS-*Lb*NOX-Flag, or Ndi1-Flag. Analysis at E15.5. (**A**) GFP RFP electroporated cells are shown relative to cortical layers defined by Hoechst density. Scale bar, 50 μm. (**B**) Quantification of the distribution of electroporated cells across VZ/SVZ, IZ, and CP under the indicated conditions (EPHA2 WT + MTS-Flag *n* = 6, EPHA2 5A + MTS-Flag *n* = 7, EPHA2 5A + MTS-*Lb*NOX-Flag *n* = 6, EPHA2 5A + Ndi1-Flag *n* = 8 embryos). (**C**) Validation of MTS-*Lb*NOX-Flag and Ndi1-Flag constructs in primary NSPCs, demonstrating accurate mitochondrial localization. Scale bar, 3 μm. (**D**–**F**) Saline or NAD^+^ (10 mg in 100 μL saline) or NMN (500 mg/kg) was intraperitoneally injected daily into pregnant dams from E12 to E15, following IUE of either EGFP-KD EPHA2 + RFP-EPHA2 WT or EGFP-KD EPHA2 + RFP-EPHA2 5A at E13.5. Analysis at E15.5. (**D**) Complex I enzyme activity in mitochondria isolated from embryonic brains after maternal saline or NAD^+^ treatment. mOD/min, millioptical density per minute. (**E**) GFP RFP electroporated cells are shown relative to cortical layers defined by Hoechst density. Scale bar, 50 μm. (**F**) Quantification of the distribution of electroporated cells across VZ/SVZ, IZ, and CP under the indicated conditions (Saline + EPHA2 WT *n* = 8, Saline + EPHA2 5A *n* = 8, NAD^+^ + EPHA2 WT *n* = 6, NAD^+^ + EPHA2 5A *n* = 7, NMN + EPHA2 WT *n* = 7, NMN + EPHA2 5A *n* = 8 embryos). Statistical analysis was performed using two-way ANOVA with Šídák’s multiple comparisons test (**B**, **F**) and unpaired *t*-test with Welch’s correction (**D**). Quantitative data were presented as mean ± SD. Exact *P* values are indicated above the bars in each quantification plot. Source data are available online for this figure.

Mitochondrial Complex I carries out at least two coupled activities: electron transfer from NADH to ubiquinone and generation of a proton gradient that drives ATP synthesis via OXPHOS. To functionally disentangle these effects, we compared MTS-*Lb*NOX, which selectively enhances mitochondrial NAD^+^ regeneration without perturbing ETC flux, with Ndi1, which regenerates NAD^+^ while transferring electrons to the CoQ pool of the ETC and thereby contributes to ATP production (Fig. 6A–C). Expression of MTS-*Lb*NOX shifted the abnormal laminar distribution of EPHA2 5A-electroporated cells toward the EPHA2 WT pattern. In contrast, Ndi1 expression did not restore VZ/SVZ retention and instead biased cells toward the cortical plate (CP) (Fig. 6B).

We next tested whether increasing NAD^+^ availability in vivo modifies this phenotype. Maternal NAD^+^ supplementation from E12 to E15 was associated with increased ex vivo Complex I activity in mitochondria isolated from E15.5 embryonic brains (Fig. 6D). Under saline treatment, EPHA2 5A-electroporated cells showed reduced VZ/SVZ retention and increased IZ accumulation relative to EPHA2 WT. Following maternal NAD^+^ or the NAD^+^ precursor NMN administration, these WT *vs*. 5A differences were no longer detected in the laminar distribution analysis (Fig. 6E,F). These results support the view that mitochondrial NAD^+^ regeneration and redox balance are important downstream outputs of ligand-independent EPHA2 signaling in cortical progenitors.

### ER–mitochondria contacts regulate NSPC behavior independently of mitochondrial redox balance downstream of EPHA2

**Figure EV6:**
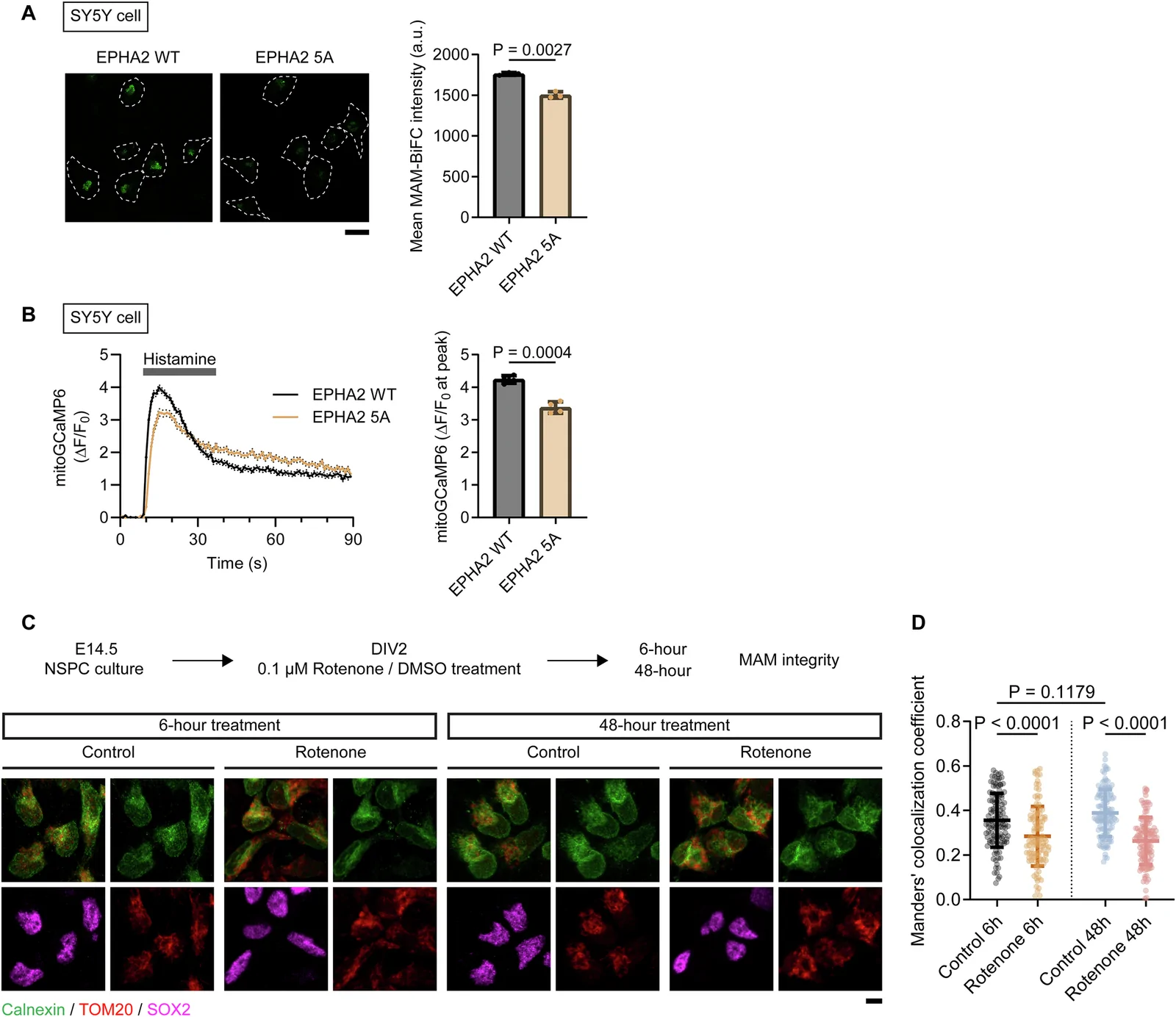
Ligand-independent EPHA2 signaling regulates ER–mitochondria contact sites. (**A**) MAM-BiFC-based analysis of ER–mitochondria contact sites (MAMs) in inducible EPHA2 WT and EPHA2 5A cells (EPHA2 WT *n* = 3 replicates, EPHA2 5A *n* = 3 replicates). Scale bar, 20 µm. (**B**) Mitochondrial calcium uptake assay measured by mitoGCaMP6 sensor following stimulation by 250 μM histamine in inducible EPHA2 WT and EPHA2 5A cells (EPHA2 WT *n* = 4 replicates, EPHA2 5A *n* = 4 replicates). (**C**) Representative images assessing ER–mitochondria contacts using ER marker Calnexin and mitochondrial marker TOM20 after Rotenone treatment in primary NSPCs. Scale bar, 5 µm. (**D**) Quantification of MAM integrity, assessed by co-localization between Calnexin and TOM20 after Rotenone treatment (Control 6 h *n* = 121, Rotenone 6 h *n* = 109, Control 48 h *n* = 123, Rotenone 48 h *n* = 120 cells). Statistical analysis was performed using unpaired *t*-test with Welch’s correction (**A**, **B**) and one-way ANOVA followed by Tukey’s post hoc test (**D**). Quantitative data are presented as mean ± SD. Exact *P* values are indicated above the bars in each quantification plot.

**Figure EV7:**
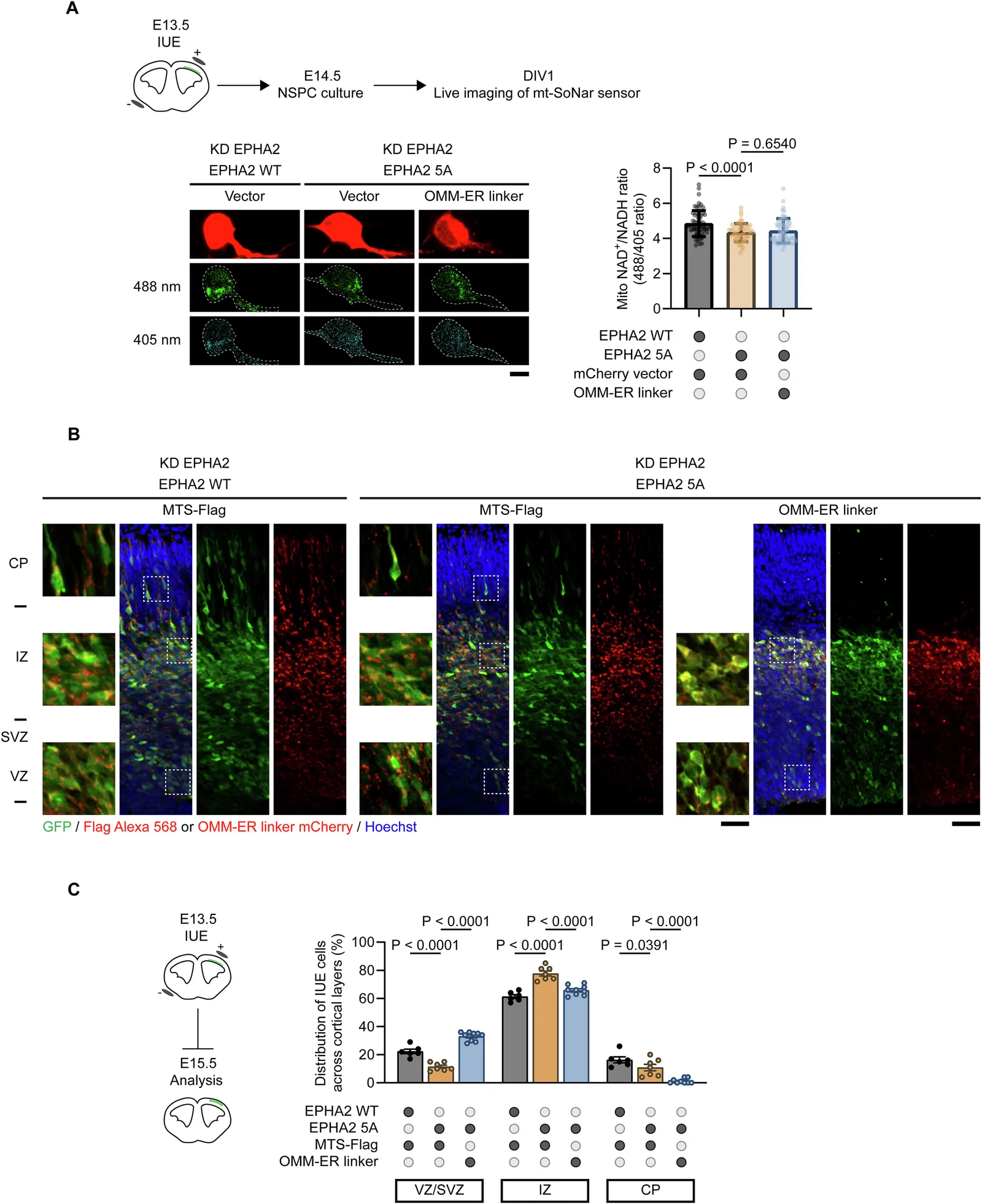
ER–mitochondria contacts regulate NSPC behavior independently of mitochondrial redox balance downstream of EPHA2. (**A**) Mitochondrial NAD^+^/NADH readout assessed by mt-SoNar sensor in primary NSPCs expressing EPHA2 WT or EPHA2 5A with or without an artificial OMM–ER linker (EPHA2 WT + Vector *n* = 61, EPHA2 5A +  Vector *n* = 60, EPHA2 5A + OMM–ER linker *n* = 63 cells). Scale bar, 5 µm. (**B**,** C**) IUE at E13.5 with EGFP-KD EPHA2 + EGFP-EPHA2 WT or EGFP-KD EPHA2 + EGFP-EPHA2 5A together with either MTS-Flag or OMM–ER linker. Analysis at E15.5. (**B**) GFP RFP electroporated cells are shown relative to cortical layers defined by Hoechst density. Boxed areas denote zoomed areas. Scale bars, 20 µm (left), 50 µm (right). (**C**) Quantification of the distribution of electroporated cells across VZ/SVZ, IZ, and CP under the indicated conditions (EPHA2 WT + MTS-Flag *n* = 6, EPHA2 5A + MTS-Flag *n* = 7, EPHA2 5A + OMM–ER linker *n* = 9 embryos). Statistical analysis was performed using one-way ANOVA followed by Tukey’s post hoc test (**A**) and two-way ANOVA with Šídák’s multiple comparisons test (**C**). Quantitative data are presented as mean ± SD. Exact *P* values are indicated above the bars in each quantification plot.

EPHA2 5A cells exhibited reduced ER–mitochondria proximity and mitochondrial Ca^2+^ uptake relative to EPHA2 WT (Fig. EV6A,B). We then asked whether loss of ER–mitochondria contacts can occur secondary to mitochondrial dysfunction, before the onset of the progenitor phenotype. In primary NSPCs, rotenone decreased ER–mitochondria co-localization as early as 6 h post-treatment, at a time point when NSPC proliferation remained unaffected (Fig. EV6C,D). By 48 h, both ER–mitochondria co-localization and NSPC proliferation were significantly reduced. We next tested whether restoring ER–mitochondria contacts rescues the EPHA2 5A phenotype. Expression of an artificial OMM–ER tether did not correct the mitochondrial NAD^+^/NADH defect, as measured by mt-SoNar sensor, in EPHA2 5A-expressing NSPCs (Fig. EV7A). In vivo, however, the tether altered the cortical distribution of electroporated cells. It significantly increased their retention within the VZ/SVZ, with minimal migration to the CP (Fig. EV7B,C). These data indicate that reduced ER–mitochondria contacts in the EPHA2 5A condition are unlikely to be the primary cause of the mitochondrial redox imbalance, but they may affect cortical cell distribution through a mechanistically independent manner.

### ECSIT links ligand-independent EPHA2 signaling to mitochondrial Complex I function

**Figure EV8:**
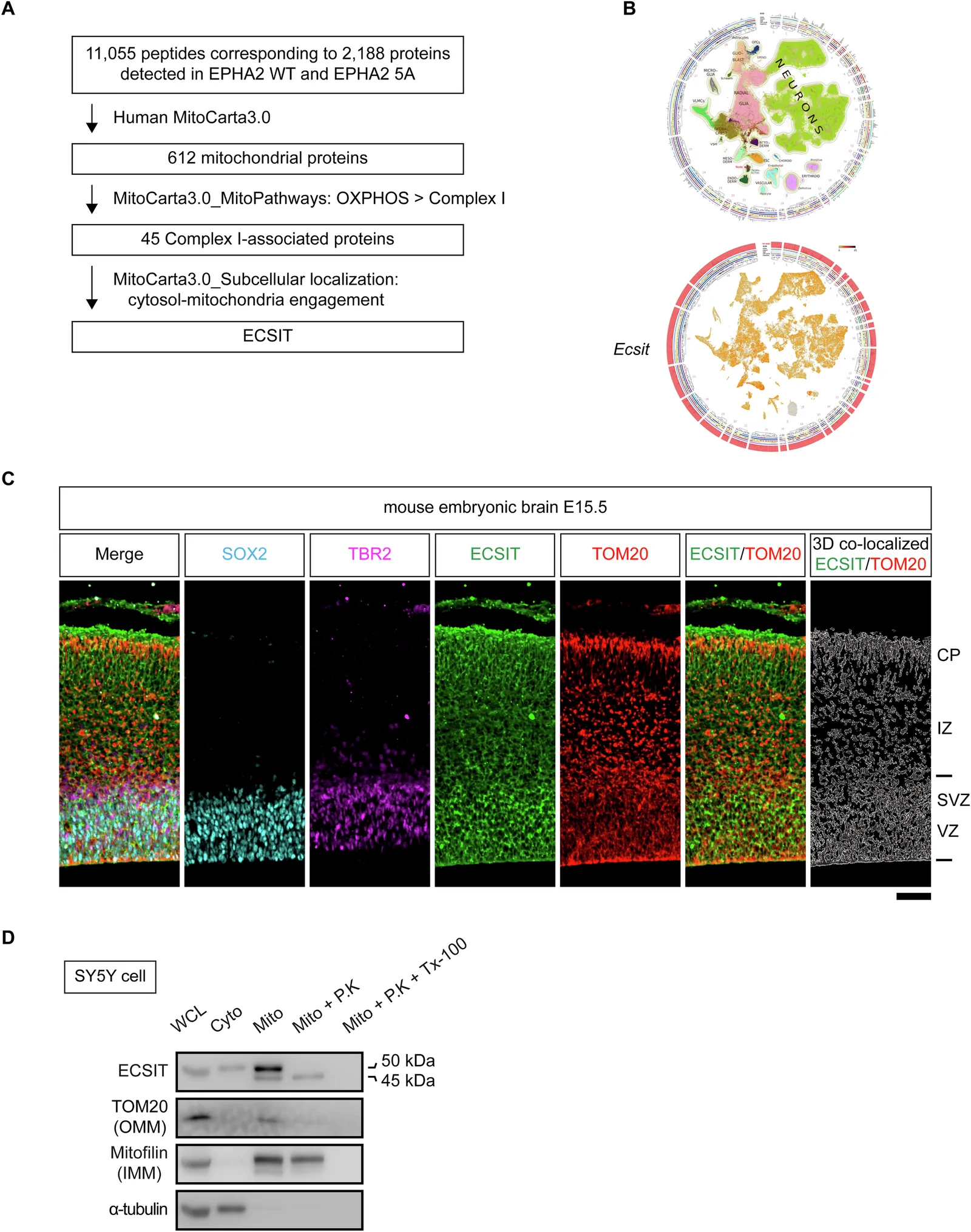
Layered evidence supporting ECSIT as an EPHA2-sensitive mitochondrial intermediary. (**A**) Schematic summary of the criteria used to prioritize ECSIT as a mechanistic intermediary downstream of EPHA2. (**B**) Single-cell transcriptomic map of the developing mouse brain showing *Ecsit* expression. Dataset from La Manno et al, 2021. (**C**) Immunofluorescence of E15.5 mouse cortex stained for ECSIT and TOM20. The 3D co-localized area between ECSIT and TOM20 was generated using Imaris software. Scale bar, 50 μm. (**D**) Proteinase K protection assay showing protease protection of the processed ECSIT species in intact mitochondria. PK proteinase K, Tx-100 Triton X-100.

**Figure 7:**
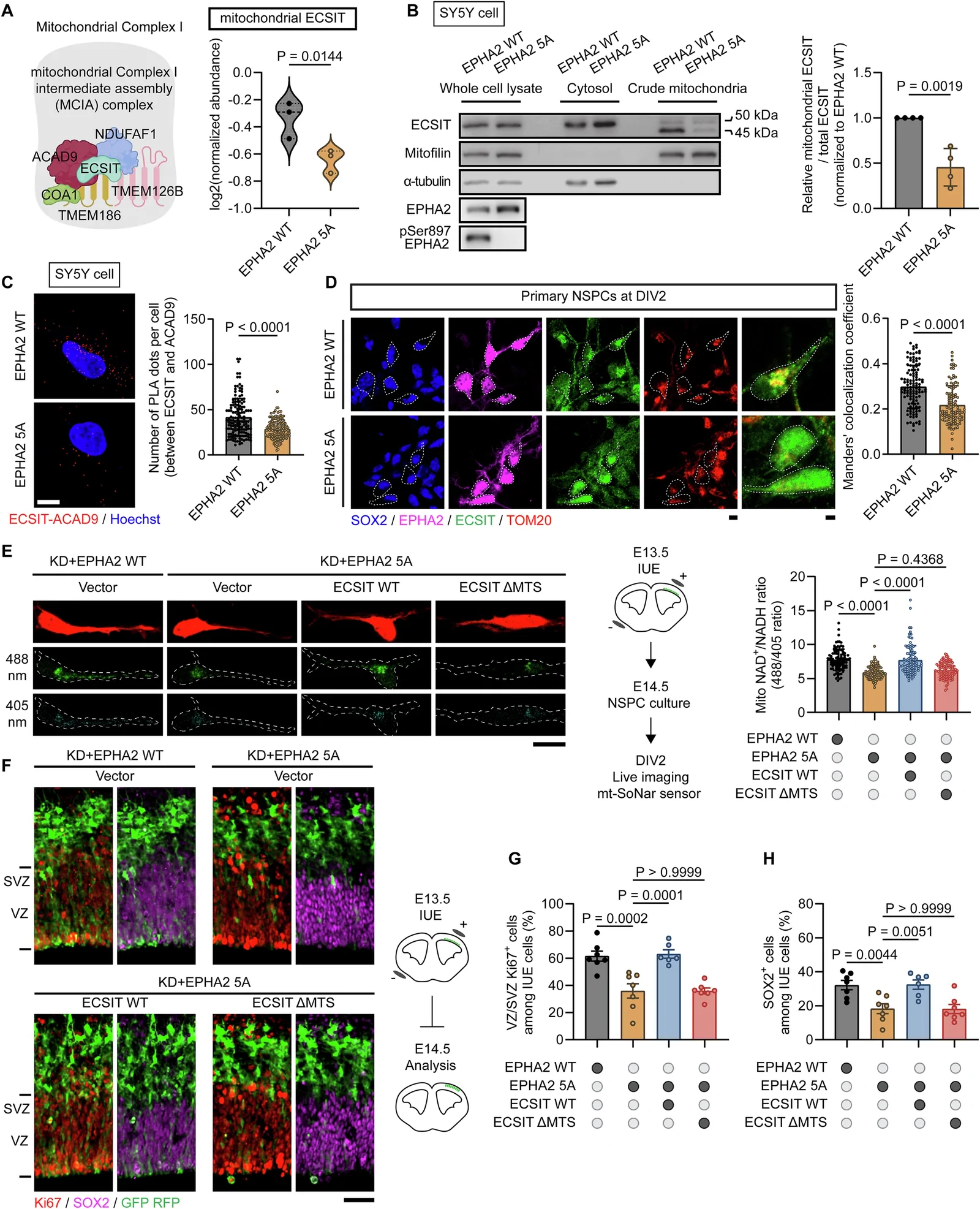
ECSIT links ligand-independent EPHA2 signaling to mitochondrial Complex I function. (**A**) Illustration of the components of the mitochondrial Complex I intermediate assembly (MCIA) complex, created with BioRender.com. Mitochondrial ECSIT abundance from proteomic analysis of inducible EPHA2 WT and EPHA2 5A cells. (**B**) Subcellular fractionation followed by immunoblotting for ECSIT, showing a cytosolic precursor (~50 kDa) and a processed mitochondrial ECSIT species (~45 kDa), with quantification of mitochondrial ECSIT relative to total ECSIT. (**C**) Proximity ligation assay (PLA) for endogenous ECSIT and ACAD9 in inducible EPHA2 WT and EPHA2 5A cells, with quantification of PLA red puncta per cell (EPHA2 WT *n* = 143 cells, EPHA2 5A *n* = 128 cells). Scale bar, 10 µm. (**D**) Immunofluorescence of primary NSPCs expressing EPHA2 WT or 5A stained for endogenous ECSIT and TOM20, with quantification of mitochondrial co-localization (EPHA2 WT *n* = 118 cells, EPHA2 5A *n* = 106 cells). Scale bars, 5 µm (left), 2 µm (right). (**E**) IUE at E13.5 with the mt-SoNar sensor and RFP-KD EPHA2 + RFP-EPHA2 WT, RFP-EPHA2 5A together with either RFP-empty vector, ECSIT WT, or ECSIT ΔMTS, followed by primary NSPC culture at E14.5 and live imaging at day in vitro 2 (DIV2). mt-SoNar imaging in primary NSPCs showing rescue of the mitochondrial NAD^+^/NADH readout by ECSIT WT, but not ECSIT ΔMTS (EPHA2 WT + Vector *n* = 100 cells, EPHA2 5A + Vector *n* = 102 cells, EPHA2 5A + ECSIT WT *n* = 100 cells, EPHA2 5A + ECSIT ΔMTS *n* = 96 cells). Scale bar, 15 µm. (**F**–**H**) IUE at E13.5 with RFP-KD EPHA2 + RFP-EPHA2 WT, RFP-EPHA2 5A together with either EGFP-empty vector, ECSIT WT, or ECSIT ΔMTS. Analysis at E14.5. (**F**) GFP RFP (green), Ki67 (red), and SOX2 (magenta) quadruple IF. Scale bar, 50 μm. (**G**) Quantification of Ki67-positive electroporated cells (EPHA2 WT + Vector *n* = 7, EPHA2 5A + Vector *n* = 7, EPHA2 5A + ECSIT WT *n* = 6, EPHA2 5A + ECSIT ΔMTS *n* = 7 embryos). (**H**) Quantification of SOX2-positive electroporated cells (EPHA2 WT + Vector *n* = 7, EPHA2 5A + Vector *n* = 7, EPHA2 5A + ECSIT WT *n* = 6, EPHA2 5A + ECSIT ΔMTS* n* = 7 embryos). Statistical analysis was performed using unpaired *t*-test with Welch’s correction (**A**–**D**) and one-way ANOVA followed by Tukey’s post hoc test (**E**, **G**,** H**). Quantitative data were presented as mean ± SD. Exact *P* values are indicated above the bars in each quantification plot. Source data are available online for this figure.

To identify a factor that could link plasma membrane EPHA2 signaling to mitochondrial Complex I activity, we prioritized mitochondrial proteins with established roles in Complex I biology, dual cytosolic and mitochondrial localization, and relevance to cortical progenitors. ECSIT emerged as a plausible candidate under this framework (Fig. EV8A) (Vogel et al, 2007; Xiao et al, 2003). *Ecsit* was broadly expressed across all cell types in the developing mouse brain, including neural progenitors (Fig. EV8B). Interestingly, ECSIT co-localized with TOM20 most prominently in the VZ/SVZ and CP, with weaker overlap in the IZ, where EPHA2 5A-expressing cells preferentially accumulate (Fig. EV8C). Mitochondrial proteomics revealed lower ECSIT abundance in EPHA2 5A mitochondria (Fig. 7A). Biochemical fractionation resolved ECSIT as a ~50 kDa cytosolic precursor and a ~45 kDa mitochondrial species, and EPHA2 5A selectively reduced the mitochondrial species without a clear change in whole cell ECSIT abundance (Fig. 7B). Proteinase K protection assay supported the interpretation that the lower band (~45 kDa) represents an imported mitochondrial form rather than a nonspecific degradation product (Fig. EV8D). Consistent with this, ECSIT proximity to ACAD9, a Complex I-associated mitochondrial factor, was reduced in EPHA2 5A cells, and primary NSPCs expressing EPHA2 5A similarly exhibited decreased ECSIT co-localization with TOM20 (Fig. 7C,D). Collectively, these data indicate that ligand-independent EPHA2 signaling regulates the mitochondrial pool of ECSIT rather than its total abundance.

We next asked whether this EPHA2-sensitive mitochondrial ECSIT pool is functionally relevant. In primary NSPCs, EPHA2 5A reduced the mitochondrial NAD^+^/NADH readout by mt-SoNar sensor, and this defect was restored by ECSIT WT but not by a mitochondrial targeting-deficient mutant ECSIT ΔMTS (Fig. 7E). In vivo, ECSIT WT, but not ECSIT ΔMTS, rescued the proportion of Ki67^+^ and SOX2^+^ progenitors in the VZ/SVZ of EPHA2 5A brains (Fig. 7F–H). Together, these results identify mitochondrial ECSIT as a functionally important intermediary linking ligand-independent EPHA2 signaling to Complex I mitochondrial redox phenotype.

### EPHA2 controls mitochondrial ECSIT accumulation and progenitor maintenance through a PP2A-sensitive ECSIT T179 phospho-state

**Figure EV9:**
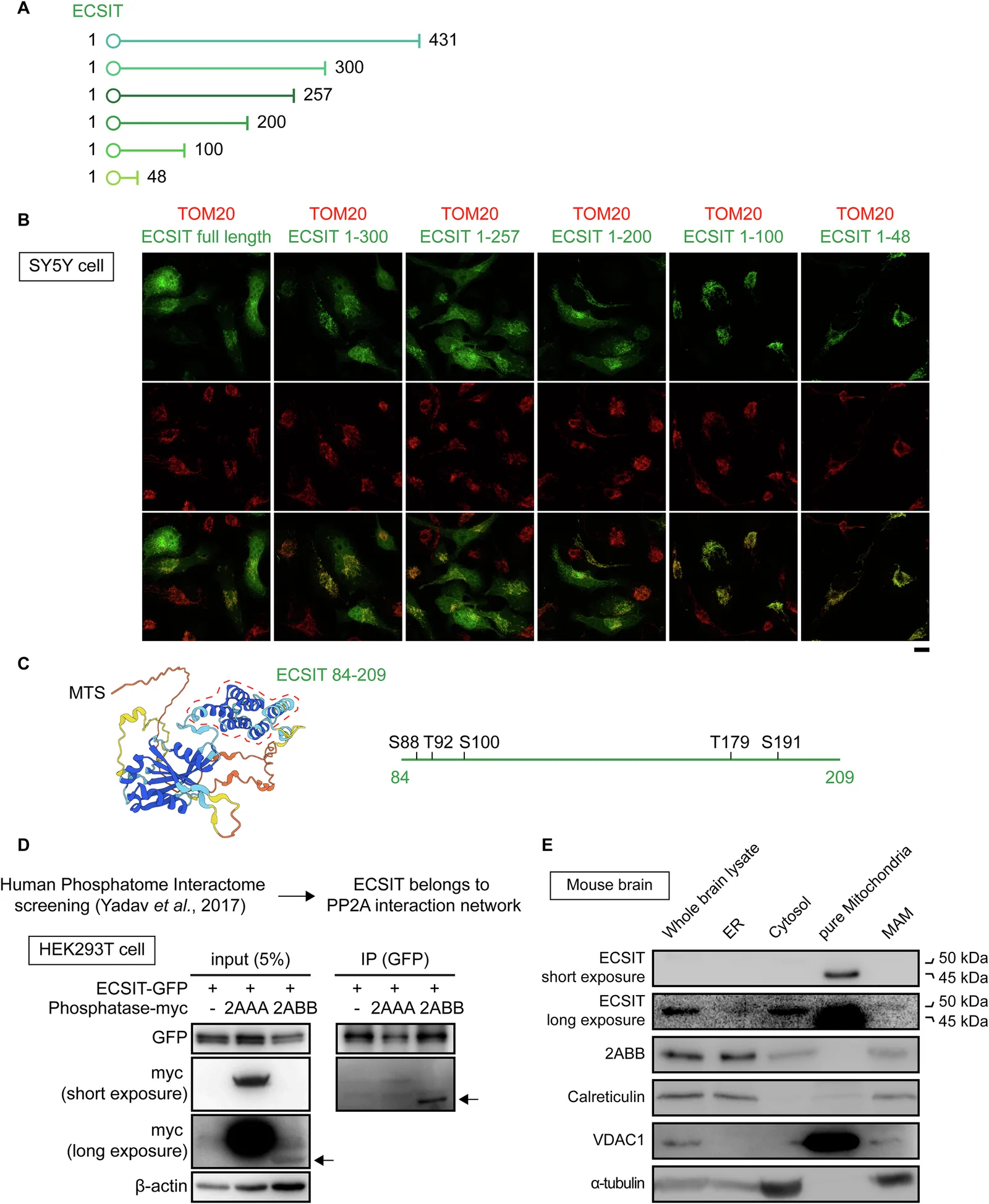
Mapping the ECSIT region and phosphosite that regulate mitochondrial accumulation. (**A**) Schematic of ECSIT truncation constructs used to define the region controlling mitochondrial localization. (**B**) Representative images of SH-SY5Y cells expressing full-length or truncated ECSIT-GFP constructs with TOM20 staining. Scale bar, 10 µm. (**C**) AlphaFold-based structural model of ECSIT highlighting a cluster of alpha helices spanning amino acids 84–209 that contains candidate serine and threonine residues. (**D**) Immunoprecipitation showing association between ECSIT and the PP2A regulatory subunit PPP2R2B/2ABB in HEK293T cells. (**E**) Subcellular fractionation of mouse brain lysates showing ECSIT and PPP2R2B/2ABB distribution across ER, cytosol, pure mitochondria, and MAM fractions.

**Figure 8:**
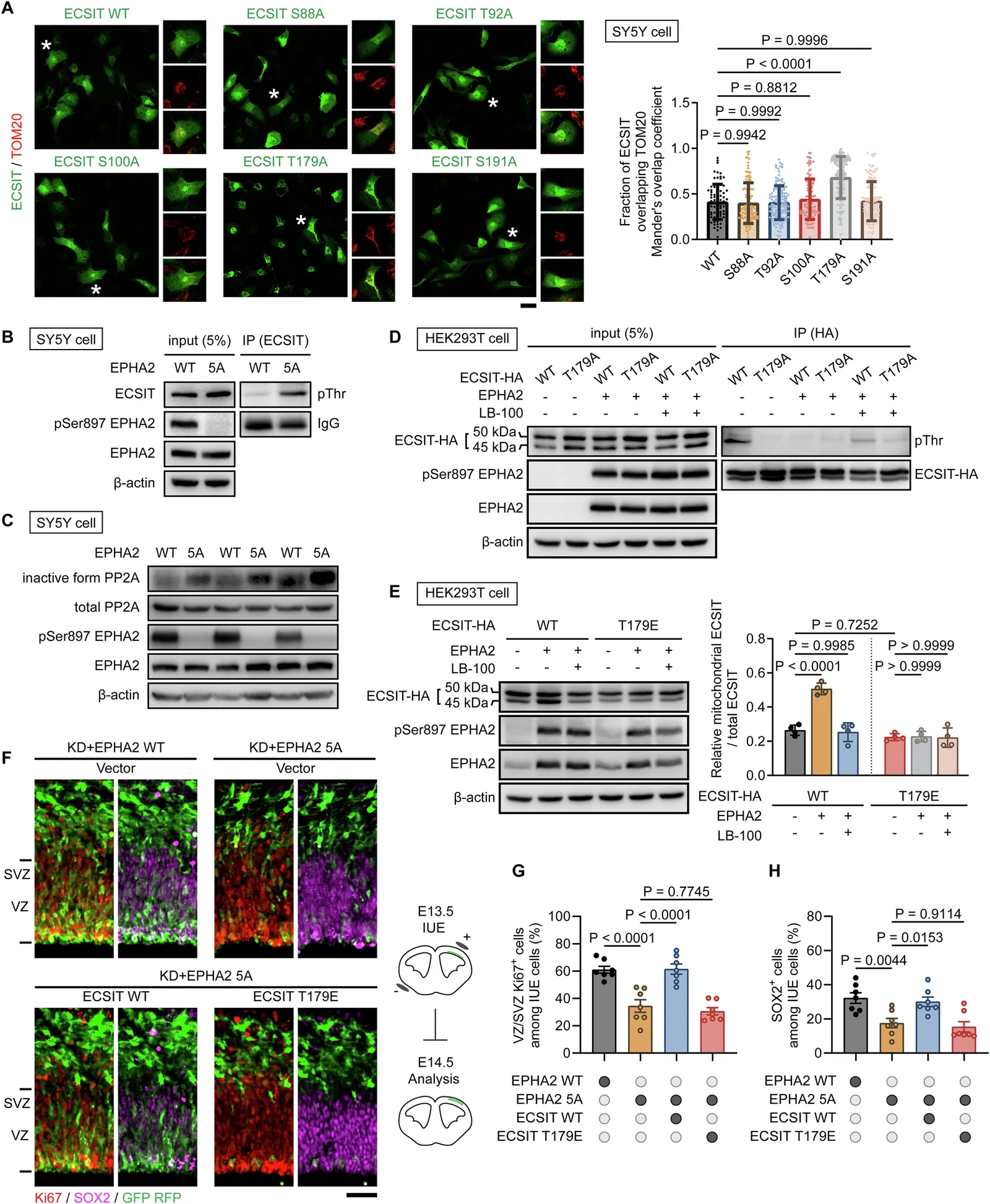
EPHA2 controls mitochondrial ECSIT accumulation and progenitor maintenance through a PP2A-sensitive ECSIT T179 phospho-state. (**A**) Representative images of ECSIT WT and phosphosite mutants expressed in SH-SY5Y cells, with TOM20 staining. The asterisk-marked cell is in focus. Quantification of mitochondrial co-localization (ECSIT WT *n* = 74 cells, S88A *n* = 121 cells, T92A *n* = 138 cells, S100A *n* = 113 cells, T179A *n* = 158 cells, S191A *n* = 126 cells). Scale bar, 20 µm. (**B**) Immunoprecipitation of endogenous ECSIT followed by phospho-threonine immunoblotting in inducible EPHA2 WT and EPHA2 5A cells. (**C**) Immunoblot analysis of PP2A Tyr307 phosphorylation, a marker of its inactive form, in inducible EPHA2 WT and EPHA2 5A cells. (**D**) Reconstitution assay showing EPHA2-dependent reduction of ECSIT phospho-threonine signal and partial reversal by the PP2A inhibitor LB-100 (2.5 μM for 6 h). (**E**) Mitochondrial localization assay comparing ECSIT WT and ECSIT T179E in the presence or absence of EPHA2 co-expression and PP2A inhibition, with quantification of mitochondrial ECSIT relative to total ECSIT (*n* = 4 replicates per condition). (**F**–**H**) IUE at E13.5 with RFP-KD EPHA2 + RFP-EPHA2 WT, RFP-EPHA2 5A together with either EGFP-empty vector, ECSIT WT, or ECSIT T179E. Analysis at E14.5. (**F**) GFP RFP (green), Ki67 (red), and SOX2 (magenta) quadruple IF. Scale bar, 50 μm. (**G**) Quantification of Ki67-positive electroporated cells (EPHA2 WT + Vector *n* = 7, EPHA2 5A + Vector *n* = 7, EPHA2 5A + ECSIT WT *n* = 7, EPHA2 5A + ECSIT T179E *n* = 7 embryos). (**H**) Quantification of SOX2-positive electroporated cells (EPHA2 WT + Vector *n* = 7, EPHA2 5A + Vector *n* = 7, EPHA2 5A + ECSIT WT *n* = 7, EPHA2 5A + ECSIT T179E *n* = 7 embryos). Statistical analysis was performed using one-way ANOVA followed by Dunnett’s post hoc test (**A**) and one-way ANOVA followed by Tukey’s post hoc test (**E**, **G**, **H**). Quantitative data were presented as mean ± SD. Exact *P* values are indicated above the bars in each quantification plot. Source data are available online for this figure.

To define how EPHA2 regulates the mitochondrial pool of ECSIT, we first mapped the ECSIT region responsible for its mitochondrial accumulation. ECSIT constructs containing only amino acids 1–100 completely co-localized with TOM20, whereas those including residues 100–200 still exhibited cytosolic distribution, indicating that the 100–200 region restricts mitochondrial localization of ECSIT (Fig. EV9A,B). AlphaFold-based structure prediction revealed a cluster of alpha helices spanning amino acids 84–209. Because recent work has linked ECSIT dephosphorylation to ACAD9 association inside mitochondria (McGregor et al, 2023), and because we hypothesize that ECSIT functions downstream of a serine/threonine (S/T) signaling cascade initiated by ligand-independent EPHA2, we tested five S/T-to-A mutants within this segment (Fig. EV9C). Among these, only T179A reproducibly increased mitochondrial ECSIT localization relative to WT and the other phosphosite mutants (Fig. 8A). Consistent with this result, endogenous ECSIT showed lower phospho-threonine signal in EPHA2 WT than in EPHA2 5A cells (Fig. 8B), linking ligand-independent EPHA2 signaling to ECSIT phospho-state.

Since ECSIT was identified exclusively as a PP2A-interacting protein—among many phosphatases surveyed—in screening datasets (Yadav et al, 2017), we next asked whether PP2A couples EPHA2 to ECSIT regulation. ECSIT was associated with the PP2A regulatory subunit PPP2R2B/2ABB in our system (Fig. EV9D). We therefore focused on examining ECSIT phosphorylation state and PP2A activity. EPHA2 5A cells retained higher ECSIT threonine phosphorylation than EPHA2 WT cells (Fig. 8B). In parallel, EPHA2 5A increased inactive-form Tyr307 levels of PP2A, consistent with reduced PP2A activity (Fig. 8C). Together, these results suggest that PP2A activity links ligand-independent EPHA2 signaling to the phosphorylation state of ECSIT.

In HEK293T cell reconstitution assay, EPHA2 co-expression reduced the phospho-threonine signal on ECSIT WT, whereas pharmacological PP2A inhibition with LB-100 partially restored this signal (Fig. 8D). EPHA2 also promoted mitochondrial localization of ECSIT WT, and this effect was blocked by LB-100. By contrast, the phosphomimetic mutant ECSIT T179E remained poorly localized to mitochondria irrespective of EPHA2 co-expression or PP2A inhibition (Fig. 8E). Functionally, ECSIT WT, but not ECSIT T179E, rescued the Ki67 and SOX2 defects induced by EPHA2 5A in vivo (Fig. 8F–H). These data support the view that ligand-independent EPHA2 signaling regulates a PP2A-sensitive ECSIT T179 phospho-state that influences mitochondrial ECSIT accumulation and downstream progenitor maintenance.

## Discussion

Our study supports a model in which an EPHA2 signaling mode linked to embryonic progenitor maintenance is coupled to a mitochondrial redox output. The work positions a receptor-state-associated EPHA2 pathway upstream of mitochondrial ECSIT abundance, Complex I-driven mitochondrial NAD^+^ regeneration, and progenitor maintenance, thereby conceptually advancing beyond the established requirement for mitochondrial function in neural progenitors (Cabello-Rivera et al, 2019; Khacho et al, 2017; Scandella et al, 2023). In this framework, EPHA2 perturbation reduces mitochondrial respiration and Complex I activity, alters mitochondrial redox balance, and promotes premature depletion of cortical progenitors. The present data collectively provide a rare functional link between receptor state at the cell surface and mitochondrial redox control during corticogenesis. At the same time, the mechanistic boundary of the study is clear. The PP2A experiments point to a phospho-sensitive step involving ECSIT, including T179, but do not establish direct dephosphorylation of ECSIT by PP2A. The designation as ligand-independent is likewise inferred from signaling-selective mutants and Ser897-associated readouts, not from direct exclusion of ephrin input in vivo. In addition, part of the mechanistic dissection was carried out in SH-SY5Y cells to enable biochemical analysis, whereas the developmental phenotype and key rescue logic were established in primary NSPCs and embryonic cortex.

Mitochondrial Complex I performs at least two coupled functions: it transfers electrons from NADH to ubiquinone while helping generate the proton gradient that drives ATP production via OXPHOS (Okoye et al, 2023). Our data suggest that, downstream of EPHA2, the Complex I output most relevant to progenitor maintenance is mitochondrial NAD^+^ regeneration rather than ATP production itself. This interpretation is supported by the rotenone phenocopy and by the differential rescue effects of mitochondrial *Lb*NOX and Ndi1, which together implicate matrix NAD^+^ regeneration as the critical downstream function in the context of EPHA2 signaling. These findings are consistent with the view that embryonic NSPCs rely primarily on glycolysis to meet their energy demands (Dong et al, 2022), whereas OXPHOS-dependent ATP production is less central at this stage. Accordingly, the role of Complex I downstream of EPHA2 is likely weighted more toward preserving mitochondrial NAD^+^/NADH redox balance than toward supporting ATP synthesis for progenitor maintenance.

NAD^+^ and NMN rescue data further support a redox-based link. By oxidizing mitochondrial NADH to regenerate NAD^+^, the ETC can also indirectly influence cytosolic NADH oxidation through shuttle systems, potentially affecting broader NAD^+^-dependent pathways (Titov et al, 2016). Thus, although our data are consistent with a model in which restoration of mitochondrial redox state and Complex I function contributes to the rescue of developmental defects caused by EPHA2 perturbation, we cannot exclude additional contributions from broader NAD^+^-dependent processes, including sirtuin signaling and PARP activity. Further work is needed to elucidate how mitochondrial NAD^+^/NADH balance or broader NAD^+^-dependent pathways influence NSPC behavior. From the present data, the most direct conclusion is that EPHA2 helps preserve a mitochondrial redox state required for progenitor maintenance, with ECSIT acting downstream.

ECSIT provides an important mechanistic bridge in this pathway. ECSIT is a known Complex I assembly factor within the MCIA machinery (McGregor et al, 2023; Vogel et al, 2007). Here, EPHA2 perturbation lowers mitochondrial ECSIT abundance, reduces ECSIT proximity to ACAD9, and makes both mitochondrial redox balance and progenitor maintenance dependent on ECSIT mitochondrial targeting. These findings argue that the functionally relevant pool is the mitochondrial ECSIT pool, rather than total cellular ECSIT. What remains unresolved is the precise step being controlled, whether EPHA2 signaling influences import competence, mitochondrial retention, processing, or assembly-factor engagement after entry of ECSIT.

The phospho-state data offer one plausible regulatory layer within this signaling axis. T179A enhances mitochondrial ECSIT accumulation, T179E fails to rescue the progenitor phenotype, and PP2A inhibition opposes the EPHA2-dependent reduction in ECSIT phospho-threonine signal. Taken together, these results are consistent with a PP2A-sensitive phospho-regulatory step acting on ECSIT downstream of EPHA2. This conclusion does not require a fully resolved EPHA2–PP2A–ECSIT chain, and the present data do not yet provide one. At this stage, it is sufficient to place reversible ECSIT phospho-regulation within the pathway linking EPHA2 to mitochondrial redox control. This interpretation is also compatible with prior work showing that phosphorylation can influence mitochondrial precursor handling and import-related steps (Law et al, 2015; Rao et al, 2012; Schmidt et al, 2011).

The ER–mitochondria contact phenotype suggests that EPHA2 may influence additional mitochondrial inputs beyond ECSIT, but the current results do not place this branch upstream of the primary redox defect. Likewise, the upstream cues that sustain ligand-independent EPHA2 signaling in cortical progenitors remain unresolved. Cerebrospinal fluid (CSF)-derived signals are plausible candidates, given that the apical surface of early NSPCs is directly exposed to a CSF milieu rich in nutrients, growth factors, and signaling molecules important for proliferation and differentiation (Chau et al, 2015; Lehtinen et al, 2011). Several growth factors present in CSF have also been reported to activate EPHA2 through ligand-independent mechanisms (Miao et al, 2009). More broadly, our findings raise the possibility that EPHA2 acts in a temporally distinct manner during corticogenesis, with an early ligand-independent state supporting progenitor expansion and later ligand-dependent activation contributing to neuronal differentiation as ephrin-A1 expression rises in surrounding lineages (Aoki et al, 2004). Although speculative, these ideas provide directions for future investigation. Taken together, our study suggests that EPHA2 supports embryonic cortical progenitor maintenance by preserving mitochondrial ECSIT abundance and Complex I-linked redox homeostasis, thereby linking receptor signaling state at the cell surface to mitochondrial redox control during neocortical development.

## Methods

**Table:** Reagents and tools table

Reagent/resource	Reference or source	Identifier or catalog number
**Experimental models**		
HEK293T (Human embryonic kidney cell line)	ATCC	Cat# CRL-3216
SH-SY5Y (Human neuroblastoma cell line)	Korean cell line bank	Cat# 22266
Neuro-2a (Mouse neuroblastoma cell line)	ATCC	Cat# CCL-131
WAe009-A (Human embryonic stem cell line)	WiCell	RRID: CVCL_9773
Pregnant C57BL/6J (*Mus musculus*)	Hyochang Science	
**Recombinant DNA**		
pCAG-human EPHA2 WT T2A RFP	This study	
pCAG-human EPHA2 5A T2A RFP	This study	
pCAG-human EPHA2 K646R T2A RFP	This study	
pCAG-human EPHA2 D739N T2A RFP	This study	
pCAG-RFP vector	This study	
pLL3.7-EGFP vector	This study	
pLL3.7-EGFP-shRNA mouse EPHA2	This study	
pLL3.7-RFP vector	This study	
pLL3.7-RFP shRNA mouse EPHA2	This study	
pCAG-EGFP vector	This study	
pCAG-human ECSIT WT T2A EGFP	This study	
pCAG-human ECSIT WT T2A RFP	This study	
pCAG-human ECSIT ΔMTS T2A EGFP	This study	
pCAG-human ECSIT ΔMTS T2A RFP	This study	
pCAG-human ECSIT T179E T2A EGFP	This study	
pCAG-MTS-Flag	This study	
pCAG-MTS-*Lb*NOX-Flag	This study	
pCAG-Ndi1-Flag	This study	
pCAG-OMM-ER linker-mCherry	This study	
pCW57.1-human EPHA2 WT	This study	
pCW57.1-human EPHA2 5A	This study	
pcDNA3.1-human EPHA2 WT -3xFlag	This study	
pcDNA3.1-myc 6xHis vector	Invitrogen	Cat# V80020
pcDNA3.1-human PPP2R1A -myc 6xHis	This study	
pcDNA3.1-human PPP2R2B -myc 6xHis	This study	
pCMV3-human ECSIT WT -HA	Sino Biological	Cat# HG14497-CY
pCMV3-human ECSIT T179A -HA	This study	
pCMV3-human ECSIT T179E -HA	This study	
Human ECSIT full length/WT -EGFP	This study	
Human ECSIT 1–300 -EGFP	This study	
Human ECSIT 1–257 -EGFP	This study	
Human ECSIT 1–200 -EGFP	This study	
Human ECSIT 1–100 -EGFP	This study	
Human ECSIT 1–48 -EGFP	This study	
Human ECSIT S88A -EGFP	This study	
Human ECSIT T92A -EGFP	This study	
Human ECSIT S100A -EGFP	This study	
Human ECSIT T179A -EGFP	This study	
Human ECSIT S191A -EGFP	This study	
pcDNA-mt-Sonar	FR Biotechnology	
pC1-mitoRexYFP	Addgene	Cat# 60246
MitoSypHer Red	This study	
**Antibodies**		
Mouse monoclonal anti-SOX2	R and D Systems	Cat# MAB2018
Rabbit polyclonal anti-SOX2	Millipore	Cat# AB5603
Chicken polyclonal anti-SOX2	Aves Labs	Cat# SOX2-0020
Rabbit polyclonal anti-TBR2	Millipore	Cat# AB2283
Rabbit recombinant monoclonal TBR2 conjugated to Alexa Fluor 647	Abcam	Cat# ab315356
Guinea pig polyclonal anti-NEUN	Millipore	Cat# ABN90P
Mouse monoclonal anti-SATB2	Abcam	Cat# ab51502
Rat monoclonal anti-CTIP2	Abcam	Cat# ab18465
Rabbit polyclonal anti-TBR1	Abcam	Cat# ab31940
Rabbit polyclonal anti-Ki67	Abcam	Cat# ab15580
Rat monoclonal anti-BrdU	Abcam	Cat# ab6326
Rat monoclonal anti-PH3	Abcam	Cat# ab10543
Rabbit polyclonal anti-cleaved Caspase 3	Cell Signaling Technology	Cat# 9661S
Rabbit polyclonal anti-EPHA2	Cell Signaling Technology	Cat# 6997S
Mouse monoclonal anti-EPHA2	Santa Cruz Biotechnology	Cat# sc-398832
Rabbit monoclonal anti-phospho-EPHA2 (Ser897)	Cell Signaling Technology	Cat# 6347S
Rabbit monoclonal anti-ECSIT	Thermo Fisher Scientific	Cat# PA5-20082
Mouse monoclonal anti-ACAD9	OriGene Technologies	Cat# TA811802
Mouse monoclonal anti-TOM20	Abcam	Cat# ab56783
Rabbit polyclonal anti-Mitofilin	Novus Biologicals	Cat# NB100-1919
Mouse monoclonal anti-phospho-Threonine	Cell Signaling Technology	Cat# 9386S
Mouse monoclonal anti-phospho-Tyrosine	Santa Cruz Biotechnology	Cat# sc-7020
Rabbit polyclonal anti-PP2A catalytic subunit	R and D Systems	Cat# AF1653
Rabbit polyclonal anti-phospho-PP2A (Y307)	R and D Systems	Cat# AF3989
Rabbit polyclonal anti-LC3B	Cell Signaling Technology	Cat# 2775
Mouse monoclonal anti-beta actin	Sigma-Aldrich	Cat# A5441
Mouse monoclonal anti-alpha tubulin	Proteintech	Cat# 66031-1-Ig
Mouse monoclonal anti-GAPDH	Santa Cruz Biotechnology	Cat# sc-32233
Mouse monoclonal anti-His tag	Santa Cruz Biotechnology	Cat# sc-53073
Rabbit polyclonal anti-HA tag	Bethyl	Cat# A190-108A
Mouse monoclonal anti-c-Myc	Santa Cruz Biotechnology	Cat# sc-40
Rabbit polyclonal anti-GFP	Thermo Fisher Scientific	Cat# A-11122
Guinea pig polyclonal anti-GFP	Synaptic Systems	Cat# 132 005
Chicken polyclonal anti-mCherry	Aves Labs	Cat# MCHERRY-0020
Mouse OXPHOS human WB antibody cocktail	Thermo Fisher Scientific	Cat# 45-8199
Rabbit polyclonal anti-PPP2R2B	Proteintech	Cat# 13123-1-AP
Rabbit polyclonal anti-Calreticulin	Abcam	Cat# ab2907
Rabbit polyclonal anti-Calnexin	Abcam	Cat# ab22595
Rabbit polyclonal anti-VDAC1	Proteintech	Cat# 55259-1-AP
HRP-conjugated sheep anti-mouse IgG	GE Healthcare	Cat# NA931
HRP-conjugated donkey anti-rabbit IgG	GE Healthcare	Cat# NA934
Alexa Fluor Plus 405 conjugated goat anti-mouse IgG	Thermo Fisher Scientific	Cat# A55057
Alexa Fluor 568 conjugated goat anti-mouse IgG	Thermo Fisher Scientific	Cat# A-11004
Alexa Fluor 405 conjugated goat anti-rabbit IgG	Thermo Fisher Scientific	Cat# A-31556
Alexa Fluor 488 conjugated goat anti-rabbit IgG	Thermo Fisher Scientific	Cat# A-11008
Alexa Fluor 647 conjugated goat anti-rabbit IgG	Thermo Fisher Scientific	Cat# A-21245
Alexa Fluor 647 conjugated goat anti-rat IgG	Thermo Fisher Scientific	Cat# A-21247
Alexa Fluor Plus 405 conjugated goat anti-chicken IgY	Thermo Fisher Scientific	Cat# A48260
Alexa Fluor 568 conjugated goat anti-chicken IgY	Thermo Fisher Scientific	Cat# A-11041
Alexa Fluor 488 conjugated goat anti-guinea pig IgG	Thermo Fisher Scientific	Cat# A-11073
Alexa Fluor 647 conjugated goat anti-guinea pig IgG	Thermo Fisher Scientific	Cat# A-21450
**Oligonucleotides and other sequence-based reagents**		
sgRNA-human *EPHA2* #1: ACGTGGAGGAGCGCTCCGTG	Macrogen	
sgRNA-human *EPHA2* #2: ACAGGCACCGATATCCTGGA	Macrogen	
sgRNA-human *EPHA2* #3: ACGCCACGTGAAGCTGAACG	Macrogen	
shRNA mouse *Epha2*: GGCGTGGTCTCTAAATACA	Macrogen	
Mouse *Epha2* qPCR forward primer: AGAAGTTGTCTCTGTCGGCG	Macrogen	
Mouse *Epha2* qPCR reverse primer: AGGCAGAAACCGACTGCC	Macrogen	
Mouse *Gapdh* qPCR forward primer: CACTGAAGGGCATCTTGG	Macrogen	
Mouse *Gapdh* qPCR reverse primer: TTACTCCTTGGAGGCCATG	Macrogen	
**Chemicals, enzymes and other reagents**		
5-Bromo-2’-deoxyuridine (BrdU)	Sigma-Aldrich	Cat# B5002
NAD^+^	Roche	Cat# 10127965001
NMN	Sigma-Aldrich	Cat# N3501
LB-100	Selleck Chemicals	Cat# S7537
Doxycycline (Hyclate)	Stem Cell Technologies	Cat# 72742
Tetramethylrhodamine methyl ester perchlorate (TMRM)	Sigma-Aldrich	Cat# T5428
Rotenone	Sigma-Aldrich	Cat# R8875
Oligomycin A	Sigma-Aldrich	Cat# 75351
Carbonyl cyanide 4-(trifluoromethoxy)phenylhydrazone (FCCP)	Sigma-Aldrich	Cat# C2920
Proteinase K	Sigma-Aldrich	Cat# P2308
Triton X-100	Sigma-Aldrich	Cat# T9284
Phenylmethylsulfonyl fluoride (PMSF)	Sigma-Aldrich	Cat# P7626
MitoSOX Mitochondrial Superoxide Indicators	Invitrogen	Cat# M36008
Acetone	Burdick&Jackson	Cat# AH010-4
Urea	Sigma-Aldrich	Cat# 51456
Ammonium bicarbonate	Sigma-Aldrich	Cat# A6141
Dithiothreitol	Sigma-Aldrich	Cat# D0632
Iodoacetamide	Sigma-Aldrich	Cat# I1149
Trifluoroacetic acid	Thermo Scientific	Cat# 85183
Toluene	Sigma-Aldrich	Cat# 244511
tert-Butyl methyl ether (MTBE)	Sigma-Aldrich	Cat# 650560
Ammonium formate	Sigma-Aldrich	Cat# 70221
Ammonium acetate	Sigma-Aldrich	Cat# 73594
Formic acid	Sigma-Aldrich	Cat# 533002
Water (LC-MS grade)	Merck	Cat# 115333
2-propanol	Merck	Cat# 102781
Methanol	Merck	Cat# 103726
Acetonitrile	J.T.Baker	Cat# 9017-88
DMEM	Welgene	Cat# LM001-05
Fetal bovine serum (FBS)	Corning	Cat# 35-015-CV
Penicillin/Streptomycin (P/S)	HyClone	Cat# SV30010
Antibiotic-Antimycotic (A/A)	Gibco	Cat# 15240062
HBSS	Gibco	Cat# 14175-095
MEM	HyClone	Cat# SH30024.01
Lipofectamine 2000	Invitrogen	Cat# 11668019
Trypsin	HyClone	Cat# SH30042.02
DNase I	Sigma-Aldrich	Cat# DN25
Neurobasal medium	Gibco	Cat# 21103049
B-27 Supplement	Gibco	Cat# 17504001
N-2 Supplement	Gibco	Cat# 17502048
GlutaMAX Supplement	Gibco	Cat# 35050061
Poly-D-lysine hydrobromide	Sigma-Aldrich	Cat# P0899
Laminin	Corning	Cat# 354232
Phosphatase inhibitor cocktail	Thermo Scientific	Cat# A32957
Protease inhibitor cocktail	Thermo Scientific	Cat# A32963
Clarity Western ECL Substrate	Bio-Rad	Cat# 1705061
Protein A agarose beads	Roche	Cat# 5015979001
basic fibroblast growth factor (bFGF)	Peprotech	Cat# 100-18B
epidermal growth factor (EGF)	R and D Systems	Cat# 2028-EG
DMEM/F12	Gibco	Cat# 11330032
KnockOut Serum Replacement	Gibco	Cat# 10828010
MEM Non-Essential Amino Acids Solution	Gibco	Cat# 11140050
2-Mercaptoethanol	Gibco	Cat# 21985023
Collagenase, Type IV	Gibco	Cat# 17104019
Dorsomorphin	Stem Cell Technologies	Cat# 72102
A 83-01	Stem Cell Technologies	Cat# 72022
CHIR99021	Stem Cell Technologies	Cat# 72052
SB431542 (Hydrate)	Stem Cell Technologies	Cat# 72232
Matrigel	Corning	Cat# 354277
Insulin	Sigma-Aldrich	Cat# I9278
TRIzol	Virginia Tech BioTechnology	Cat# TS200-001
FastStart Universal SYBR Green Master (Rox)	Roche	Cat# 4913914001
Isoflurane	Hana Pharm	
Fast Green FCF	Sigma-Aldrich	Cat# 68724
Surgipath FSC22 Clear OCT solution	Sakura Finetek	Cat# HIO-0051
CAS-Block Histochemical Reagent	Invitrogen	Cat# 008120
Fluorescence Mounting Medium	Agilent Technologies	Cat# S302380-2
Pierce BCA Protein Assay Kit	Thermo Scientific	Cat# 23227
Mitochondria Isolation Kit for Cultured Cells	Thermo Scientific	Cat# 89874
Complex I Enzyme Activity Microplate Assay Kit (Colorimetric)	Abcam	Cat# ab109721
MitoXpress Xtra Oxygen Consumption Assay	Agilent Technologies	Cat# MX-200-4
Seahorse XF Mito Stress Test Kit	Agilent Technologies	Cat# 103015-100
Seahorse XF Glycolytic Rate Assay Kit	Agilent Technologies	Cat# 103344-100
Duolink In Situ Detection Reagents Red	Sigma-Aldrich	Cat# DUO92008
Duolink In Situ PLA Probe Anti-Rabbit PLUS	Sigma-Aldrich	Cat# DUO92002
Duolink In Situ PLA Probe Anti-Mouse MINUS	Sigma-Aldrich	Cat# DUO92004
Invitrogen SuperScript IV Reverse Transcriptase	Invitrogen	Cat# 18-090-050
EndoFree plasmid maxi kit	QIAGEN	Cat# 12362
**Software**		
ImageJ (Fiji)	Schindelin et al, 2012 (PMID: 22743772)	
EasyPubPlot shiny app	Tien et al, 2025 (PMID: 40053871)	
Imaris	Bitplane	RRID: SCR_007370
Olympus cellSens software	Olympus	RRID: SCR_016238
GraphPad Prism	GraphPad	RRID: SCR_002798
R for statistical computing (version 4.2.3)	R Core Team	RRID: SCR_001905
MATLAB R2022b	MATLAB	RRID: SCR_001622
**Other**		
Olympus Confocal Laser Scanning Microscope Fluoview FV3000	Olympus	
ECM 830 Square Wave Electroporation System	Harvard Apparatus	Cat# W3 45-0052
StepOnePlus Real-Time PCR System	Applied Biosystems	Cat# 15360337
Infinite M200 PRO Multimode Microplate Reader	TECAN	Cat# 490016-280
Q-Exactive Plus Orbitrap	Thermo Fisher Scientific	RRID: SCR_020556
Transmission electron microscope Tecnai G2	Thermo Fisher Scientific	RRID: SCR_021365
Seahorse XF Pro Analyzer	Agilent Technologies	

### Ethical statement

All animal experiments were conducted with ethical approval from the Institutional Animal Care and Use Committee (IACUC) of Pohang University of Science and Technology, under protocols POSTECH-2022-0086, POSTECH-2023-0096, and POSTECH-2024-0092. All procedures adhered strictly to the approved institutional guidelines.

### Animals

Pregnant C57BL/6J mice (Hyochang Science, Daegu, South Korea) were used for *in utero* electroporation and primary NSPC culture experiments. The animals were maintained under a 12-h light/12-h dark photoperiod, with unrestricted access to food and water. Environmental conditions were controlled at a temperature of 22 ± 2 °C and relative humidity of 50 ± 5%.

### Antibodies and plasmids

Anti-SOX2 mouse (R and D Systems, Cat# MAB2018), anti-SOX2 rabbit (Millipore, Cat# AB5603), anti-SOX2 chicken (Aves Labs, Cat# SOX2-0020), anti-TBR2 rabbit (Millipore, Cat# AB2283), anti-NEUN guinea pig (Millipore, Cat# ABN90P), anti-SATB2 mouse (Abcam, Cat# ab51502), anti-CTIP2 rat (Abcam, Cat# ab18465), anti-TBR1 rabbit (Abcam, Cat# ab31940), anti-Ki67 rabbit (Abcam, Cat# ab15580), anti-BrdU rat (Abcam, Cat# ab6326), anti-PH3 rat (Abcam, Cat# ab10543), anti-cleaved Caspase 3 rabbit (Cell Signaling Technology, Cat# 9661S), anti-EPHA2 rabbit (Cell Signaling Technology, Cat# 6997S), anti-phospho-EPHA2 (Ser897) rabbit (Cell Signaling Technology, Cat# 6347S), anti-ECSIT rabbit (Thermo Fisher Scientific, Cat# PA5-20082), anti-GFP guinea pig (Synaptic Systems, Cat# 132 005), anti-mCherry chicken (Aves Labs, Cat# MCHERRY-0020) were used for immunohistochemistry experiments for 1:200-1:500. Anti-TOM20 mouse (Abcam, Cat# ab56783), anti-ACAD9 mouse (OriGene Technologies, Cat# TA811802) were used for immunocytochemistry experiments for 1:500. Alexa Fluor Plus 405 and 568 conjugated goat anti-mouse IgG (Cat# A55057 and Cat# A-11004); Alexa Fluor 405, 488, and 647 conjugated goat anti-rabbit IgG (Cat# A-31556, Cat# A-11008, and Cat# A-21245); Alexa Fluor 647 conjugated goat anti-rat IgG (Cat# A-21247); Alexa Fluor Plus 405 and 568 conjugated goat anti-chicken IgY (Cat# A48260 and Cat# A-11041); Alexa Fluor 488 and 647 conjugated goat anti-guinea pig IgG (Cat# A-11073 and Cat# A-21450), all from Thermo Fisher Scientific, were used as secondary antibodies for 1:200.

The following plasmid constructs were engineered and employed for immunoprecipitation and immunoblotting analyses: pcDNA3.1-human EPHA2 WT-3xFlag, pcDNA3.1-myc 6xHis vector, pcDNA3.1-human PPP2R1A-myc 6xHis, pcDNA3.1-human PPP2R2B-myc 6xHis, pCMV3-human ECSIT WT/T179A/T179E-HA. Details regarding additional plasmid constructs are provided in the relevant sections.

### Cell culture, transfection, and lentiviral transduction

HEK293T and Neuro-2a cells were maintained in high-glucose DMEM (Welgene, Cat# LM001-05) supplemented with 10% fetal bovine serum (FBS, Corning, Cat# 35-015-CV) and 1% antibiotic-antimycotic (Gibco, Cat# 15240062). SH-SY5Y neuroblastoma cells were cultured in MEM (HyClone, Cat# SH30024.01) with the same concentrations of FBS and antibiotics. Cell identity was confirmed by morphological inspection, and absence of mycoplasma contamination was verified using a commercial detection kit. All cells were transfected by Lipofectamine 2000 (Invitrogen, Cat# 11668019) according to the manufacturer’s protocol.

To generate EPHA2-knockout (KO) cells, SH-SY5Y cells were transfected with a CRISPR-Cas9 ribonucleoprotein complex containing Cas9 nuclease and guide RNAs (sgRNAs). The following target DNA sequences for human *EPHA2* were used: 5’-ACGTGGAGGAGCGCTCCGTG-3’, 5’-ACAGGCACCGATATCCTGGA-3’, and 5’- ACGCCACGTGAAGCTGAACG-3’. Following sgRNAs/Cas9 delivery via Lipofectamine 2000, cells were selected with 1 mg/mL Geneticin (Gibco, Cat# 10131035, concentration determined by kill‑curve) for 4 days. Single-cell clones were then isolated by limiting dilution, expanded over 10–14 days, and screened for EPHA2 loss by Western blotting.

For inducible re-expression, EPHA2 WT or 5A mutant constructs were subcloned into a TET-ON lentiviral vector pCW57.1 (Addgene #41393). To prepare lentivirus, HEK293T cells were transfected with the packaging plasmids psPAX2 (Addgene #12260), pMD2.G (Addgene #12259), and pCW57.1-human EPHA2 WT or EPHA2 5A. EPHA2 KO cells were transduced and, 48 h later, subjected to 1 µg/mL Puromycin (Gibco, Cat# A1113803) selection for 4–5 days to isolate single clones. To verify uniform expression, clones were treated with 0.5 µg/mL Doxycycline (Stem Cell Technologies, Cat# 72742) for 24 h, lysed, and analyzed by Western blotting. Only those clones exhibiting comparable EPHA2 WT and EPHA2 5A induction levels were chosen for subsequent experiments. They are briefly referred to as EPHA2 WT and EPHA2 5A cells in the main text and the remainder of the Methods section.

### *In utero* electroporation (IUE) of embryonic mouse neocortex

Pregnant C57BL/6J mice at embryonic day 13.5 (E13.5) were anesthetized with isoflurane (induction in a chamber: 2.8%, surgery via mask: 2.5%, Hana Pharm Corporation). Plasmid DNA (1.5–2 µg/µL, mixed with 0.1% Fast Green) in either pLL3.7 (This paper, pLL3.7-EGFP/RFP vector and pLL3.7-EGFP/RFP shRNA mouse EPHA2) or pCAG backbone (This paper, pCAG-human EPHA2 WT/5A/K646R/D739N T2A RFP, pCAG-RFP vector, pCAG-EGFP vector, pCAG-human ECSIT WT T2A EGFP, pCAG-human ECSIT WT T2A RFP, pCAG-human ECSIT ΔMTS T2A EGFP, pCAG-human ECSIT ΔMTS T2A RFP, pCAG-human ECSIT T179E T2A EGFP) were prepared using EndoFree plasmid maxi kit (QIAGEN, Cat# 12362) and injected into the lateral ventricles of embryos through pulled glass microcapillaries (Drummond Scientific, Cat# 3-000-203-G/X). Electroporation was performed using a tweezer-type electrode containing two disc-shaped electrodes positioned at an appropriate angle. Five electric pulses (50 ms duration, 35 V) were delivered at 950-ms intervals via an electroporator (Harvard Apparatus, Cat# W3 45-0052). Embryos were returned to the uterus, the incision sutured, and the dam returned to its home cage. To analyze S-phase entry or cell cycle exit, BrdU (Sigma-Aldrich, Cat# B5002) was administered by intraperitoneal injection (50 mg/kg in PBS) either 2 or 24 h, respectively, before sacrifice. Mice were euthanized at E14.5, E15.5, or E18.5, depending on experimental purpose. To minimize the sample-by-sample variation, we analyzed the samples with comparable tissue quality, electroporated region, and total electroporation efficiency in each experimental set.

To investigate the role of mitochondrial NAD^+^/NADH in NSPC proliferation, mitochondria-targeted *Lb*NOX (This paper; derived from Addgene #74448) or Ndi1 (This paper; derived from Addgene #127503) under the pCAG promoter were electroporated to E13.5 brains, and embryos were analyzed at E15.5. To examine the functional contribution of mitochondria–endoplasmic reticulum contact sites (MAMs), an artificial outer mitochondrial membrane–endoplasmic reticulum (OMM–ER) linker (a gift from Hyun-Woo Rhee) under pCAG backbone was introduced at E13.5. Brains were subsequently analyzed either for laminar distribution at E15.5 or subjected to live-cell imaging following NSPC culture.

### Pharmacological treatment

NAD^+^ (10 mg in 100 μL saline) or the NAD^+^ precursor NMN (500 mg/kg) was administered to pregnant dams via daily intraperitoneal injection from E12 to E15, modified from a published protocol (Subburaju et al, 2020). In a subset of experiments, IUE was additionally performed at E13.5.

Low-dose Rotenone (0.1 μM) or vehicle (DMSO) was applied to NSPCs at DIV2 to induce mitochondrial Complex I dysfunction. NSPC proliferation and MAM integrity were subsequently assessed at 6 or 48 h following treatment.

### Mt-SoNar imaging

Pregnant C57BL/6J mice at E13.5 were anesthetized with isoflurane (induction: 2.8%, maintenance: 2.5%). DNA solution containing pCAG plasmids encoding target genes and mt-SoNar (FR Biotechnology) (1.5–2 µg/µL, mixed with 0.1% Fast Green) was microinjected into the embryonic lateral ventricles through pulled glass microcapillaries. Electroporation followed as above. Mice were euthanized at E14.5, and neural stem cells from cortices were isolated and cultured in Neurobasal medium (Gibco, Cat# 21103049) supplemented with 1× GlutaMAX (Gibco, Cat# 35050061), 1× B-27 supplement (Gibco, Cat# 17504001), 1× N-2 supplement (Gibco, Cat# 17502048), 1× penicillin-streptomycin (P/S, HyClone, Cat# SV30010), 20 ng/mL epidermal growth factor (EGF, R&D Systems, Cat# 2028-EG), and 20 ng/mL basic fibroblast growth factor (bFGF, Peprotech, Cat# 100-18B). On day 2 in vitro (DIV2), cultured neural stem cells were embedded in Tyrode’s buffer (NaCl 138 mM, KCl 3.7 mM, HEPES 20 mM, MgSO_4_ 1.2 mM, KH_2_PO_4_ 1.2 mM, CaCl_2_ 1 mM, and D-glucose 5 mM). Cells were then imaged using confocal microscopy (Olympus) with a 100× (1.35 NA oil) objective lens with dual excitation wavelengths at 405 nm and 488 nm. Emission was collected at 510–550 nm to detect the signals of mt-SoNar (Hu et al, 2021).

### Immunohistochemistry (IHC)

Brains were rinsed with PBS and subsequently fixed overnight in a solution containing 4% paraformaldehyde (Sigma-Aldrich, Cat# 158127) and 4% sucrose (Daejung) in PBS, followed by immersion in 30% sucrose solution in PBS for 24 h. After cryoprotection, brains were embedded in OCT compound (Sakura Finetek, Cat# HIO-0051), frozen, and sectioned at 20 µm thickness using a cryostat (Leica Biosystems). Tissue sections were mounted on Superfrost Plus microscope slides (Thermo Fisher Scientific, Cat# J1800AMNZ) and examined using fluorescence microscopy to identify electroporated fluorescent cells. Dried sections were washed with PBS. Antigen retrieval was performed using sodium citrate buffer (10 mM sodium citrate, 0.05% Tween-20, pH 6.0) at 95 °C for 10 min. Slides were then permeabilized with 0.3% Triton X-100 (Sigma-Aldrich) in PBS for 10 min. Nonspecific binding was blocked by incubating samples with CAS-Block Histochemical Reagent (Invitrogen, Cat# 008120) at room temperature for 3 h. Subsequently, tissues were incubated with primary antibodies (1:500) overnight at 4 °C, followed by incubation with secondary antibodies (1:200) at room temperature for 2 h. Nuclear staining was carried out using Hoechst 33342 (Thermo Fisher Scientific, Cat# H3570). Coverslips were mounted using Dako mounting media (Agilent Technologies, Cat# S302380-2). Confocal IHC images were acquired using an FV3000 laser-scanning microscope (Olympus), equipped with objective lenses of 10× (0.4 NA), 20× (0.75 NA), or 60× (1.35 NA oil), depending on the experiment, with Z-stacks captured at 1-µm intervals.

### Forebrain organoid culture

Cerebral organoids with regional forebrain identity were generated based on a previously established protocol (Qian et al, 2018). Human embryonic stem cells (hESCs, WA09, WiCell) were maintained on mouse embryonic fibroblast feeder layers in standard stem cell culture media, which included DMEM/F12 (Gibco, Cat# 11330032), 20% knockout serum replacement (KOSR, Gibco, Cat# 10828010), 1× GlutaMax (Gibco, Cat# 35050061), 1× non-essential amino acids (NEAA, Gibco, Cat# 11140050), 1× penicillin-streptomycin (HyClone, Cat# SV30010), 1× β-mercaptoethanol (Gibco, Cat# 21985023), and 10 ng/mL basic fibroblast growth factor (Peprotech, Cat# 100-18B).

To initiate forebrain organoid formation, WA09 stem cell colonies, ~1–1.5 mm in diameter, were detached using 1 mg/mL collagenase IV (Thermo Fisher Scientific, Cat# 17104019) dissolved in DMEM/F12 for 30 min, with treatment extended up to 2 h if needed. Detached colonies were transferred to ultralow attachment six-well plates (Corning) and cultured for four days in Forebrain first media, composed of DMEM/F12, 20% KOSR, 1× GlutaMax, 1× NEAA, 1× β-mercaptoethanol, 1× penicillin-streptomycin, 2 μM dorsomorphin (Stem Cell Technologies, Cat# 72102), and 2 μM A 83-01 (Stem Cell Technologies, Cat# 72022).

On days 5 and 6, half of the media was replaced with Forebrain second media, containing DMEM/F12, 1× N-2 supplement, 1× GlutaMax, 1× NEAA, 1× penicillin-streptomycin, 1 μM CHIR99021, and 1 μM SB431542 (Hydrate) (both from Stem Cell Technologies, Cat# 72052 and Cat# 72232). By day 7, embryoid bodies were embedded in a Matrigel (Corning, Cat# 354277) and Forebrain second media mixture (3:2 ratio), placed in ultralow attachment plates, and incubated at 37 °C for 30 min to solidify. Organoids were then cultured for an additional 7 days in Forebrain second media.

On day 14, the Matrigel matrix was mechanically disrupted by pipetting with a 10-mL pipette two to three times. Subsequently, organoids were maintained in Forebrain third media, which included DMEM/F12, 1× N-2 supplement, 1× B-27 supplement, 1× GlutaMax, 1× NEAA, 1× β-mercaptoethanol, 1× penicillin-streptomycin, and 2.5 μg/mL insulin (Sigma-Aldrich, Cat# I9278), under continuous agitation at 75 rpm. On day 42, organoids were collected for immunohistochemistry.

### Image acquisition and analysis

Confocal IHC images were acquired using an FV3000 laser-scanning microscope (Olympus) equipped with a 20× (0.75 NA) objective lens, capturing Z-stacks at 1-µm intervals.

Raw images were processed using Imaris software (Bitplane). Baseline subtraction was applied to minimize background fluorescence. Based on the intensity histogram, Imaris determined an automatic threshold value that separated foreground objects from the background, then used this threshold to generate surfaces for each channel. Precisely, confocal Z-stack images were segmented in Imaris to reconstruct three-dimensional surfaces corresponding to electroporated cells (GFP/RFP) and for each marker (e.g., BrdU, Ki67, and SOX2), following baseline subtraction and automatic thresholding. Co-localized objects between reconstructed surfaces were identified using the ImarisColoc XTension module and counted within predefined ROIs (VZ/SVZ, IZ, and CP delineated based on Hoechst density). Quantitative values are expressed as the proportion of electroporated cells co-labeled with the marker divided by the total number of electroporated cells within the same ROI and embryo. Specifically for Fig. 3A–D, the percentage of Ki67^+^ BrdU^+^GFP^+^ cells (representing cells that remain in the cell cycle) was first quantified within the BrdU^+^GFP^+^ population. The percentage of Ki67^−^ BrdU^+^GFP^+^ cells (representing cells that have exited the cell cycle) was subsequently calculated by subtracting this value from 100%. Quantitative data were extracted from the Imaris Statistics tab, with surface area as the parameter for comparison. For PH3 and cleaved Caspase 3, the Spots analysis function in Imaris was applied to detect signal-positive cells. The total number of spots co-localizing with electroporated signals within the defined field of view was quantified for comparison.

### Quantitative RT-PCR (qRT-PCR) sampling and analysis

Cortices at the indicated embryonic day were homogenized, and total RNA was extracted using TRIzol reagent (Virginia Tech BioTechnology, Cat# TS200-001). cDNA was synthesized using Invitrogen SuperScript IV Reverse Transcriptase (Invitrogen, Cat# 18-090-050) and oligo-dT primers (Invitrogen, Cat# 18418020). qPCR was conducted with FastStart SYBR Green Master Mix (Roche, Cat# 4913914001) on a StepOnePlus Real-Time PCR System (Applied Biosystems), using *Gapdh* for normalization via the 2^−ΔΔCt^ method. The following primers were used for the housekeeping gene and *Epha2* target gene: mouse *Gapdh*, CACTGAAGGGCATCTTGG and TTACTCCTTGGAGGCCATG; mouse *Epha2*, AGAAGTTGTCTCTGTCGGCG and AGGCAGAAACCGACTGCC.

### Proximity ligation assay (PLA)

The spatial interaction between ECSIT and ACAD9 at the inner mitochondrial membrane was assessed using the Duolink in situ proximity ligation assay (Sigma-Aldrich, Cat# DUO92002, Cat# DUO92004, and Cat# DUO92008), following the manufacturer’s instructions. Cells were fixed with 4% paraformaldehyde, permeabilized using 0.3% Triton X-100, and blocked prior to overnight incubation at 4 °C with primary antibodies: rabbit anti-ECSIT (1:500) and mouse anti-ACAD9 (1:500). The PLA procedure continued with a 1-h incubation with PLA probes, a 30-minute ligation step, and a 2-h amplification at 37 °C. Nuclei were counterstained with Hoechst for visualization. Confocal images were captured using an Olympus FV3000 microscope equipped with a UPLSAPO 100× (1.35 NA oil) objective. PLA signals, indicating ECSIT–ACAD9 proximity, were visualized via Texas Red-labeled probes. Quantification of PLA signal puncta per cell was performed using Imaris software.

### Transmission electron microscopy

Cells were prefixed with 2% paraformaldehyde (Electron Microscopy Sciences, Cat# 19210) and 2.5% glutaraldehyde (Electron Microscopy Sciences, Cat# 16200) in 0.15 M sodium cacodylate buffer (pH 7.4) for 1 h, followed by three washes. Post-fixation was done on ice using 2% osmium tetroxide and 1.5% potassium ferrocyanide in 0.1 M cacodylate buffer for 1 h. After rinsing with ice-cold water, cells were stained overnight at 4 °C with 1% uranyl acetate (Electron Microscopy Sciences, Cat# 22400) in the dark, then washed again. Dehydration was carried out using graded ethanol (30–100%) at 4 °C, followed by resin infiltration with ethanol:Epon 812 mixtures (3:1, 1:1, 1:3), and overnight incubation in pure resin. Samples were polymerized at 60 °C for 48 h. Ultrathin sections (60 nm) were prepared using an ultramicrotome (UC7, Leica Microsystems) and mounted on formvar-coated grids. Sections were stained with UranyLess (Electron Microscopy Sciences, Cat# 22409) and 3% lead citrate (Electron Microscopy Sciences, Cat# 22410) for contrast. TEM imaging was performed with a Tecnai G2 microscope (120 kV, Thermo Fisher Scientific) using a US1000XP CCD detector (Gatan). Image analysis was conducted using the brush selection tool in Microscopy Image Browser (MIB), enabling the selective identification and masking of mitochondria. Mitochondrial morphology was quantified based on four parameters: number per cell, perimeter (µm), area (µm^2^), and aspect ratio (major axis/minor axis).

### Subcellular fractionation

Mouse brain fractionation was performed by differential ultracentrifugation using a Percoll gradient, as previously described (Thi My Nhung et al, 2023). Fractions corresponding to whole brain lysate, ER, cytosol, pure mitochondria, and MAMs were collected and lysed in modified RIPA buffer. Protein concentrations were quantified using a BCA assay (Thermo Fisher Scientific, Cat# 23228). Fractions were subsequently analyzed by immunoblotting using anti-ECSIT (Thermo Fisher Scientific, Cat# PA5-20082), anti-PPP2R2B/2ABB (Proteintech, Cat# 13123-1-AP), anti-Calreticulin (Abcam, Cat# ab2907), anti-VDAC1 (Proteintech, Cat# 55259-1-AP), and anti-alpha tubulin (Proteintech, Cat# 66031-1-Ig) antibodies.

### Proteinase K protection assay

Proteinase K protection assay was performed to determine the submitochondrial localization of ECSIT in SH-SY5Y cells. Mitochondria were isolated using the Mitochondria Isolation Kit for Cultured Cells (Thermo Scientific, Cat# 89874) according to the manufacturer’s instructions and resuspended in ice-cold mitochondrial isolation buffer. Equal amounts of mitochondrial protein were incubated with proteinase K (10 µg of proteinase K/mg of mitochondrial protein) on ice for 30 min in the absence or presence of 0.5% Triton X-100. The proteolytic reaction was terminated by adding phenylmethylsulfonyl fluoride (PMSF, 2 mM). Samples were then mixed with SDS sample buffer, boiled, and analyzed by SDS-PAGE followed by immunoblotting. TOM20 and Mitofilin were used as markers for the outer mitochondrial membrane and inner mitochondrial membrane, respectively.

### Immunoprecipitation and immunoblotting

Mouse brain homogenates or transfected cell lysates were prepared by washing specimens in ice-cold PBS, then sonicating them in lysis buffer (50 mM Tris-HCl, pH 8.0, 50 mM HEPES, pH 7.4, 150 mM NaCl, 5 mM EDTA, 1.5 mM MgCl_2_, protease/phosphatase inhibitors) containing either 1% NP-40 (Thermo Scientific) (brain) or 0.1% Triton X-100 (cells). After clarification, 1–2 µg of anti-EPHA2 antibody (brain lysate) or the indicated antibodies (anti-ECSIT rabbit, anti-His tag mouse, anti-HA tag rabbit, anti-GFP tag rabbit) (cell lysate) was added, and the mixture was rotated overnight at 4 °C, followed by incubation with 35 µL Protein A agarose beads (Roche) under the same conditions. Immune complexes were washed three times with lysis buffer, resuspended in 5× sodium dodecyl sulfate (SDS) loading buffer (2% SDS, 60 mM Tris, pH 6.8, 24% glycerol, 0.1% bromophenol blue, 5% β-mercaptoethanol), boiled at 70 °C for 15 min, resolved on 8–10% SDS-PAGE, and transferred to PVDF membranes. Membranes were blocked in either 5% skim milk (Seoul Milk) in TBST (20 mM Tris pH 8.0, 137.5 mM NaCl, 0.25% Tween-20) or 5% bovine serum albumin (GeorgiaChem) in TBST for 1 h at room temperature, incubated overnight at 4 °C with primary antibodies (1:1000). The following primary antibodies were utilized: anti-phospho-Threonine mouse (Cell Signaling Technology, Cat# 9386S), anti-phospho-Tyrosine mouse (Santa Cruz Biotechnology, Cat# sc-7020), anti-PP2A (R&D Systems, Cat# AF1653), anti-phospho-PP2A (R&D Systems, Cat# AF3989), anti-beta actin mouse (Sigma-Aldrich, Cat# A5441), anti-alpha tubulin mouse (Proteintech, Cat# 66031-1-Ig), anti-GAPDH mouse (Santa Cruz Biotechnology, Cat# sc-32233), anti-c-Myc mouse (Santa Cruz Biotechnology, Cat# sc-40), mouse OXPHOS human WB antibody cocktail (Thermo Fisher Scientific, Cat# 45-8199), anti-Mitofilin (Novus Biologicals, Cat# NB100-1919). Membranes were then washed (3 × 10 min), incubated with horseradish peroxidase-conjugated secondary antibodies (GE Healthcare, Cat# NA931 and Cat# NA934, 1:10,000) for 2 h at room temperature, and developed by ECL (Bio-Rad, Cat# 1705061) on an Azure Biosystems imager.

### Mass spectrometry

For proteomic analysis, EPHA2 WT and 5A SH-SY5Y cells were seeded at 1.5 × 10^7^ cells per T75 flask. After 24 h, they were treated with 0.5 µg/mL Doxycycline for 24 h before harvesting. Mitochondria were isolated using the Mitochondrial Isolation Kit (Thermo Scientific, Cat# 89874) as per the manufacturer’s specifications. Pellets were lysed with 2% SDS in LC-grade water (Merck, Cat# 115333) containing protease inhibitors, followed by sonication (three sets, 50% amplitude, 1 s on, 15 pulses per set) and centrifugation at 12,000×*g*, 25 °C. Proteins were precipitated with cold acetone (Burdick&Jackson, Cat# AH010-4, −20 °C), washed with cold acetone containing 10% TBS, centrifuged, and air-dried. Pellets were denatured overnight in 8 M urea (Sigma-Aldrich, Cat# 51456) in 50 mM ammonium bicarbonate (ABC) buffer (Sigma-Aldrich, Cat# A6141), sonicated (three sets, 50% amplitude, 1 s on, 10 pulses per set), and quantified by BCA assay.

For reduction and alkylation, 20 µg protein in 8 M urea was treated with 10 mM dithiothreitol (DTT, Sigma-Aldrich, Cat# D0632, 1 h, 37 °C) and 40 mM iodoacetamide (IAA, Sigma-Aldrich, Cat# I1149, 1 h, 37 °C). Samples were diluted to 1 M urea, supplemented with 1 mM CaCl_2_, and digested with trypsin (20:1 protein:trypsin) for 4 h at 37 °C. Peptides were desalted (Pierce C18 Spin Tips), dried, and resuspended in 0.1% trifluoroacetic acid (TFA, Thermo Scientific, Cat# 85183) for LC-MS/MS.

NanoLC-MS/MS was performed using a Q-Exactive Plus mass spectrometer with an Easy Spray C18 column (Thermo Fisher, Cat# ES803A). Peptides were separated with a gradient from 10% to 90% acetonitrile (J.T.Baker, Cat# 9017-88) over 130 min at 300 nL/min. Data were acquired with a 1.5 kV spray voltage and a mass scan range of 350–1800 m/z, with the top ten precursor ions selected for fragmentation.

A label-free data processing workflow with modifications, as described by Hutchings et al, was employed in R version 4.4.2 (Hutchings et al, 2023). In particular, raw mass spectral data were processed using Proteome Discoverer (PD) 2.3 with SEQUEST against the UniProtKB/Swiss-Prot human database. Search parameters included 10 ppm precursor tolerance, 0.02 Da fragment tolerance, and trypsin specificity (up to two missed cleavages). Peptides were identified if marked as “Master” in PD 2.3. Regarding the protein annotation, the peptide and protein false discovery rate (FDR) were controlled below 1%. The data cleaning process was applied, excluding contaminants, peptides that did not match a master protein, peptides without quantitative data, peptides not unique to a protein group, peptides with ranks that were not 1 during the identification, peptides that were not annotated as “unambiguous”, and those with ≥20% missing values. Additionally, only mitochondrial proteins were kept for downstream analyses. Mitochondrial proteins were annotated using the Human MitoCarta3.0 list. The data were then log-transformed and aggregated to the protein level, which then underwent median normalization. Missing data were imputed using the k-NN algorithm.

For data exploration, the log-transformed and normalized protein data underwent Pareto scaling prior to principal component analysis (PCA). The PCA scores plot was generated in MetaboAnalyst 6.0 (Pang et al, 2024) and further customized using the EasyPubPlot shiny app (Tien et al, 2025). Univariate analysis was conducted in R 4.4.2, using the log-transformed median-normalized data. A one-sided *t*-test was first performed for the ECSIT protein with a significance cut-off, *P* value, of 0.05. Next, the limma method was used for single-factor differential analysis of the remaining proteins to identify differential proteins, with a *P* value cut-off of 0.05 and an FDR cut-off of 0.25. The volcano plot, heatmap, and boxplot were visualized via the EasyPubPlot (Tien et al, 2025). A heatmap with proteins grouped by biological pathways was visualized using the ComplexHeatmap R package version 2.22.0 (Gu et al, 2016). Differential proteins were then subjected to over-representation analysis (ORA) using the clusterProfiler R package version 4.14.4 (Yu et al, 2012) with the Reactome knowledgebase. An FDR cut-off of 0.05 was used as the significance threshold to identify altered pathways. The dot plot was visualized using the EasyPubPlot (Tien et al, 2025).

### Seahorse oxygen consumption rate (OCR) assay

Mitochondrial respiration was measured using the Seahorse XF Mito Stress Test Kit (Agilent Technologies, Cat# 103015-100) according to the manufacturer’s instructions. EPHA2 WT or 5A inducible cells were seeded at a density of 2 × 10^4^ cells per well in media containing 0.5 µg/mL Doxycycline in Seahorse XF cell culture microplates and incubated overnight. On the day of the assay, culture medium was replaced with Seahorse XF DMEM assay medium (Cat# 103575-100) supplemented with glucose, L-glutamine and pyruvate, and cells were equilibrated in a non-CO_2_ incubator for 1 h prior to measurement. The OCR was measured under basal conditions and following sequential injections of oligomycin (1.5 μM), FCCP (0.5 μM), and rotenone/antimycin A (0.5 μM). OCR measurements were obtained using the Seahorse XF Pro Analyzer and analyzed with Agilent Seahorse Analytics software.

### Seahorse glycolytic rate assay

Glycolytic activity was measured using the Seahorse XF Glycolytic Rate Assay Kit (Agilent Technologies, Cat# 103344-100) according to the manufacturer’s instructions. IUE of EPHA2 WT or 5A constructs with EGFP reporter was performed at E13.5, and the precisely electroporated regions were microdissected at E14.5 under a fluorescence microscope, followed by primary NSPC culture. Primary NSPCs expressing EPHA2 WT or 5A were seeded at a density of 1.2 × 10^5^ cells per well in Seahorse XF cell culture microplates and incubated overnight. On the day of the assay, culture medium was replaced with Seahorse XF DMEM assay medium supplemented with glucose, L-glutamine, and pyruvate. The assay medium was additionally supplemented with 20 ng/mL EGF and 20 ng/mL basic FGF. Cells were then equilibrated for 1 h in a humidified 37 °C non-CO_2_ incubator prior to measurement. The proton efflux rate (PER) was measured using the Seahorse XF Pro Analyzer following sequential injections of rotenone/antimycin A (0.5 μM) and 2-deoxy-D-glucose (50 mM), and data were analyzed using Agilent Seahorse Analytics software.

### MitoXpress assay

EPHA2 WT or 5A inducible cells were plated in 96-well plates at 4 × 10^4^ cells per well in 200 µL of media containing 0.5 µg/mL Doxycycline. After 24 h, we measured oxygen consumption with the MitoXpress Xtra probe (Agilent Technologies, Cat# MX-200-4) per the manufacturer’s instructions. We added 1 µM MitoXpress to each well, overlaid with 100 µL mineral oil, and recorded time-resolved fluorescence at 650 nm excitation and 380 nm emission (30 µs delay, 100 µs gate) at 37 °C using a TECAN microplate reader Infinite M200 Pro.

### Mitochondrial Complex I enzyme activity

For SY5Y cells, EPHA2 WT or 5A inducible cells were cultured in 150 mm dishes until 90% confluency and treated with 0.5 µg/mL Doxycycline for 24 h. For embryonic brain samples, IUE of EPHA2 WT or 5A constructs with EGFP reporter was performed at E13.5, and the precisely electroporated regions were microdissected at E15.5 under a fluorescence microscope. Mitochondria were isolated using the Mitochondria Isolation Kit for Cultured Cells. The Complex I Enzyme Activity Microplate Assay Kit (Abcam, Cat# ab109721) was employed to immunocapture mitochondrial Complex I from isolated mitochondria. Enzyme activity was determined by measuring the colorimetric reaction through absorbance increase at 450 nm for 60 min. All steps were carried out exactly as specified in the manufacturer’s protocol.

### TMRM staining

For tetramethylrhodamine methyl ester (TMRM, Sigma-Aldrich, Cat# T5428) imaging, EPHA2 WT or 5A inducible cells were seeded onto poly-D-lysine-coated coverslips in 500 µL of medium supplemented with 0.5 µg/mL Doxycycline. After 24 h, the cells were incubated with 20 nM TMRM in Hanks’ Balanced Salt Solution (HBSS) containing 25 mM HEPES, 2 mM CaCl_2_, 1 mM MgSO_4_, 4 mM NaHCO_3_, and 30 mM D-glucose for 30 min at 37 °C. Fluorescence intensity was subsequently recorded using a confocal microscope equipped with a UPLSAPO 20× (0.75 NA) objective in the presence of 5 nM TMRM in the imaging solution. TMRM was excited at 543 nm, and emission was detected at 573 nm. Imaging was performed over 6 min, with images acquired every 15 s. Baseline fluorescence was recorded for the initial 2 min, followed by sequential 2-min recordings after the addition of 2.5 µg/mL oligomycin (Sigma-Aldrich, Cat# 75351) and 5 µM FCCP (Sigma-Aldrich, Cat# C2920), respectively. Data acquisition was conducted using cellSens software.

### MitoSOX staining

To assess mitochondrial superoxide production, EPHA2 WT or 5A inducible cells were seeded onto poly-D-lysine-coated coverslips in 500 µL of medium supplemented with 0.5 µg/mL Doxycycline. After 24 h, the cells were incubated with 1 μM MitoSOX Red (Invitrogen, Cat# M36008) for 30 min. Imaging was performed using a confocal microscope with a UPLSAPO 20× (0.75 NA) objective. MitoSOX Red was excited at 510 nm and emitted at 580 nm. The fluorescence intensity was acquired and quantified using ImageJ.

### MitoRexYFP imaging

To account for the effect of pH on mitoRexYFP signal, the pH-sensitive control protein mitoSypHer red was used. Wild-type and EPHA2 KO SH-SY5Y cells were transfected with empty vector/EPHA2 WT/EPHA2 5A/EPHA2 K646R constructs, together with mitoRexYFP sensor (Addgene #60246) and mitoSypHer red sensor (This paper; derived from Addgene #48252). Imaging was conducted using a confocal microscope with a UPLSAPO 20× (0.75 NA) objective. MitoRexYFP and mitoSypHer red were excited at 488 and 568 nm, respectively. The mitoRexYFP signal, normalized to mitoSypHer red, was calculated to assess the mitochondrial NAD^+^/NADH ratio in individual cells (Bilan et al, 2014).

### Mitophagy analysis

EPHA2 WT or 5A inducible cells were exposed to 0.5 µg/mL Doxycycline for 24 h. Cells were lysed and subjected to immunoblotting using an anti-LC3B antibody. The level of autophagy was quantified by measuring the intensity of the lipidated LC3B-II band relative to GAPDH. Mitophagy was induced by treating cells with carbonyl cyanide m-chlorophenyl hydrazone (CCCP, 20 μM) for 6 h. For immunofluorescence analysis, cells were fixed with 4% paraformaldehyde, permeabilized with 0.3% Triton X-100, and blocked prior to incubation with anti-LC3B (Cell Signaling Technology, Cat# 2775S) and anti-TOM20 antibodies. Images were acquired using a confocal microscope with a UPLSAPO 100× (1.35 NA oil) objective. Mitophagy was evaluated by quantifying LC3B puncta and analyzing the co-localization of LC3B puncta with TOM20-positive mitochondria using Imaris software.

### MAM-BiFC intensity measurement

EPHA2 WT or 5A inducible cells transfected with the MAM-BiFC sensor were exposed to 0.5 µg/mL Doxycycline for 24 h. Fixed samples were imaged using a confocal microscope with a UPLSAPO 20× (0.75 NA) objective. Region of interest (ROI) corresponding to the whole cell were selected, and YFP fluorescence intensity was quantified as the mean gray value in ImageJ after background subtraction.

### Co-localization assay

IUE of EPHA2 WT or 5A was performed at E13.5. Primary NSPCs were isolated at E14.5, cultured in vitro, and fixed at DIV2. Cells were incubated with primary antibodies against ECSIT (1:400), TOM20 (1:200), and the NSPC marker SOX2 (1:500), followed by appropriate fluorescent secondary antibodies. Confocal images were acquired, and co-localization between ECSIT and TOM20 signals was analyzed in SOX2-positive NSPCs. To check MAM integrity, co-localization between Calnexin (Abcam, Cat# ab22595) (1:200) and TOM20 (1:200) signals was analyzed in SOX2-positive NSPCs.

SH-SY5Y wild-type cells were transiently transfected with wild-type or mutant ECSIT-GFP constructs (human ECSIT full length/WT/1–300/1–257/1–200/1–100/1–48/S88A/T92A/S100A/T179A/S191A-EGFP) using Lipofectamine 2000 following the manufacturer’s protocol. After 24 h, cells were fixed with 4% paraformaldehyde, permeabilized using 0.3% Triton X-100, and blocked prior to overnight incubation at 4 °C with anti-TOM20 antibody (1:200). Alexa Fluor 568 conjugated secondary antibody (1:200) was applied for 2 h at room temperature.

Coverslips were mounted in mounting media (Dako) and imaged on an Olympus laser-scanning confocal microscope equipped with a 100× (1.35 NA oil) objective. Co-localization was measured in ImarisColoc using Costes auto-thresholds to create an overlap channel, from which overlapping voxel counts, % region of interest (ROI) overlap, and Manders’ coefficients (M1 and M2) were obtained for each cell.

### Calcium imaging

EPHA2 WT or 5A inducible cells transfected with mitoGCaMP6 were exposed to 0.5 µg/mL Doxycycline for 24 h. Cells were treated with 250 μM histamine and imaged every 2 s using a confocal microscope with a UPLSAPO 20× (0.75 NA) objective. Images were analyzed using Cellsense software. Average fluorescence intensities were background-corrected. ΔF was calculated as (F - F_0_), where F is peak intensity and F_0_ is the average of 5 pre-stimulation frames used for normalization.

### Statistical analysis

Quantitative data are presented as mean ± SD. Data analysis was performed without blinding. Sample sizes varied depending on the experimental context. Statistical analyses were performed using GraphPad Prism. Two-tailed unpaired *t*-tests were used to compare two independent groups. One-way or two-way ANOVA followed by Tukey’s post hoc test was used for comparisons among more than two groups. Exact *P* values are indicated above the bars in each quantification plot.

## Supplementary information

Peer Review File

Source data Fig. 1

Source data Fig. 2

Source data Fig. 3

Source data Fig. 4

Source data Fig. 5

Source data Fig. 6

Source data Fig. 7

Source data Fig. 8

Expanded View Figures

## Data Availability

The mass spectrometry proteomics data reported in this study have been deposited in the ProteomeXchange Consortium via the jPOST partner repository under accession code “PXD079834” for ProteomeXchange and “JPST004710” for jPOST. Other data are available from the corresponding author on request. The source data of this paper are collected in the following database record: biostudies:S-SCDT-10_1038-S44319-026-00858-6.
