# Supplementary material for: Ligand-independent EPHA2 signaling sustains neural progenitors via ECSIT and NAD+
Source: EMBO Rep. 2026 Jul 10;27(16):4782–823. doi: 10.1038/s44319-026-00858-6 (PMC13503776; doi:10.1038/s44319-026-00858-6)
Supplement: Supplementary file 1 [file 44319_2026_858_MOESM10_ESM.pdf]

Expanded View Figures

Figure EV1. Additional validation of ligand-independent EPHA2 signaling in embryonic NSPCs.

(**A**) Immunoblot validation of shRNA-mediated knockdown of endogenous mouse *Epha2* in Neuro-2a cells. (**B**) Schematic of EPHA2 domain structure and mutants used to selectively disrupt ligand-dependent (K646R and D739N) or ligand-independent (5A) signaling, with corresponding phospho-Ser897 EPHA2 and phospho-tyrosine immunoblots after expression in HEK293T cells. LBD ligand-binding domain, EGF epidermal growth factor-like, FN1 and FN2 fibronectin type III, JxM juxtamembrane segment, KD kinase domain, SAM sterile alpha motif, PDZ PDZ-binding motif. (**C**–**H**) IUE at E13.5 with EGFP-empty vector (control), EGFP-shRNA targeting mouse *Epha2* (KD EPHA2) together with either RFP-empty vector, EPHA2 WT, EPHA2 5A, EPHA2 K646R, or EPHA2 D739N. Analysis at E15.5. (**C**) GFP RFP (green) and Ki67 (red) triple IF. Scale bar, 50 µm. (**D**) Quantification of Ki67-positive electroporated cells (Control $n = 10$, KD EPHA2 $n = 11$, KD EPHA2 + EPHA2 WT $n = 13$, KD EPHA2 + EPHA2 5A $n = 13$, KD EPHA2 + EPHA2 K646R $n = 6$, KD EPHA2 + EPHA2 D739N $n = 6$ embryos). (**E**) GFP RFP (green) and SOX2 (magenta) triple IF. Scale bar, 50 µm. (**F**) Quantification of SOX2-positive electroporated cells (Control $n = 7$, KD EPHA2 $n = 6$, KD EPHA2 + EPHA2 WT $n = 9$, KD EPHA2 + EPHA2 5A $n = 8$, KD EPHA2 + EPHA2 K646R $n = 6$, KD EPHA2 + EPHA2 D739N $n = 6$ embryos). (**G**) GFP RFP (green) and TBR2 (red) triple IF. Scale bar, 50 µm. (**H**) Quantification of TBR2-positive electroporated cells (Control $n = 7$, KD EPHA2 $n = 9$, KD EPHA2 + EPHA2 WT $n = 10$, KD EPHA2 + EPHA2 5A $n = 8$ embryos). Statistical analysis was performed using one-way ANOVA followed by Tukey's post hoc test (**D**, **F**, **H**). Quantitative data were presented as mean ± SD. Exact *P* values are indicated above the bars in each quantification plot.

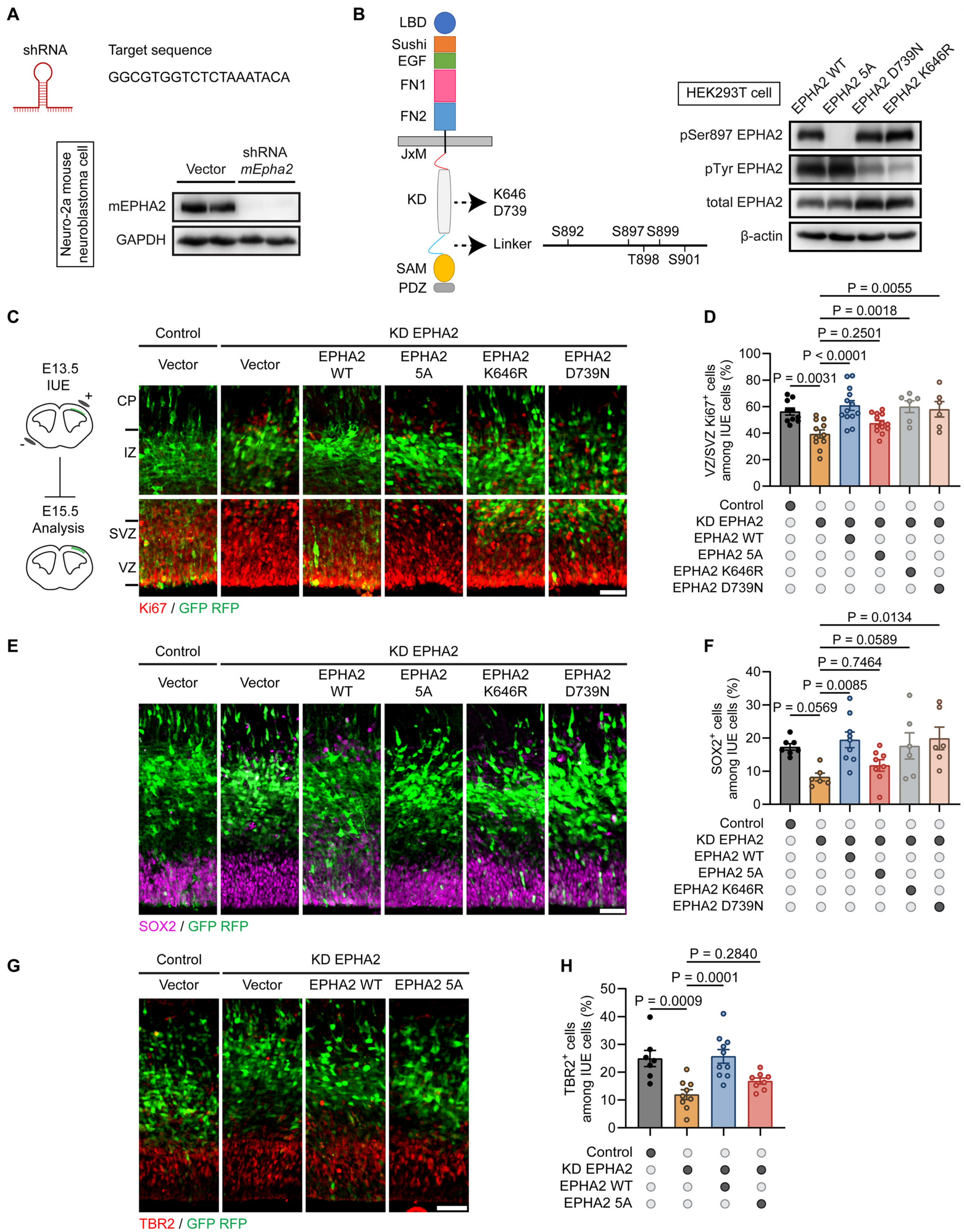
A
shRNA
Target sequence
GGCGTGGTCTCTAAATACA
Neuro-2a mouse neuroblastoma cell
Vector
shRNA mEpha2
mEPHA2
GAPDH
B
LBD
Sushi
EGF
FN1
FN2
JxM
KD
K646
D739
Linker
SAM
PDZ
S892
S897 S899
T898 S901
HEK293T cell
EPHA2 WT
EPHA2 5A
EPHA2 D739N
EPHA2 K646R
pSer897 EPHA2
pTyr EPHA2
total EPHA2
β-actin
C
E13.5 IUE
E15.5 Analysis
Control
KD EPHA2
Vector
EPHA2 WT
EPHA2 5A
EPHA2 K646R
EPHA2 D739N
CP
IZ
SVZ
VZ
Ki67 / GFP RFP
D
VZ/SVZ Ki67+ cells among IUE cells (%)
P = 0.0055
P = 0.0018
P = 0.2501
P < 0.0001
P = 0.0031
E
SOX2 / GFP RFP
F
SOX2+ cells among IUE cells (%)
P = 0.0134
P = 0.0589
P = 0.7464
P = 0.0085
P = 0.0569
G
EPHA2 WT
EPHA2 5A
TBR2 / GFP RFP
H
TBR2+ cells among IUE cells (%)
P = 0.2840
P = 0.0001
P = 0.0009

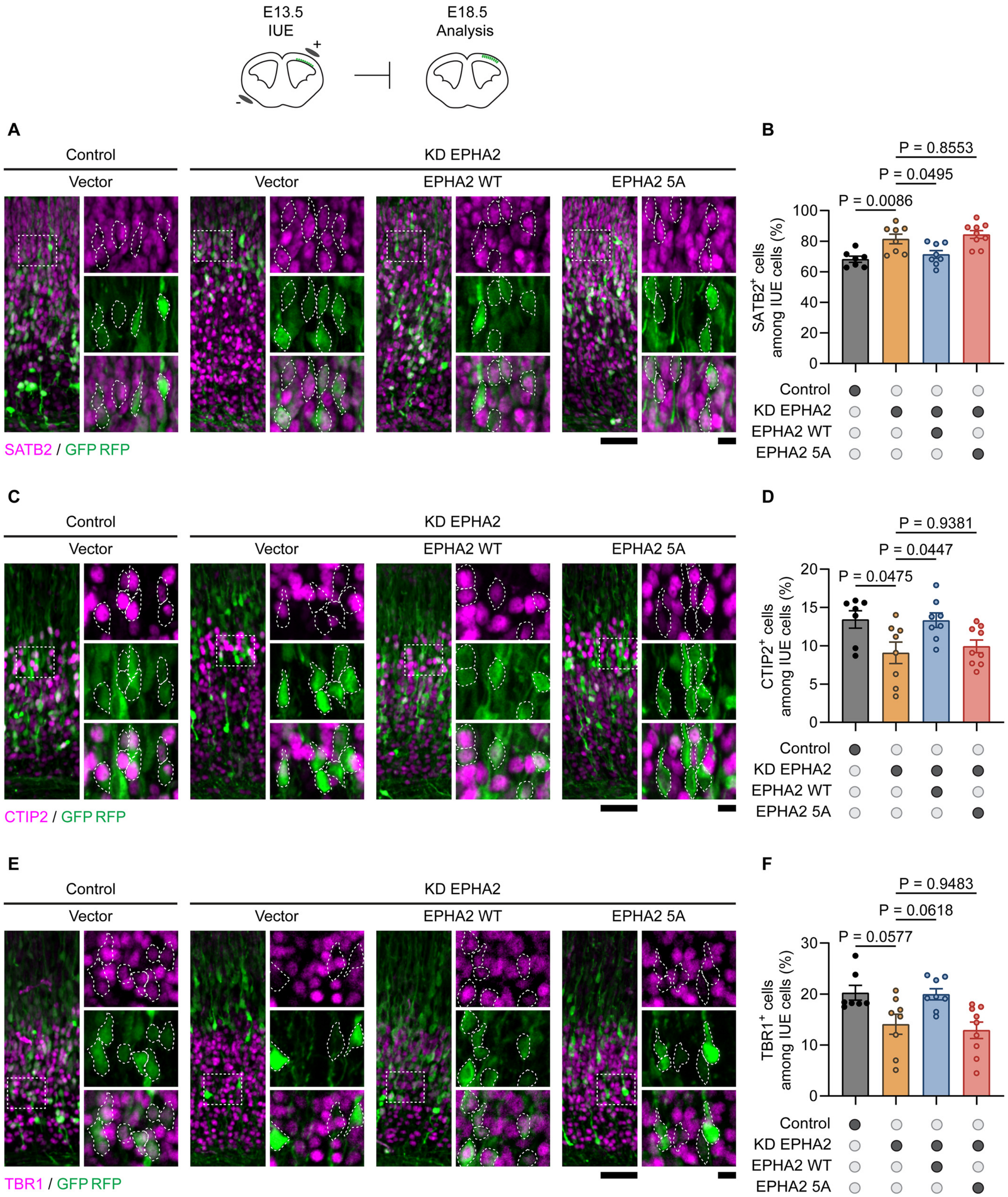
E13.5
IUE
E18.5
Analysis
A
Control
KD EPHA2
Vector
Vector
EPHA2 WT
EPHA2 5A
SATB2 / GFP RFP
B
P = 0.8553
P = 0.0495
P = 0.0086
SATB2+ cells among IUE cells (%)
Control
KD EPHA2
EPHA2 WT
EPHA2 5A
C
Control
KD EPHA2
Vector
Vector
EPHA2 WT
EPHA2 5A
CTIP2 / GFP RFP
D
P = 0.9381
P = 0.0447
P = 0.0475
CTIP2+ cells among IUE cells (%)
Control
KD EPHA2
EPHA2 WT
EPHA2 5A
E
Control
KD EPHA2
Vector
Vector
EPHA2 WT
EPHA2 5A
TBR1 / GFP RFP
F
P = 0.9483
P = 0.0618
P = 0.0577
TBR1+ cells among IUE cells (%)
Control
KD EPHA2
EPHA2 WT
EPHA2 5A

◀ **Figure EV2. Ligand-independent EPHA2 disruption alters cortical projection neuron composition.**

IUE at E13.5 with EGFP-empty vector (control), EGFP-KD EPHA2 together with either RFP-empty vector, EPHA2 WT, or EPHA2 5A. Analysis at E18.5. (**A**) GFP RFP (green) and SATB2 (magenta) triple IF. Boxed areas denote zoomed areas. Dashed lines encircle electroporated cells that are SATB2-positive or SATB2-negative. Scale bars, 50 µm (left), 10 µm (right). (**B**) Quantification of SATB2-positive electroporated cells (Control $n = 7$, KD EPHA2 $n = 8$, KD EPHA2 + EPHA2 WT $n = 8$, KD EPHA2 + EPHA2 5A $n = 9$ embryos). (**C**) GFP RFP (green) and CTIP2 (magenta) triple IF. Boxed areas denote zoomed areas. Dashed lines encircle electroporated cells that are CTIP2-positive or CTIP2-negative. Scale bars, 50 µm (left), 10 µm (right). (**D**) Quantification of CTIP2-positive electroporated cells (Control $n = 7$, KD EPHA2 $n = 8$, KD EPHA2 + EPHA2 WT $n = 8$, KD EPHA2 + EPHA2 5A $n = 9$ embryos). (**E**) GFP RFP (green) and TBR1 (magenta) triple IF. Boxed areas denote zoomed areas. Dashed lines encircle electroporated cells that are TBR1-positive or TBR1-negative. Scale bars, 50 µm (left), 10 µm (right). (**F**) Quantification of TBR1-positive electroporated cells (Control $n = 7$, KD EPHA2 $n = 8$, KD EPHA2 + EPHA2 WT $n = 8$, KD EPHA2 + EPHA2 5A $n = 9$ embryos). Statistical analysis was performed using one-way ANOVA followed by Tukey's post hoc test (**B**, **D**, **F**). Quantitative data were presented as mean ± SD. Exact *P* values are indicated above the bars in each quantification plot.

 © The Author(s)

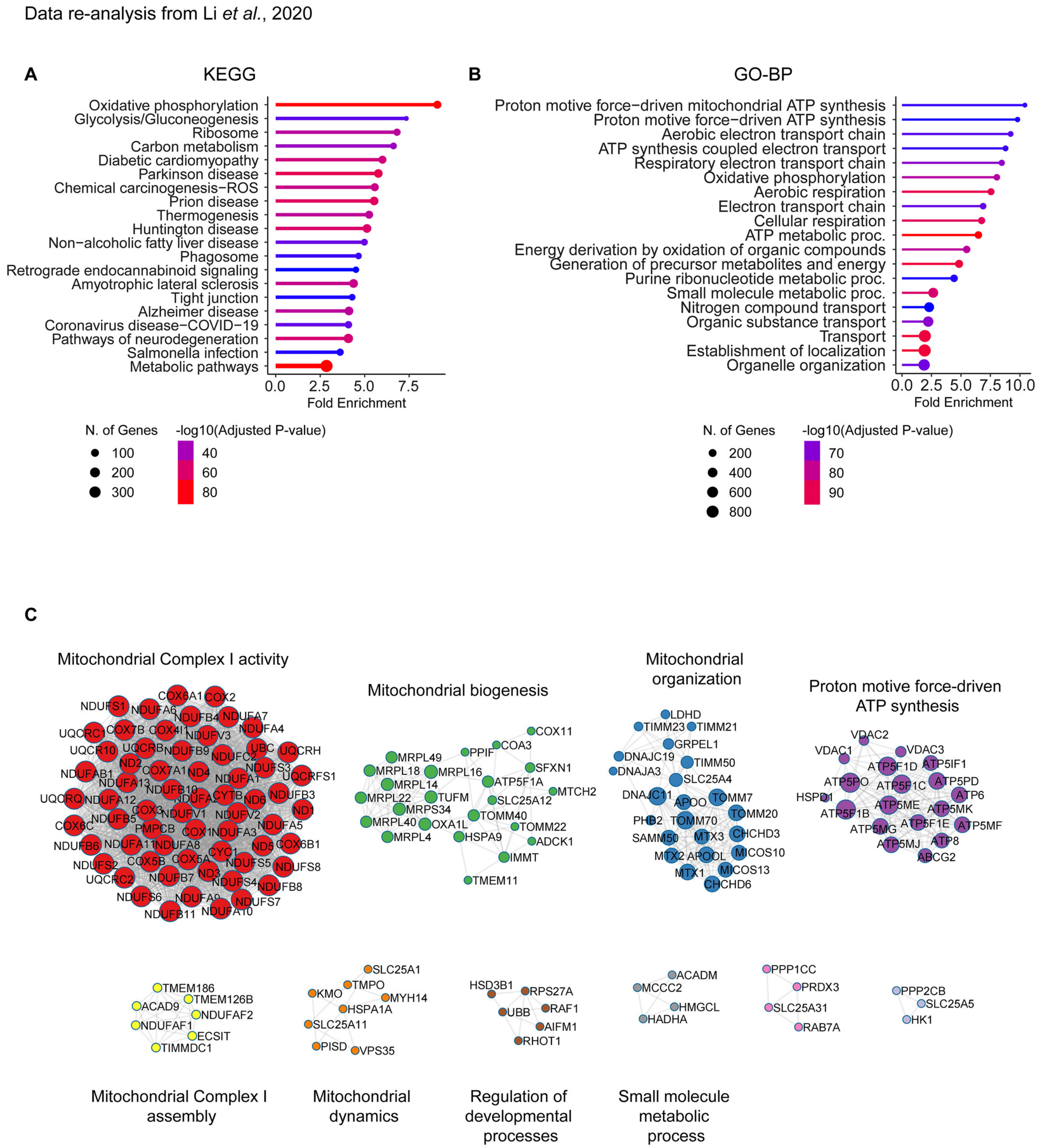

Figure EV3. Re-analysis of the EPHA2-associated proteome highlights mitochondrial pathways.

(**A–C**) EPHA2-associated proteome from non-cancerous mouse kidney tissue. Dataset from Li et al, 2020, was refined to include only proteins conserved in *Homo sapiens*, yielding 1757 interaction partners. (**A**) Kyoto Encyclopedia of Genes and Genomes (KEGG) pathway enrichment analysis of the EPHA2-associated proteome derived from non-cancerous mouse tissue. (**B**) Gene Ontology Biological Process (GO-BP) enrichment analysis of the same dataset. (**C**) Metascape protein interaction network of 321 mitochondrial proteins associated with EPHA2, highlighting modules linked to respiratory function and mitochondrial organization.

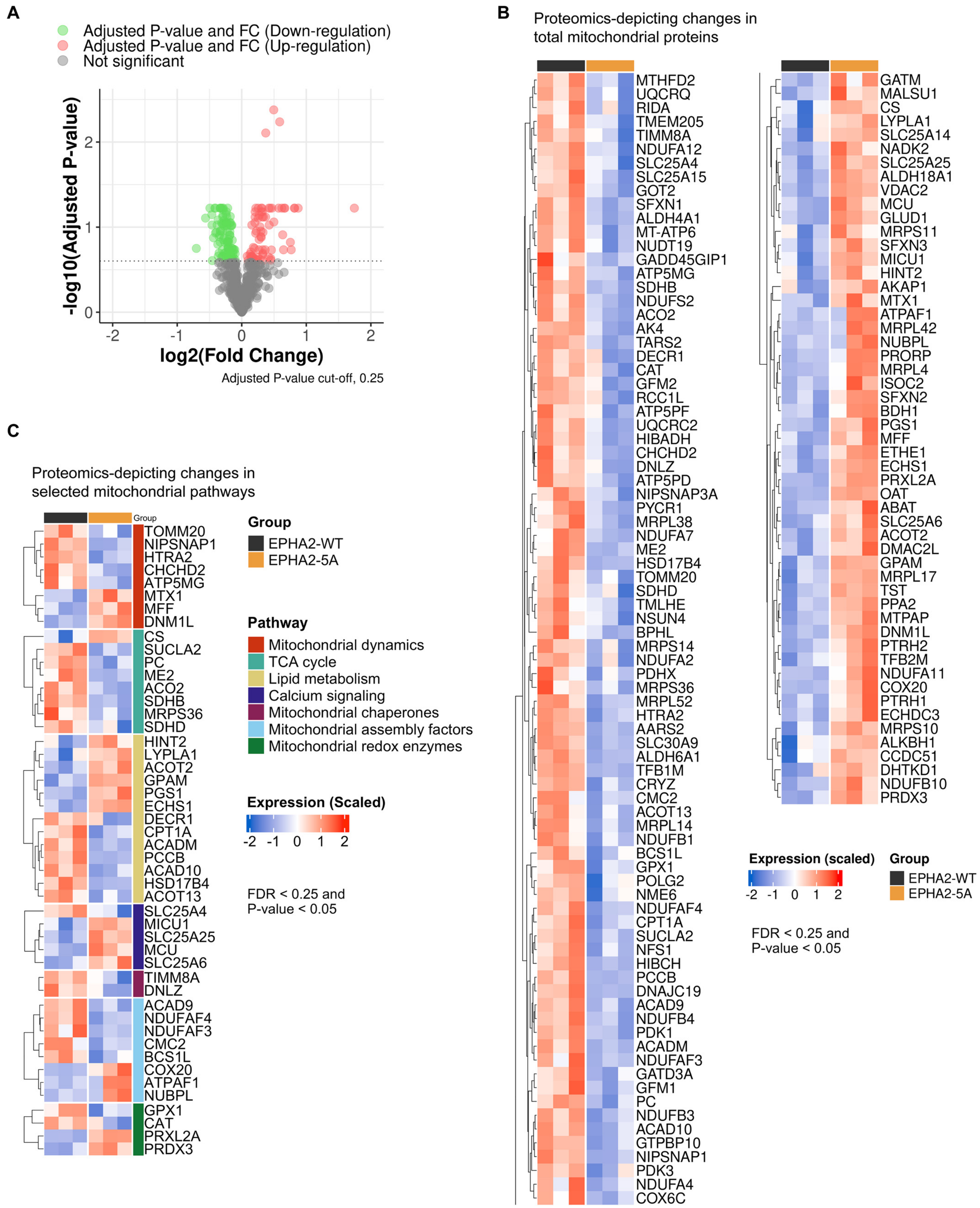
A
Adjusted P-value and FC (Down-regulation)
Adjusted P-value and FC (Up-regulation)
Not significant
-log10(Adjusted P-value)
0
1
2
-2
-1
0
1
2
log2(Fold Change)
Adjusted P-value cut-off, 0.25
B
Proteomics-depicting changes in total mitochondrial proteins
MTHFD2
UQCRQ
RIDA
TMEM205
TIMM8A
NDUFA12
SLC25A4
SLC25A15
GOT2
SFXN1
ALDH4A1
MT-ATP6
NUDT19
GADD45GIP1
ATP5MG
SDHB
NDUFS2
ACO2
AK4
TARS2
DECR1
CAT
GFM2
RCC1L
ATP5PF
UQCRC2
HIBADH
CHCHD2
DNLZ
ATP5PD
NIPSNAP3A
PYCR1
MRPL38
NDUFA7
ME2
HSD17B4
TOMM20
SDHD
TMLHE
NSUN4
BPHL
MRPS14
NDUFA2
PDHX
MRPS36
MRPL52
HTRA2
AARS2
SLC30A9
ALDH6A1
TFB1M
CRYZ
CMC2
ACOT13
MRPL14
NDUFB1
BCS1L
GPX1
POLG2
NME6
NDUFAF4
CPT1A
SUCLA2
NFS1
HIBCH
PCCB
DNAJC19
ACAD9
NDUFB4
PDK1
ACADM
NDUFAF3
GATD3A
GFM1
PC
NDUFB3
ACAD10
GTPBP10
NIPSNAP1
PDK3
NDUFA4
COX6C
GATM
MALSU1
CS
LYPLA1
SLC25A14
NADK2
SLC25A25
ALDH18A1
VDAC2
MCU
GLUD1
MRPS11
SFXN3
MICU1
HINT2
AKAP1
MTX1
ATPAF1
MRPL42
NUBPL
PRORP
MRPL4
ISOC2
SFXN2
BDH1
PGS1
MFF
ETHE1
ECHS1
PRXL2A
OAT
ABAT
SLC25A6
ACOT2
DMAC2L
GPAM
MRPL17
TST
PPA2
MTPAP
DNM1L
PTRH2
TFB2M
NDUFA11
COX20
PTRH1
ECHDC3
MRPS10
ALKBH1
CCDC51
DHTKD1
NDUFB10
PRDX3
Expression (scaled)
-2 -1 0 1 2
Group
EPHA2-WT
EPHA2-5A
FDR < 0.25 and
P-value < 0.05
C
Proteomics-depicting changes in selected mitochondrial pathways
Group
TOMM20
NIPSNAP1
HTRA2
CHCHD2
ATP5MG
MTX1
MFF
DNM1L
CS
SUCLA2
PC
ME2
ACO2
SDHB
MRPS36
SDHD
HINT2
LYPLA1
ACOT2
GPAM
PGS1
ECHS1
DECR1
CPT1A
ACADM
PCCB
ACAD10
HSD17B4
ACOT13
SLC25A4
MICU1
SLC25A25
MCU
SLC25A6
TIMM8A
DNLZ
ACAD9
NDUFAF4
NDUFAF3
CMC2
BCS1L
COX20
ATPAF1
NUBPL
GPX1
CAT
PRXL2A
PRDX3
Group
EPHA2-WT
EPHA2-5A
Pathway
Mitochondrial dynamics
TCA cycle
Lipid metabolism
Calcium signaling
Mitochondrial chaperones
Mitochondrial assembly factors
Mitochondrial redox enzymes
Expression (Scaled)
-2 -1 0 1 2
FDR < 0.25 and
P-value < 0.05

 © The Author(s)

Figure EV4. Expanded mitochondrial proteome analysis following disruption of ligand-independent EPHA2 signaling.

(**A**) Volcano plot of differentially abundant mitochondrial proteins in inducible EPHA2 WT and EPHA2 5A cells ($n = 3$ replicates per condition). (**B**) Heatmap of significantly altered mitochondrial proteins. (**C**) Analysis of selected mitochondrial pathways revealed that mitochondrial dynamics, TCA cycle, and calcium signaling were also sensitive to disruption of ligand-independent EPHA2 signaling. Statistical analysis was performed using limma-based moderated *t*-test (**A**–**C**). Proteins shown met the significance thresholds used in the proteomic analysis. Exact thresholds are indicated in the figure.

© The Author(s)

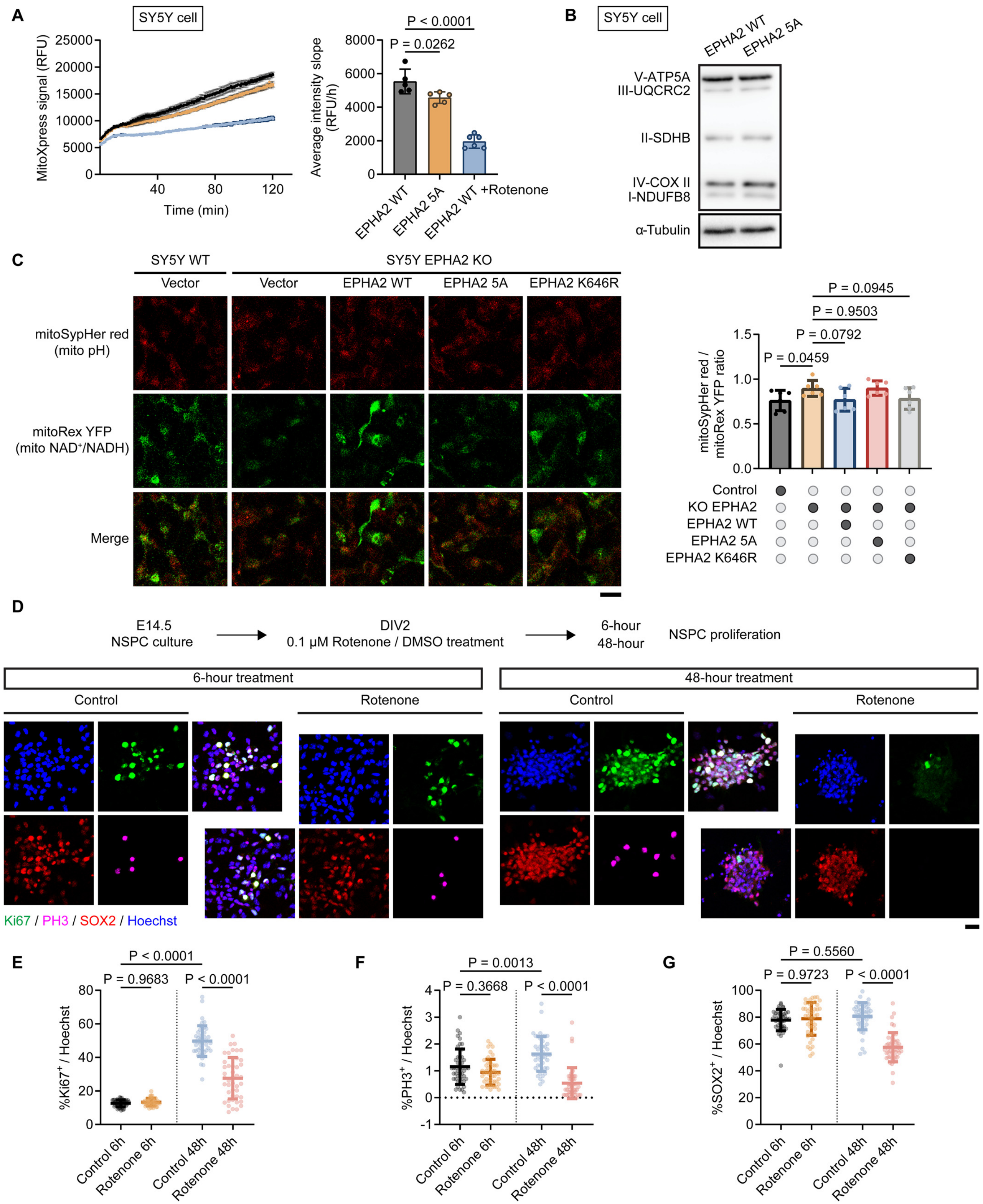
A
SY5Y cell
MitoXpress signal (RFU)
Time (min)
Average intensity slope (RFU/h)
P < 0.0001
P = 0.0262
EPHA2 WT
EPHA2 5A
EPHA2 WT +Rotenone
B
SY5Y cell
EPHA2 WT
EPHA2 5A
V-ATP5A
III-UQCRC2
II-SDHB
IV-COX II
I-NDUFB8
α-Tubulin
C
SY5Y WT
SY5Y EPHA2 KO
Vector
Vector
EPHA2 WT
EPHA2 5A
EPHA2 K646R
mitoSypHer red (mito pH)
mitoRex YFP (mito NAD+/NADH)
Merge
mitoSypHer red / mitoRex YFP ratio
P = 0.0945
P = 0.9503
P = 0.0792
P = 0.0459
Control
KO EPHA2
EPHA2 WT
EPHA2 5A
EPHA2 K646R
D
E14.5 NSPC culture
DIV2 0.1 μM Rotenone / DMSO treatment
6-hour 48-hour
NSPC proliferation
6-hour treatment
48-hour treatment
Control
Rotenone
Control
Rotenone
Ki67 / PH3 / SOX2 / Hoechst
E
%Ki67+ / Hoechst
P < 0.0001
P = 0.9683
P < 0.0001
Control 6h
Rotenone 6h
Control 48h
Rotenone 48h
F
%PH3+ / Hoechst
P = 0.0013
P = 0.3668
P < 0.0001
Control 6h
Rotenone 6h
Control 48h
Rotenone 48h
G
%SOX2+ / Hoechst
P = 0.5560
P = 0.9723
P < 0.0001
Control 6h
Rotenone 6h
Control 48h
Rotenone 48h

 © The Author(s)

Figure EV5. Pharmacological Complex I inhibition phenocopies the progenitor defect caused by EPHA2 disruption.

(**A**) MitoXpress assay showing reduced oxygen consumption in inducible EPHA2 5A cells. Rotenone at 1 μM used as assay control (EPHA2 WT $n = 5$, EPHA2 5A $n = 5$, Rotenone $n = 6$ replicates). RFU/h, relative fluorescence units per hour. (**B**) Immunoblot analysis of total OXPHOS complex subunits in inducible EPHA2 WT and EPHA2 5A cells. (**C**) Ratiometric mitoRexYFP and mitoSypHer red imaging showing rescue of the mitochondrial redox phenotype by EPHA2 WT and EPHA2 K646R, but not by EPHA2 5A ($n = 6$ replicates per condition). Scale bar, 30 μm. (**D**) Representative images of primary NSPCs treated with vehicle or low-dose Rotenone and stained for proliferation markers. For each condition, 45 randomly selected microscopic fields were acquired. Scale bar, 20 μm. (**E**) Quantification of Ki67-positive NSPCs after Rotenone treatment ($n = 45$ per condition). (**F**) Quantification of PH3-positive NSPCs after Rotenone treatment ($n = 45$ per condition). (**G**) Quantification of SOX2-positive NSPCs after Rotenone treatment ($n = 45$ per condition). Statistical analysis was performed using one-way ANOVA followed by Tukey's post hoc test (**A**, **C**, **E**–**G**). Quantitative data are presented as mean ± SD. Exact *P* values are indicated above the bars in each quantification plot.

© The Author(s)

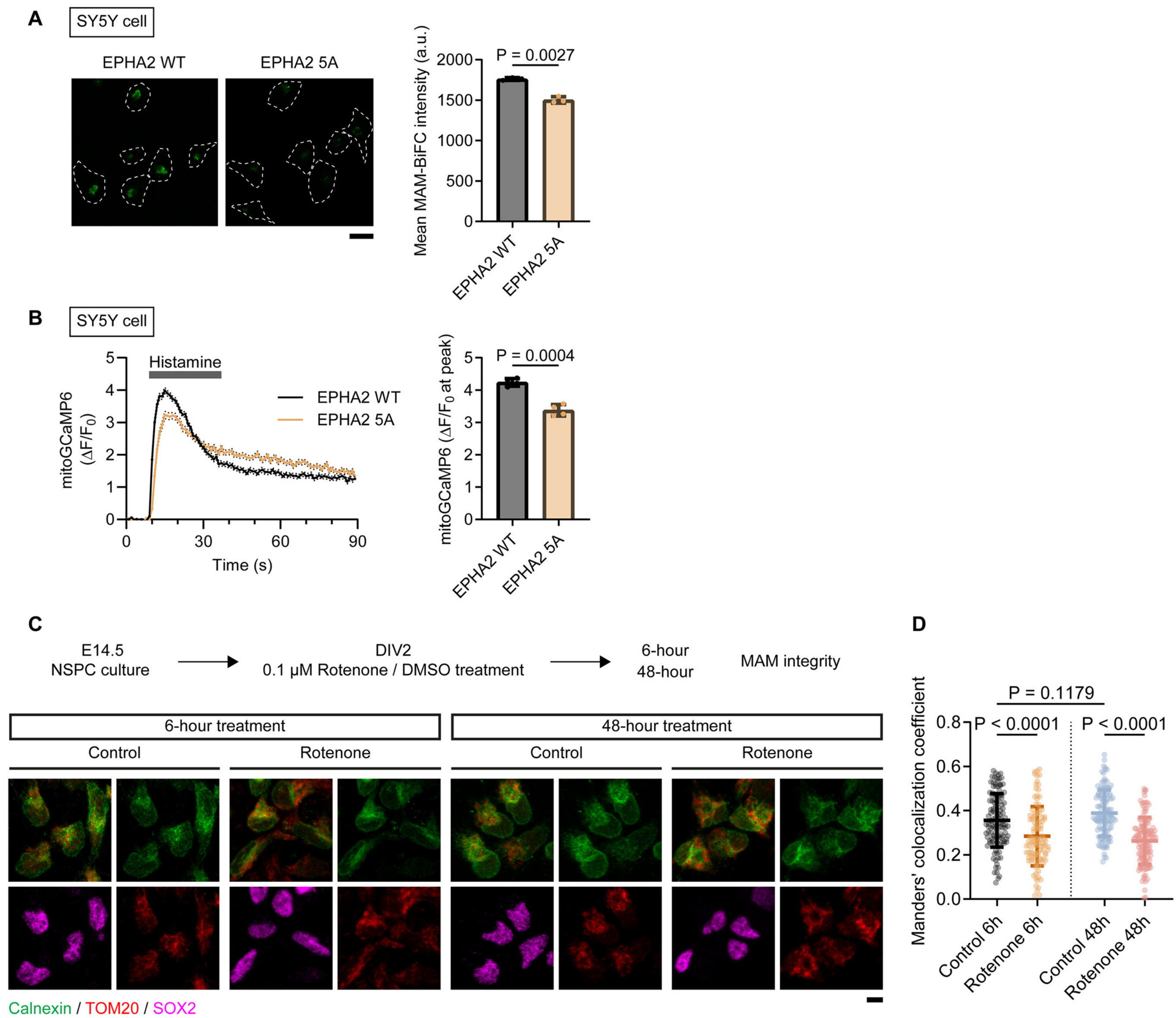

Figure EV6. Ligand-independent EPHA2 signaling regulates ER-mitochondria contact sites.

(**A**) MAM-BiFC-based analysis of ER-mitochondria contact sites (MAMs) in inducible EPHA2 WT and EPHA2 5A cells (EPHA2 WT $n = 3$ replicates, EPHA2 5A $n = 3$ replicates). Scale bar, 20 µm. (**B**) Mitochondrial calcium uptake assay measured by mitoGCaMP6 sensor following stimulation by 250 µM histamine in inducible EPHA2 WT and EPHA2 5A cells (EPHA2 WT $n = 4$ replicates, EPHA2 5A $n = 4$ replicates). (**C**) Representative images assessing ER-mitochondria contacts using ER marker Calnexin and mitochondrial marker TOM20 after Rotenone treatment in primary NSPCs. Scale bar, 5 µm. (**D**) Quantification of MAM integrity, assessed by co-localization between Calnexin and TOM20 after Rotenone treatment (Control 6 h $n = 121$, Rotenone 6 h $n = 109$, Control 48 h $n = 123$, Rotenone 48 h $n = 120$ cells). Statistical analysis was performed using unpaired *t*-test with Welch's correction (**A**, **B**) and one-way ANOVA followed by Tukey's post hoc test (**D**). Quantitative data are presented as mean ± SD. Exact *P* values are indicated above the bars in each quantification plot.

© The Author(s)

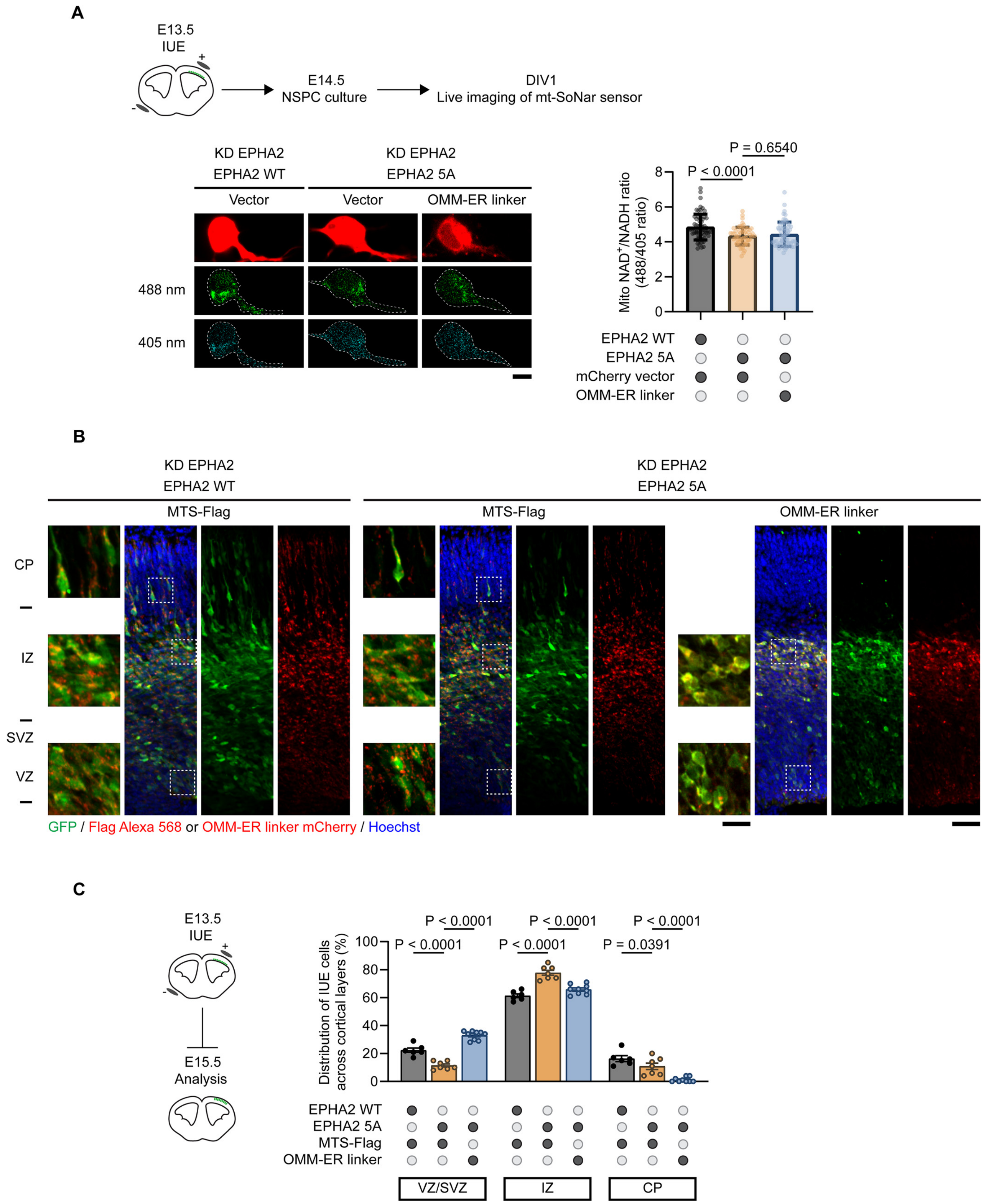
A
E13.5
IUE
E14.5
NSPC culture
DIV1
Live imaging of mt-SoNar sensor
KD EPHA2
EPHA2 WT
KD EPHA2
EPHA2 5A
Vector
Vector
OMM-ER linker
488 nm
405 nm
P = 0.6540
P < 0.0001
Mito NAD+/NADH ratio
(488/405 ratio)
EPHA2 WT
EPHA2 5A
mCherry vector
OMM-ER linker
B
KD EPHA2
EPHA2 WT
KD EPHA2
EPHA2 5A
MTS-Flag
MTS-Flag
OMM-ER linker
CP
IZ
SVZ
VZ
GFP / Flag Alexa 568 or OMM-ER linker mCherry / Hoechst
C
E13.5
IUE
E15.5
Analysis
P < 0.0001
P < 0.0001
P < 0.0001
P < 0.0001
P < 0.0001
P = 0.0391
Distribution of IUE cells
across cortical layers (%)
EPHA2 WT
EPHA2 5A
MTS-Flag
OMM-ER linker
VZ/SVZ
IZ
CP

Figure EV7. ER–mitochondria contacts regulate NSPC behavior independently of mitochondrial redox balance downstream of EPHA2.

(**A**) Mitochondrial NAD^+/NADH readout assessed by mt-SoNar sensor in primary NSPCs expressing EPHA2 WT or EPHA2 5A with or without an artificial OMM–ER linker (EPHA2 WT + Vector $n = 61$, EPHA2 5A + Vector $n = 60$, EPHA2 5A + OMM–ER linker $n = 63$ cells). Scale bar, 5 µm. (**B**, **C**) IUE at E13.5 with EGFP-KD EPHA2 + EGFP-EPHA2 WT or EGFP-KD EPHA2 + EGFP-EPHA2 5A together with either MTS-Flag or OMM–ER linker. Analysis at E15.5. (**B**) GFP RFP electroporated cells are shown relative to cortical layers defined by Hoechst density. Boxed areas denote zoomed areas. Scale bars, 20 µm (left), 50 µm (right). (**C**) Quantification of the distribution of electroporated cells across VZ/SVZ, IZ, and CP under the indicated conditions (EPHA2 WT + MTS-Flag $n = 6$, EPHA2 5A + MTS-Flag $n = 7$, EPHA2 5A + OMM–ER linker $n = 9$ embryos). Statistical analysis was performed using one-way ANOVA followed by Tukey's post hoc test (**A**) and two-way ANOVA with Šídák's multiple comparisons test (**C**). Quantitative data are presented as mean ± SD. Exact *P* values are indicated above the bars in each quantification plot.

 © The Author(s)

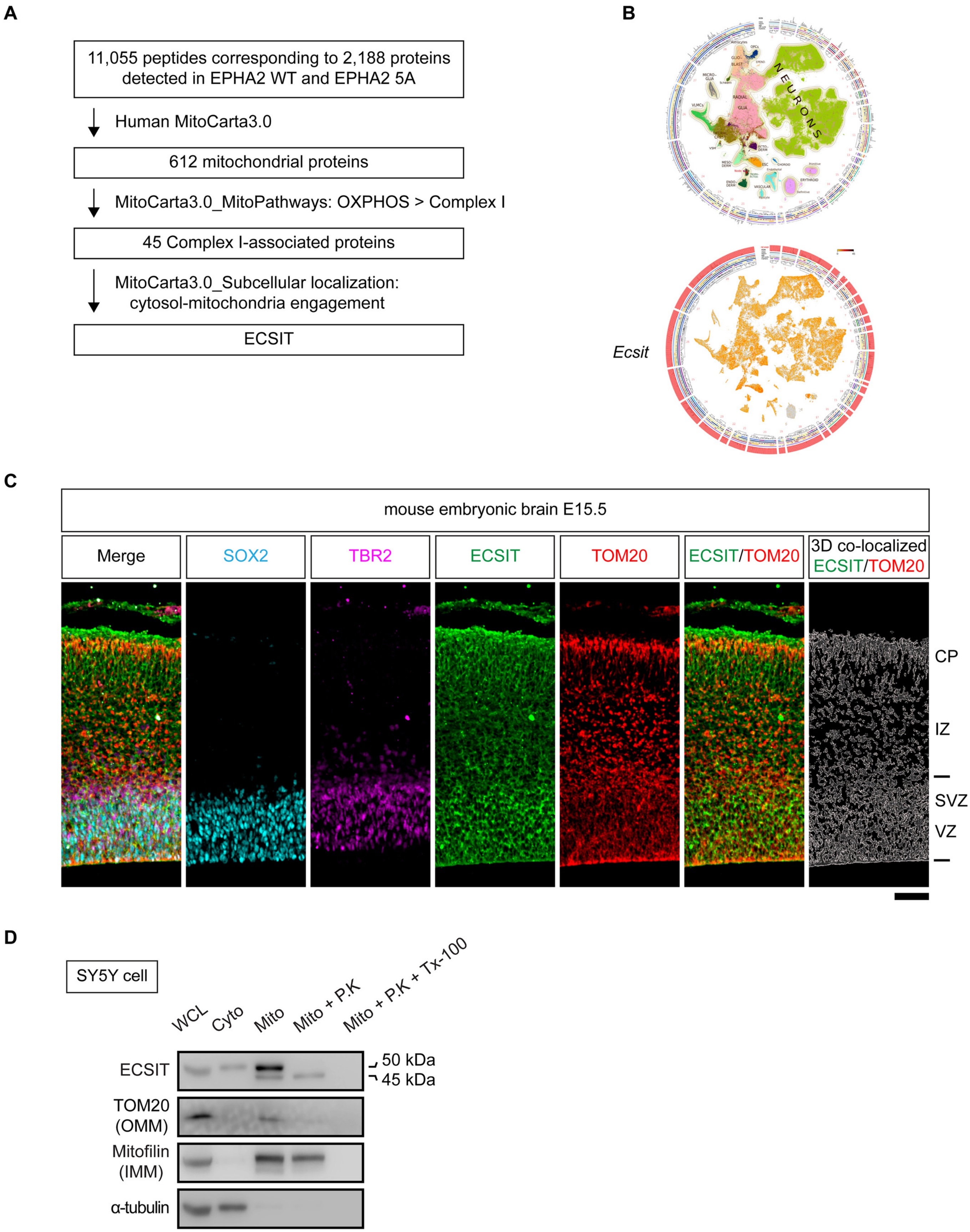
A
11,055 peptides corresponding to 2,188 proteins detected in EPHA2 WT and EPHA2 5A
Human MitoCarta3.0
612 mitochondrial proteins
MitoCarta3.0_MitoPathways: OXPHOS > Complex I
45 Complex I-associated proteins
MitoCarta3.0_Subcellular localization: cytosol-mitochondria engagement
ECSIT
B
NEURONS
Ecsit
C
mouse embryonic brain E15.5
Merge
SOX2
TBR2
ECSIT
TOM20
ECSIT/TOM20
3D co-localized ECSIT/TOM20
CP
IZ
SVZ
VZ
D
SY5Y cell
WCL
Cyto
Mito
Mito + P.K
Mito + P.K + Tx-100
ECSIT
50 kDa
45 kDa
TOM20 (OMM)
Mitofilin (IMM)
α-tubulin

Figure EV8. Layered evidence supporting ECSIT as an EPHA2-sensitive mitochondrial intermediary.

(**A**) Schematic summary of the criteria used to prioritize ECSIT as a mechanistic intermediary downstream of EPHA2. (**B**) Single-cell transcriptomic map of the developing mouse brain showing *Ecsit* expression. Dataset from La Manno et al, 2021. (**C**) Immunofluorescence of E15.5 mouse cortex stained for ECSIT and TOM20. The 3D co-localized area between ECSIT and TOM20 was generated using Imaris software. Scale bar, 50 µm. (**D**) Proteinase K protection assay showing protease protection of the processed ECSIT species in intact mitochondria. PK proteinase K, Tx-100 Triton X-100.

 © The Author(s)

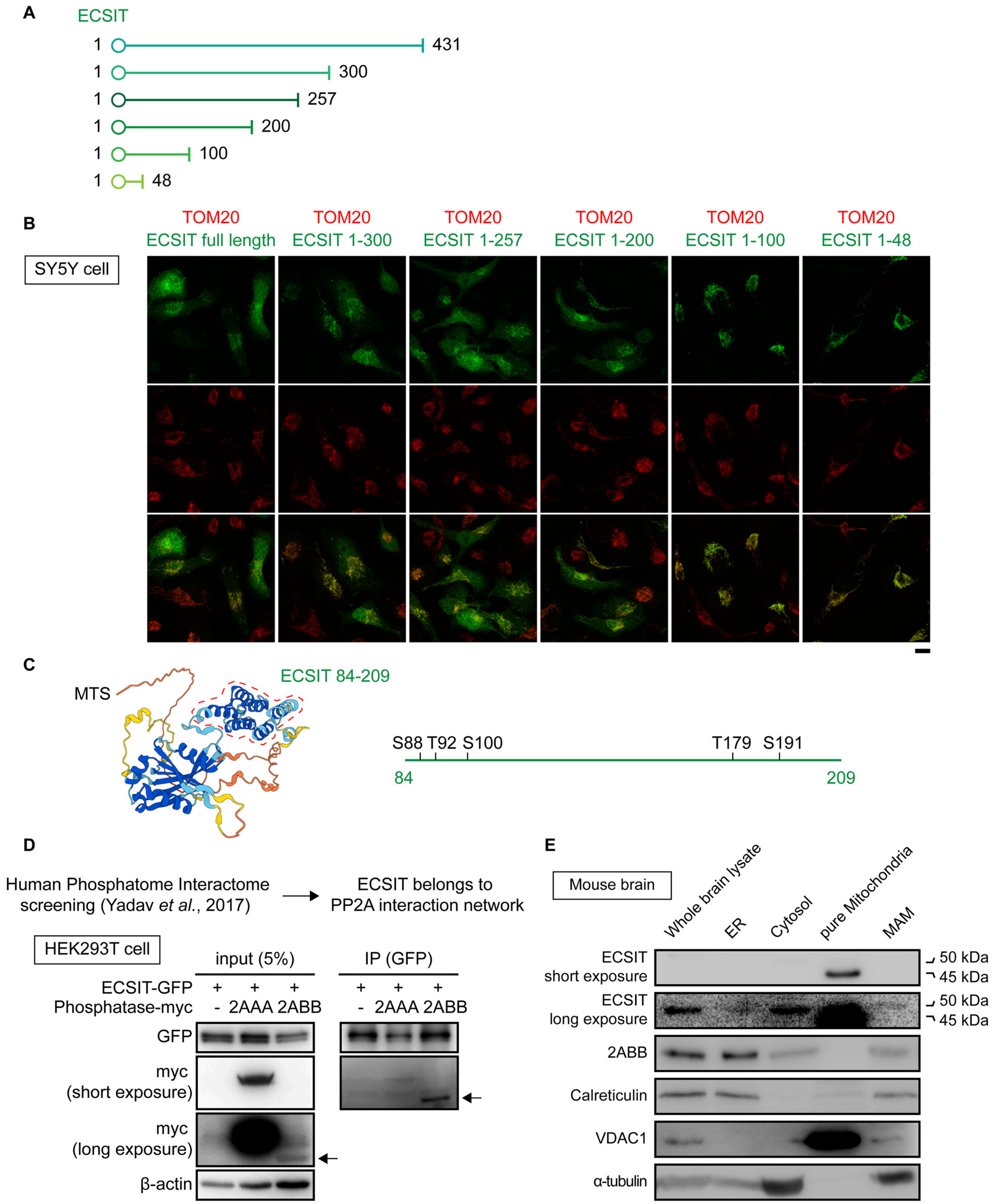
A
ECSIT
1 431
1 300
1 257
1 200
1 100
1 48
B
TOM20 ECSIT full length
TOM20 ECSIT 1-300
TOM20 ECSIT 1-257
TOM20 ECSIT 1-200
TOM20 ECSIT 1-100
TOM20 ECSIT 1-48
SY5Y cell
C
MTS
ECSIT 84-209
S88 T92 S100 T179 S191
84 209
D
Human Phosphatome Interactome screening (Yadav et al., 2017)
ECSIT belongs to PP2A interaction network
HEK293T cell
input (5%)
IP (GFP)
ECSIT-GFP + + + + + +
Phosphatase-myc - 2AAA 2ABB - 2AAA 2ABB
GFP
myc (short exposure)
myc (long exposure)
β-actin
E
Mouse brain
Whole brain lysate
ER
Cytosol
pure Mitochondria
MAM
ECSIT short exposure
ECSIT long exposure
50 kDa
45 kDa
2ABB
Calreticulin
VDAC1
α-tubulin

Figure EV9. Mapping the ECSIT region and phosphosite that regulate mitochondrial accumulation.

(**A**) Schematic of ECSIT truncation constructs used to define the region controlling mitochondrial localization. (**B**) Representative images of SH-SY5Y cells expressing full-length or truncated ECSIT-GFP constructs with TOM20 staining. Scale bar, 10 µm. (**C**) AlphaFold-based structural model of ECSIT highlighting a cluster of alpha helices spanning amino acids 84–209 that contains candidate serine and threonine residues. (**D**) Immunoprecipitation showing association between ECSIT and the PP2A regulatory subunit PPP2R2B/2ABB in HEK293T cells. (**E**) Subcellular fractionation of mouse brain lysates showing ECSIT and PPP2R2B/2ABB distribution across ER, cytosol, pure mitochondria, and MAM fractions.

 © The Author(s)
