# Supplementary material for: Ligand-independent EPHA2 signaling sustains neural progenitors via ECSIT and NAD+
Source: EMBO Rep. 2026 Jul 10;27(16):4782–823. doi: 10.1038/s44319-026-00858-6 (PMC13503776; doi:10.1038/s44319-026-00858-6)
Supplement: Supplementary file 2 [file 44319_2026_858_MOESM1_ESM.pdf]

EMBO reports

Ligand-independent EPHA2 signaling sustains neural progenitors via ECSIT and NAD+

Sang Ki Park, Tran Diem Nghi, Truong My Nhung, Seunghyun Kim, Jonghyeok Yun, Nguyen Tran Nam Tien, Ara Lee, Minkyo Jung, Eun Jin Jang, Bon Seong Goo, Jin Yeong Yoo, Sung Ryong Shin, Ji-Young Mun, Kyeng Min Park, Nguyen Phuoc Long, and Cana Park

Corresponding author(s): Sang Ki Park (skpark@postech.ac.kr)

Review Timeline:		
	Transfer Date:	7th May 26
	Editorial Decision:	11th Jun 26
	Revision Received:	19th Jun 26
	Accepted:	23rd Jun 26

Editor: Esther Schnapp

Transaction Report: A revised version of this manuscript was transferred to EMBO reports following peer review at the EMBO Journal.

(Note: With the exception of the correction of typographical or spelling errors that could be a source of ambiguity, letters and reports are not edited. Depending on transfer agreements, referee reports obtained elsewhere may or may not be included in this compilation. Referee reports are anonymous unless the Referee chooses to sign their reports.)

Referee #1:

In this manuscript by Nghi and colleagues, the authors reveal a role for non-canonical EPHA2 signaling in controlling mitochondrial metabolism in neural progenitor cells, which in turn impacts their proliferation and/or self-renewal. They use in utero electroporation and lipofection in cell lines to lower down EPHA2 levels and rescue the effects with either WT or mutant forms of EPHA2. Since most significant changes upon EPHA2 knockdown are related to mitochondrial metabolism, they further explore the link between non-canonical EPHA2 and mitochondria. Their experiments show that EPH2A controls mitochondrial function promoting the mitochondrial localization of ECSIT, a factor essential for the correct assembly of complex I. The authors also show that restoration of NAD+ levels is sufficient to rescue the defects caused by loss of non-canonical EPHA2 signaling and that the phosphatase PP2A is a mediator (although not a direct one) of the effects of EPHA2 on ECSIT. In short, the manuscript shows a relevant example of how plasma membrane signals can regulate mitochondrial metabolism, which is an interesting and relevant finding for many fields. However, in order to fully support their important conclusions, some additional controls and clarifications are necessary:
-The effects of EPHA2 during cortical development remain largely inconclusive from their experiments. While there is some evidence that NSPCs lose their self-renewal capacity, this is never directly measured. The authors should quantify the % of electroporated cells in each layer (VZ, SVZ, IZ, CP), as it is routinely done for IUE studies. If there is indeed increased differentiation (and therefore increased migration out of the VZ/SVZ), this would be clearly seen with such quantification. This quantification would also make evident the proposed accumulation of cells in the IZ due to their inability to exit the cell cycle. Similarly, proliferation rates within NSPCs are not measured (only respect to the total population of electroporated cells). To quantify NSPC proliferation, the authors should count the % of proliferating NSPCs out of those still remaining in the VZ/SVZ.
-BrdU experiments in 2F/G and EV2A-D should be better explained in the text.
-In general, it is not clear from the main text or M&M how the co-localizations made by immunohistochemistry are transformed into a cell % (e.g. in EV2).
-Throughout the manuscript, cells (or even microscopic fields) are considered ns (e.g. Figure 3). This can lead to significance in statistical tests, even when the differences are very small and not relevant. The number of independent ns performed for each experiment should be indicated in the figure legends, and for each n, all cells should be averaged. In the specific case of figure 3C the data should be shown as an average for each of the 12 samples, assuming each sample is one n (please also explain what independent samples mean).

-In figure EV3 the control and EPHA2 KD conditions are missing, without which it is hard to interpret the results.
-In the proteomics experiment, there is a clear outlier in the WT samples (seen in the PCA in EV6A). Could the authors test whether that sample should be excluded (and if so, re-analyze the data)? Also in the proteomics data, the statistical significance of the changes shown in Figure 4B/C and EV6C is not shown, please add this information.
-Citations are missing for several statements throughout the text, please revise. Just as two examples: in line 2, add the citation that shows that/which other family members of EPH family are associated with brain development; and in lines85/87, add the references for well-characterized mutational sites on EPHA2 and for Ser897 being important for non-canonical signaling.
-In figure 5, to fully prove that mitochondrial metabolism is controlled by ligand-independent EPHA2 signaling, it should be shown that kinase-dead mutants, unlike 5A, can rescue the mitochondrial defects.

Other minor points:
-In the introduction, lines 5-6: it would be clear if the shift from symmetric self-renewal to asymmetric self-renewal/differentiation was mentioned. As it is, the text seems to suggest RG do not self-renew anymore.
-Through the paper experiments are done in either HEK293T or SH-SY5Y lines, please state in the text and figures clearly when each cell type is used.
-The authors make a strong point dismissing cancer contexts as not able to replicate physiological conditions (line 122). However, they mostly use the glioblastoma line SH-SY5Y for their experiments, the implication of which should be discussed.
- In Figure 1F, the SOX2 staining did not work, as it does not appear nuclear. Please revise. In the same figure, it is interesting that EPHA2 does not seem to be only in the apical membrane, as the authors stated in the introduction. A comment on this would be clarifying.
-In some places in the text, such as in figure 5, it is unclear if the authors are still using the KD of EPHA2, please revise and state it clearly (I assume yes).

Referee #2:

In this manuscript, Diem Nghi and colleagues report that the mitochondria complex I assembly factor ECSIT is controlled by noncanonical EPHA2 signaling to maintain the

renewal of neural stem and progenitor cells (NSPCs). They suggest that ligand-independent EPHA2 signaling activates PP2A-mediated dephosphorylation of ECSIT, which in turn allows ECSIT to enter mitochondria and assemble complex I. This enables NSPCs to proliferate due to their ability to generate NAD+.

This is a data heavy manuscript, and the authors have undertaken a huge amount of work, and in many cases, with significant quantification.

While the figures were well constructed, the manuscript was challenging to read and interpret the results given the brief nature of the written material and the short figure legends. The lack of label for each figure also made it less easy to review. In my review, I will focus on the mitochondrial aspects of the manuscript. In summary, I do not believe that the authors are able to demonstrate a specific complex I defect, not a specific loss of ECSIT targeting to mitochondria that affects assembly and NAD levels.

In Fig. 3, the effects on mitochondrial function following expression of EPHA2 5A are quite subtle given the suggestion that complex I activity is perturbed. I am concerned about the cell based imaging approaches used to assess mitochondrial function and this needs to be strengthened. In 3B, membrane potential effects would be more strongly reduced with a complex I depletion. A Seahorse analysis is the norm for looking at this. In 3C, there is no rationale for decreased ROS in 5A cells. The suggestion of a disrupted NAD+/NADH ratio in 5A cells (Fig. EV5E) was performed by looking at a mitoSypHer/mitoRexYFP ratio. This is a critical element of the manuscript and the range is really variable across the cells so it is unclear if there is a strong effect. It would be better tested using an enzymatic approach.

The proteomic approach shows incredible variability in OXPHOS subunit levels in EPHA2-5A expressing cells - and not specific to complex I. For example, in Fig 4C, complex V subunit (MT-ATP6), two complex II subunits (SDHB, SDHD), a complex III subunit (UQCRQ) and a complex IV subunit (COA3) are all down while some subunits are up. This does not at all point to a specific reduction in a complex I assembly factor. In fact, loss of ECSIT leads to a specific loss of complex I subunits and virtually all of them are downregulated as they are proteolytically degraded if unassembled (see PMID: 27626371). The authors need to show whether there is an assembly defect using blue native PAGE and compare it to other complexes.
Regarding the ECSIT localisation (Fig 5), the two bands shown for ECSIT (fig 5B) does not indicate mitochondrial location. There is no doublet in the whole cell fraction yet it appears in the crude mito fraction. This is not clear and could be a degradation product. The authors would need to perform more detailed biochemical analysis here and include other

control proteins.
In Figure 6B, the authors do an IP with the PP2A regulatory subunit and enrich a "mitochondrial" ECSIT. But PP2A is in the cytosol so should not interact with a mito form. Another confounding factor is that a cytosolic form of a folded protein is unlikely to enter mitochondria after undergoing a dephosphorylation event.
It is well established that mitochondrial protein import requires proteins to be unfolded to enter mitochondria (they are imported post translation but before folding can occur). Suggesting that PP2A dephosphorylates Ecsit such that it can then be targeted to mitochondria, unfold and then refold in the matrix would be an unusual finding.
The ECSIT-GFP constructs in Fig Ev8 are also unclear. The authors are biased towards high expressing GFP expression and it is clear that the low expressing ECSIT forms are targeted into cells with colocalization with TOM20.

Referee #3:

This manuscript reports the discovery of a ligand-independent EPHA2 signaling axis that maintains neural stem and progenitor cell (NSPC) self-renewal through mitochondrial Complex I-dependent NAD^+ regeneration. The authors demonstrate that EPHA2, enriched in radial glia and intermediate progenitors, operates independently of ephrin ligands to sustain proliferation by preventing premature cell cycle exit. Mechanistically, ligand-free EPHA2 promotes PP2A-mediated dephosphorylation of ECSIT at threonine 179, enabling its mitochondrial translocation, Complex I integration, and NAD^+ regeneration. Disruption of this pathway reduces Complex I activity, alters mitochondrial morphology, and impairs progenitor proliferation, which can be rescued by maternal NAD^+ supplementation.
This study uncovers a novel mechanistic link between membrane receptor signaling and mitochondrial metabolism in developing neural progenitors, addressing a long-standing gap in how extracellular cues regulate mitochondrial function during corticogenesis.
With that, there are numerous things to address to strengthen this manuscript and the major conclusions.

-The model shows that EPHA2 activation promotes PP2A-mediated dephosphorylation of ECSIT, yet the data remain correlative. Evidence of direct interaction or activation between EPHA2 and PP2A would strengthen causality. Alternatively, determining whether PP2A activity toward ECSIT is lost upon EPHA2 inhibition would address this gap.

-The NAD^+ supplementation experiment is compelling but broad in interpretation. The

conclusion that NAD^+ acts via Complex I-dependent regeneration could be overstated. NAD^+ also affects PARPs, sirtuins, and nucleotide biosynthesis, which may contribute to the observed effects. It would be important to clarify whether rescue occurs via mitochondrial NAD^+/NADH redox balance or more global NAD^+-dependent processes. Including direct measurements of NAD^+/NADH in vivo, or partial rescue with NADH-insensitive analogs, could help delineate specificity. Also, testing whether mitochondrially targeted NAD^+ precursors (e.g., NMN vs. NR) produce differential rescue outcomes would clarify whether the effect is indeed mitochondrial.

-The authors convincingly show decreased Complex I activity and oxygen consumption in EPHA2 5A cells, but these data stop short of demonstrating that Complex I-mediated NAD^+ regeneration is the causal determinant of NSPC proliferation. It would be important to assess whether pharmacological inhibition of Complex I (ex. rotenone) phenocopies EPHA2 loss in NSPCs and whether alternative NAD^+-regenerating pathways (e.g., NDI1 expression) can rescue proliferation. Measurements of total cellular and mitochondrial NAD^+/NADH ratios would strengthen the claim beyond sensor-based imaging.

-The EM images show "fragmented and swollen mitochondria," but quantitative morphometric analysis is needed to confirm these qualitative observations. Inclusion of mitophagy markers (e.g., PINK1, LC3-II, p62) would clarify whether mitochondrial turnover is altered.

-The reduction in MAM-BiFC signal and Ca^{2+} uptake is intriguing, yet the causal link between MAM disruption and progenitor proliferation remains speculative. Are these changes secondary to Complex I dysfunction, or do they occur upstream? Functional validation using artificial tether constructs (e.g., synthetic MAM linker expression) could reveal whether restoring ER-mitochondrial contacts rescues metabolic or proliferative defects.

-The data suggest a mild OXPHOS impairment, but embryonic NSPCs are generally glycolytic. It would strengthen the metabolic argument to measure glycolytic flux (ECAR) and ATP source partitioning (glycolysis vs. OXPHOS).

-The proteomic dataset is a valuable resource, yet the focus is largely on Complex I subunits. Additional analysis of mitochondrial chaperones, assembly factors, and redox enzymes could reveal whether a broader reorganization of the mitochondrial proteome occurs.

-SH-SY5Y cells are a convenient system but metabolically distinct from primary NSPCs. Studies must be confirmed in primary NSPCs.

Point-by-Point Response

We express our sincere appreciation to the three reviewers for their rigorous and constructive evaluations, as well as for their insightful recommendations, and we have attempted to address virtually all of the experimental issues they raised. We also avoided potential overinterpretation regarding PP2A action on mature mitochondrial ECSIT or a defined PP2A-driven import mechanism. Instead, the data now support a more precise conclusion: a PP2A-sensitive ECSIT T179 phospho-state regulates mitochondrial ECSIT accumulation downstream of ligand-independent EPHA2 signaling, upstream of Complex I function and progenitor maintenance.

To summarize the significant additions and modifications,

1. We quantified the fraction of electroporated cells across cortical layers under the compared conditions throughout the manuscript to clarify the effects of EPHA2 signaling in Fig. 2A-B, Fig. 4A-B, Fig. 4E-F, and Fig. EV9B-C.
2. We updated the *in vivo* analysis to quantify progenitor maintenance directly within the VZ/SVZ in Fig. 2C-F.
3. We added Seahorse analyses in Fig. 3E-H and direct Complex I enzymatic assays in both cell line and embryonic brain tissue in Fig. 3I-J, which now establish impaired mitochondrial respiration and reduced Complex I-dependent NADH oxidation when ligand-independent EPHA2 signaling is disrupted. Accordingly, the original Fig. 3 was moved to Expanded View Fig. EV5, presented as supportive evidence of perturbed mitochondrial homeostasis.
4. In Fig. 4A-C, we compared the differential rescue effects of MTS-*Lb*NOX, which selectively enhances mitochondrial NAD^+ regeneration without perturbing ETC flux, and Ndi1, which regenerates NAD^+ and donates electrons to the CoQ pool of the ETC, ultimately contributing to ATP synthesis.
5. We further confirmed the importance of mitochondrial redox balance by adding treatment with the mitochondrially targeted NAD^+ precursor NMN in Fig. 4E-F.
6. We strengthened the ECSIT arm of the study through compartment-resolved fractionation in Fig. 5B and proteinase K protection assay of the processed mitochondrial species in Fig. EV10D.
7. The effects on progenitor proliferation following pharmacological inhibition of Complex I by low-dose rotenone were added in Fig. EV7D-G.
8. The relationship between disruption of ER-mitochondria contacts, Complex I dysfunction, and progenitor maintenance was provided in Fig. EV8 and Fig. EV9.
9. The mitochondrial proteomics data were re-analyzed after assessment of potential outlier samples and now report changes not only in ETC subunits but also in other mitochondrial pathways. The updated data are shown in Fig. 3B-D and Fig. EV6A-C.
10. We elaborated and modified the text and figures wherever necessary for clarity and adequate presentation of the data.

Point-by-point responses are as follows. For terminological consistency, figures from the originally submitted manuscript are designated as “**original Fig.**”, whereas those from the revised manuscript are denoted as “**revised Fig.**”.

Referee #1:

In this manuscript by Nghi and colleagues, the authors reveal a role for non-canonical EPHA2 signaling in controlling mitochondrial metabolism in neural prsogenitor cells, which in turn impacts their proliferation and/or self-renewal. They use in utero electroporation and lipofection in cell lines to lower down EPHA2 levels and rescue the effects with either WT or mutant forms of EPHA2. Since most significant changes upon EPHA2 knockdown are related to mitochondrial metabolism, they further explore the link between non-canonical EPHA2 and mitochondria. Their experiments show that EPH2A controls mitochondrial function promoting the mitochondrial localization of ECSIT, a factor essential for the correct assembly of complex I. The authors also show that restoration of NAD+ levels is sufficient to rescue the defects caused by loss of non-canonical EPHA2 signaling and that the phosphatase PP2A is a mediator (although not a direct one) of the effects of EPHA2 on ECSIT. In short, the manuscript shows a relevant example of how plasma membrane signals can regulate mitochondrial metabolism, which is an interesting and relevant finding for many fields. However, in order to fully support their important conclusions, some additional controls and clarifications are necessary:

1) The effects of EPHA2 during cortical development remain largely inconclusive from their experiments. While there is some evidence that NSPCs lose their self-renewal capacity, this is never directly measured. The authors should quantify the % of electroporated cells in each layer (VZ, SVZ, IZ, CP), as it is routinely done for IUE studies. If there is indeed increased differentiation (and therefore increased migration out of the VZ/SVZ), this would be clearly seen with such quantification. This quantification would also make evident the proposed accumulation of cells in the IZ due to their inability to exit the cell cycle.

Similarly, proliferation rates within NSPCs are not measured (only respect to the total population of electroporated cells). To quantify NSPC proliferation, the authors should count the % of proliferating NSPCs out of those still remaining in the VZ/SVZ.

Thank you for this helpful suggestion. In **original Fig. 4H**, we quantified and compared the fraction of electroporated cells across cortical layers (VZ/SVZ, IZ, and CP) under EPHA2 WT and 5A conditions, revealing enhanced displacement out of the VZ/SVZ and accumulation within the IZ in EPHA2 5A, consistent with Reviewer #1’s observation. We also agree that these data would be more appropriately positioned earlier in the manuscript to enhance clarity. Accordingly, we have incorporated these quantitative analyses into **revised Fig. 2A, B**, where the distribution of electroporated cells across cortical layers is compared among control, EPHA2 knockdown, and rescue conditions with EPHA2 WT, 5A, and kinase-dead mutants (K646R and D739N).

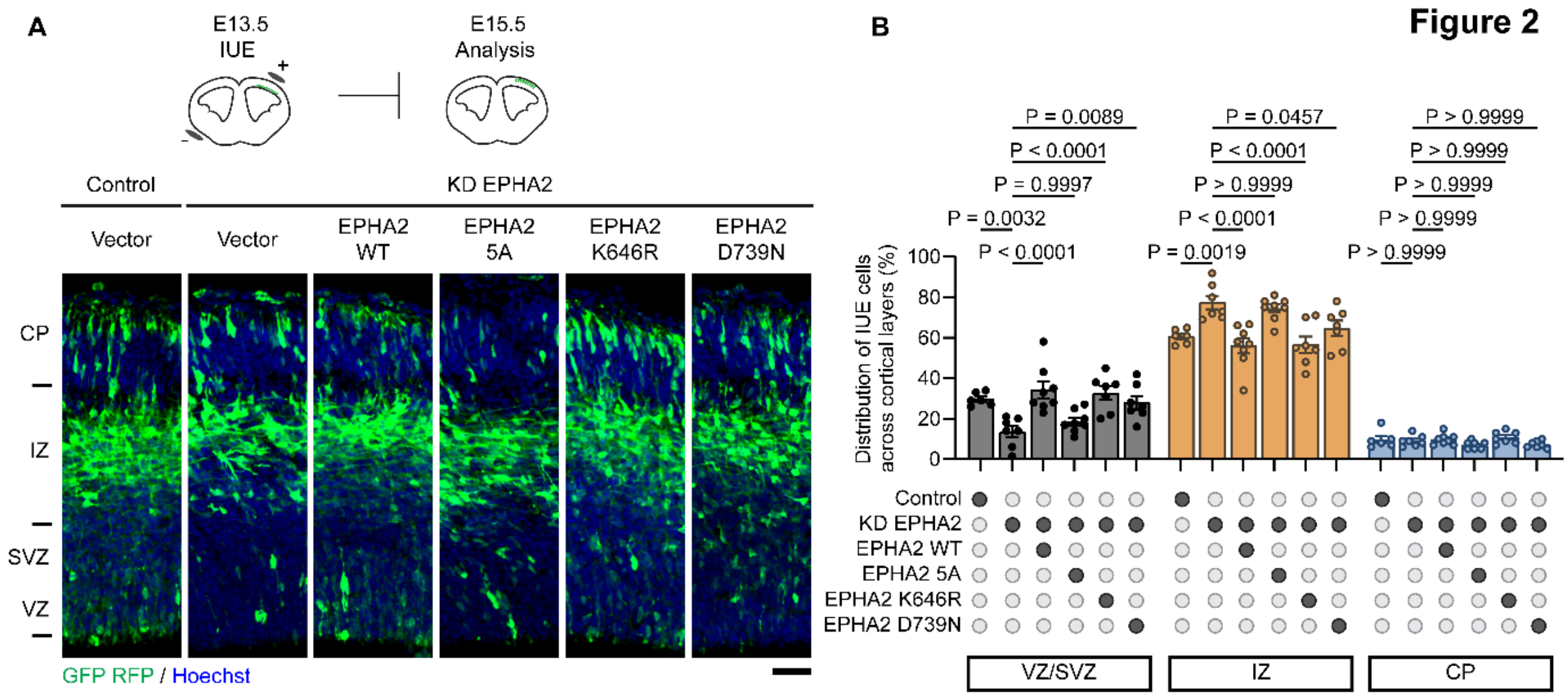

Figure 2

IUE at E13.5 with EGFP-empty vector (control), EGFP-shRNA targeting mouse *Epha2* (KD EPHA2) together with either RFP-empty vector, EPHA2 WT, EPHA2 5A, EPHA2 K646R, or EPHA2 D739N. Analysis at E15.5.

(A) GFP RFP electroporated cells are shown relative to cortical layers defined by Hoechst density. Scale bar, 50 µm.

(B) Quantification of the distribution of electroporated cells across VZ/SVZ, IZ, and CP under the indicated conditions, showing rescue by EPHA2 WT and kinase-dead mutants, but not by EPHA2 5A.

Furthermore, **original Fig. 2F–I** have been replaced with **revised Fig. 2C–F**, in which we specifically quantify proliferation within NSPCs. This was achieved by measuring the proportion of S-phase BrdU-positive (following a 2-hour pulse) and M-phase PH3-positive cells among electroporated cells localized to the VZ/SVZ, rather than across the total electroporated population, in accordance with established methodology (Naher *et al*, 2025). White dashed lines are used to highlight IUE cells that are either positive or negative for BrdU and PH3. These analyses reveal a reduction in both BrdU$^+$ and PH3$^+$ cells among IUE cells within the VZ/SVZ under EPHA2 knockdown, a phenotype that is rescued by EPHA2 WT but not by the 5A mutant.

Additionally, **original Fig. 2A–D**, which reported the proportions of Ki67$^+$ and SOX2$^+$ cells among IUE cells, have been relocated to **revised Fig. EV1C–F**. These data are now presented alongside the quantification of TBR2$^+$ cells, thereby consolidating related progenitor cell analyses and demonstrating that disruption of ligand-independent EPHA2 signaling depletes both apical and basal progenitor pools.

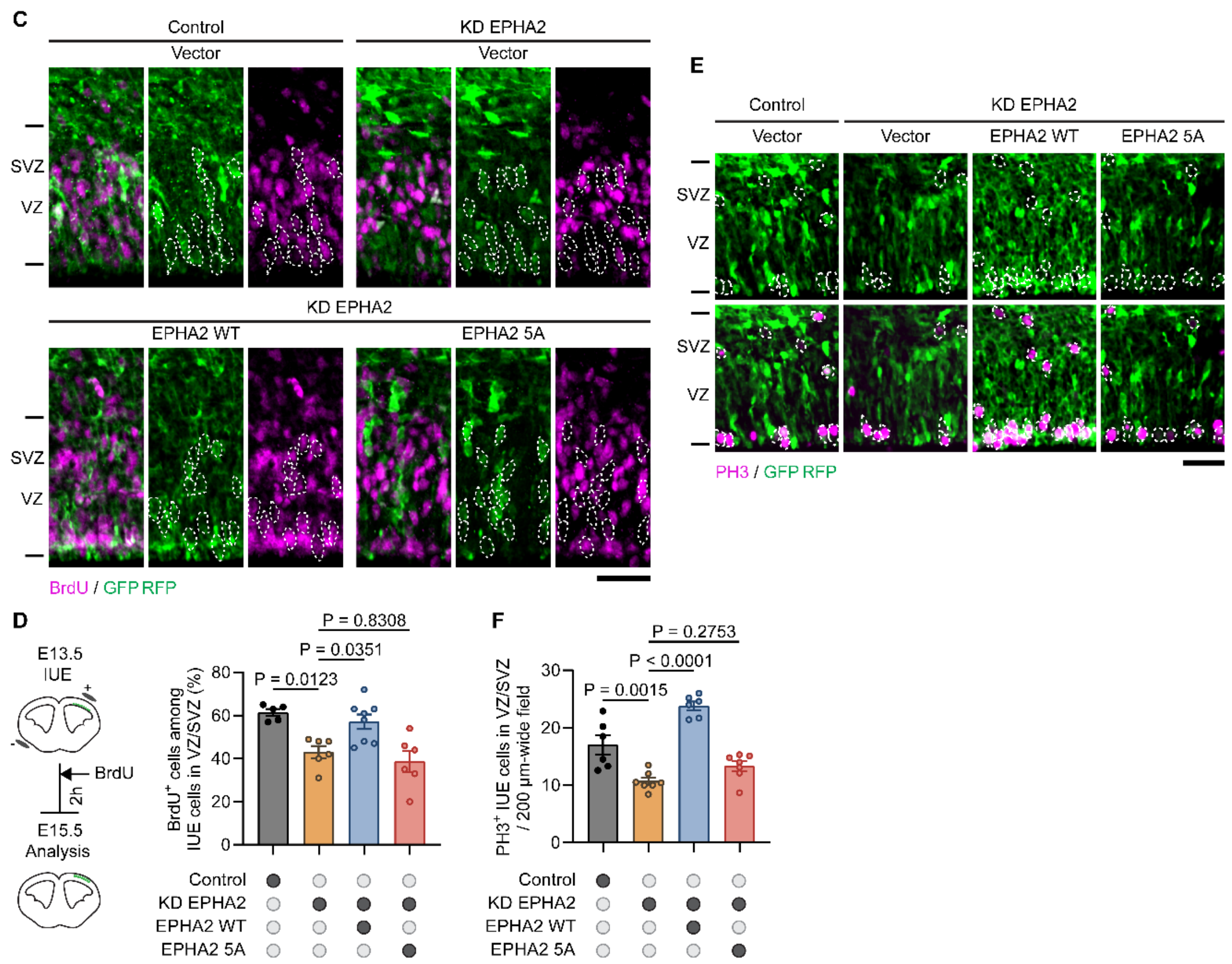

Figure 2

(C) GFP RFP (green) and BrdU (magenta) triple IF. White dashed lines highlight IUE cells that are either BrdU-positive or BrdU-negative. Scale bar, 50 µm.

(D) Quantification of S-phase BrdU-positive electroporated cells in the VZ/SVZ.

(E) GFP RFP (green) and PH3 (magenta) triple IF. White dashed lines highlight IUE cells that are either PH3-positive or PH3-negative. Scale bar, 50 µm.

(F) Quantification of mitotic PH3-positive electroporated cells in the VZ/SVZ.

2) BrdU experiments in 2F/G and EV2A-D should be better explained in the text.

Thank you for this comment. In the revised manuscript, we have expanded the Results section corresponding to **revised Fig. 2C, D** and **Fig. EV2A–D** to explicitly define the two BrdU-labeling paradigms. For **Fig. 2C, D**, we clarify that “S-phase proliferating cells were pulse-labeled with BrdU two hours prior to tissue collection at E15.5” (*page 5, lines 90-91*). For **Fig. EV2A–D**, we state that “BrdU/Ki67 co-labeling was performed to assess cell cycle exit (Fig. EV2A). BrdU injection at E14.5 labels S-phase cells during a 24-hour pulse, while Ki67 marks actively cycling cells at the time of analysis at E15.5. $Ki67^{-}$ $BrdU^{+}$ cells thus represent those that exited the cell cycle within this labeling interval” (*page 5, lines 106-109*).

3) In general, it is not clear from the main text or M&M how the co-localizations made by immunohistochemistry are transformed into a cell % (e.g. in EV2).

Thank you for this helpful comment. In the revised Methods section ("Image acquisition and analysis"), we have added that "Precisely, confocal Z-stack images were segmented in Imaris to reconstruct three-dimensional surfaces corresponding to electroporated cells (GFP/RFP) and for each marker (*e.g.*, BrdU, Ki67, SOX2), following baseline subtraction and automatic thresholding. Co-localized objects between reconstructed surfaces were identified using the ImarisColoc XTension module and counted within predefined ROIs (VZ/SVZ, IZ, and CP delineated based on Hoechst density). Quantitative values are expressed as the proportion of electroporated cells co-labeled with marker divided by the total number of electroporated cells within the same ROI and embryo. Specifically for **Fig. EV2A–D**, the percentage of $Ki67^{+}$ $BrdU^{+}GFP^{+}$ cells (representing cells that remain in the cell cycle) was first quantified within the $BrdU^{+}GFP^{+}$ population. The percentage of $Ki67^{-}BrdU^{+}GFP^{+}$ cells (representing cells that have exited the cell cycle) was subsequently calculated by subtracting this value from 100%." (*page 15, lines 526-536*).

4) Throughout the manuscript, cells (or even microscopic fields) are considered ns (e.g. Figure 3). This can lead to significance in statistical tests, even when the differences are very small and not relevant. The number of independent ns performed for each experiment should be indicated in the figure legends, and for each n, all cells should be averaged. In the specific case of figure 3C the data should be shown as an average for each of the 12 samples, assuming each sample is one n (please also explain what independent samples mean).

We thank the reviewer for highlighting this important statistical issue. We have re-analyzed all imaging-based cell-line datasets using the biological replicate, rather than individual cells or microscopic fields, as the unit of n. For *in vivo* experiments, one n corresponds to one embryo. For cell-based assays, one n corresponds to one independently prepared culture or transfection/doxycycline induction. Within each biological replicate, all cells or fields were averaged before statistical testing, and all figure legends have been updated to state the biological unit explicitly. For the **original Fig. 3C** (now **revised Fig. EV5F**) MitoSOX analysis, the original "12 samples" came from 3 independent biological experiments with 4 samples per experiment, and statistics are now performed on the 3 experiment-level means. For the EM analysis, all mitochondria were averaged within each cell before statistical testing.

5) In figure EV3 the control and EPHA2 KD conditions are missing, without which it is hard to interpret the results.

We have incorporated control and EPHA2 KD conditions into **revised Fig. EV3**, including both representative images and quantitative analyses for SATB2, CTIP2, and TBR1. We also report embryo n for each group, and revise the Results text to interpret EPHA2 5A in the context of these baselines (*page 5, lines 118-123*).

Figure EV3

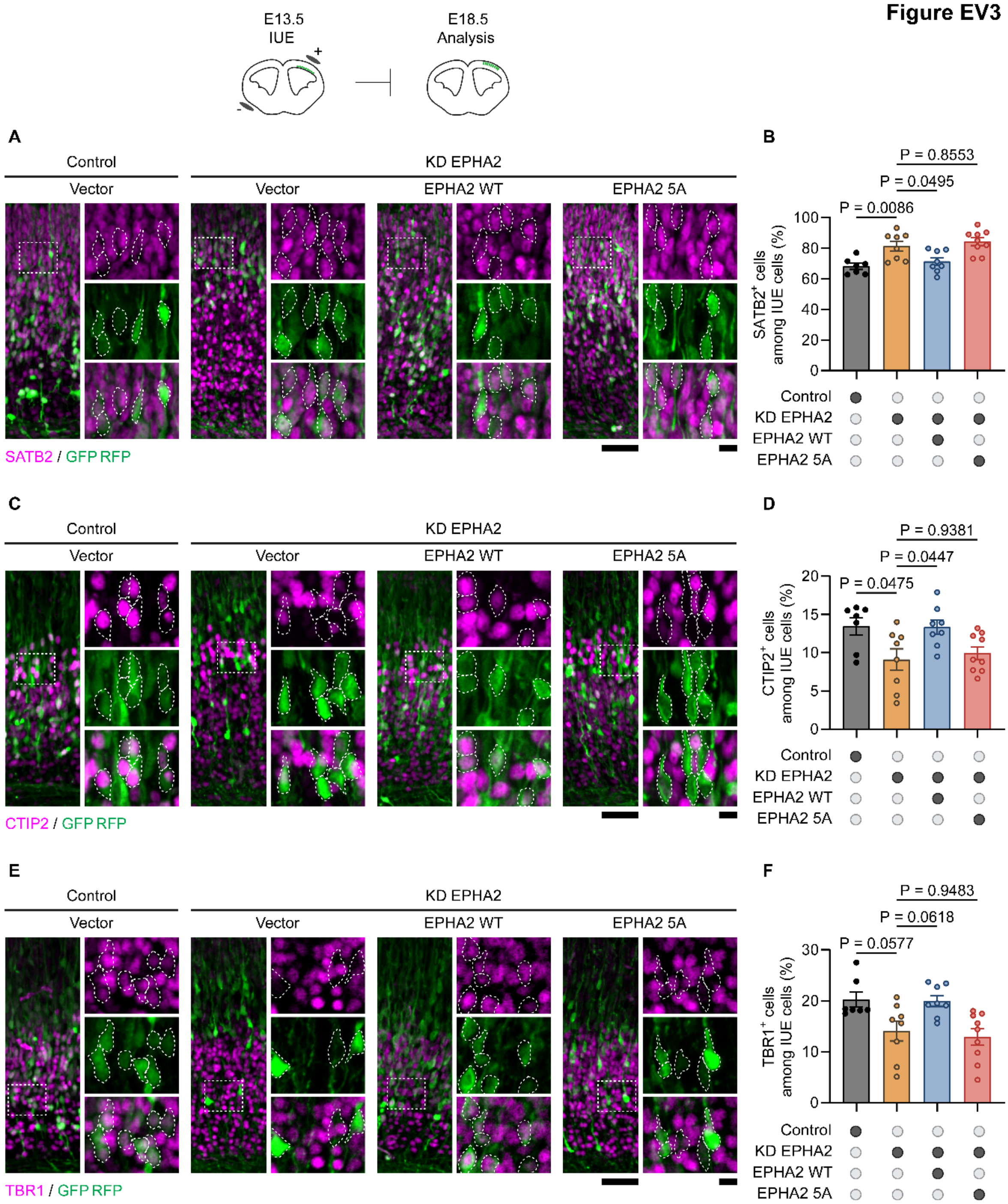

Figure EV3

IUE at E13.5 with EGFP-empty vector (control), EGFP-KD EPHA2 together with either RFP-empty vector, EPHA2 WT, or EPHA2 5A. Analysis at E18.5.

(A) GFP RFP (green) and SATB2 (magenta) triple IF. Boxed areas denote zoomed areas. Dashed lines encircle electroporated cells that are SATB2-positive or SATB2-negative. Scale bars, 50 µm (left), 10 µm (right).

(B) Quantification of SATB2-positive electroporated cells.

(C) GFP RFP (green) and CTIP2 (magenta) triple IF. Boxed areas denote zoomed areas. Dashed lines encircle electroporated cells that are CTIP2-positive or CTIP2-negative. Scale bars, 50 µm (left), 10 µm (right).

(D) Quantification of CTIP2-positive electroporated cells.

(E) GFP RFP (green) and TBR1 (magenta) triple IF. Boxed areas denote zoomed areas. Dashed lines encircle electroporated cells that are TBR1-positive or TBR1-negative. Scale bars, 50 µm (left), 10 µm (right).

(F) Quantification of TBR1-positive electroporated cells.

6) In the proteomics experiment, there is a clear outlier in the WT samples (seen in the PCA in EV6A). Could the authors test whether that sample should be excluded (and if so, re-analyze the data)? Also in the proteomics data, the statistical significance of the changes shown in Figure 4B/C and EV6C is not shown, please add this information.

We thank the reviewer for this important comment and for the opportunity to clarify the basis for our assessment of data quality. Although formal statistical approaches for outlier detection, including quantile regression, ensemble-based statistical methods such as SEAOP, and robust covariance-based approaches such as robust PCA, can in principle be considered, they are less suited to our dataset because of the small sample size and high dimensionality ($n = 4$ per group, $p = 600$; thus $n << p$). We therefore based our assessment primarily on multiple technical quality control (QC) metrics, supported by exploratory analyses such as PCA. Specifically, the sample in question (sample F5 of EPHA2 WT in **Figure 1 for reviewer**) showed increased missingness (A), weaker inter-sample correlation within the WT group (B), a shifted intensity distribution (C), and reduced identification depth relative to the other samples (D). PCA likewise showed separation of this sample from the main WT cluster (E). Together, these orthogonal QC analyses support the interpretation that F5 is technically atypical relative to the remaining samples and may indeed represent a potential outlier.

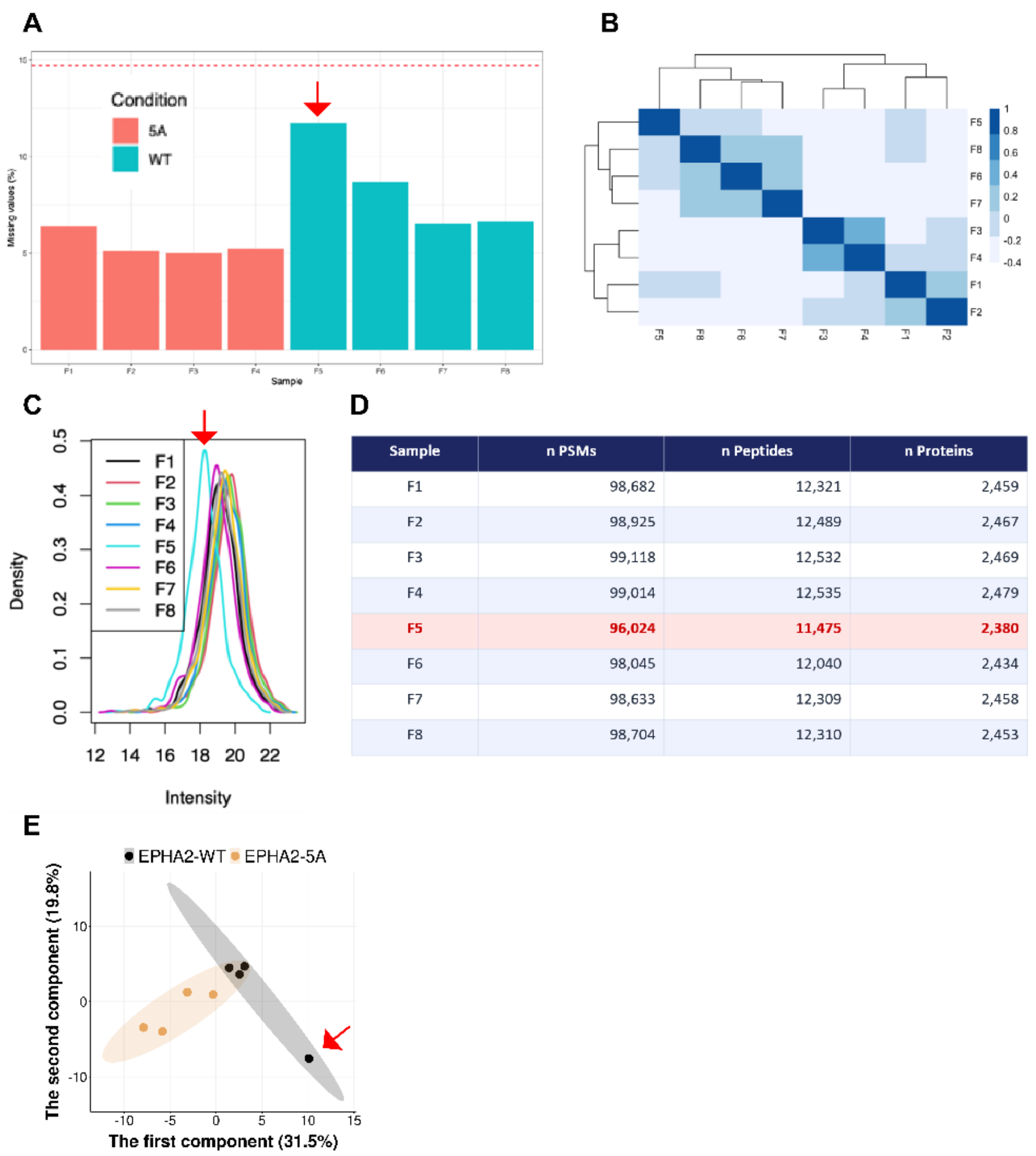

Sample	n PSMs	n Peptides	n Proteins
F1	98,682	12,321	2,459
F2	98,925	12,489	2,467
F3	99,118	12,532	2,469
F4	99,014	12,535	2,479
F5	96,024	11,475	2,380
F6	98,045	12,040	2,434
F7	98,633	12,309	2,458
F8	98,704	12,310	2,453

Figure 1 for reviewer

(A) Missing-value fraction for each sample. F5 showed the highest missingness.

(B) Inter-sample correlation heatmap with hierarchical clustering showing weaker similarity of F5 to the other WT samples.

(C) Density plot of MS intensities showing a left-shifted distribution for F5.

(D) Numbers of confidently detected Peptide-Spectrum Matches (PSMs), peptides, and proteins detected in each sample, showing reduced identification depth for F5.

(E) Principal component analysis (PCA) (**original Fig. EV6A**) showing separation of F5 from the other WT samples.

In response to the reviewer's concern, we have excluded this WT sample (F5) along with its batch-matched counterpart (F1 of EPHA2 5A), which were processed within the same experimental batch, to minimize potential batch effects. The entire dataset was subsequently re-analyzed. Removal of these samples increased the variance explained by PC1 from 31.5% to 41.0%, indicating improved dataset coherence and a clearer separation of the dominant biological signal while preserving the original biological conclusions. The updated PCA is now presented in **revised Fig. 3B**. In accordance with recommendations from Reviewer #2 and #3, we further include pathway enrichment analysis in **revised Fig. 3C** and proteomic profiling of mitochondrial electron transport chain (ETC) subunits in **revised Fig. 3D**.

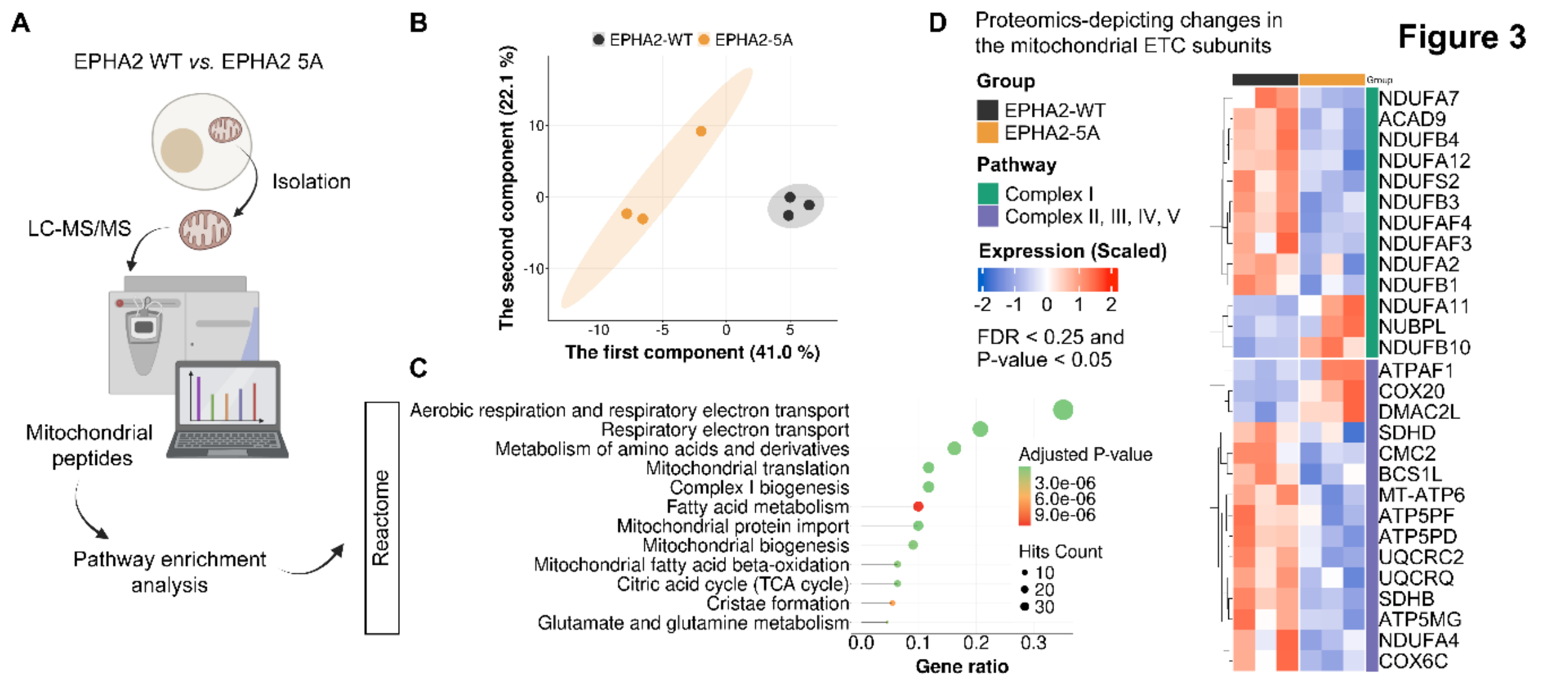

Figure 3

(A) Experimental workflow for label-free quantitative proteomics of mitochondria isolated from inducible EPHA2 WT and EPHA2 5A SH-SY5Y cells.

(B) Principal component analysis of mitochondrial proteomes, showing separation of EPHA2 WT and EPHA2 5A samples.

(C) Reactome pathway enrichment analysis of differentially abundant mitochondrial proteins.

(D) Heatmap of altered electron transport chain proteins, grouped by respiratory complex.

Additionally, a volcano plot, proteomic changes in total mitochondrial proteins, and a more focused analysis of selected mitochondrial pathways are provided in **revised Fig. EV6**. Statistical thresholds of false discovery rate (FDR) < 0.25 and P-value < 0.05 are explicitly indicated in the revised figures.

Figure EV6

(A) Volcano plot of differentially abundant mitochondrial proteins in inducible EPHA2 WT and EPHA2 5A cells.

(B) Heatmap of significantly altered mitochondrial proteins.

(C) Analysis of selected mitochondrial pathways revealed that mitochondrial dynamics, TCA cycle, and calcium signaling were also sensitive to disruption of ligand-independent EPHA2 signaling.

Proteins shown met the significance thresholds used in the proteomic analysis. Exact thresholds are indicated in the figure.

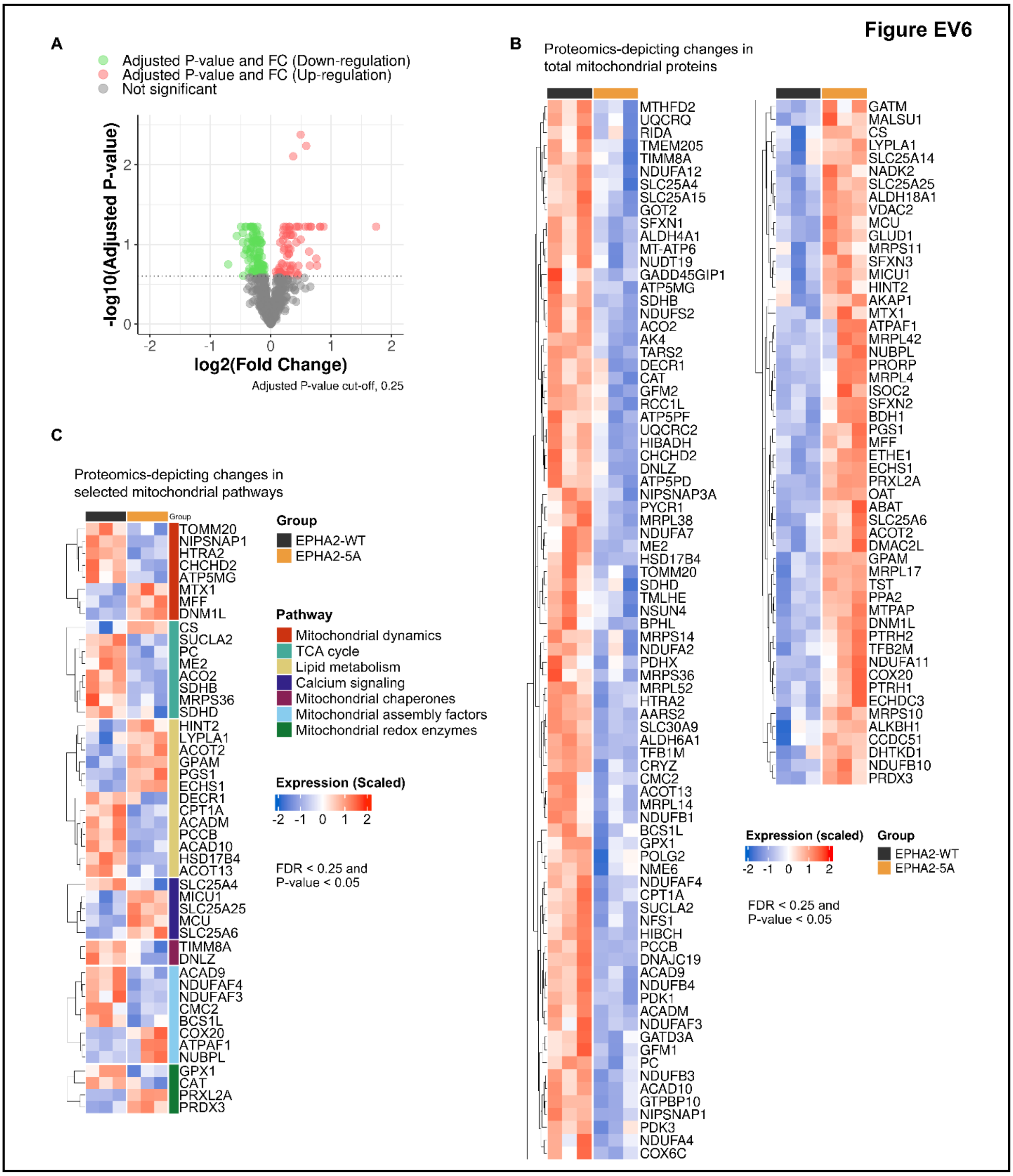

7) Citations are missing for several statements throughout the text, please revise. Just as two examples: in line 2, add the citation that shows that/which other family members of EPH family are associated with brain development; and in lines85/87, add the references for well-characterized mutational sites on EPHA2 and for Ser897 being important for non-canonical signaling.

We have systematically reviewed the manuscript and incorporated appropriate citations where previously absent. Specifically, statements regarding the role of EPH receptors in brain development in the Introduction are now supported by Wilkinson (Nat Rev Neurosci,

2001) and Rasool & Jahani-Asl (Transl Psychiatry, 2024) (*page 3, lines 26-29*). References for EPHA2 mutational sites and the non-canonical Ser897 phosphorylation site have been added, citing Lechtenberg *et al.* (Nat Commun, 2021) (*page 3, line 35*) and Miao *et al.* (Cancer Cell, 2009) (*page 3, line 29*). In addition, Melo-Braga *et al.* (Mol Cell Proteomics, 2014) is now cited to provide context for NSPC phosphosite analyses (*page 3, line 42*). These citations have been inserted at the relevant statements and are reflected accordingly in the reference list.

8) In figure 5, to fully prove that mitochondrial metabolism is controlled by ligand-independent EPHA2 signaling, it should be shown that kinase-dead mutants, unlike 5A, can rescue the mitochondrial defects.

We thank the reviewer for this point. We have expanded the analysis of mitochondrial NAD^+/NADH by comparing control, EPHA2 KO, and rescue conditions with EPHA2 WT, 5A, and K646R in **revised Fig. EV7C**. To achieve this, we employed the genetically encoded RexYFP sensor (Bilan *et al*, 2014), in which NADH binding to the Rex domain induces a conformational change that decreases cpYFP fluorescence. Because mitochondrial Complex I oxidizes NADH to NAD^+, impaired Complex I activity results in NADH accumulation and a concomitant reduction in RexYFP intensity. Since cpYFP fluorescence is pH-sensitive, we normalized RexYFP signals to the pH sensor SypHer red to eliminate pH-dependent artifacts. Accordingly, when expressed as a SypHer/Rex ratio, reduced Complex I activity leads to an increase in the normalized signal. Using this approach, we observed a significant increase in the mitochondrial SypHer red/RexYFP ratio in EPHA2 KO cells. This phenotype was partially rescued by EPHA2 WT and the kinase-dead mutant K646R, but not by the 5A mutant, indicating that the kinase-dead variant, in contrast to 5A, is capable of restoring mitochondrial NAD^+/NADH homeostasis.

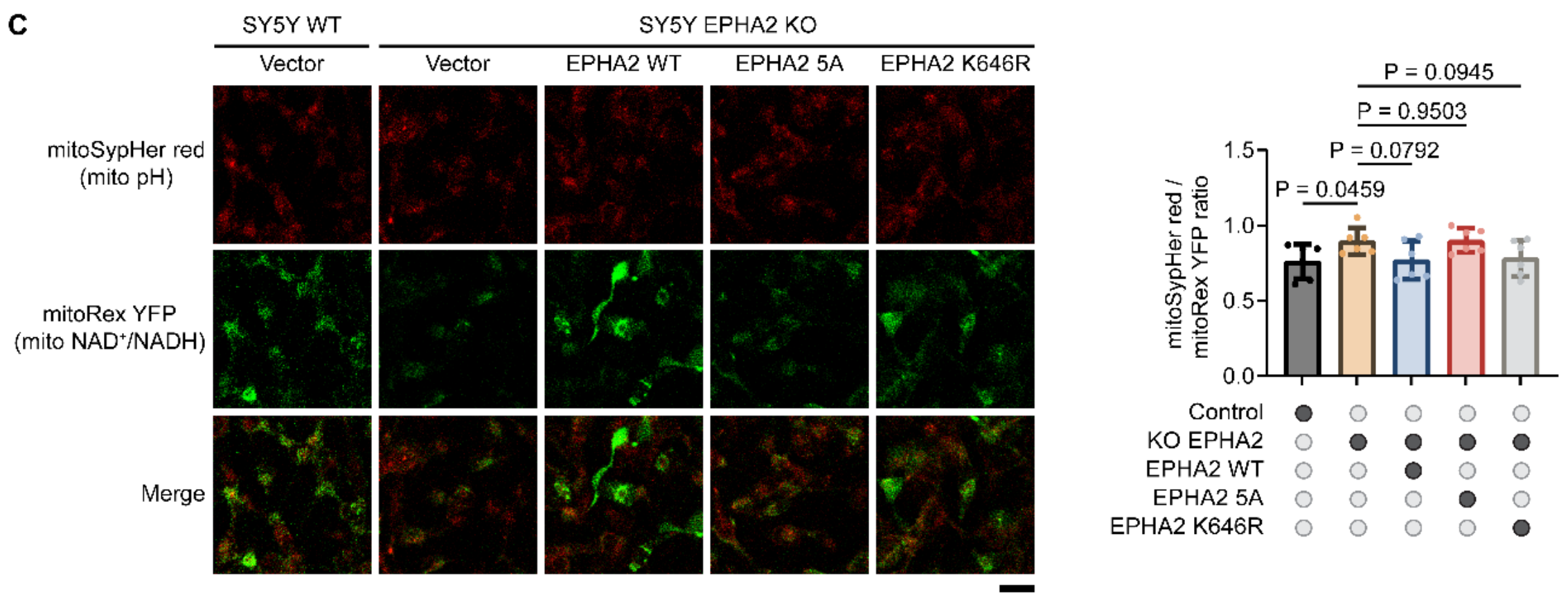

Figure EV7

(C) Ratiometric mitoRexYFP and mitoSypHer red imaging showing rescue of the mitochondrial redox phenotype by EPHA2 WT and EPHA2 K646R, but not by EPHA2 5A. Scale bar, 30 µm.

Other minor points:

1) In the introduction, lines 5-6: it would be clear if the shift from symmetric self-renewal to asymmetric self-renewal/differentiation was mentioned. As it is, the text seems to suggest RG do not self-renew anymore.

We agree that the original wording may have inadvertently implied a loss of self-renewal capacity of RGCs at later stages. We have therefore revised the sentence to: "During early corticogenesis, radial glial cells (RGCs) in the ventricular zone (VZ) undergo symmetric proliferative divisions to expand the progenitor pool. As development progresses, asymmetric neurogenic divisions become predominant, generating neurons directly or via intermediate progenitors" (*page 3, lines 3-6*). By specifying that asymmetric neurogenic divisions become predominant, the continued self-renewal capacity of RGCs at later stages is implicitly preserved.

2) Through the paper experiments are done in either HEK293T or SH-SY5Y lines, please state in the text and figures clearly when each cell type is used.

In the revised manuscript, we have explicitly annotated the cell type in all figure panels and corresponding legends (*e.g.*, "SY5Y cell", "HEK293T cell", "Neuro-2a cell", "Primary NSPCs").

3) The authors make a strong point dismissing cancer contexts as not able to replicate physiological conditions (line 122). However, they mostly use the glioblastoma line SH-SY5Y for their experiments, the implication of which should be discussed.

We appreciate the reviewer's thoughtful comment. In addition to the primary NSPC mt-SoNar measurements and *in vivo* IUE data, we have further validated key findings by examining the mitochondrial localization of ECSIT in primary NSPCs (**revised Fig. 5D**). We also assessed basal mitochondrial respiration and glycolytic activity in primary NSPCs expressing EPHA2 WT *versus* the 5A mutant (**revised Fig. 3F–H**), as well as Complex I enzyme activity in mitochondria isolated from embryonic brain tissue (**revised Fig. 3J**). Furthermore, the Discussion has been revised to explicitly state that SH-SY5Y is a neuroblastoma cell line. Its use is now clearly contextualized as a mechanistic model that complements, but does not replace, primary NSPC and *in vivo* metabolic analyses, thereby acknowledging this as a limitation of the study (*page 9, lines 288-290*).

4) In Figure 1F, the SOX2 staining did not work, as it does not appear nuclear. Please revise. In the same figure, it is interesting that EPHA2 does not seem to be only in the apical membrane, as the authors stated in the introduction. A comment on this would be clarifying.

We thank the reviewer for raising this point. In the original submission, SOX2 immunostaining was acquired using a 60× objective, which limited the clarity of nuclear signals. In the revised manuscript, we have re-acquired these images using a 20× objective with digital zoom, resulting in improved visualization of the nuclear SOX2 signal (**revised Fig. 1F**), and have replaced the panel accordingly. Regarding EPHA2 localization, we acknowledge that its distribution is not restricted to the apical membrane. We have revised the text to clarify this point and now state: "Immunostaining of brains at E15.5 confirmed EPHA2 enrichment in the VZ, beyond known vascular expression (Fig. 1F), in line with the transcriptomic profile" (*page 4, lines 68-70*).

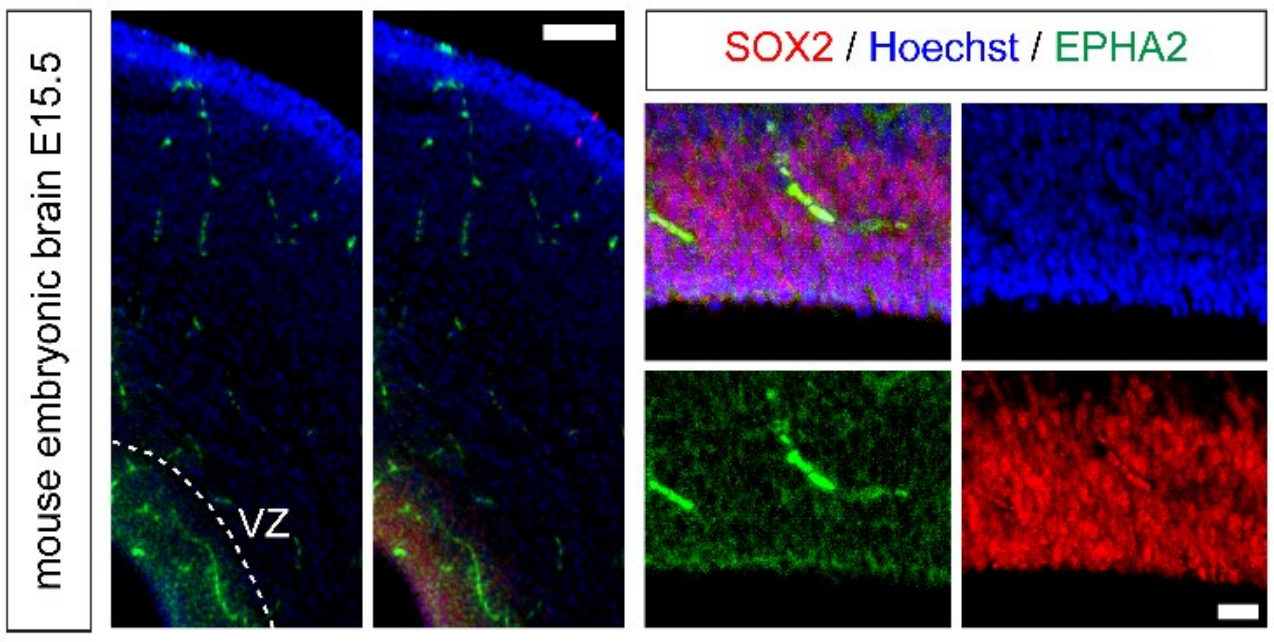

Figure 1

(F) Immunofluorescence of E15.5 mouse cortex stained for SOX2 and EPHA2, showing enrichment of EPHA2 in the ventricular zone. Scale bars, 100 µm (left), 20 µm (right).

5) In some places in the text, such as in figure 5, it is unclear if the authors are still using the KD of EPHA2, please revise and state it clearly (I assume yes).

We thank the reviewer for highlighting this point. **Revised Fig. 4A, B**; **4E, F**; **Fig. 5E–H**, and **Fig. 6F–H** are now explicitly annotated as EPHA2 KD with rescue experiments. The figure panels and legends have been updated to clearly indicate "KD + rescue" and to specify the constructs used (EPHA2 WT or 5A ± ECSIT variants).

Referee #2:

In this manuscript, Diem Nghi and colleagues report that the mitochondria complex I assembly factor ECSIT is controlled by noncanonical EPHA2 signaling to maintain the renewal of neural stem and progenitor cells (NSPCs). They suggest that ligand-independent EPHA2 signaling activates PP2A-mediated dephosphorylation of ECSIT, which in turn allows ECSIT to enter mitochondria and assemble complex I. This enables NSPCs to proliferate due to their ability to generate NAD+.

This is a data heavy manuscript, and the authors have undertaken a huge amount of work, and in many cases, with significant quantification.

While the figures were well constructed, the manuscript was challenging to read and interpret the results given the brief nature of the written material and the short figure legends. The lack of label for each figure also made it less easy to review. In my review, I will focus on the mitochondrial aspects of the manuscript. In summary, I do not believe that the authors are able to demonstrate a specific complex I defect, not a specific loss of ECSIT targeting to mitochondria that affects assembly and NAD levels.

We thank the reviewer for the careful assessment and apologize for the lack of clarity in the original submission. In the revised manuscript, we improved readability by adding explicit figure panel labels, expanding the figure legends to clarify the experimental design, and refining the Results section to ensure that the interpretation remains confined to what is directly supported by the present data and does not overreach.

1) In Fig. 3, the effects on mitochondrial function following expression of EPHA2 5A are quite subtle given the suggestion that complex I activity is perturbed. I am concerned about the cell based imaging approaches used to assess mitochondrial function and this needs to be strengthened. In 3B, membrane potential effects would be more strongly reduced with a complex I depletion. A Seahorse analysis is the norm for looking at this. In 3C, there is no

rationale for decreased ROS in 5A cells. The suggestion of a disrupted NAD+/NADH ratio in 5A cells (Fig. EV5E) was performed by looking at a mitoSypHer/mitoRexYFP ratio. This is a critical element of the manuscript and the range is really variable across the cells so it is unclear if there is a strong effect. It would be better tested using an enzymatic approach.

We thank the reviewer for raising this point. In the originally submitted manuscript, our conclusion relies on basal oxygen consumption (measured by MitoXpress Xtra oxygen consumption assay) and a Complex I enzymatic assay (defined as NADH oxidation to NAD$^+$) (**original Fig. 4E, F**), with the imaging data treated as supportive. We agree that the original imaging-based readouts were modest and, on their own, may not sufficient to establish a Complex I defect. In response, we have incorporated Seahorse XF respirometry in SY5Y cells and primary embryonic NSPCs, and enzymatic measurements of Complex I activity in mitochondria isolated from SY5Y cells and embryonic brains.

In SY5Y cells, EPHA2 5A reduced basal respiration, maximal respiration, and ATP-linked respiration, with no significant change in proton leak (**revised Fig. 3E**). In primary NSPCs, EPHA2 5A also reduced basal mitochondrial respiration and basal glycolytic activity (**revised Fig. 3F–H**). In addition, we performed enzymatic measurements of Complex I activity in isolated mitochondria. EPHA2 5A reduced mitochondrial NADH dehydrogenase activity in both SY5Y cells and embryonic brain tissues (**revised Fig. 3I, J**). Together, these data support impaired mitochondrial respiration with reduced Complex I activity in the EPHA2 5A condition.

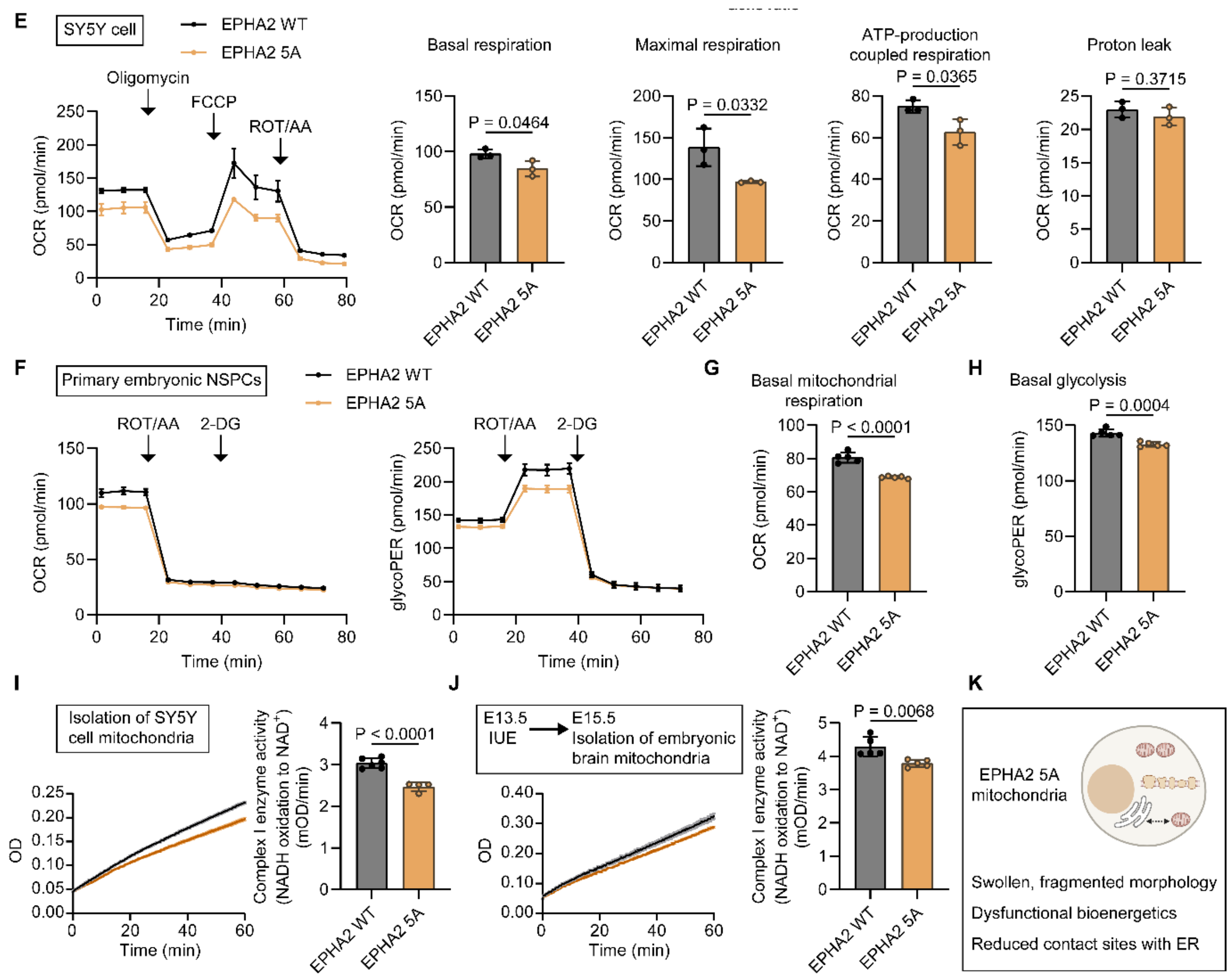

Figure 3

(E) Seahorse analysis of oxygen consumption (Mito Stress Test) in inducible EPHA2 WT and EPHA2 5A SH-SY5Y cells, with quantification of basal, maximal, ATP-linked respiration, and proton leak.

(F) Seahorse analysis of oxygen consumption and proton efflux rate (Glycolytic Rate Assay) in primary NSPCs expressing EPHA2 WT or EPHA2 5A.

(G) Quantification of basal mitochondrial respiration in primary NSPCs.

(H) Quantification of basal glycolytic activity in primary NSPCs.

(I) Complex I enzyme activity in mitochondria isolated from inducible EPHA2 WT and EPHA2 5A SH-SY5Y cells. mOD/min, millioptical density per minute.

(J) Complex I enzyme activity in mitochondria isolated from embryonic brains expressing EPHA2 WT or EPHA2 5A. mOD/min, millioptical density per minute.

(K) Schematic of mitochondrial defects in EPHA2 5A cells. Illustrations created with BioRender.com.

Addressing the reviewer's point, we have moved the TMRM and MitoSOX data to the Expanded View (**revised Fig. EV5E, F**) for better presentation. We also moderated the interpretation accordingly. Together with the electron microscopy-based mitochondrial morphometric analyses, these data support perturbed mitochondrial homeostasis in EPHA2 5A, but do not by themselves define the primary lesion. The mitoSypHer/mitoRexYFP biosensor imaging data (**revised Fig. EV7C**) provide supportive evidence for an altered mitochondrial redox state, consistent with the reduced Complex I NADH dehydrogenase activity established by direct enzymatic assays.

2) The proteomic approach shows incredible variability in OXPHOS subunit levels in EPHA2-5A expressing cells - and not specific to complex I. For example, in Fig 4C, complex V subunit (MT-ATP6), two complex II subunits (SDHB, SDHD), a complex III subunit (UQCRQ) and a complex IV subunit (COA3) are all down while some subunits are up. This does not at all point to a specific reduction in a complex I assembly factor. In fact, loss of ECSIT leads to a specific loss of complex I subunits and virtually all of them are downregulated as they are proteolytically degraded if unassembled (see PMID: 27626371). The authors need to show whether there is an assembly defect using blue native PAGE and compare it to other complexes.

We thank the reviewer for this important point. We agree that the mitochondrial proteomics dataset in the original manuscript did not, by itself, establish a specific Complex I defect, as several ETC subunits outside Complex I were also altered. In the revised manuscript, we have therefore repositioned this dataset as an unbiased survey of mitochondrial proteome remodeling and avoided describing it as evidence for Complex I specificity (**revised Fig. 3A–D**).

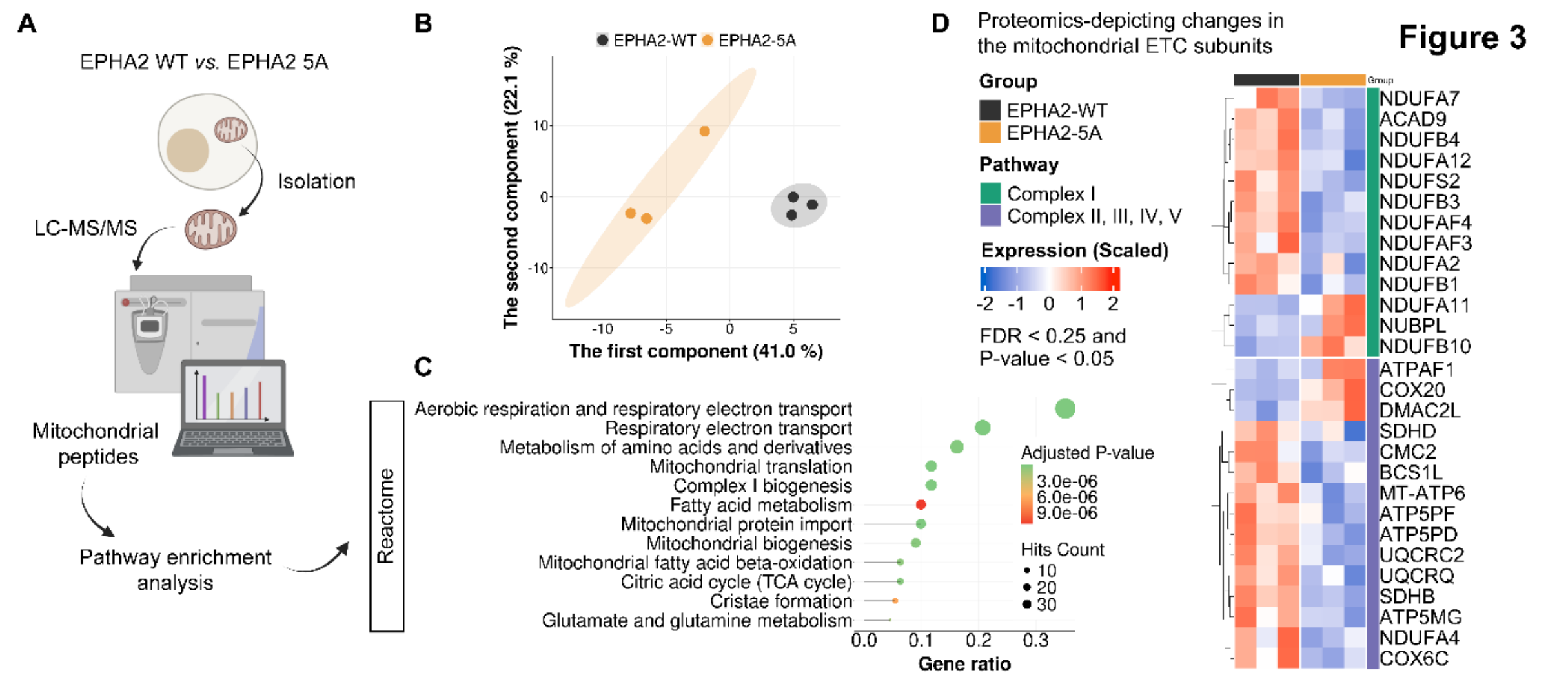

Figure 3

(A) Experimental workflow for label-free quantitative proteomics of mitochondria isolated from inducible EPHA2 WT and EPHA2 5A SH-SY5Y cells.

(B) Principal component analysis of mitochondrial proteomes, showing separation of EPHA2 WT and EPHA2 5A samples.

(C) Reactome pathway enrichment analysis of differentially abundant mitochondrial proteins.

(D) Heatmap of altered electron transport chain proteins, grouped by respiratory complex.

We agree that BN-PAGE is the appropriate assay to assess whether EPHA2 perturbation causes a stable respiratory Complex I assembly defect relative to the other OXPHOS complexes. We therefore performed BN-PAGE followed by immunoblotting for NDUFB11, COX5B, and SDHA. Under our conditions, we did not detect a drastic difference between EPHA2 WT and EPHA2 5A in the native bands detected with these markers. Because the Complex I readout relies on NDUFB11, we cannot exclude changes in other Complex I subunits or assembly intermediates that are not captured by our assay. We have therefore revised description to avoid implying selective holo-Complex I loss or a definitive Complex I assembly defect.

Figure 2 for reviewer

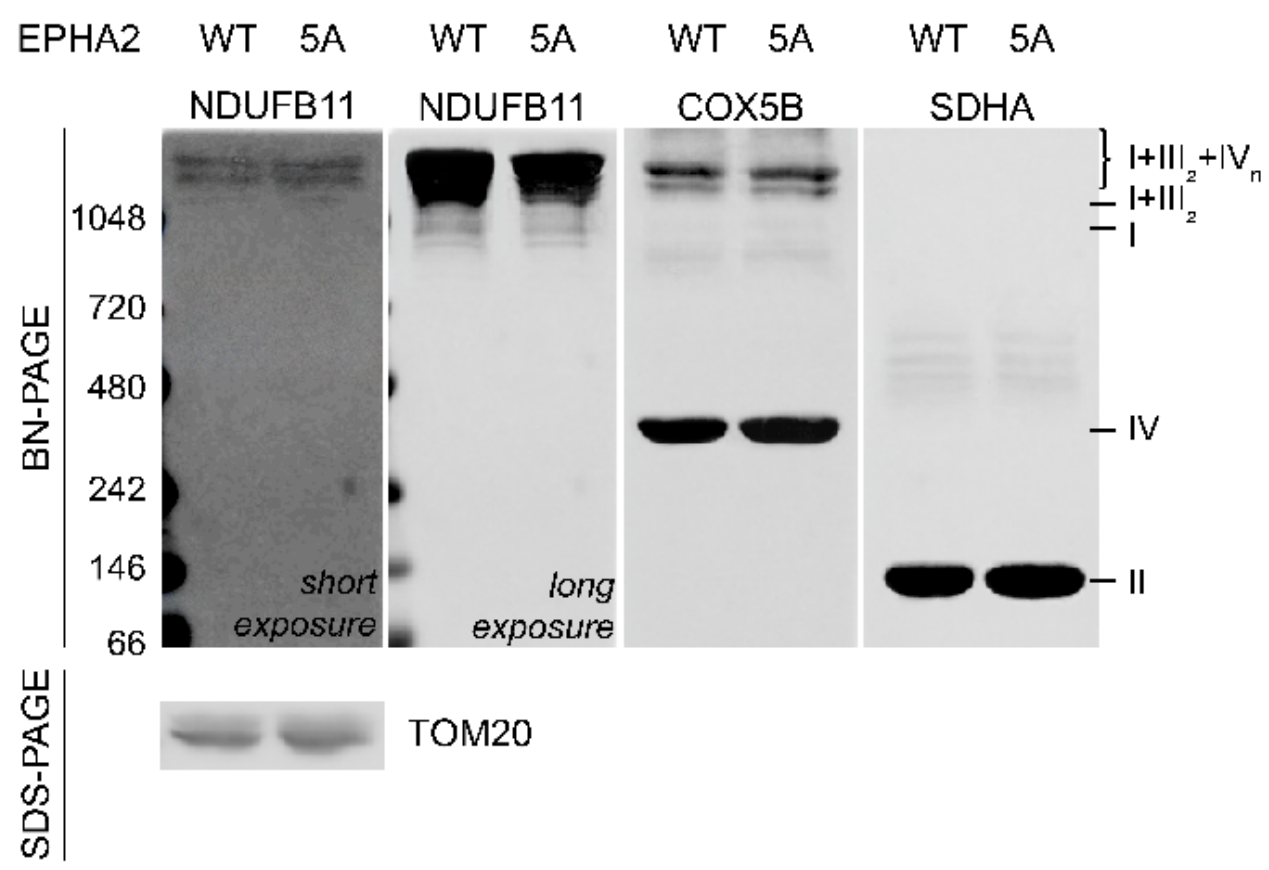

Isolated mitochondria from inducible EPHA2 WT and 5A cells were analyzed by BN-PAGE following the published workflow (Timón-Gómez *et al*, 2020). Briefly, mitochondrial pellets were resuspended in aminocaproic acid/Bis-Tris buffer under native conditions, solubilized with digitonin, clarified by centrifugation, supplemented with Coomassie G-250-containing native loading buffer, and resolved on NativePAGE 3%-12% Bis-Tris gels. Electrophoresis was run first in dark blue cathode buffer and then in light blue cathode buffer, after which the gels were processed for immunoblotting with antibodies against NDUFB11, COX5B, and SDHA. TOM20 was analyzed in parallel by SDS-PAGE as a mitochondrial loading control.

Accordingly, we revised the conclusion based more on direct functional data including newly provided over the revision: (1) Pathway enrichment analysis highlights Complex I biogenesis alongside respiratory electron transport (**revised Fig. 3C**) and respiratory-chain heatmaps show broad ETC remodeling with notable representation of Complex I-related proteins (**revised Fig. 3D**). (2) Seahorse analyses in SY5Y cells and primary embryonic NSPCs show reduced mitochondrial respiration in the EPHA2 5A condition (**revised Fig. 3E–H**). (3) Direct enzymatic assays in isolated mitochondria from SY5Y cells and embryonic brain further show reduced Complex I NADH dehydrogenase activity (**revised Fig. 3I, J**). These data support impaired mitochondrial respiration with reduced Complex I-linked NADH oxidation.

In this line, we would like to emphasize that the selection of ECSIT was not based solely on the proteomics dataset nor on a targeted selection of an assembly factor. It was prioritized as a hypothesis-driven candidate because it is known to be functionally linked to Complex I, exist in a regulated mitochondrial pool rather than as a constitutive resident alone, and be compatible with a role in progenitor maintenance (Vogel *et al*, 2007; Xiao *et al*, 2003) (**revised Fig. EV10A**). To support the mechanistic relevance of ECSIT, we added several independent datasets. Mitochondrial ECSIT abundance was reduced in mitochondrial proteomics and biochemical fractionation, while total ECSIT abundance in whole cell lysate was not detectably reduced (**revised Fig. 5A, B**). PLA showed reduced proximity between ECSIT and ACAD9, a mitochondrial interactor of ECSIT; and there was a decreased co-localization of endogenous ECSIT with TOM20 in primary NSPCs (**revised Fig. 5C, D**). In addition, re-expression of ECSIT WT, but not mitochondria-targeting-deficient ECSIT ΔMTS, restored the mitochondrial redox readout and rescued NSPC maintenance phenotypes *in vitro* and *in vivo* (**revised Fig. 5E–H**).

We also acknowledge the reviewer's point that complete loss of ECSIT has been reported to cause broad depletion of Complex I subunits because unassembled components are degraded. Our experimental condition does not reproduce that null or total knockout phenotype. Instead, our data support a milder and more selective reduction in the mitochondrial pool of ECSIT associated with impaired Complex I-linked NADH oxidation.

3) Regarding the ECSIT localization (Fig 5), the two bands shown for ECSIT (fig 5B) does not indicate mitochondrial location. There is no doublet in the whole cell fraction yet it appears in the crude mito fraction. This is not clear and could be a degradation product. The authors would need to perform more detailed biochemical analysis here and include other control proteins.

We thank the reviewer for this important point. We agree that the doublet in the **original Fig. 5B** could be misinterpreted without additional controls. In the revised manuscript, we have strengthened this analysis in two ways.

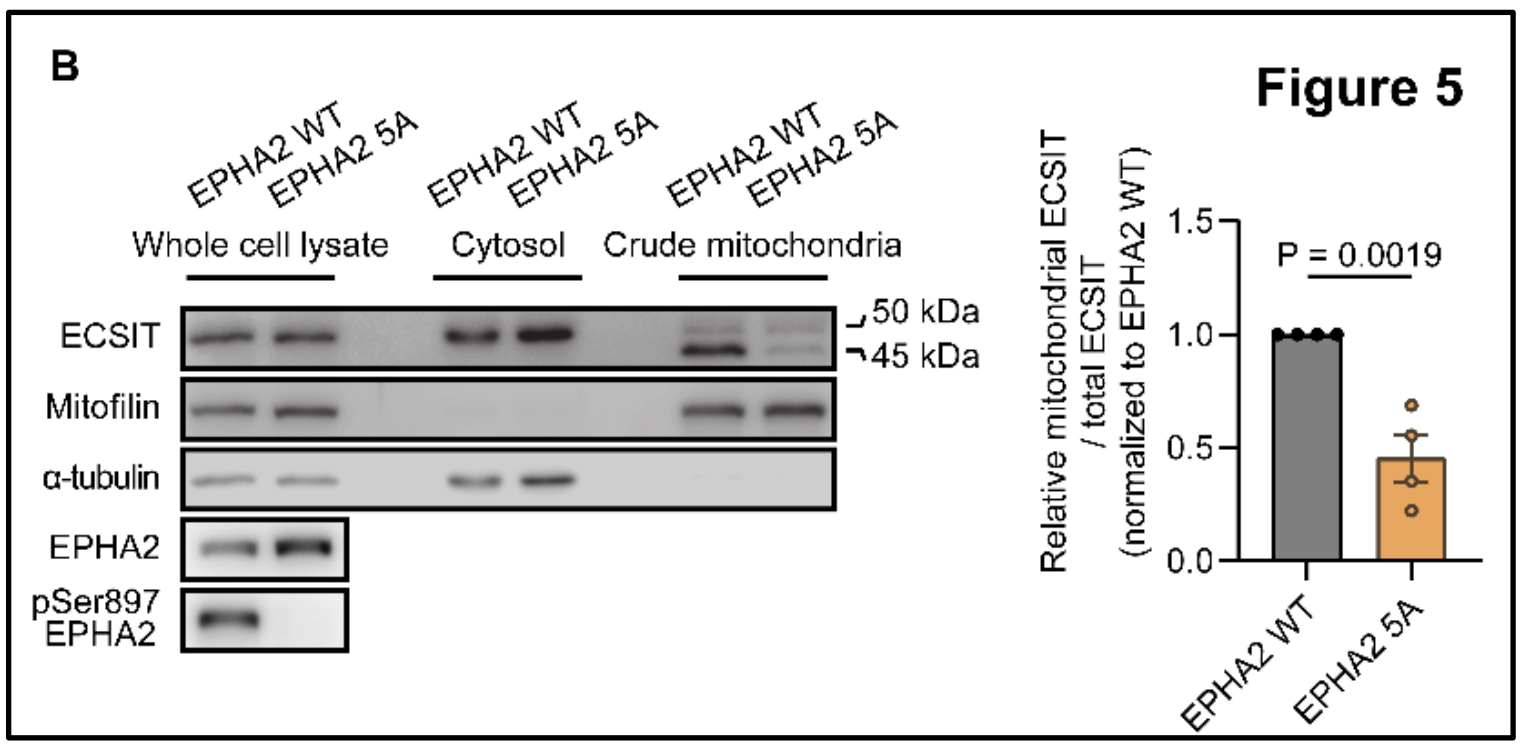

First, we repeated the fractionation with whole cell lysate, cytosolic fraction, and crude mitochondrial fraction loaded on the same blot, together with compartment markers, α-tubulin for cytosol and Mitofilin for mitochondria and relabeled the ECSIT species as ~50 kDa and ~45 kDa (**revised Fig. 5B**). In this experiment, the ~50 kDa species is enriched in the cytosolic fraction, whereas the ~45 kDa species is enriched in the mitochondrial fraction. We therefore quantified specifically the ~45 kDa mitochondrial species relative to total ECSIT.

Second, to directly address the reviewer's concern that the lower band could reflect degradation, we added a proteinase K protection assay using intact mitochondria (**revised Fig. EV10D**). The ~45 kDa ECSIT species was protease-protected, consistent with an imported mitochondrial form rather than a nonspecific degradation product. This interpretation is also consistent with prior work (Nicol *et al*, 2023; Vogel *et al.*, 2007) showing that ECSIT contains an N-terminal mitochondrial targeting sequence, localizes to mitochondria, and is present as a ~50 kDa precursor and a ~45 kDa processed mitochondrial species generated by cleavage of the targeting sequence. We have therefore revised the manuscript so that mitochondrial localization of ECSIT is supported by fractionation with compartment controls and proteinase K protection, rather than by the doublet alone.

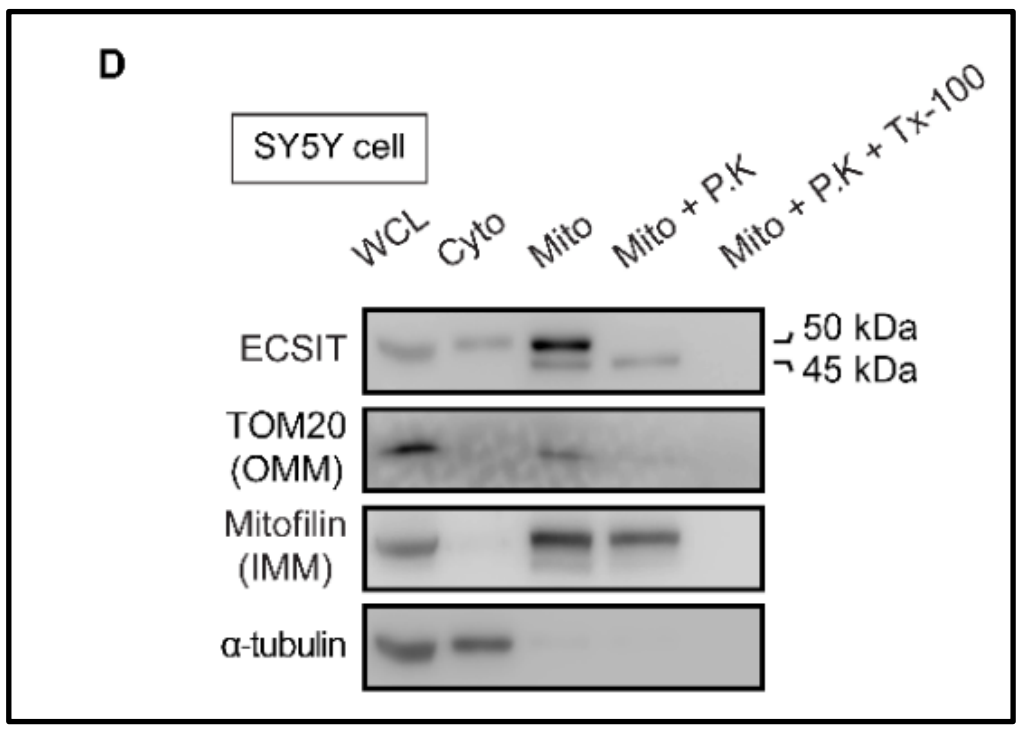

4) In Figure 6B, the authors do an IP with the PP2A regulatory subunit and enrich a "mitochondrial" ECSIT. But PP2A is in the cytosol so should not interact with a mito form. Another confounding factor is that a cytosolic form of a folded protein is unlikely to enter mitochondria after undergoing a dephosphorylation event.

We thank the reviewer for this important point. Because the immunoprecipitation was performed from whole-cell detergent lysate, mitochondria were solubilized and both ECSIT species (~50 kDa and ~45 kDa) were present in the same extract. Although the result supports a link between PP2A and ECSIT, it cannot definitively distinguish whether the recovered ECSIT corresponds to the cytosolic precursor, the processed mitochondrial species released during lysis, or an association formed after lysis. The experiment therefore does not establish that PP2A directly interacts with the mature mitochondrial form of ECSIT or define the subcellular site of action. Accordingly, we have replaced this panel with **revised Fig. 6C** and now base this part of the model on data showing a correlation between altered PP2A activity and ECSIT phospho-state downstream of EPHA2. Compared to EPHA2 WT, EPHA2 5A retains higher ECSIT threonine phosphorylation (**revised Fig. 6B**). In parallel, EPHA2 5A increases the level of PP2A inactive form, consistent with reduced PP2A activity (**revised Fig. 6C**). In addition, EPHA2 co-expression lowers ECSIT threonine phosphorylation, and this

effect is partially reversed by PP2A inhibition with LB-100 (**revised Fig. 6D**).

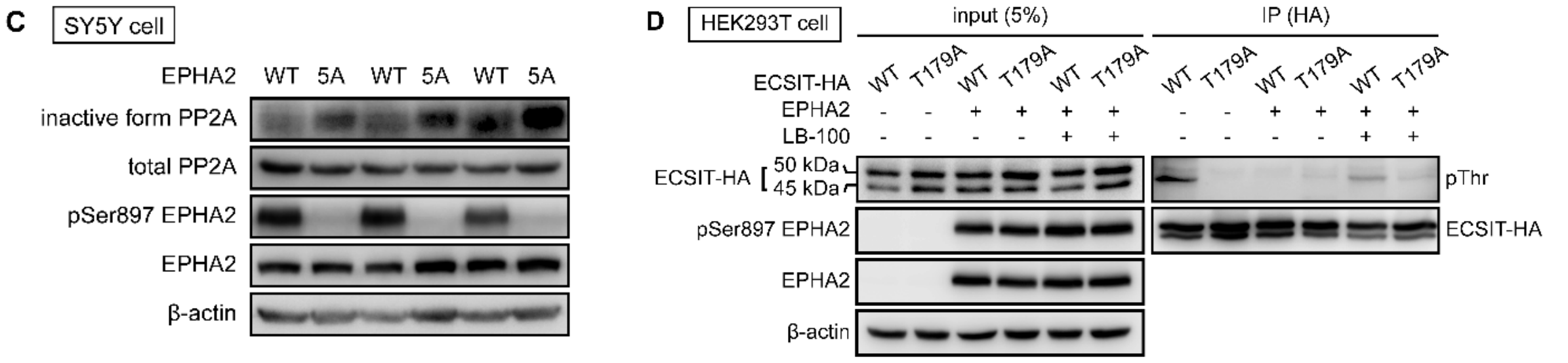

Figure 6

(C) Immunoblot analysis of PP2A Tyr307 phosphorylation, a marker of its inactive form, in inducible EPHA2 WT and EPHA2 5A cells.

(D) Reconstitution assay showing EPHA2-dependent reduction of ECSIT phospho-threonine signal and partial reversal by the PP2A inhibitor LB-100 (2.5 μM for 6 hours).

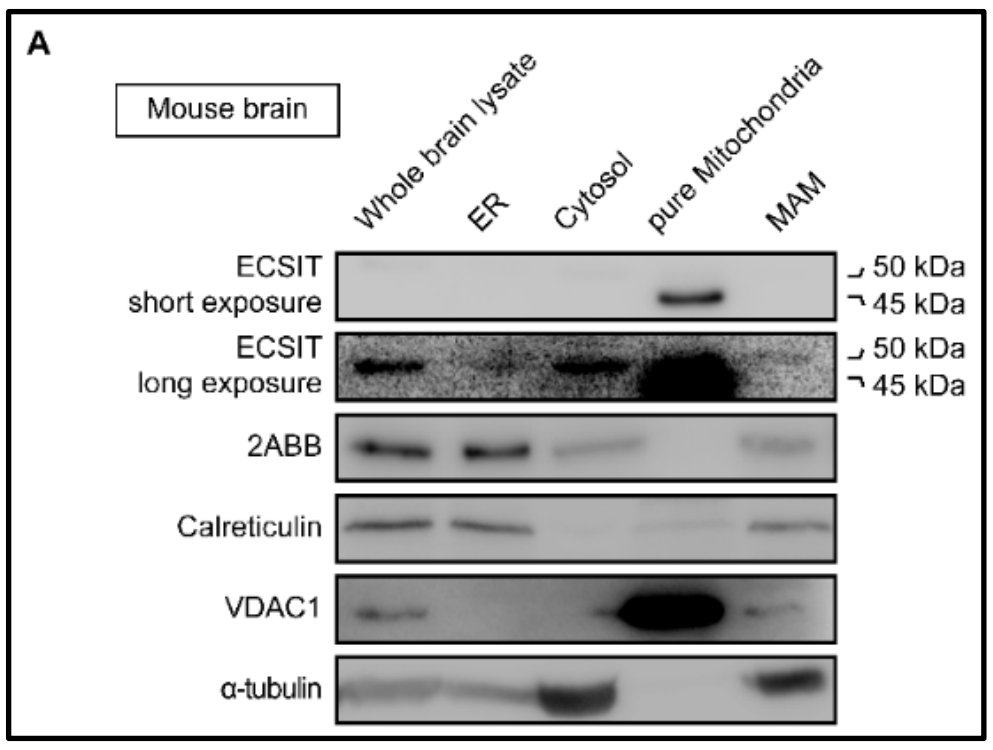

To address compartmentalization more directly, we added a Percoll gradient-based fractionation analysis showing that the processed ~45 kDa ECSIT species is enriched in purified mitochondria, whereas PPP2R2B/2ABB is not enriched in that fraction (**revised Fig. EV12A**). Moreover, the ~50 kDa ECSIT form and 2ABB are present in the cytosolic fraction and in mitochondria-associated membranes (MAMs). These data argue against PP2A acting on the mature intramitochondrial ECSIT pool. We therefore revised the manuscript and model throughout to avoid implying direct PP2A action on mature mitochondrial ECSIT.

We also agree that our original description could be read as suggesting that PP2A dephosphorylates a fully folded cytosolic ECSIT species and thereby enables mitochondrial import. In the revised manuscript, we restrict our interpretation to the narrower conclusion directly supported by our data: that PP2A activity is functionally linked to ECSIT threonine dephosphorylation downstream of EPHA2, with T179 identified as the functionally relevant site in our current assays. Perturbation of this pathway reduces mitochondrial ECSIT abundance and is associated with reduced Complex I enzymatic activity, without assigning this effect to a specific import stage or ECSIT form (fully folded cytosolic or mitochondria-proximal ECSIT precursor) (response to Comment #5). What remains unresolved are the precise steps being controlled, whether EPHA2 signaling influences import competence, mitochondrial retention, processing, or assembly-factor engagement after entry of ECSIT, and this will require further investigation.

5) It is well established that mitochondrial protein import requires proteins to be unfolded to enter mitochondria (they are imported post translation but before folding can occur). Suggesting that PP2A dephosphorylates Ecsit such that it can then be targeted to mitochondria, unfold and then refold in the matrix would be an unusual finding.

We thank the reviewer for this important conceptual point and agree that our original

wording was too strong. We admit that our data do not resolve the folding state of ECSIT, nor do they define the exact step at which phosphorylation affects the mitochondrial pool of ECSIT. To more explicitly reflect this limitation, we have revised the manuscript to state the narrower conclusion that PP2A is functionally linked to ECSIT threonine dephosphorylation downstream of EPHA2, with T179 as the relevant site in our current assays. Disruption of this pathway reduces mitochondrial ECSIT abundance and is associated with reduced Complex I activity.

This revised conclusion is supported by several independent observations. First, among the tested S/T-to-A mutants, only T179A increases mitochondrial enrichment relative to WT and the other phosphosite mutants (**revised Fig. 6A**). Second, EPHA2 reduces ECSIT threonine phosphorylation, and this effect is partially reversed by the specific PP2A inhibitor LB-100, supporting PP2A-dependent control of ECSIT phospho-state downstream of EPHA2 (**revised Fig. 6D**). Third, the phosphomimetic ECSIT T179E remains less enriched in mitochondria despite EPHA2 co-expression (**revised Fig. 6E**). Fourth, ECSIT WT, but not ECSIT T179E, rescues the Ki67 and SOX2 defects induced by EPHA2 5A *in vivo* (**revised Fig. 6F–H**). Finally, mitochondrial targeting is functionally required, because ECSIT WT, but not ECSIT ΔMTS, restores the mitochondrial redox readout and NSPC maintenance phenotypes in the EPHA2 5A condition (**revised Fig. 5E–H**). Phosphorylation-dependent regulation of mitochondrial precursor proteins has been documented in other systems, including MORF3 (Law *et al*, 2015), GSTA4-4 (Robin *et al*, 2003), CYP2B1 (Anandatheerthavarada *et al*, 1999), CNP2 (Lee *et al*, 2006), Tom22 precursor (Schmidt *et al*, 2011), and Tom40 precursor (Rao *et al*, 2012). However, we cite these studies only to illustrate that phospho-regulation can influence mitochondrial precursor handling or import in other contexts, not as direct evidence for the mechanism proposed here. Because our data do not resolve where ECSIT is dephosphorylated, whether pre-import ECSIT precursor is involved, or which step of mitochondrial import is affected, we avoid assigning PP2A activity to a specific import stage. Instead, the output directly supported by our data, after all, is the mitochondrial pool of ECSIT. Given the potential for misinterpretation, we removed the schematic (**original Fig. 6H**) and now explicitly state that the relevant molecular intermediates and precise subcellular steps remain unresolved in the current study (*page 10, lines 315-333*).

6) The ECSIT-GFP constructs in Fig Ev8 are also unclear. The authors are biased towards high expressing GFP expression and it is clear that the low expressing ECSIT forms are targeted into cells with colocalization with TOM20.

We thank the reviewer for this important point. We agree that the original representative images could be interpreted as being influenced by expression level. To make this clearer, we have replaced the representative images with single-channel ECSIT-GFP views that better illustrate ECSIT distribution independently of TOM20 merging (**revised Fig. 6A**). In fact, we analyzed the ECSIT-GFP localization data using an objective per-cell pipeline. For each construct, all transfected cells within the imaged fields that were above background and below saturation were included in the analysis, without selecting for highly expressing cells. Images were acquired using identical settings across constructs, and co-localization with TOM20 was quantified on a per-cell basis using Costes auto-thresholding and Manders' overlap coefficient (M1, fraction of ECSIT signal overlapping TOM20).

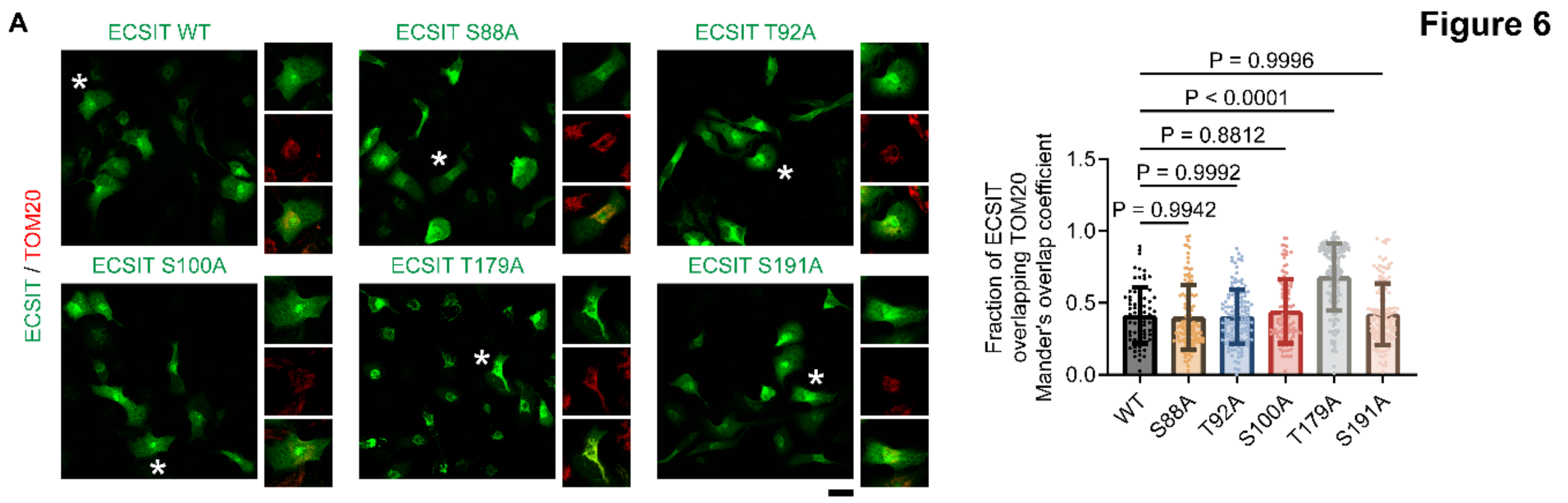

[Figure 6](#)

(A) Representative images of ECSIT WT and phosphosite mutants expressed in SH-SY5Y cells, with TOM20 staining. The asterisk-marked cell is in focus. Quantification of mitochondrial co-localization. Scale bar, 20 μm.

[Figure 3 for reviewer](#)

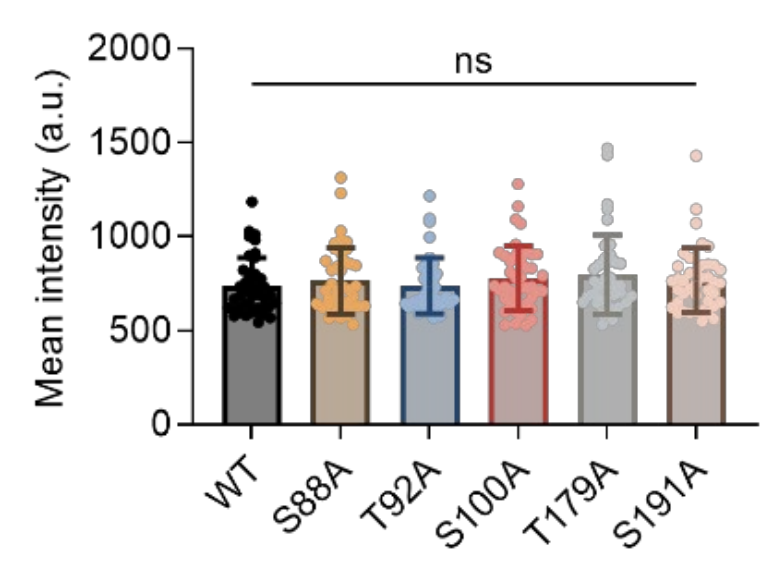

To test the reviewer's concern regarding expression-level bias, we quantified ECSIT-GFP mean intensity for every analyzed cell. We did not detect significant differences in GFP intensity among WT, S88A, T92A, S100A, T179A, and S191A constructs, indicating that the increased TOM20 overlap observed for ECSIT T179A is not explained by preferential inclusion of more highly expressing cells.

As noted by the reviewer, some mitochondrial co-localization is detectable across all constructs, including low-expressing cells. This assay is therefore not intended to suggest that only ECSIT T179A localizes to mitochondria, but rather to show that T179A increases mitochondrial enrichment relative to WT and the other phosphosite mutants across the analyzed cell population. Together with this imaging-based assay, the conclusion that ECSIT T179 phospho-state regulates mitochondrial ECSIT accumulation is independently supported by immunoblot analysis and functional rescue experiments.

Referee #3:

This manuscript reports the discovery of a ligand-independent EPHA2 signaling axis that maintains neural stem and progenitor cell (NSPC) self-renewal through mitochondrial Complex I-dependent NAD^+ regeneration. The authors demonstrate that EPHA2, enriched in radial glia and intermediate progenitors, operates independently of ephrin ligands to sustain proliferation by preventing premature cell cycle exit. Mechanistically, ligand-free EPHA2 promotes PP2A-mediated dephosphorylation of ECSIT at threonine 179, enabling its mitochondrial translocation, Complex I integration, and NAD^+ regeneration. Disruption of this pathway reduces Complex I activity, alters mitochondrial morphology, and impairs progenitor proliferation, which can be rescued by maternal NAD^+ supplementation.

This study uncovers a novel mechanistic link between membrane receptor signaling and mitochondrial metabolism in developing neural progenitors, addressing a long-standing gap

With that, there are numerous things to address to strengthen this manuscript and the major conclusions.

1) The model shows that EPHA2 activation promotes PP2A-mediated dephosphorylation of ECSIT, yet the data remain correlative. Evidence of direct interaction or activation between EPHA2 and PP2A would strengthen causality. Alternatively, determining whether PP2A activity toward ECSIT is lost upon EPHA2 inhibition would address this gap.

We agree that our original data did not sufficiently establish the PP2A step. Our revised manuscript has incorporated multiple experiments that functionally place PP2A downstream of EPHA2 and upstream of ECSIT dephosphorylation.

First, ECSIT threonine phosphorylation is increased in EPHA2 5A cells relative to EPHA2 WT cells, indicating that ECSIT dephosphorylation is impaired when EPHA2 signaling is perturbed (**revised Fig. 6B**). Second, in the newly added data, the inactive form of PP2A, monitored by Tyr307 phosphorylation, is increased in EPHA2 5A cells without an obvious change in total PP2A, consistent with reduced PP2A activity under the same condition (**revised Fig. 6C**). Third, in a reconstitution assay, EPHA2 co-expression markedly reduces ECSIT threonine phosphorylation, whereas pharmacological PP2A inhibition with LB-100 partially restores the ECSIT pThr signal despite EPHA2 activation, monitored by Ser897 phosphorylation (**revised Fig. 6D**). These data indicate that EPHA2-dependent ECSIT dephosphorylation requires PP2A activity. Importantly, we also tested the functional relevance of the mapped ECSIT phosphosite. EPHA2 increases mitochondrial ECSIT abundance in a PP2A-sensitive manner, whereas the phosphomimetic ECSIT T179E mutant fails to show this response (**revised Fig. 6E**). *In vivo*, ECSIT WT, but not ECSIT T179E, rescues the progenitor phenotype caused by EPHA2 5A expression, further supporting T179 dephosphorylation as the relevant downstream event (**revised Fig. 6F–H**).

Figure 6

(B) Immunoprecipitation of endogenous ECSIT followed by phospho-threonine immunoblotting in inducible EPHA2 WT and EPHA2 5A cells.

(C) Immunoblot analysis of PP2A Tyr307 phosphorylation, a marker of its inactive form, in inducible EPHA2 WT and EPHA2 5A cells.

(D) Reconstitution assay showing EPHA2-dependent reduction of ECSIT phospho-threonine signal and partial reversal by the PP2A inhibitor LB-100 (2.5 μM for 6 hours).

(E) Mitochondrial localization assay comparing ECSIT WT and ECSIT T179E in the presence or absence of EPHA2 co-expression and PP2A inhibition, with quantification of mitochondrial ECSIT relative to total ECSIT.

(F–H) IUE at E13.5 with RFP-KD EPHA2 + RFP-EPHA2 WT, RFP-EPHA2 5A together with either EGFP-empty vector, ECSIT WT, or ECSIT T179E. Analysis at E14.5.

(F) GFP RFP (green), Ki67 (red), and SOX2 (magenta) quadruple IF. Scale bar, 50 μm.

(G) Quantification of Ki67-positive electroporated cells.

(H) Quantification of SOX2-positive electroporated cells.

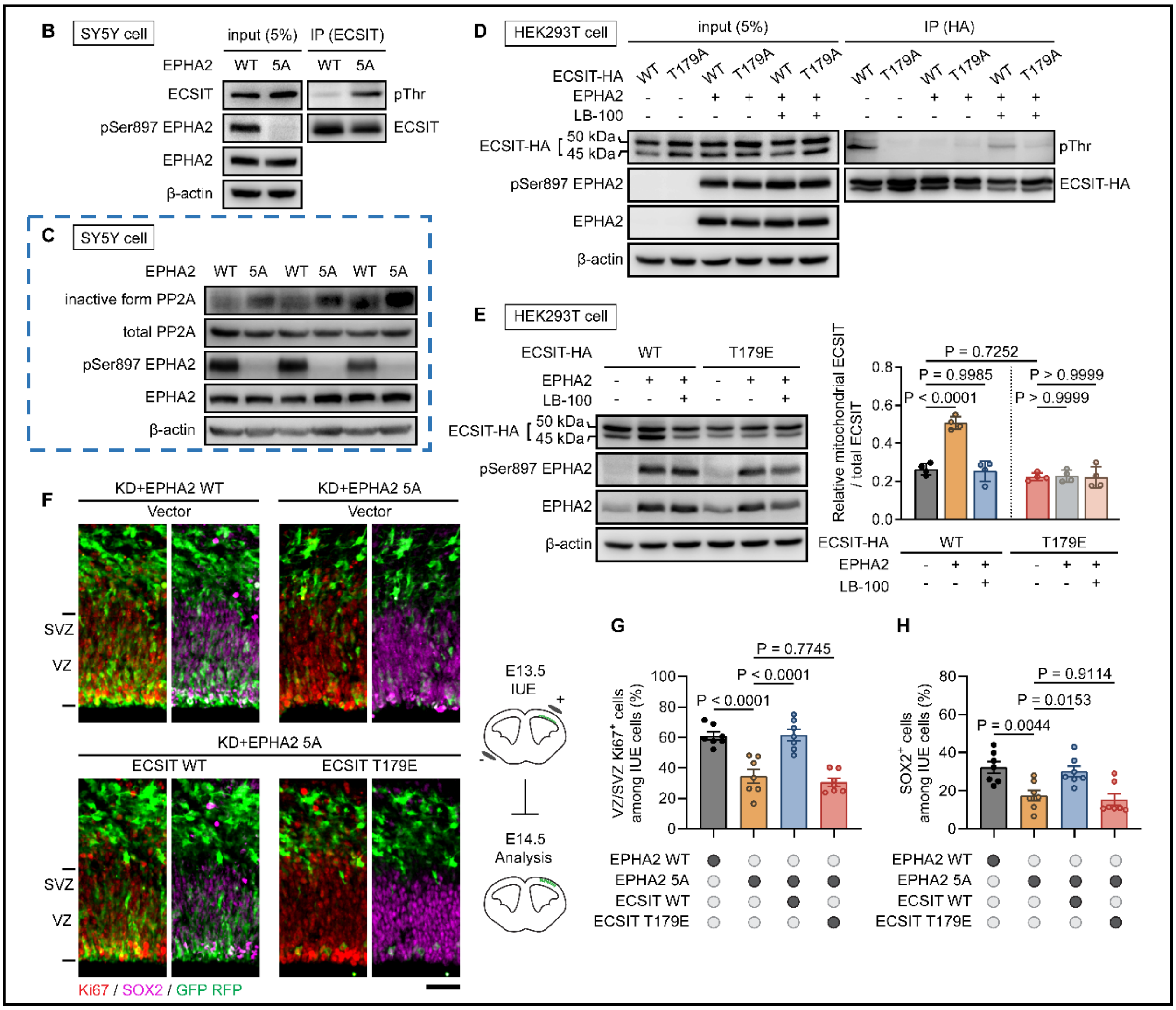

Together, these data support a model in which EPHA2 signaling promotes PP2A-dependent ECSIT dephosphorylation. Nonetheless, we agree that these experiments do not yet demonstrate a direct physical interaction between EPHA2 and PP2A. We have therefore revised the text and model to avoid claiming direct EPHA2 to PP2A binding or direct enzymatic activation. Furthermore, we explicitly state in the Discussion that the molecular mechanism underlying EPHA2-mediated modulation of PP2A activity is not fully resolved and warrants further investigation (*page 10, lines 324-330*).

2) The NAD^+ supplementation experiment is compelling but broad in interpretation. The conclusion that NAD^+ acts via Complex I-dependent regeneration could be overstated. NAD^+ also affects PARPs, sirtuins, and nucleotide biosynthesis, which may contribute to the observed effects. It would be important to clarify whether rescue occurs via mitochondrial NAD^+/NADH redox balance or more global NAD^+-dependent processes. Including direct measurements of NAD^+/NADH in vivo, or partial rescue with NADH-insensitive analogs, could help delineate specificity. Also, testing whether mitochondrially targeted NAD^+ precursors (e.g., NMN vs. NR) produce differential rescue outcomes would clarify whether the effect is indeed mitochondrial.

We thank the reviewer for this important point. We agree that the rescue observed after maternal NAD^+ supplementation should not be interpreted as proof that the phenotype is

corrected exclusively through Complex I-dependent mitochondrial NAD⁺ regeneration, because NAD⁺ can also influence broader NAD⁺-dependent pathways. We have therefore tempered our interpretation and added new experiments that address the mitochondrial component more directly.

First, after maternal NAD⁺ administration, mitochondria isolated from embryonic brains showed increased Complex I-dependent NADH oxidation activity relative to saline controls (**revised Fig. 4D**). We now describe this result as a biochemical readout of mitochondrial NADH oxidation capacity, rather than as a direct absolute measurement of mitochondrial NAD⁺/NADH ratio.

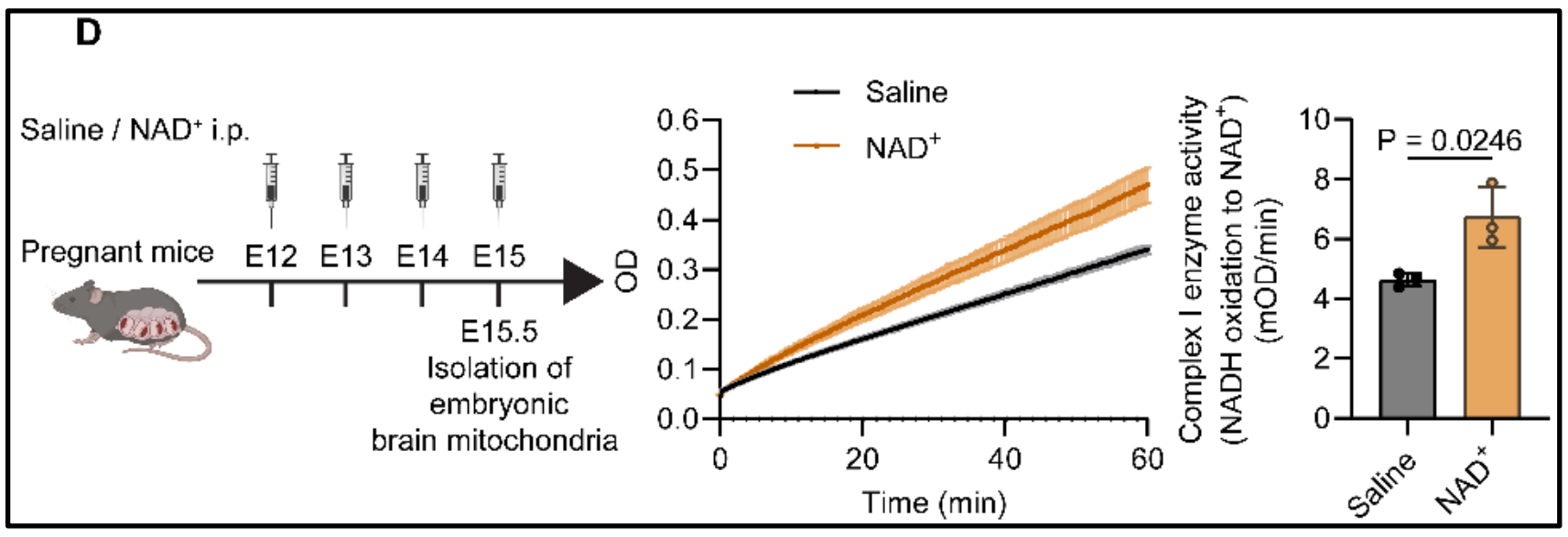

Second, NMN supplementation reproduced the rescue of the EPHA2 5A laminar distribution phenotype and normalized it to the EPHA2 WT range (**revised Fig. 4E, F**). This indicates that the rescue is not unique to exogenous NAD⁺ itself. NMN was selected over NR for testing based on evidence that it can access and accumulate within mitochondria, leading to increased mitochondrial NAD⁺/NADH ratio (Hu *et al*, 2021; Stein & Imai, 2012).

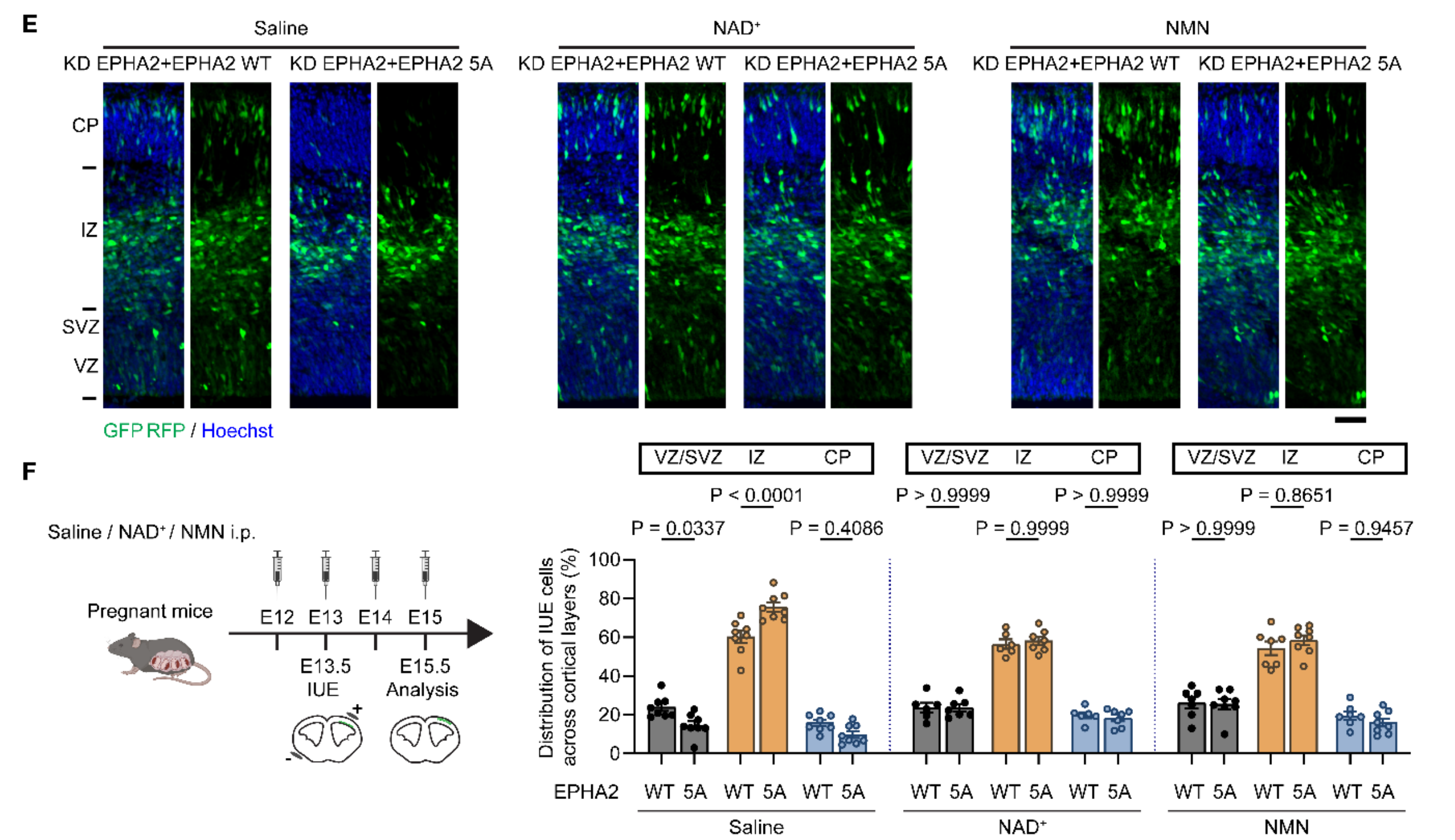

Figure 4

(D–F) Saline or NAD⁺ (10 mg in 100 µL saline) or NMN (500 mg/kg) was intraperitoneally injected daily into pregnant dams from E12 to E15, following IUE of either EGFP-KD EPHA2 + RFP-EPHA2 WT or EGFP-KD EPHA2 + RFP-EPHA2 5A at E13.5. Analysis at E15.5.

(D) Complex I enzyme activity in mitochondria isolated from embryonic brains after maternal saline or NAD⁺ treatment. mOD/min, millioptical density per minute.

(E) GFP RFP electroporated cells are shown relative to cortical layers defined by Hoechst density. Scale bar, 50 μm.

(F) Quantification of the distribution of electroporated cells across VZ/SVZ, IZ, and CP under the indicated conditions.

Third, selective enhancement of mitochondrial NAD^+ regeneration by MTS-*Lb*NOX also rescued the EPHA2 5A phenotype, providing an independent genetic argument for the importance of mitochondrial redox control (response to Comment #3).

Taken together, these new data support a substantial contribution of mitochondrial NADH oxidation and redox balance to the rescue phenotype. However, we acknowledge that NAD^+ and NMN supplementation may also influence broader NAD^+-dependent pathways, including PARPs and sirtuins. The ETC recycles mitochondrial NADH to NAD^+, with a secondary oxidation of cytosolic NADH via shuttles, thereby could potentially influencing global NAD^+-dependent pathways (Titov *et al*, 2016). Therefore, although our data are consistent with a model in which improved mitochondrial redox and Complex I function contribute to the rescue, we cannot exclude contributions from broader NAD^+-dependent processes. We now explicitly expand the Discussion to state that: “Further work is needed to elucidate how mitochondrial NAD^+/NADH balance or broader NAD^+-dependent pathways influence NSPC behavior” (*page 10, lines 311-312*).

3) The authors convincingly show decreased Complex I activity and oxygen consumption in EPHA2 5A cells, but these data stop short of demonstrating that Complex I-mediated NAD^+ regeneration is the causal determinant of NSPC proliferation. It would be important to assess whether pharmacological inhibition of Complex I (ex. rotenone) phenocopies EPHA2 loss in NSPCs and whether alternative NAD^+-regenerating pathways (e.g., NDI1 expression) can rescue proliferation. Measurements of total cellular and mitochondrial NAD^+/NADH ratios would strengthen the claim beyond sensor-based imaging.

We agree that reduced respiration in the cell line alone was not sufficient to establish causality. To address this point, we have added pharmacological, genetic, and biochemical experiments that directly test the role of Complex I-linked mitochondrial NAD^+ regeneration in NSPC maintenance.

First, low-dose rotenone treatment in primary NSPCs phenocopied the EPHA2 perturbation phenotype. After 48 hours, rotenone significantly reduced the proportion of Ki67$^+$, PH3$^+$, and SOX2$^+$ cells, indicating impaired progenitor proliferation and maintenance (**revised Fig. EV7D–G**).

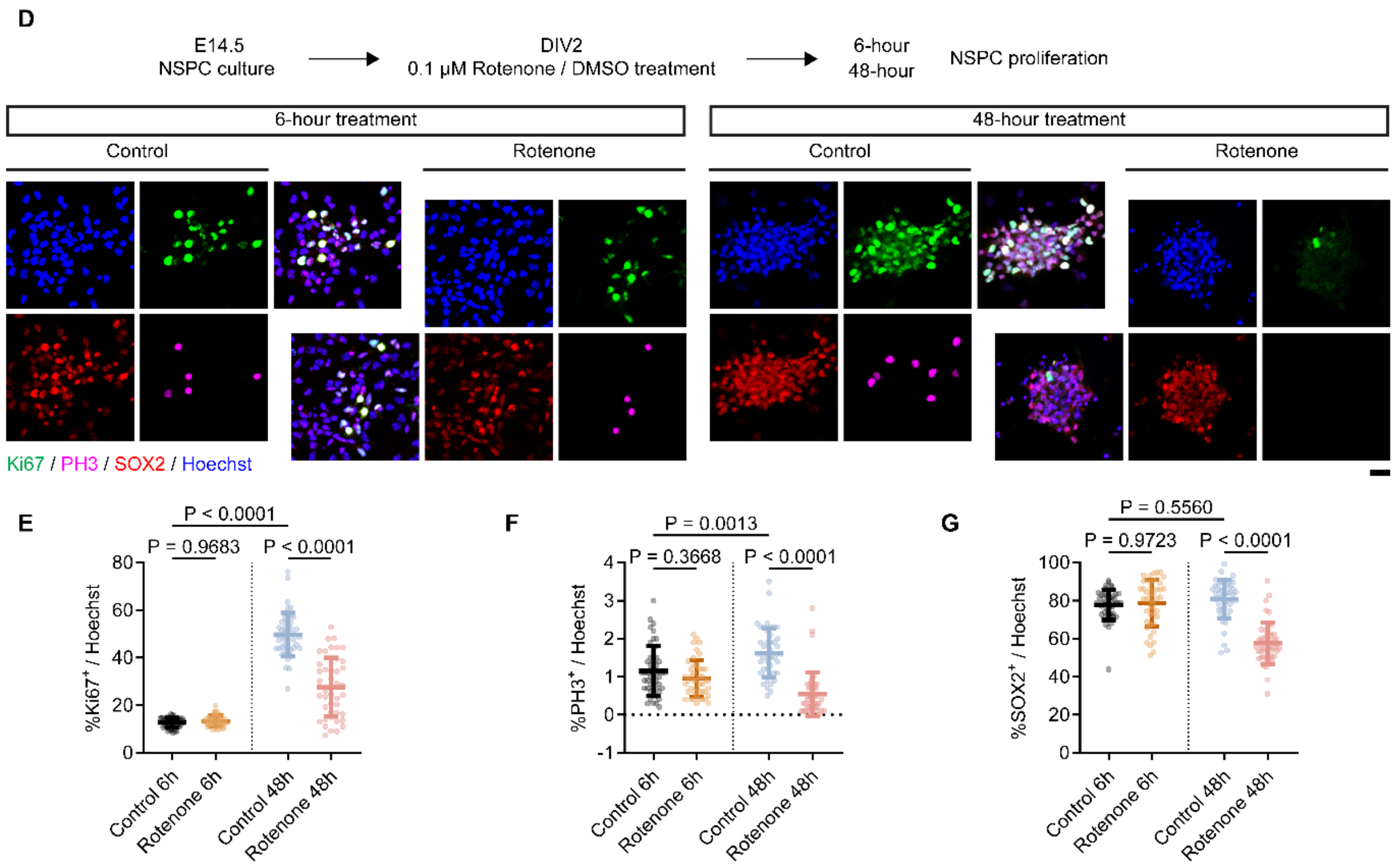

Figure EV7

(D) Representative images of primary NSPCs treated with vehicle or low-dose Rotenone and stained for proliferation markers. For each condition, 45 randomly selected microscopic fields were acquired. Scale bar, 20 µm.

(E) Quantification of Ki67-positive NSPCs after Rotenone treatment.

(F) Quantification of PH3-positive NSPCs after Rotenone treatment.

(G) Quantification of SOX2-positive NSPCs after Rotenone treatment.

Second, we tested whether alternative mitochondrial NAD^+-regenerating mechanisms could rescue the phenotype *in vivo*. MTS-*Lb*NOX, which selectively oxidizes mitochondrial NADH to NAD^+ without feeding electrons into the respiratory chain, restored the distribution of EPHA2 5A-expressing cells to the EPHA2 WT range (**revised Fig. 4A, B**). By contrast, Ndi1, which bypasses Complex I by oxidizing NADH and transferring electrons directly to CoQ, did not rescue and instead shifted cells toward the cortical plate (**revised Fig. 4A, B**). This may reflect the ability of Ndi1 to support mitochondrial ATP production, a process associated with enhanced differentiation (Lisowski *et al*, 2018). Thus, selective restoration of mitochondrial NAD^+ regeneration is sufficient for rescue, whereas bypassing Complex I with altered ETC output does not recapitulate the effect in this developmental context.

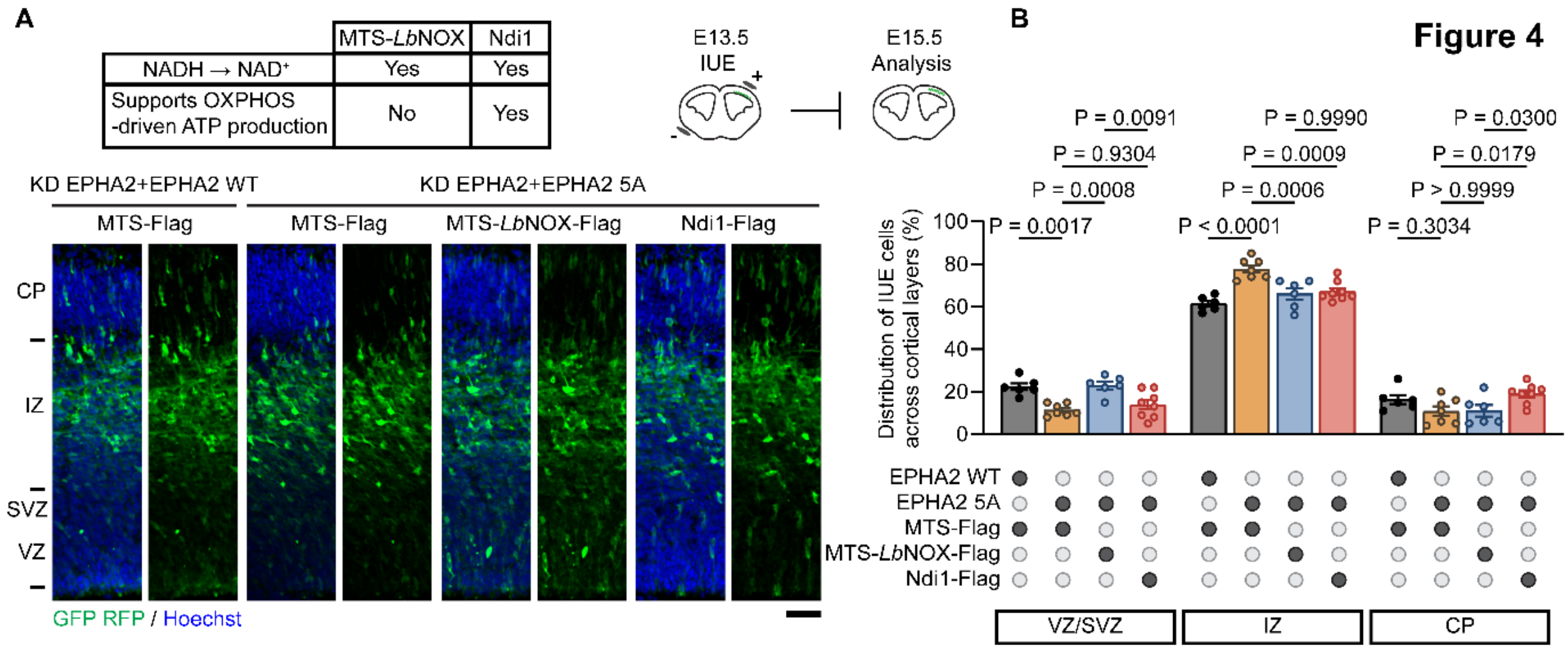

Figure 4

(A, B) Functional comparison of MTS-*Lb*NOX and Ndi1 as tools to restore mitochondrial NAD^+ regeneration. IUE at E13.5 with EGFP-KD EPHA2 + RFP-EPHA2 WT, EGFP-KD EPHA2 + RFP-EPHA2 5A together with either MTS-Flag, MTS-*Lb*NOX-Flag, or Ndi1-Flag. Analysis at E15.5.

(A) GFP RFP electroporated cells are shown relative to cortical layers defined by Hoechst density. Scale bar, 50 µm.

(B) Quantification of the distribution of electroporated cells across VZ/SVZ, IZ, and CP under the indicated conditions.

Third, we added an orthogonal biochemical assay using mitochondria isolated from embryonic brains. EPHA2 5A reduced Complex I-dependent NADH oxidation activity relative to EPHA2 WT (**revised Fig. 3J**), and maternal NAD^+ supplementation increased the same readout *ex vivo* (**revised Fig. 4D**). These data complement the sensor-based measurements and support the conclusion that EPHA2 regulates NSPC behavior through a mitochondrial Complex I/NAD^+ regeneration axis.

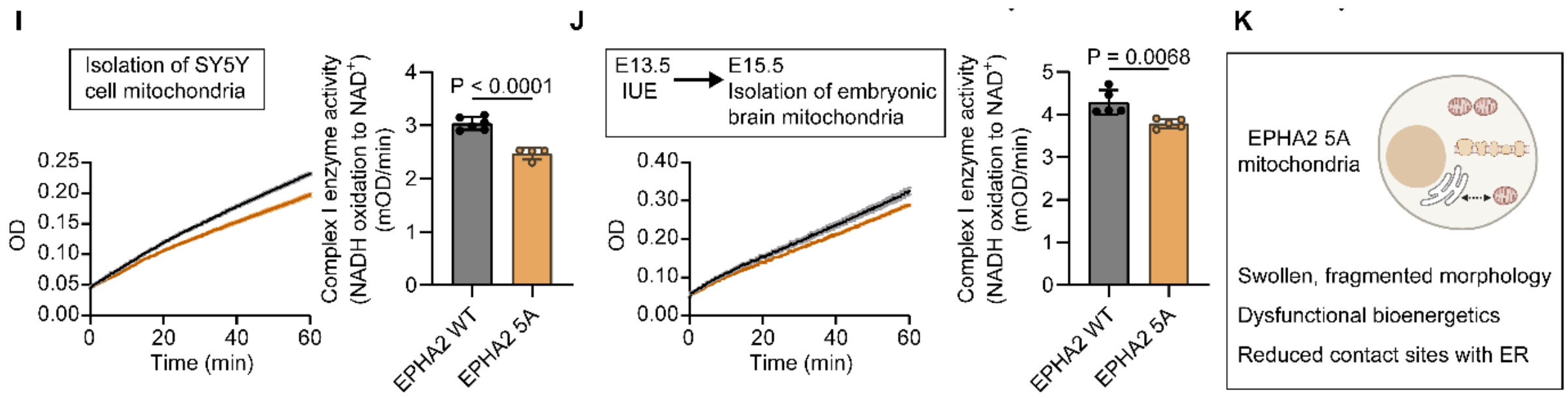

Figure 3

(I) Complex I enzyme activity in mitochondria isolated from inducible EPHA2 WT and EPHA2 5A SH-SY5Y cells. mOD/min, millioptical density per minute.

(J) Complex I enzyme activity in mitochondria isolated from embryonic brains expressing EPHA2 WT or EPHA2 5A. mOD/min, millioptical density per minute.

(K) Schematic of mitochondrial defects in EPHA2 5A cells. Illustrations created with BioRender.com.

We agree that absolute measurements of total cellular and mitochondrial NAD^+/NADH pools would further strengthen this point. We did not obtain those measurements in the present revision, and we have therefore tempered the manuscript accordingly. Our revised conclusion is that mitochondrial Complex I-dependent NAD^+ regeneration is a key downstream effector of EPHA2 signaling in NSPCs, rather than claiming that it is the only mechanism involved (*page 9, lines 304-308* and *page 10, lines 309-311*).

4) The EM images show "fragmented and swollen mitochondria," but quantitative morphometric analysis is needed to confirm these qualitative observations. Inclusion of mitophagy markers (e.g., PINK1, LC3-II, p62) would clarify whether mitochondrial turnover is altered.

We thank the reviewer for this helpful suggestion. In the **original Fig. 3A** (now **revised Fig. EV5D**), the EM dataset had already been quantified for mitochondrial number per cell, perimeter, area, and aspect ratio, and the description of “fragmented and swollen mitochondria” was based on these morphometric analyses rather than on qualitative inspection.

To further address mitochondrial turnover, we added LC3-based analyses. Under basal conditions, LC3B-II levels in EPHA2 5A cells showed a modest downward trend relative to EPHA2 WT (**revised Fig. EV5G**), and we therefore do not rely on this result alone. We then performed a damage-induced assay using CCCP and quantified LC3B recruitment to TOM20-positive mitochondria. Under these conditions, EPHA2 5A cells showed significantly reduced mitochondrial LC3B puncta compared with EPHA2 WT (**revised Fig. EV5H**). We interpret these data more cautiously as evidence for an altered mitophagy-related response after mitochondrial damage, rather than a definitive block in mitochondrial turnover. We have revised the manuscript accordingly (*page 6, lines 141-142*).

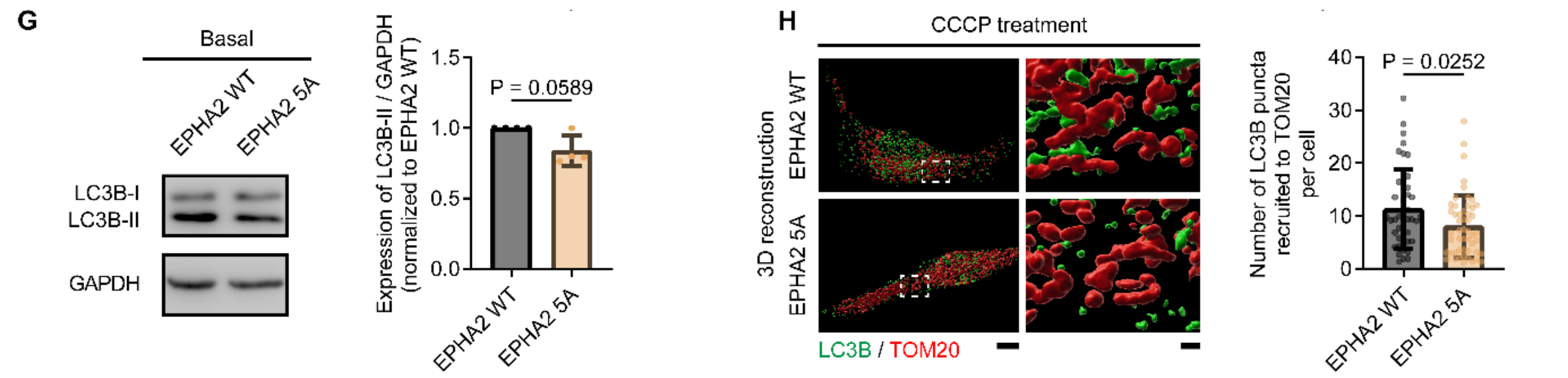

Figure EV5

(G) Basal LC3B-II immunoblot analysis in inducible EPHA2 WT and EPHA2 5A cells.

(H) CCCP-induced mitophagy assay showing LC3B puncta co-localization with TOM20. Boxed areas denote zoomed areas. Cells were reconstructed using Imaris software. Scale bars, 10 μm (left), 1 μm (right).

5) The reduction in MAM-BiFC signal and Ca^{2+} uptake is intriguing, yet the causal link between MAM disruption and progenitor proliferation remains speculative. Are these changes secondary to Complex I dysfunction, or do they occur upstream? Functional validation using artificial tether constructs (e.g., synthetic MAM linker expression) could reveal whether restoring ER-mitochondrial contacts rescues metabolic or proliferative defects.

We thank the reviewer for this important point. We agree that the original manuscript did not establish whether the MAM phenotype lies upstream or downstream of the metabolic defect. To address this, we performed two additional experiments.

First, we tested whether pharmacological Complex I inhibition affects ER-mitochondria contacts (MAMs) in primary NSPCs. Acute inhibition of Complex I in primary NSPCs using low-dose rotenone reduced MAM integrity as early as 6 hours post-treatment, a time point at which NSPC proliferation remained unchanged. By 48 hours, both MAM integrity and NSPC proliferation were significantly reduced (**revised Fig. EV7D–G** response to Comment #3 and **Fig. EV8C, D**). These data show that Complex I dysfunction is accompanied by loss of ER-mitochondria contacts, before the onset of the progenitor phenotype.

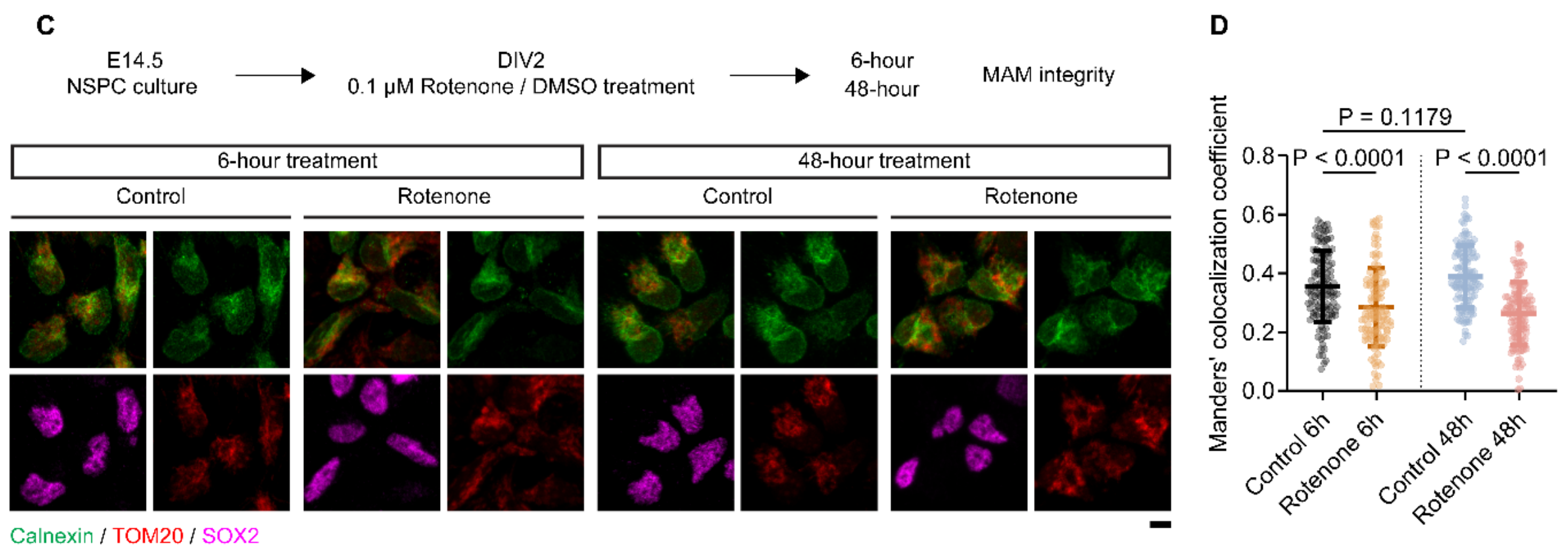

Figure EV8

(C) Representative images assessing ER–mitochondria contacts using ER marker Calnexin and mitochondrial marker TOM20 after Rotenone treatment in primary NSPCs. Scale bar, 5 µm.

(D) Quantification of MAM integrity, assessed by co-localization between Calnexin and TOM20 after Rotenone treatment.

Second, we re-expressed an artificial OMM-ER tether in EPHA2 5A NSPCs. Enforced tethering did not normalize the mt-SoNar readout (**revised Fig. EV9A**), indicating that restoration of ER-mitochondria contacts alone is not sufficient to rescue the mitochondrial redox defect. Interestingly, it increased the retention of electroporated cells within the VZ/SVZ, with minimal migration to the CP (**revised Fig. EV9B, C**).

Together, these data suggest that reduced ER-mitochondria contacts in EPHA2 5A are not the primary cause of the mitochondrial NAD^+/NADH defect but may contribute to the progenitor distribution phenotype through a partially separable mechanism, warranting further investigation. We have revised the Results and Discussion to reflect this notion (*page 7, lines 193-209* and *page 10, lines 334-336*).

Figure EV9

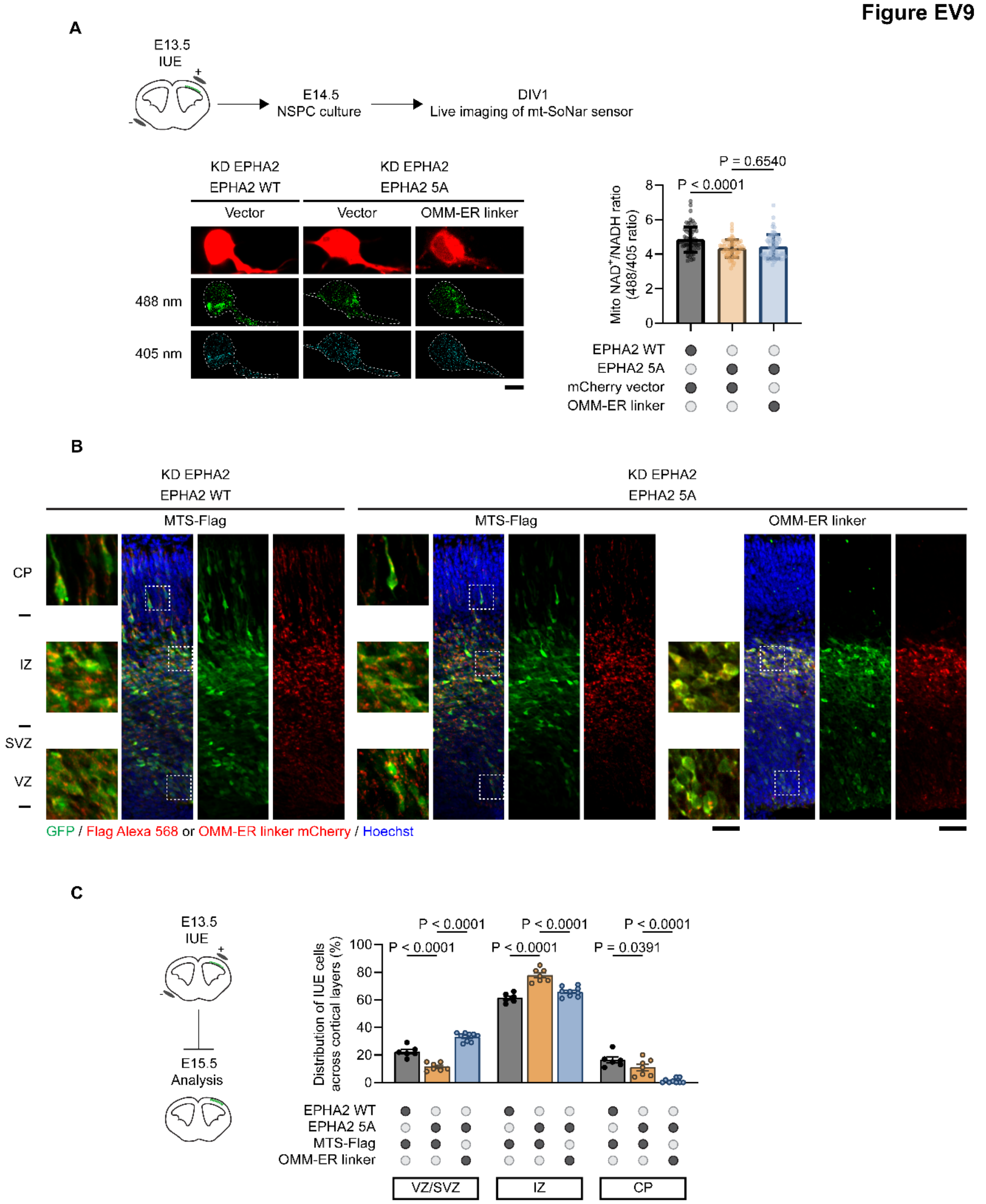

Figure EV9

(A) Mitochondrial NAD⁺/NADH readout assessed by mt-SoNar sensor in primary NSPCs expressing EPHA2 WT or EPHA2 5A with or without an artificial OMM–ER linker. Scale bar, 5 µm.

(B, C) IUE at E13.5 with EGFP-KD EPHA2 + EGFP-EPHA2 WT or EGFP-KD EPHA2 + EGFP-EPHA2 5A together with either MTS-Flag or OMM–ER linker. Analysis at E15.5.

(B) GFP RFP electroporated cells are shown relative to cortical layers defined by Hoechst density. Boxed areas denote zoomed areas. Scale bars, 20 µm (left), 50 µm (right).

(C) Quantification of the distribution of electroporated cells across VZ/SVZ, IZ, and CP under the indicated conditions.

6) The data suggest a mild OXPHOS impairment, but embryonic NSPCs are generally glycolytic. It would strengthen the metabolic argument to measure glycolytic flux (ECAR) and ATP source partitioning (glycolysis vs. OXPHOS).

We thank the reviewer for this critical suggestion. To strengthen the metabolic analysis in the relevant cell type, we now measured both mitochondrial respiration and glycolytic flux in primary embryonic NSPCs expressing EPHA2 WT or EPHA2 5A. We found that EPHA2 5A significantly reduced both basal respiration and basal glycolysis relative to EPHA2 WT (**revised Fig. 3F–H**). Thus, the metabolic phenotype in primary NSPCs is not restricted to OXPHOS alone. One possible explanation is that impaired mitochondrial NAD^+ regeneration disrupts cellular redox homeostasis, which may secondarily limit glycolytic flux. In particular, accumulation of mitochondrial NADH and reduced NAD^+ availability could constrain NADH shuttle activity, thereby limiting cytosolic NAD^+ regeneration required for NAD^+-dependent steps in glycolysis (*e.g.*, GAPDH). Consistent with this notion, a similar reduction in both mitochondrial respiration and glycolysis has been reported in cells lacking SLC25A51, a mitochondrial NAD^+ transporter that regulates mitochondrial NAD^+/NADH balance (Lu *et al*, 2024).

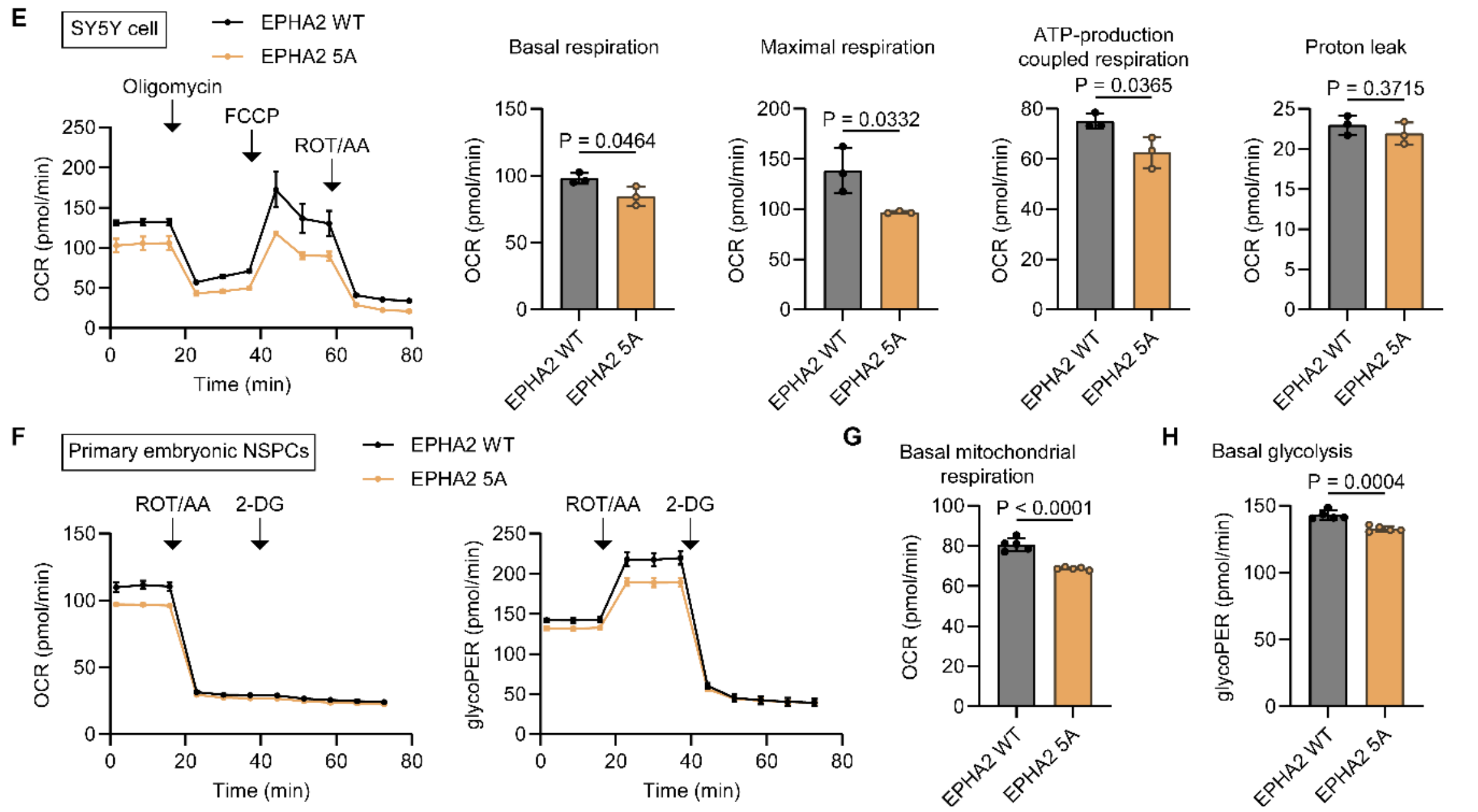

[**Figure 3**]

(E) Seahorse analysis of oxygen consumption (Mito Stress Test) in inducible EPHA2 WT and EPHA2 5A SH-SY5Y cells, with quantification of basal, maximal, ATP-linked respiration, and proton leak.

(F) Seahorse analysis of oxygen consumption and proton efflux rate (Glycolytic Rate Assay) in primary NSPCs expressing EPHA2 WT or EPHA2 5A.

(G) Quantification of basal mitochondrial respiration in primary NSPCs.

(H) Quantification of basal glycolytic activity in primary NSPCs.

The concurrent reduction in mitochondrial respiration and glycolytic flux in NSPCs, together with reduced ATP-linked respiration in the cell line (**revised Fig. 3E**), supports the conclusion that EPHA2 perturbation broadly suppresses cellular bioenergetic activity, rather

than inducing compensatory glycolysis. This is consistent with the observed reduction in progenitor proliferative capacity in EPHA2 5A condition. Because we did not perform a formal ATP rate assay in primary NSPCs, we limit our conclusion in the revised manuscript to the directly measured findings, namely that loss of ligand-independent EPHA2 signaling suppresses both oxidative and glycolytic metabolism.

7) The proteomic dataset is a valuable resource, yet the focus is largely on Complex I subunits. Additional analysis of mitochondrial chaperones, assembly factors, and redox enzymes could reveal whether a broader reorganization of the mitochondrial proteome occurs.

We thank the reviewer for this valuable suggestion.

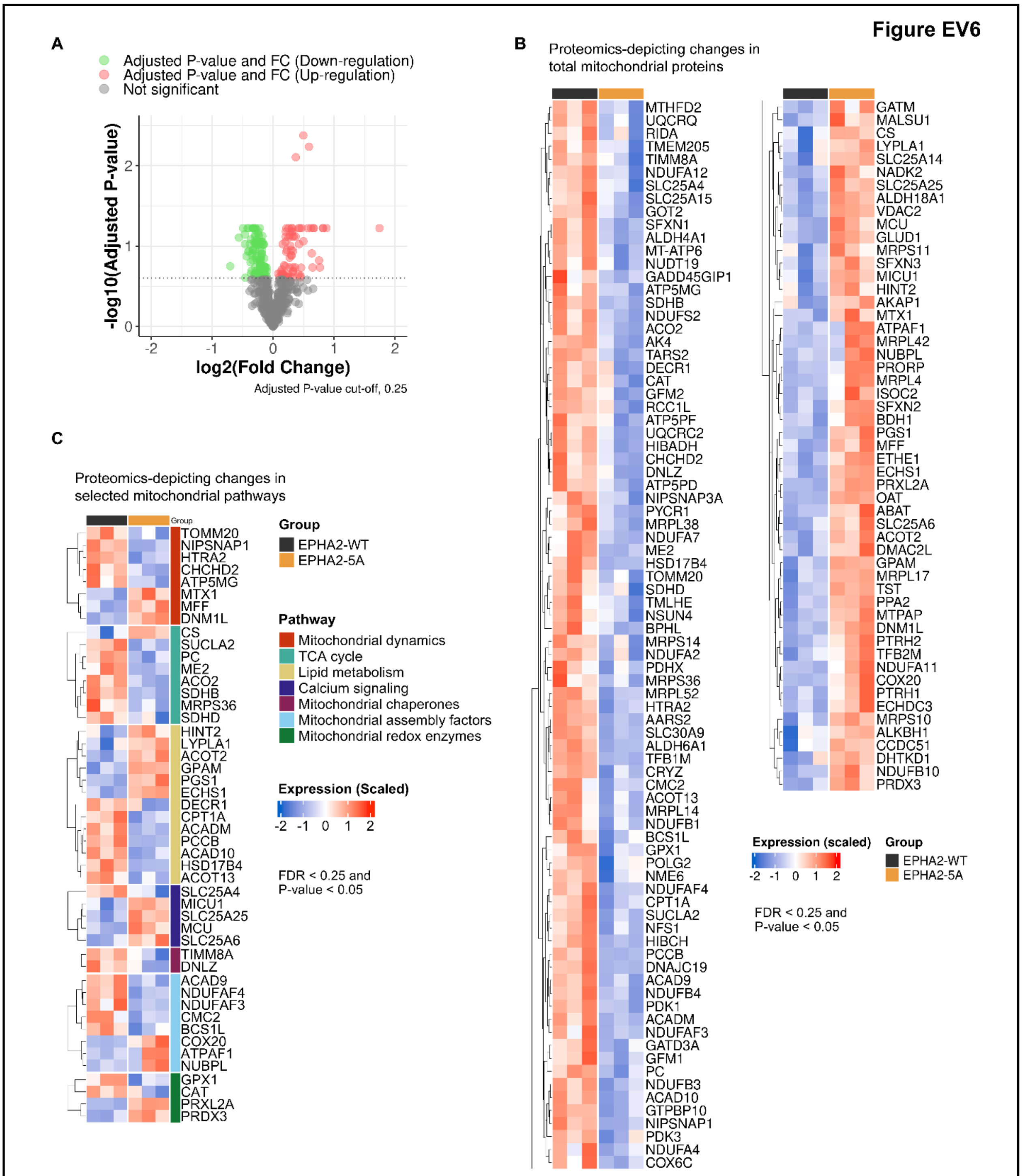

Figure EV6

Figure EV6

(A) Volcano plot of differentially abundant mitochondrial proteins in inducible EPHA2 WT and EPHA2 5A cells.

(B) Heatmap of significantly altered mitochondrial proteins.

(C) Analysis of selected mitochondrial pathways revealed that mitochondrial dynamics, TCA cycle, and calcium signaling were also sensitive to disruption of ligand-independent EPHA2 signaling.

We agree that the proteomic dataset should be interpreted more broadly than ETC or Complex I subunits alone. In the revised manuscript, we therefore expanded the analysis to include additional mitochondrial pathways such as mitochondrial dynamics, TCA cycle, lipid metabolism, calcium signaling, chaperones, assembly factors, and redox enzymes. **Revised Fig. EV6A, B** now show the overall distribution of significantly altered mitochondrial proteins, and **revised Fig. EV6C** highlights changes in representative pathways. Statistical thresholds are now indicated explicitly in the figures, FDR < 0.25 and P < 0.05.

These additions indicate that EPHA2 5A is associated with broader mitochondrial proteome remodeling, rather than an isolated change in a small subset of Complex I subunits.

8) SH-SY5Y cells are a convenient system but metabolically distinct from primary NSPCs. Studies must be confirmed in primary NSPCs.

We appreciate the reviewer's point and agree that the central conclusions should be supported in primary NSPCs and embryonic brain tissue, not only in SH-SY5Y cells. In the revised manuscript, we therefore highlight the findings that were independently validated beyond the cell line system. ECSIT mitochondrial co-localization was confirmed in primary NSPCs (**revised Fig. 5D**). The metabolic phenotype was reproduced in primary NSPCs, where EPHA2 5A reduced both basal respiration and basal glycolysis (**revised Fig. 3F–H**). In addition, Complex I activity was measured in mitochondria isolated from embryonic brain tissue (**revised Fig. 3J**), and MAM or redox-related analyses were extended to primary NSPC and *in vivo* IUE paradigms (**revised Fig. EV8** and **Fig. EV9**).

We have revised the Discussion to state explicitly that SH-SY5Y is used here as a tractable mechanistic system, whereas the core metabolic and developmental conclusions are supported by primary NSPC and embryonic brain data. At the same time, we acknowledge that some biochemical mapping experiments remain cell line-based for technical reasons, and we now note this limitation explicitly (*page 9, lines 288-290*).

References

Anandatheerthavarada HK, Biswas G, Mullick J, Sepuri NB, Otvos L, Pain D, Avadhani NG (1999) Dual targeting of cytochrome P4502B1 to endoplasmic reticulum and mitochondria involves a novel signal activation by cyclic AMP-dependent phosphorylation at ser128. *Embo j* 18: 5494-5504

Bilan DS, Matlashov ME, Gorokhovatsky AY, Schultz C, Enikolopov G, Belousov VV (2014) Genetically encoded fluorescent indicator for imaging NAD(+)/NADH ratio changes in different cellular compartments. *Biochim Biophys Acta* 1840: 951-957

Hu Q, Wu D, Walker M, Wang P, Tian R, Wang W (2021) Genetically encoded biosensors for evaluating NAD(+)/NADH ratio in cytosolic and mitochondrial compartments. *Cell Rep Methods* 1

Law YS, Zhang R, Guan X, Cheng S, Sun F, Duncan O, Murcha MW, Whelan J, Lim BL (2015) Phosphorylation and Dephosphorylation of the Presequence of Precursor MULTIPLE ORGANELLAR RNA EDITING FACTOR3 during Import into Mitochondria from Arabidopsis. *Plant Physiol* 169: 1344-1355

Lee J, O'Neill RC, Park MW, Gravel M, Braun PE (2006) Mitochondrial localization of CNP2 is regulated by phosphorylation of the N-terminal targeting signal by PKC: implications of a mitochondrial function for CNP2 in glial and non-glial cells. *Mol Cell Neurosci* 31: 446-462

Lisowski P, Kannan P, Mlody B, Prigione A (2018) Mitochondria and the dynamic control of stem cell homeostasis. *EMBO Rep* 19

Lu MJ, Busquets J, Impedovo V, Wilson CN, Chan HR, Chang YT, Matsui W, Tiziani S, Cambronne XA (2024) SLC25A51 decouples the mitochondrial NAD(+)/NADH ratio to control proliferation of AML cells. *Cell Metab* 36: 808-821.e806

Naher S, Iemura K, Miyashita S, Hoshino M, Tanaka K, Niwa S, Tsai JW, Kikkawa T, Osumi N (2025) Kinesin-like motor protein KIF23 maintains neural stem and progenitor cell pools in the developing cortex. *Embo j* 44: 331-355

Nicol T, Falcone S, Blease A, Vikhe P, Civiletto G, Omairi SS, Viscomi C, Patel K, Potter PK (2023) Tissue-specific differences in the assembly of mitochondrial Complex I are revealed by a novel ENU mutation in ECSIT. *Cardiovasc Res* 119: 2213-2229

Rao S, Schmidt O, Harbauer AB, Schönfisch B, Guiard B, Pfanner N, Meisinger C (2012) Biogenesis of the preprotein translocase of the outer mitochondrial membrane: protein kinase A phosphorylates the precursor of Tom40 and impairs its import. *Mol Biol Cell* 23: 1618-1627

Robin MA, Prabu SK, Raza H, Anandatheerthavarada HK, Avadhani NG (2003) Phosphorylation enhances mitochondrial targeting of GSTA4-4 through increased affinity for binding to cytoplasmic Hsp70. *J Biol Chem* 278: 18960-18970

Schmidt O, Harbauer AB, Rao S, Eyrich B, Zahedi RP, Stojanovski D, Schönfisch B, Guiard B, Sickmann A, Pfanner N *et al* (2011) Regulation of mitochondrial protein import by cytosolic kinases. *Cell* 144: 227-239

Stein LR, Imai S (2012) The dynamic regulation of NAD metabolism in mitochondria. *Trends Endocrinol Metab* 23: 420-428

Timón-Gómez A, Pérez-Pérez R, Nyvltova E, Ugalde C, Fontanesi F, Barrientos A (2020) Protocol for the Analysis of Yeast and Human Mitochondrial Respiratory Chain Complexes and Supercomplexes by Blue Native Electrophoresis. *STAR Protoc* 1

Titov DV, Cracan V, Goodman RP, Peng J, Grabarek Z, Mootha VK (2016) Complementation of mitochondrial electron transport chain by manipulation of the NAD+/NADH ratio. *Science* 352: 231-235

Vogel RO, Janssen RJ, van den Brand MA, Dieteren CE, Verkaart S, Koopman WJ, Willems PH, Pluk W, van den Heuvel LP, Smeitink JA *et al* (2007) Cytosolic signaling protein Ecsit also localizes to mitochondria where it interacts with chaperone NDUFAF1 and functions in complex I assembly. *Genes Dev* 21: 615-624

Xiao C, Shim JH, Klüppel M, Zhang SS, Dong C, Flavell RA, Fu XY, Wrana JL, Hogan BL, Ghosh S (2003) Ecsit is required for Bmp signaling and mesoderm formation during mouse embryogenesis. *Genes Dev* 17: 2933-2949

Dear Prof. Park,

Thank you for the submission of your revised manuscript. We have now received the enclosed reports from the referees and I am happy to say that both support its publication now. Referee 1 has also assessed your response to referee 2's comments, as this referee could not be reached. Referee 1 still has a few more minor suggestions that I would like you to incorporate before we can proceed with the official acceptance of your manuscript. Please submit a point-by-point response to all comments with your final ms.

A few editorial requests will also need to be addressed:

- Please submit the final ms as a word file.

- Please add a Data Availability Section (DAS) to the end of the Methods. This section should list all data that were generated as part of this study and deposited in public databases. If no such data were generated please mention this fact in the DAS.

- Please remove the author credits from the ms file. All credits need to be entered during online ms submission.

- Please co-submit with your final ms a completed author checklist, which you can download from our author guidelines <https://link.springer.com/journal/44319/submission-guidelines> The completed author checklist will also be part of the transparent peer-review file.

- The FUNDING INFO is missing in our online submission system. Each funder needs to be added as a separate entry and the info must be matching with the one in the ms file.

- The number of EV figures needs to be reduced to below 10. You can may be move 2 EV figures to the main figures, and also add the data in Fig EV12 to another figure?

- The Materials and Methods section should include a separate Reagents and Tools Table file (listing key reagents, experimental models, software and relevant equipment and including their sources and relevant identifiers) and a Methods and Protocols section in which methods should be described using a step-by-step protocol format with bullet points. More information and a downloadable Table template is available in our guide to authors online: https://link.springer.com/journal/44319/submission-guidelines

- Materials and Methods should be Methods

- The nomenclature of EV figure legends needs correction to Figure EV1, Figure EV2, etc. instead of Expanded View Figure 1, Expanded View Figure 2, etc.

- Our routine image analysis detected 2 possible figure panel/image reuses between Figure 2A and Figure 7C that is not listed in the figure legends. Can you please clarify ?

* Figure Legends - Comments *

1. Please note that the exact p values are not provided in the legends of 1E; 2B,F; 3G,I; 4B,F; 5C,D,E; 6A,E,G; EV-1D; EV-5C,D; EV-7A,E,F,G; EV-8D; EV-9A,C. Please provide exact values as reasonable.

2. Please indicate the statistical test used for data analysis in the legends of figures 1E; 2B,D,F; 3C,E,G-J; 4B,D,F; 5A-E,G,H; 6A,E,G,H; EV-1D,F,H; EV-2B,C,D,F; EV-3B,D,F; EV-4A,B; EV-5C-H; EV-6A-C; EV-7A,C,E,F,G; EV-8D.

3. Please note that information related to n is missing in the legends 1E; 3E-J; 6E; EV-5D,E,F,G; EV-5D-G; EV-6A; EV-7A,C,E,F,G; EV-8A,B.

I would like to suggest some minor changes to the ms title. Do you agree with:

Ligand-independent EPHA2 signaling (or activity) sustains neural progenitors via ECSIT and NAD+

EMBO press papers are accompanied online by A) a short (1-2 sentences) summary of the findings and their significance, B) 2-3 bullet points highlighting key results and C) a synopsis image that is exactly 550 pixels wide and 300 pixels high. The synopsis

image should provide a sketch of the major findings, like a graphical abstract. Please note that text needs to be readable at the final size. Please send us this information along with the final manuscript.

I look forward to seeing a final version of your manuscript as soon as possible.

Best regards,
Esther

Esther Schnapp, PhD
Senior Editor
EMBO reports

Referee #1:

The authors have adequately addressed my concerns and I suggest only a few minor changes:

-In line 67, the authors state that Epha2 expression peaked at E13.5. But this is the first time point they check, and therefore the peak of expression cannot be determined (could be earlier).
-In Figure EV5C, cells and not biological "ns" are displayed and quantified, please correct.
-The proteomics data in Figure 3 improved by removing the outlier. It could be interesting to show what drives PC1, as it seems to be the main difference between the conditions (presumably mitochondria-related genes).

Referee #3:

This manuscript is acceptable for publication in EMBO report. All issues pertaining to my have been fully addressed.

Response to the Editor

Dear Dr. Schnapp,

We sincerely thank you and the referees for the positive evaluation of our revised manuscript and for the constructive final suggestions. We are pleased to hear that both referees support publication of our manuscript. We have carefully addressed all remaining editorial requests and figure legend comments, as detailed below.

Response to editorial requests

Comment 1. Please submit the final ms as a word file.

We have submitted the final manuscript as a Word file.

Comment 2. Please add a Data Availability Section (DAS) to the end of the Methods. This section should list all data that were generated as part of this study and deposited in public databases. If no such data were generated please mention this fact in the DAS.

We have added a Data Availability Section at the end of the Methods section. This section now states that the mass spectrometry proteomics data generated in this study have been deposited in the ProteomeXchange Consortium via the jPOST partner repository under the accession codes PXD079834 for ProteomeXchange and JPST004710 for jPOST. We have also indicated that other data are available from the corresponding author upon reasonable request.

Comment 3. Please remove the author credits from the ms file. All credits need to be entered during online ms submission.

We have removed the author contribution section from the manuscript file. The author credits will be entered separately during the online manuscript submission process.

Comment 4. Please co-submit with your final ms a completed author checklist, which you can download from our author guidelines. The completed author checklist will also be part of the transparent peer-review file.

We have completed the author checklist and co-submitted it together with the final manuscript.

Comment 5. The FUNDING INFO is missing in our online submission system. Each funder needs to be added as a separate entry and the info must be matching with the one in the ms file.

We have entered the funding information in the online submission system. Each funder has been added as a separate entry, and the information has been checked to ensure that it matches the funding statement in the manuscript file.

Comment 6. The number of EV figures needs to be reduced to below 10. You can may be move 2 EV figures to the main figures, and also add the data in Fig EV12 to another figure?

We have reduced the number of EV figures to fewer than 10, as requested. Specifically, the previous Figure EV2 has been moved to main Figure 3, and the previous Figure EV5 has been moved to main Figure 4. In addition, the data from the previous Figure EV12 have been incorporated into the final Figure EV9. The final manuscript now contains 8 main figures and 9 EV figures.

Comment 7. The Materials and Methods section should include a separate Reagents and Tools Table file and a Methods and Protocols section in which methods should be described using a step-by-step protocol format with bullet points.

We have prepared and co-submitted a separate Reagents and Tools Table file listing key reagents, experimental models, software, relevant equipment, sources, and identifiers.

Comment 8. Materials and Methods should be Methods.

We have changed the section heading from “Materials and Methods” to “Methods”.

Comment 9. The nomenclature of EV figure legends needs correction to Figure EV1, Figure EV2, etc. instead of Expanded View Figure 1, Expanded View Figure 2, etc.

We have corrected the nomenclature of the EV figure legends throughout the manuscript to “Figure EV1”, “Figure EV2”, and so on.

Comment 10. Our routine image analysis detected 2 possible figure panel/image reuses between Figure 2A and Figure 7C that is not listed in the figure legends. Can you please clarify?

We thank the Editor for pointing this out. We note that our initial submission did not contain a Figure 7. After carefully rechecking the manuscript, we believe that the Editor may be referring to Figure EV1C rather than Figure 7C.

As described in the point-by-point responses to reviewers file for the previous revision process, before previous peer review, Figure EV1C showed IUE cells co-stained with Ki67 to illustrate the proliferation defects caused by EPHA2 perturbation. During the peer review process, however, we were asked to show IUE cells independently of additional staining in order to better illustrate the distribution of IUE cells across cortical layers. We therefore added Figure 2A during the revision process with same control and experimental groups.

Figure 2A before change

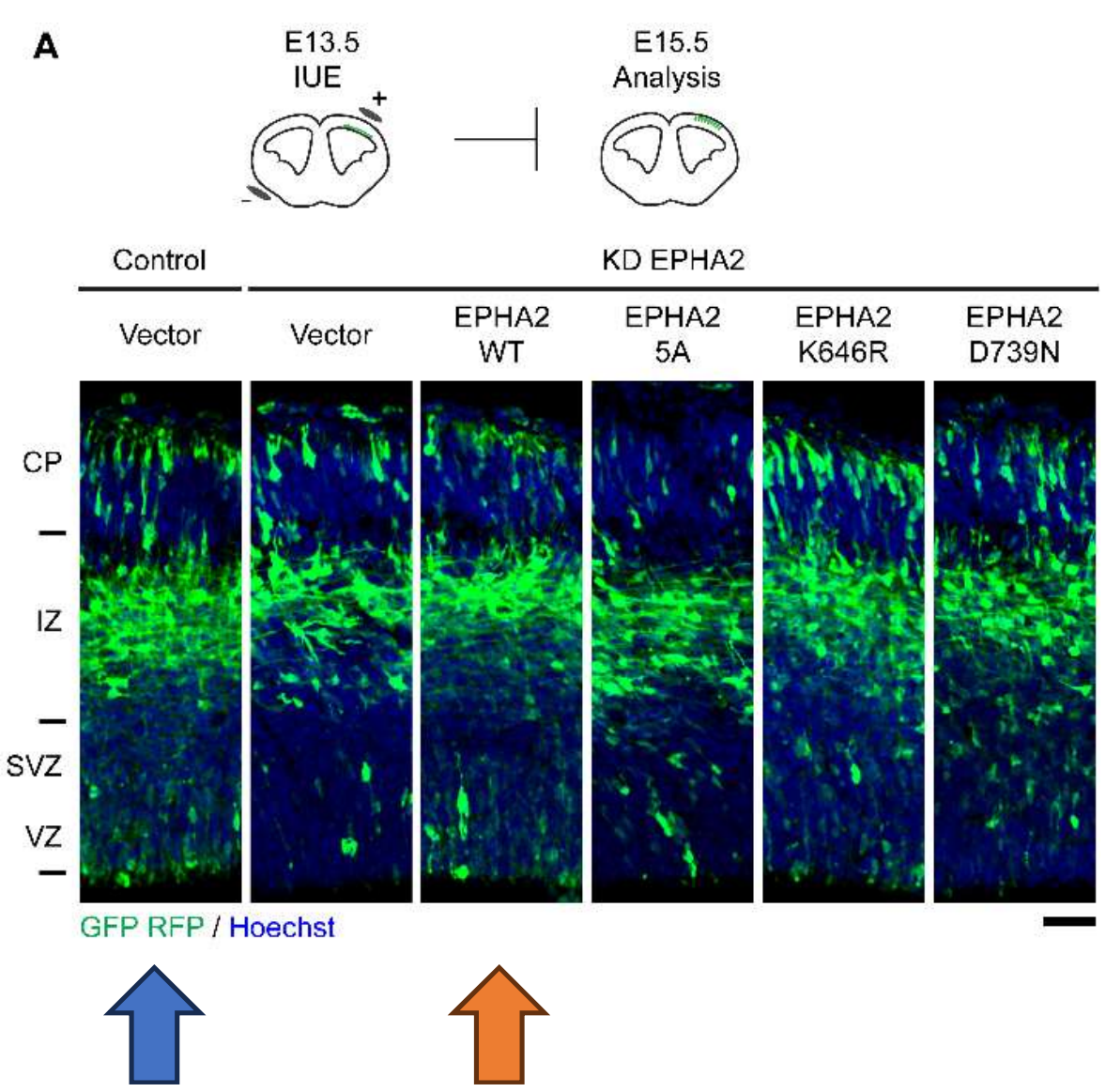

Figure EV1C

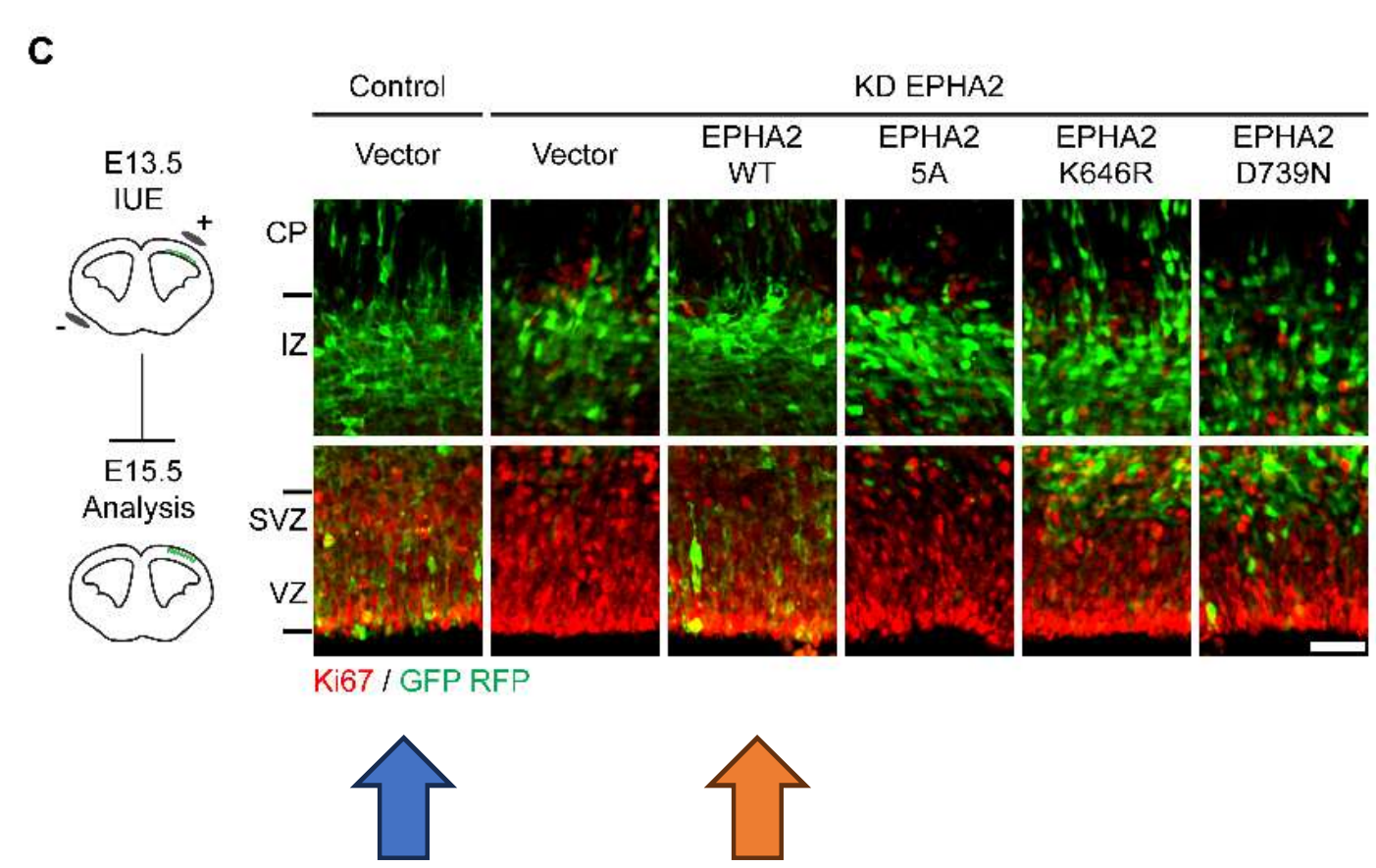

Thus, Figure 2A and Figure EV1C contained representative images from the same experimental context, but they were used to illustrate different analyses: IUE cell distribution

alone in Figure 2A and IUE cells co-stained with Ki67 in Figure EV1C. That is, the source data used for Figure 2A and EV1C are indeed identical. Anyhow, to avoid any possible confusion, we have replaced the representative images for **Control_Vector** and **KD EPHA2_EPHA2 WT rescue** in Figure 2A in the final version of the manuscript.

Figure 2A after change

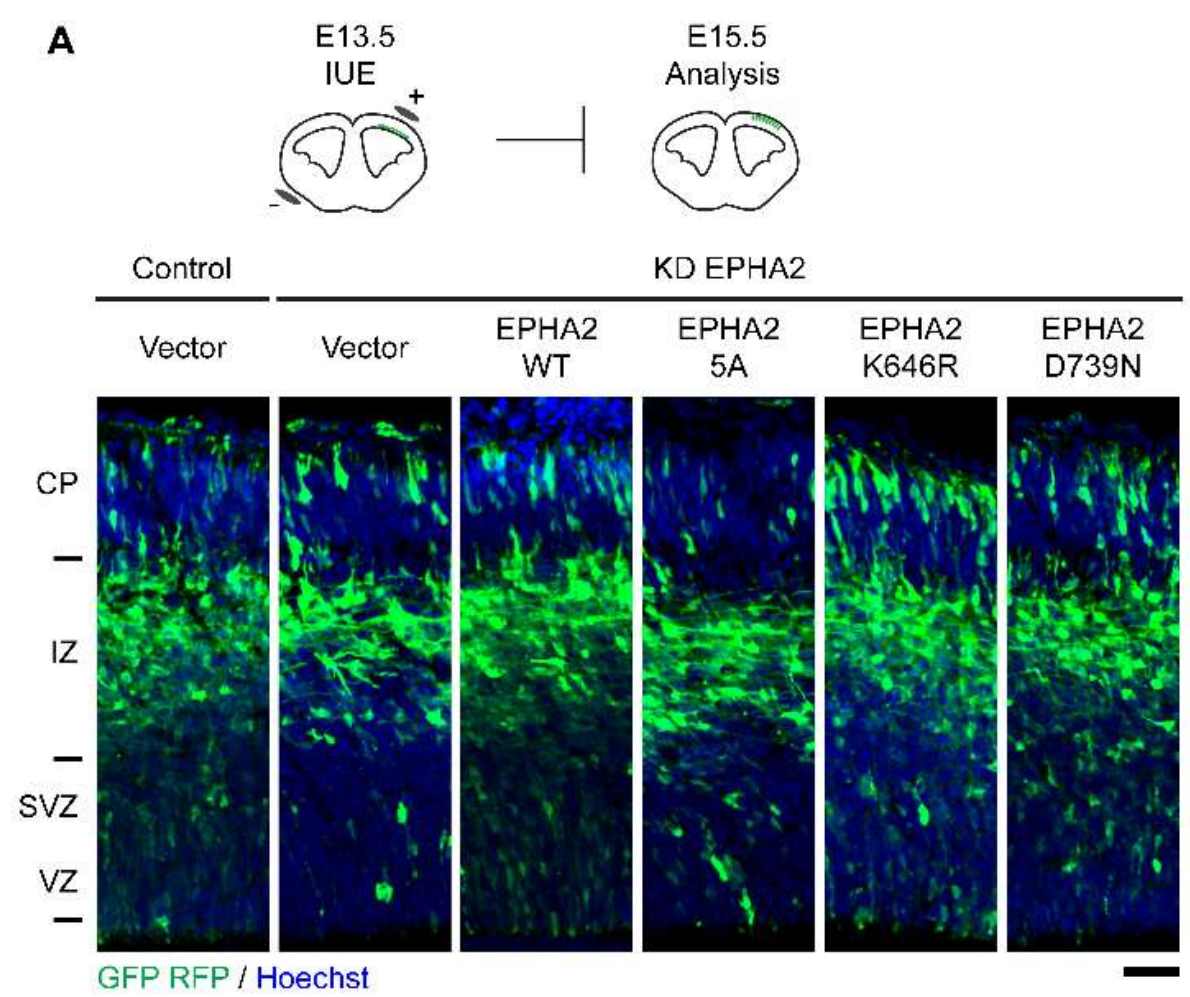

Response to Figure Legends comments

Comment 1. Please note that the exact p values are not provided in the legends of 1E; 2B,F; 3G,I; 4B,F; 5C,D,E; 6A,E,G; EV-1D; EV-5C,D; EV-7A,E,F,G; EV-8D; EV-9A,C. Please provide exact values as reasonable.

We have added the exact P-values to the indicated quantification plots, as requested. Because several plots contain multiple comparison groups, we included the exact P-values directly above the corresponding bars to ensure clarity and avoid ambiguity. In addition, we have revised the relevant figure legends to state: "Exact P-values are indicated above the bars in each quantification plot".

Comment 2. Please indicate the statistical test used for data analysis in the legends of figures 1E; 2B,D,F; 3C,E,G-J; 4B,D,F; 5A-E,G,H; 6A,E,G,H; EV-1D,F,H; EV-2B,C,D,F; EV-3B,D,F; EV-4A,B; EV-5C-H; EV-6A-C; EV-7A,C,E,F,G; EV-8D.

We have revised the indicated figure legends to specify the statistical test used for each analysis. For the previous Figure EV4A, B, which are now presented as Figure EV3A, B, the data and corresponding statistical information were curated from the published dataset reported by Li et al., 2020, rather than generated in the present study. We have clarified this point in the corresponding figure legend.

Comment 3. Please note that information related to n is missing in the legends 1E; 3E-J; 6E; EV-5D,E,F,G; EV-5D-G; EV-6A; EV-7A,C,E,F,G; EV-8A,B.

We have added the missing n information to the indicated figure legends.

Comment. I would like to suggest some minor changes to the ms title. Do you agree

with: "Ligand-independent EPHA2 signaling (or activity) sustains neural progenitors via ECSIT and NAD+"

We thank the Editor for the helpful suggestion. We agree with the suggested title revision and have changed the manuscript title to:

"Ligand-independent EPHA2 signaling sustains neural progenitors via ECSIT and NAD^+"

Comment. EMBO press papers are accompanied online by A) a short summary of the findings and their significance, B) 2-3 bullet points highlighting key results and C) a synopsis image that is exactly 550 pixels wide and 300 pixels high.

We have prepared and submitted the requested accompanying materials, including a short summary of the findings and their significance, key result bullet points, and a synopsis image.

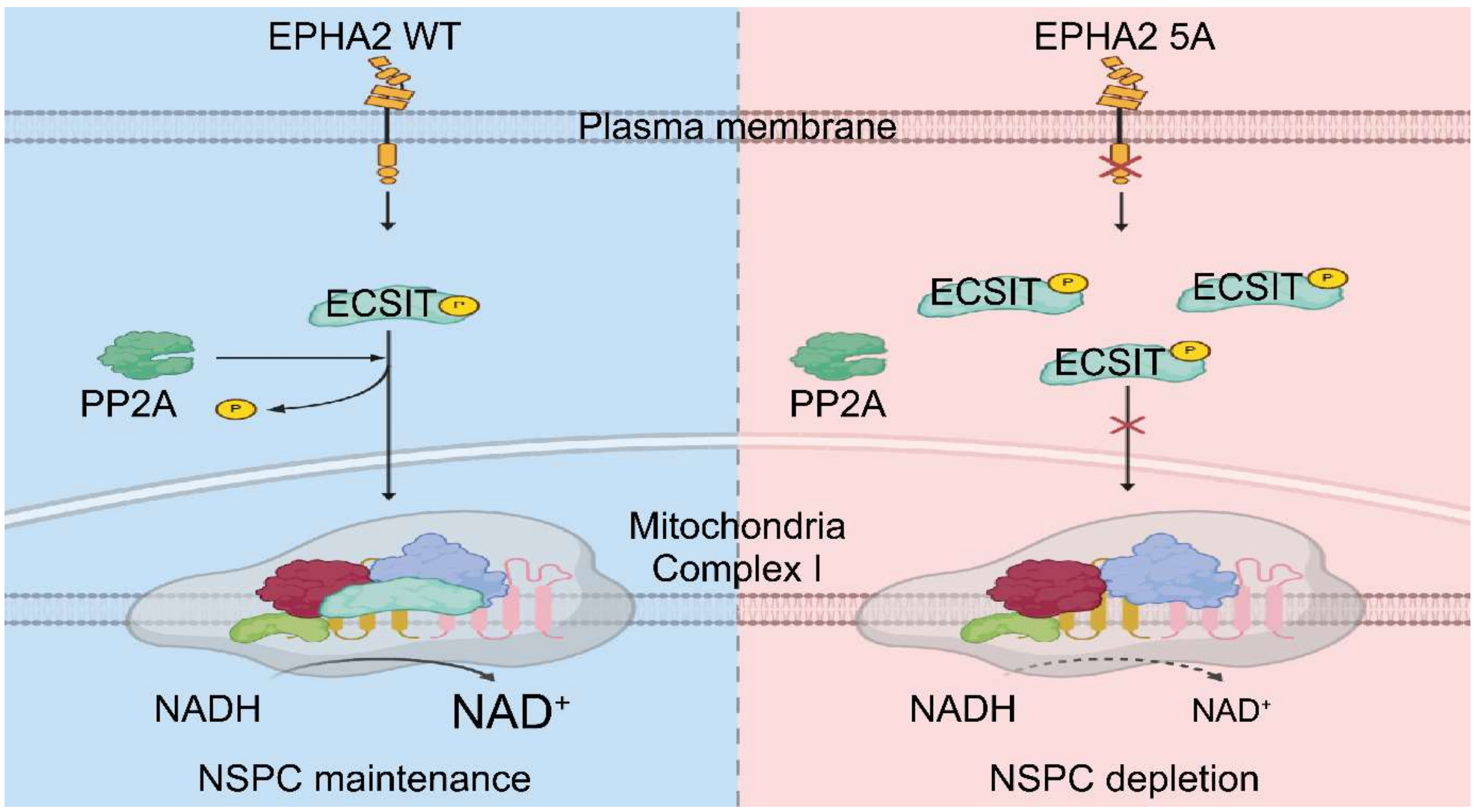

EPHA2 sustains embryonic cortical progenitor maintenance by coupling apical receptor signaling to ECSIT-dependent Complex I activity and mitochondrial NAD^+ regeneration. This provides a rare functional link between receptor state at the cell surface and mitochondrial redox control during corticogenesis.

- Ligand-independent EPHA2 signaling is required for cortical progenitor maintenance in vivo.
- EPHA2 perturbation reduces mitochondrial respiration, Complex I activity, NAD^+ regeneration, and mitochondrial ECSIT abundance.
- Restoring mitochondrial NAD^+ regeneration or mitochondrial ECSIT attenuates progenitor defects caused by EPHA2 disruption.
- A PP2A-sensitive ECSIT phospho-state, including T179, links EPHA2 signaling to mitochondrial ECSIT accumulation.

We hope that the revised manuscript and accompanying files now fully address all remaining editorial requirements. We sincerely thank you again for your guidance throughout the review and editorial process.

Point-by-point response to the Reviewers

We sincerely thank the referees for their positive evaluation of our revised manuscript and for their helpful final suggestions. We have carefully addressed the remaining minor comments from Referee #1, as detailed below.

Referee #1

Comment 1.
The authors have adequately addressed my concerns and I suggest only a few minor changes: In line 67, the authors state that Epha2 expression peaked at E13.5. But this is the first time point they check, and therefore the peak of expression cannot be determined (could be earlier).

We thank the referee for this important clarification. We have revised the sentence to avoid overinterpretation and now state that EPHA2 expression was highest at E13.5 among the examined time points and declined thereafter (page 4, lines 67-69).

Comment 2.
In Figure EV5C, cells and not biological “ns” are displayed and quantified, please correct.

We thank the referee for pointing this out. We have corrected the figure legend and the corresponding description for the previous Figure EV5C, which has now been moved to Figure 4C following the editorial request, to indicate that cells are displayed and quantified.

Comment 3.
The proteomics data in Figure 3 improved by removing the outlier. It could be interesting to show what drives PC1, as it seems to be the main difference between the conditions, presumably mitochondria-related genes.

We visualized and showed the top 30 contributors from the PCA loading of PC1 following outlier removal, as shown below. PC1 is predominantly driven by mitochondrial proteins spanning multiple mitochondrial functional categories, including OXPHOS-related proteins such as NDUFA11, NUBPL, and ATPAF1. The majority of the top PC1 contributors are statistically significant, confirming that the separation pattern between the EPHA2 WT and 5A conditions along PC1 reflects mitochondrial biology.

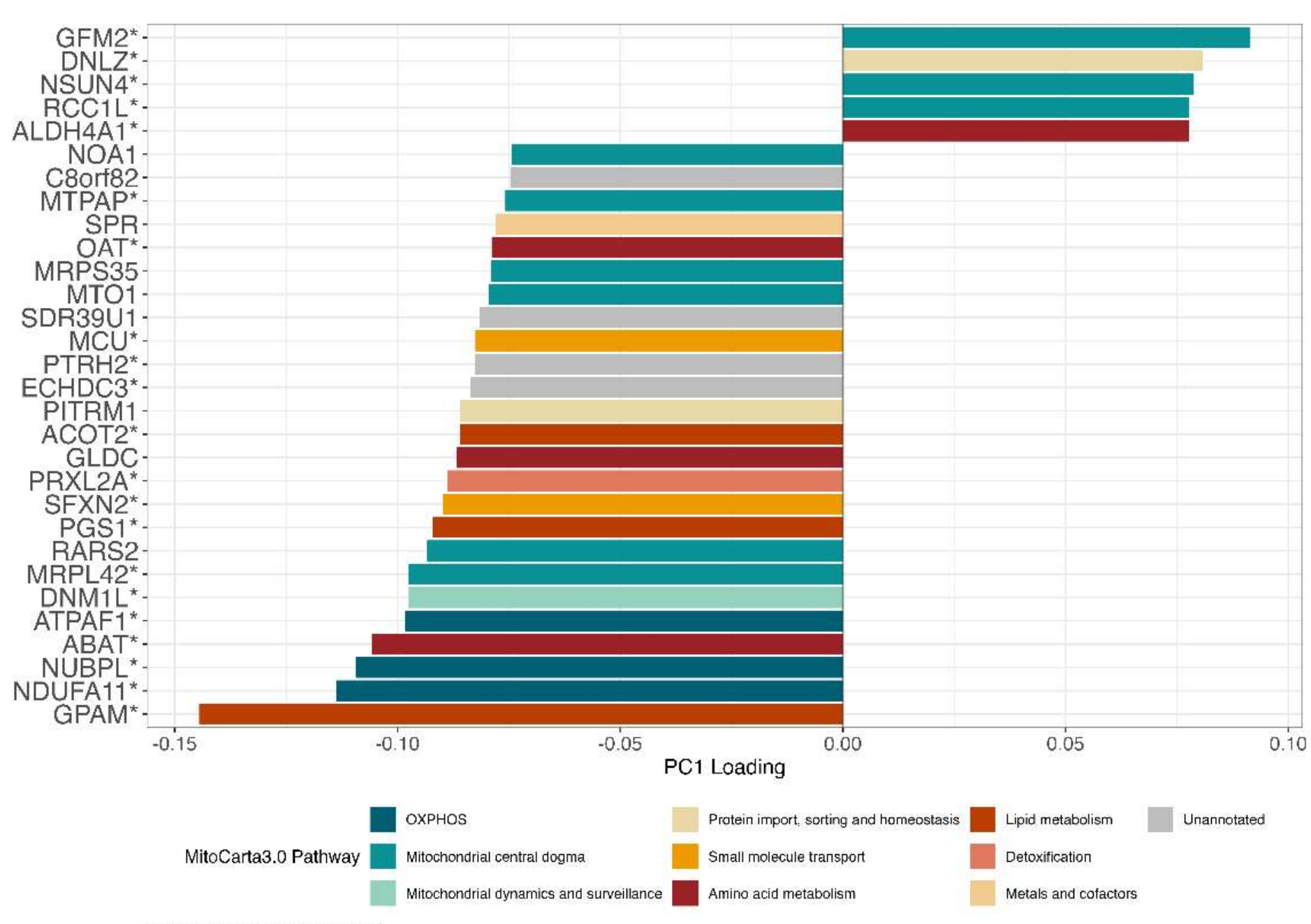

Referee #3

Comment.
This manuscript is acceptable for publication in EMBO Reports. All issues pertaining to my concerns have been fully addressed.

We sincerely thank the referee for the positive evaluation of our revised manuscript. We are pleased that the referee considers the manuscript acceptable for publication and that all previous concerns have been fully addressed.

Prof. Sang Ki Park
Pohang University of Science and Technology
Department of Life Sciences
77 Cheongam-Ro. Nam-Gu
Pohang 37673
Korea, Republic of

Dear Prof. Park,

I am very pleased to accept your manuscript for publication in the next available issue of EMBO reports. Thank you for your contribution to our journal.

Your manuscript will be processed for publication by EMBO Press. It will be copy edited and you will receive page proofs prior to publication. Please note that you will be contacted by Springer Nature Author Services to complete licensing and payment information.

You may qualify for financial assistance for your publication charges - either via a Springer Nature fully open access agreement or an EMBO initiative. Check your eligibility: https://link.springer.com/journal/44319/how-to-publish-with-us

Should you be planning a Press Release on your article, please notify the production team via this form in order to coordinate publication and release dates: https://forms.cloud.microsoft/e/eH4A8JVP6g

If you have any questions, please do not hesitate to contact the Editorial Office. Thank you for your contribution to EMBO Reports.

Best regards,
Esther

Esther Schnapp, PhD
Senior Editor
EMBO reports

>>> Please note that it is EMBO Reports policy for the transcript of the editorial process (containing referee reports and your response letter) to be published as an online supplement to each paper. If you do NOT want this, you will need to inform the Editorial Office via email immediately. More information is available here: https://link.springer.com/partners/embo-press/editorial-policies#Peer%20review

EMBO Press Author Checklist

Corresponding Author Name: Sang Ki Park	
Journal Submitted to: EMBO Reports	
Manuscript Number: EMBOR-2026-64350-T	

USEFUL LINKS FOR COMPLETING THIS FORM
The EMBO Journal - Author Guidelines
EMBO Reports - Author Guidelines
Molecular Systems Biology - Author Guidelines
EMBO Molecular Medicine - Author Guidelines

Reporting Checklist for Life Science Articles (updated January
This checklist is adapted from Materials Design Analysis Reporting (MDAR) Checklist for Authors. MDAR establishes a minimum set of requirements in transparent reporting in the life sciences (see Statement of Task: 10.31222/osf.io/9sm4x). Please follow the journal's guidelines in preparing your
Please note that a copy of this checklist will be published alongside your article.

Abridged guidelines for figures
1. Data
The data shown in figures should satisfy the following conditions:
- the data were obtained and processed according to the field's best practice and are presented to reflect the results of the experiments in an accurate and unbiased manner.
- ideally, figure panels should include only measurements that are directly comparable to each other and obtained with the same assay.
- plots include clearly labeled error bars for independent experiments and sample sizes. Unless justified, error bars should not be shown for technical
- if n<5, the individual data points from each experiment should be plotted. Any statistical test employed should be justified.
- Source Data should be included to report the data underlying figures according to the guidelines set out in the authorship guidelines on Data

2. Captions
Each figure caption should contain the following information, for each panel where they are relevant:
- a specification of the experimental system investigated (eg cell line, species name).
- the assay(s) and method(s) used to carry out the reported observations and measurements.
- an explicit mention of the biological and chemical entity(ies) that are being measured.
- an explicit mention of the biological and chemical entity(ies) that are altered/varied/perturbed in a controlled manner.
- the exact sample size (n) for each experimental group/condition, given as a number, not a range;
- a description of the sample collection allowing the reader to understand whether the samples represent technical or biological replicates (including how many animals, litters, cultures, etc.).
- a statement of how many times the experiment shown was independently replicated in the laboratory.
- definitions of statistical methods and measures:
  - common tests, such as t-test (please specify whether paired vs. unpaired), simple $\chi 2$ tests, Wilcoxon and Mann-Whitney tests, can be unambiguously identified by name only, but more complex techniques should be described in the methods section;
  - are tests one-sided or two-sided?
  - are there adjustments for multiple comparisons?
  - exact statistical test results, e.g., P values = x but not P values < x;
  - definition of 'center values' as median or average;
  - definition of error bars as s.d. or s.e.m.

Please complete ALL of the questions below.
Select "Not Applicable" only when the requested information is not relevant for your study.

Materials

Newly Created Materials	Information included in the manuscript?	In which section is the information available? (Reagents and Tools Table, Materials and Methods, Figures, Data Availability Section)
New materials and reagents need to be available; do any restrictions apply?	Not Applicable	

Antibodies	Information included in the manuscript?	In which section is the information available? (Reagents and Tools Table, Materials and Methods, Figures, Data Availability Section)
For **antibodies** provide the following information:<br>- Commercial antibodies: RRID (if possible) or supplier name, catalogue number and or/clone number<br>- Non-commercial: RRID or citation	Yes	Reagents and Tools Table

DNA and RNA sequences	Information included in the manuscript?	In which section is the information available? (Reagents and Tools Table, Materials and Methods, Figures, Data Availability Section)
**Short novel DNA or RNA including primers, probes**: provide the sequences.	Yes	Reagents and Tools Table

Cell materials	Information included in the manuscript?	In which section is the information available? (Reagents and Tools Table, Materials and Methods, Figures, Data Availability Section)
**Cell lines:** Provide species information, strain. Provide accession number in repository **OR** supplier name, catalog number, clone number, and/**OR** RRID.	Yes	Reagents and Tools Table
**Primary cultures:** Provide species, strain, sex of origin, genetic modification status.	Yes	Reagents and Tools Table
Report if the cell lines were recently **authenticated** (e.g., by STR profiling) and tested for mycoplasma contamination.	Yes	Methods

Experimental animals	Information included in the manuscript?	In which section is the information available? (Reagents and Tools Table, Materials and Methods, Figures, Data Availability Section)
**Laboratory animals or Model organisms:** Provide species, strain, sex, age, genetic modification status. Provide accession number in repository **OR** supplier name, catalog number, clone number, **OR** RRID.	Yes	Reagents and Tools Table
**Animal observed in or captured from the field:** Provide species, sex, and age where possible.	Not Applicable	
Please detail **housing and husbandry conditions**.	Yes	Methods

Plants and microbes	Information included in the manuscript?	In which section is the information available? (Reagents and Tools Table, Materials and Methods, Figures, Data Availability Section)
**Plants:** provide species and strain, ecotype and cultivar where relevant, unique accession number if available, and source (including location for collected wild specimens).	Not Applicable	
**Microbes:** provide species and strain, unique accession number if available, and source.	Not Applicable	

Human research participants	Information included in the manuscript?	In which section is the information available? (Reagents and Tools Table, Materials and Methods, Figures, Data Availability Section)
If collected and within the bounds of privacy constraints report on age, sex and gender or ethnicity for all study participants.	Not Applicable	

Core facilities	Information included in the manuscript?	In which section is the information available? (Reagents and Tools Table, Materials and Methods, Figures, Data Availability Section)
If your work benefited from core facilities, was their service mentioned in the acknowledgments section?	Not Applicable	

Design

Study protocol	Information included in the manuscript?	In which section is the information available? (Reagents and Tools Table, Materials and Methods, Figures, Data Availability Section)
If study protocol has been **pre-registered, provide DOI in the manuscript**. For clinical trials, provide the trial registration number **OR** cite DOI.	Not Applicable	
Report the **clinical trial registration number** (at ClinicalTrials.gov or equivalent), where applicable.	Not Applicable	

Laboratory protocol	Information included in the manuscript?	In which section is the information available? (Reagents and Tools Table, Materials and Methods, Figures, Data Availability Section)
Provide DOI OR other citation details if **external detailed step-by-step protocols** are available.	Yes	Methods

Experimental study design and statistics	Information included in the manuscript?	In which section is the information available? (Reagents and Tools Table, Materials and Methods, Figures, Data Availability Section)
Include a statement about **sample size** estimate even if no statistical methods were used.	Yes	Methods
Were any steps taken to minimize the effects of subjective bias when allocating animals/samples to treatment (e.g. **randomization procedure**)? If yes, have they been described?	Not Applicable	
Include a statement about **blinding** even if no blinding was done.	Yes	Methods
Describe **inclusion/exclusion criteria** if samples or animals were excluded from the analysis. Were the criteria pre-established?<br><br>If sample or data points were omitted from analysis, report if this was due to attrition or intentional exclusion and provide justification.	Not Applicable	
For every figure, are **statistical tests** justified as appropriate? Do the data meet the assumptions of the tests (e.g., normal distribution)? Describe any methods used to assess it. Is there an estimate of variation within each group of data? Is the variance similar between the groups that are being statistically compared?	Yes	Figure Legends

Sample definition and in-laboratory replication	Information included in the manuscript?	In which section is the information available? (Reagents and Tools Table, Materials and Methods, Figures, Data Availability Section)
In the figure legends: state number of times the experiment was **replicated** in laboratory.	Yes	Figure Legends
In the figure legends: define whether data describe **technical or biological replicates**.	Yes	Figure Legends

Ethics

Ethics	Information included in the manuscript?	In which section is the information available? (Reagents and Tools Table, Materials and Methods, Figures, Data Availability Section)
Studies involving **human participants**: State details of **authority granting ethics approval** (IRB or equivalent committee(s), provide reference number for approval.	Not Applicable	
Studies involving **human participants**: Include a statement confirming that **informed consent** was obtained from all subjects and that the experiments conformed to the principles set out in the WMA Declaration of Helsinki and the Department of Health and Human Services Belmont Report.	Not Applicable	
Studies involving **human participants:** For publication of **patient photos**, include a statement confirming that consent to publish was obtained.	Not Applicable	
Studies involving experimental **animals**: State details of **authority granting ethics approval** (IRB or equivalent committee(s), provide reference number for approval. Include a statement of compliance with ethical regulations.	Yes	Methods
Studies involving **specimen and field samples:** State if relevant **permits** obtained, provide details of authority approving study; if none were required, explain why.	Not Applicable	

Dual Use Research of Concern (DURC)	Information included in the manuscript?	In which section is the information available? (Reagents and Tools Table, Materials and Methods, Figures, Data Availability Section)
Could your study fall under dual use research restrictions? Please check biosecurity documents and list of **select agents and toxins** (CDC): https://www.selectagents.gov/sat/list.htm	Not Applicable	
If you used a select agent, is the security level of the lab appropriate and reported in the manuscript?	Not Applicable	
If a study is subject to dual use research of concern regulations, is the name of the **authority granting approval and reference number** for the regulatory approval provided in the manuscript?	Not Applicable	

Reporting

The MDAR framework recommends adoption of discipline-specific guidelines, established and endorsed through community initiatives. Journals have their own policy about requiring specific guidelines and recommendations to complement MDAR.

Adherence to community standards	Information included in the manuscript?	In which section is the information available? (Reagents and Tools Table, Materials and Methods, Figures, Data Availability Section)
State if relevant guidelines or checklists (e.g., **ICMJE, MIBBI, ARRIVE, PRISMA**) have been followed or provided.	Not Applicable	
For **tumor marker prognostic studies,** we recommend that you follow the **REMARK** reporting guidelines (see link list at top right). See author guidelines, under 'Reporting Guidelines'. Please confirm you have followed these guidelines.	Not Applicable	
For **phase II and III randomized controlled trials**, please refer to the **CONSORT** flow diagram (see link list at top right) and submit the CONSORT checklist (see link list at top right) with your submission. See author guidelines, under 'Reporting Guidelines'. Please confirm you have submitted this list.	Not Applicable	

Data Availability

Data availability	Information included in the manuscript?	In which section is the information available? (Reagents and Tools Table, Materials and Methods, Figures, Data Availability Section)
Have **primary datasets** been deposited according to the journal's guidelines (see 'Data Deposition' section) and the respective accession numbers provided in the Data Availability Section?	Yes	Data Availability Section
Were **human clinical and genomic datasets** deposited in a public access-controlled repository in accordance to ethical obligations to the patients and to the applicable consent agreement?	Not Applicable	
Are **computational models** that are central and integral to a study available without restrictions in a machine-readable form? Were the relevant accession numbers or links provided?	Not Applicable	
If publicly available data were reused, provide the respective **data citations in the reference list**.	Yes	Reference
