# Supplementary material for: Ligand-independent EPHA2 signaling sustains neural progenitors via ECSIT and NAD+
Source: EMBO Rep. 2026 Jul 10;27(16):4782–823. doi: 10.1038/s44319-026-00858-6 (PMC13503776; doi:10.1038/s44319-026-00858-6)
Supplement: Supplementary file 3 [file 44319_2026_858_MOESM2_ESM.zip › 1B/epha2.pdf]

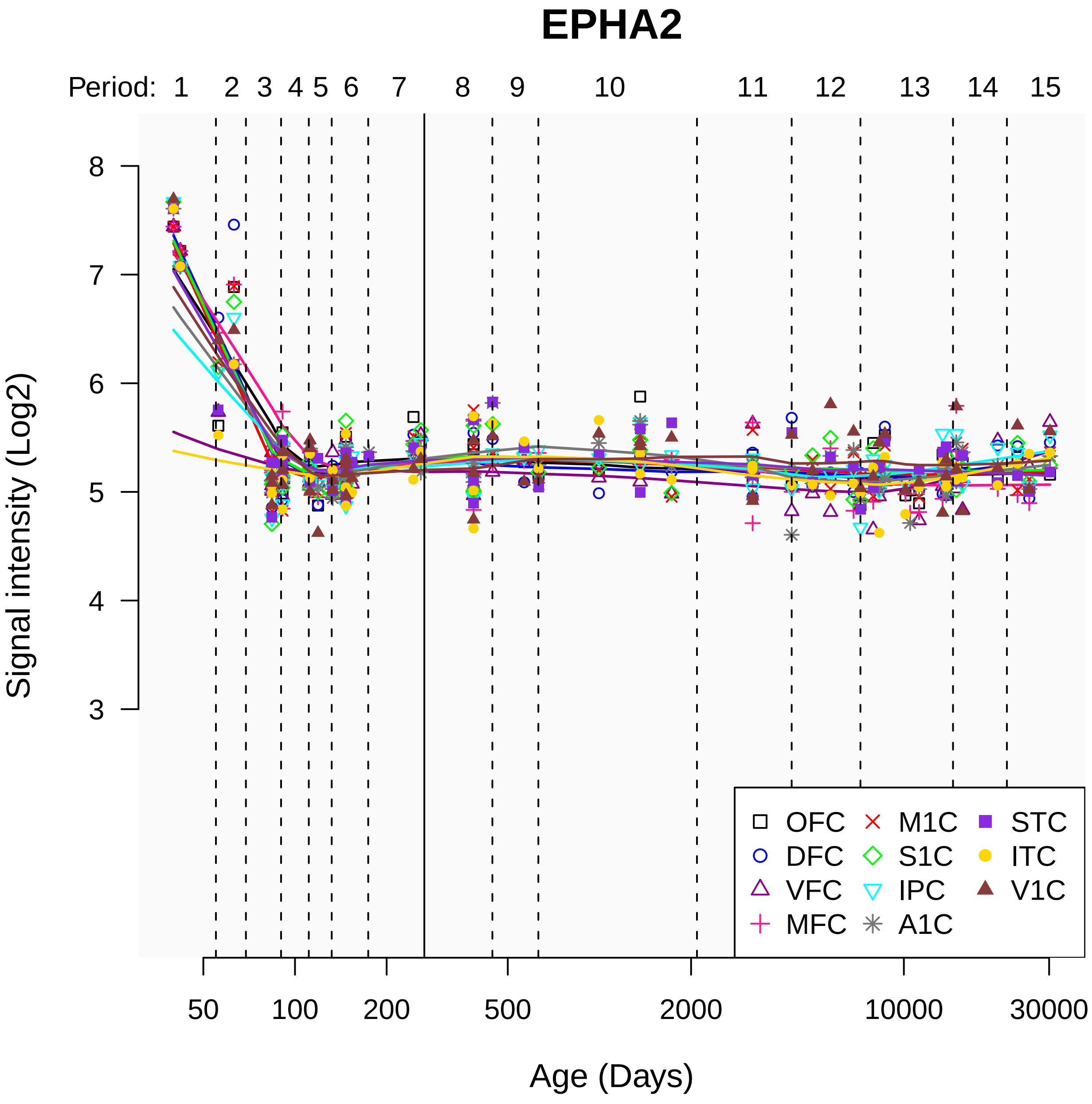

EPHA2
Period: 1 2 3 4 5 6 7 8 9 10 11 12 13 14 15
Signal intensity (Log2)
8
7
6
5
4
3
50
100
200
500
2000
10000
30000
Age (Days)
OFC
DFC
VFC
MFC
M1C
S1C
IPC
A1C
STC
ITC
V1C
