# Supplementary material for: Ligand-independent EPHA2 signaling sustains neural progenitors via ECSIT and NAD+
Source: EMBO Rep. 2026 Jul 10;27(16):4782–823. doi: 10.1038/s44319-026-00858-6 (PMC13503776; doi:10.1038/s44319-026-00858-6)
Supplement: Supplementary file 6 [file 44319_2026_858_MOESM5_ESM.zip › 4A/4A.pdf]

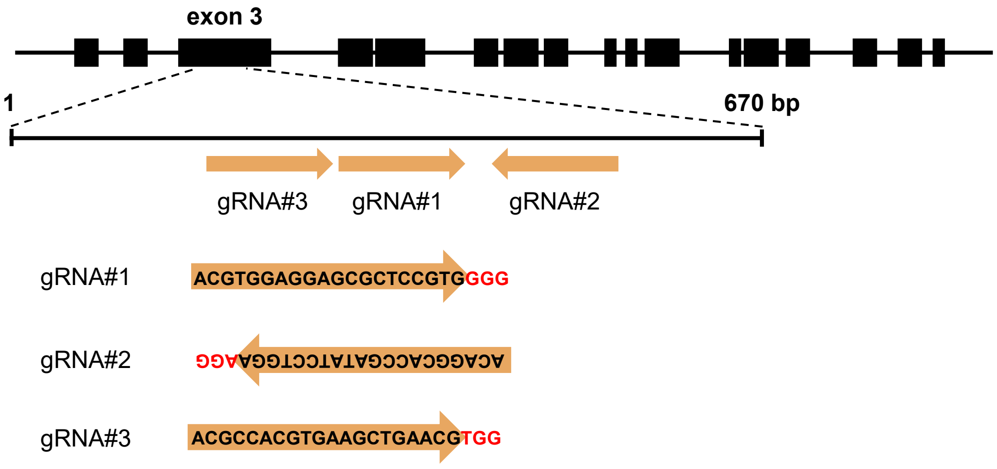
exon 3
1
670 bp
gRNA#3
gRNA#1
gRNA#2
gRNA#1
ACGTGGAGGAGCGCTCCGTGGGG
gRNA#2
ACAGGCACCGATATCCTGGAAGG
gRNA#3
ACGCCACGTGAAGCTGAACGTGG
